# Proteome analysis of *Ehrlichia chaffeensis* containing phagosome membranes revealed the presence of numerous bacterial and host proteins

**DOI:** 10.3389/fcimb.2022.1070356

**Published:** 2022-12-23

**Authors:** Chandramouli Kondethimmanahalli, Roman R. Ganta

**Affiliations:** Center of Excellence for Vector-Borne Diseases, Department of Diagnostic Medicine/Pathobiology, College of Veterinary Medicine, Kansas State University, Manhattan, KS, United States

**Keywords:** tick-borne pathogens, rickettsials, obligate pathogens, phagosome proteome, *Ehrlichia*

## Abstract

Tick-transmitted *Ehrlichia chaffeensis*, the causative agent for human monocytic ehrlichiosis, resides and multiplies within a host cell phagosome. Infection progression of *E. chaffeensis* includes internalization into a host cell by host cell membrane fusion events following engulfment leading to the formation of *E. chaffeensis* containing vacuole (ECV). Revealing the molecular composition of ECV is important in understanding the host cellular processes, evasion of host defense pathways and in defining host-pathogen interactions. ECVs purified from infected host cells were analyzed to define both host and bacterial proteomes associated with the phagosome membranes. About 160 bacterial proteins and 2,683 host proteins were identified in the ECV membranes. The host proteins included predominantly known phagosome proteins involved in phagocytic trafficking, fusion of vesicles, protein transport, Ras signaling pathway and pathogenic infection. Many highly expressed proteins were similar to the previously documented proteins of phagosome vacuole membranes containing other obligate pathogenic bacteria. The finding of many bacterial membrane proteins is novel; they included multiple outer membrane proteins, such as the p28-Omps, the 120 kDa protein, preprotein translocases, lipoproteins, metal binding proteins, and chaperonins, although the presence of ankyrin repeat proteins, several Type I and IV secretion system proteins is anticipated. This study demonstrates that ECV membrane is extensively modified by the pathogen. This study represents the first and the most comprehensive description of ECV membrane proteome. The identity of many host and *Ehrlichia* proteins in the ECV membrane will be a valuable to define pathogenic mechanisms critical for the replication of the pathogen within macrophages.

## Introduction

*Ehrlichia chaffeensis* is an emerging obligate intracellular pathogen transmitted from *Amblyomma americanum* tick to people and causing human monocytic ehrlichiosis (HME). This pathogen infections are also documented in deer (the reservoir host), dogs, goats, coyotes, and cattle (Dumler and Bakken, 1995; Parola and Raoult, 2001; Walker et al., 2008). *E. chaffeensis* infection, as a dense core form, requires its internalization into a host cell by membrane fusion events occurring between the bacterial vacuole and the host endocytic vesicles (Li et al., 2010). After internalization, *E*. *chaffeensis* develops the ability to subvert the host clearance by residing in an early phagosome. Indeed, host membrane is actively modified by insertion or trafficking of bacterial proteins, such as the proteins associated with Type I and IV secretion systems. These events lead to the formation of cytoplasmic phagosome with the prevention of phagolysosome fusion to allow *E. chaffeensis* replication as larger reticulate cells and then transforming to smaller infectious dense core cells before releasing from the infected host cells (Griffiths and Mayorga, 2007; Rogers and Foster, 2008; McBride and Walker, 2011; Dedonder et al., 2012; Eedunuri et al., 2018). In particular, *E. chaffeensis* phagosome rapidly fuses with host-cell endosomes acquiring early-endosome markers such as Rab5 and effectors proteins. Revealing the molecular composition of phagosome membranes will greatly aid in understanding host-pathogen interactions, host cellular processes and evasion of host defense pathways by the pathogen (Corsaro et al., 1999; Taylor et al., 2003; Au et al., 2007; O’Callaghan and Stebbins, 2010). Such studies have previously demonstrated the critical contributions of membrane proteins in triggering the host immune response and protection of the host from infection. A number of cellular membrane proteins have been described to associate with *E. chaffeensis* (McBride and Walker, 2011; Eedunuri et al., 2018). For example, *Ehrlichia* specific major surface proteins and immunogenic proteins are identified in the bacterial membranes recovered from macrophage and tick cells by LC/MS/MS analysis (Seo et al., 2008). Major surface proteins including OMP-/P28 family proteins and T4SS secretary proteins were detected in *E*. *chaffeensis* infected with human monocytic leukemia THP-1 cells (McBride and Walker, 2011).

Proteome characterization of bacteria containing phagosomes has advanced understanding of infectious diseases caused by several obligate intracellular pathogens (Mills and Finlay, 1998; Shevchuk et al., 2009; Urwyler et al., 2009; Hoffmann et al., 2014). For example, proteomes of *Yersinia pseudotubercuolosis*, *Salmonella typhimurium*, *Legionella pneumophila*, *Chlamydia trachomatis* and *Dictyostelium discoideum*-containing vacuoles have been analyzed and the studies continue to aid greatly in defining many proteins required for infection progressions and transmissions (Mills and Finlay, 1998; Shevchuk et al., 2009; Urwyler et al., 2009; Hoffmann et al., 2014).

Considering the crucial role of proteins on the phagosome membrane for a pathogen’s infection and transmission (Bruckert and Kwaik, 2015), we investigated in defining proteins associated with *Ehrlichia*-containing vacuole (ECV) membrane. Adopting more sensitive proteomic methods allows defining the presence of widely dynamic range of bacterial membrane proteins being composed in the phagosome membrane fractions (Li et al., 2010; Hartlova et al., 2011; Schmidt and Völker, 2011). ECV membrane composition was analyzed by mass spectrometry with a limited success earlier with a primary focus on defining the host proteins (Cheng et al., 2014). We purified phagosomes from infected host cells containing *E. chaffeensis* by employing effective cell-lysis protocol and by taking advantage of the sucrose density gradient centrifugation method of fractionation, as similar method is previously reported for the *Legionella* containing phagosome purification (Bruckert and Kwaik, 2015) and that we adopted the method for *Ehrlichia-*containing phagosome purification (Zhang et al., 2021). This study allowed the identification of about 2,683 proteins of host origin and about 160 proteins of the pathogen origin.

## Materials and methods

### Host cells culture, infection, and cultivation of *E. chaffeensis*

*E. chaffeensis* Arkansas isolate was grown in host cells as described earlier (Cheng and Ganta, 2010; Kondethimmanahalli and Ganta, 2018; Kondethimmanahalli et al., 2019). Briefly, HL-60 cells were infected with *E. chaffeensis* in a T25 flask containing minimum essential medium (MEM) supplemented with 10% serum at 37°C in 5% CO_2_ and the infection rate was assessed by Diff-Quik Kit staining (Baxter Scientific Products, Obetz, Ohio). Infected host cells with *E. chaffeensis* organisms were harvested by centrifugation at 400 x g for 10 min at 4°C and pelleted cells were disrupted with glass beads by vortexing twice for 30 sec followed by removal of cell debris by centrifugation. The supernatant containing *E. chaffeensis* was passed through a 2.7 µm syringe filter (Whatman, Pittsburgh, PA) and the filtrate was used for reinfecting cells cultured in a T150 flask. After 72 h, the infection was assessed by Diff-Quik staining and phagosomes in the morula were observed under microscope.

### *E. chaffeensis* containing vacuole purification

Infected host cell cultures were used for phagosome purification as we recently described (Zhang et al., 2021) except that we eliminated the magnetic capture method with Rab5 antibody, as it had a little impact in the phagosome purity. Briefly, infected cells were washed with PBS and with homogenization buffer (250 mM sucrose, 0.5 mM EGTA, 20 mM HEPES/KOH, protease inhibitor, pH 7.2) and scraped into a 50 mL tube and pelleted at 500 x g for 5 min at 4°C. Cells were resuspended in homogenization buffer and homogenized on using a 10 ml syringe with a 23 G needle (about 10–12 strokes). After ~80% of cell lysing, the phagosome integrity was checked under microscopy. Unbroken cells and nuclei were pelleted by centrifugation at 300 x g for 5 min at 4°C. The resulting post nuclear supernatant (PNS) was brought to 39% final concentration of sucrose. This phagosome suspension (1.5 ml) was gently layered on top of a sucrose gradient consisting of 65% sucrose (2 ml), 55% sucrose (2 ml). Above the PNS layer, 1 ml of 25% sucrose carefully layered followed by 1 ml of 10% sucrose. The gradient was then centrifuged at 100,000 x g for 1 h at 4°C in a swinging bucket rotor (Beckman SW41) using Beckman tabletop ultra centrifuge. *E. chaffeensis* phagosomes, which were in the 55–65% interface, were carefully collected and mixed with HB buffer (0.5 mM EGTA, 20 mM HEPES/KOH, pH 7.2) to a final sucrose concentration of 11% and centrifuged at 18,000 x g for 30 min at 4°C.

### Protein extraction, sodium dodecyl sulfate (SDS)-polyacrylamide gel electrophoresis

Purified phagosomes were solubilized in 1% Triton X-100 in PBS for 30 min on ice and centrifuged at 10,000 x g for 5 min to pellet bacteria and the supernatant containing ECV membranes was used for SDS-PAGE and proteomic experiments. For SDS-PAGE, proteins were precipitated and purified using a Readyprep 2D cleanup kit (BioRad, Hercules, CA) and then quantified using Bradford protein assay kit (BioRad, Hercules, CA). Twenty micrograms of proteins each from three replicates were suspended in SDS sample buffer (Invitrogen, Carlsbad, CA) and boiled for 5 min and then separated using a Mini-PROTEAN Precast TGX gel with 4% to 15% polyacrylamide gradient (Bio-Rad, Hercules, CA) at 100 V for 90 min. The proteins on the gels were stained using a Novex Colloidal Blue stain kit (Invitrogen, Carlsbad, CA).

### LC-MS/MS analysis of phagosome proteins

LC-MS/MS analysis was performed at the UCLA Proteome Research Center (https://uccore.org/ucla-proteome-research-center/). Two hundred micrograms of ECV membrane proteins each from three replicates were reduced with 5 mM Tris (2-carboxyethyl) phosphine buffer (pH 7.0) and alkylated using 10 mM iodoacetamide and diluted 7 times with 100 mM ammonium bicarbonate. Proteins were subsequently digested with trypsin 1:50 enzyme-protein ratio (Promega, Madison, WI) and Endoproteinase Lys-C 37°C as described in (Florens et al., 2006; Wohlschlegel, 2009). Digestions were stopped with the addition of 5% formic acid and then desalted using Pierce C18 tips (Thermo Fisher Scientific) and vacuum-dried. Peptides were fractionated using a 25 cm long, 75 µM inner diameter fused silica capillary column packed in-house with bulk C18 reversed phase resin in buffer A (water with 3% DMSO and 0.1% formic acid) and buffer B (acetonitrile with 3% DMSO and 0.1% formic acid). A 140 min increasing gradient of 5% to 80% acetonitrile was delivered using a Dionex Ultimate 3000 UHPLC system (Thermo Fisher Scientific) at a flow rate of 300 nl/min. Peptides were ionized using a distal 2.2 kV spray voltage and a capillary temperature of 275°C and electrosprayed into an Orbitrap Fusion Lumos mass spectrometer (Thermo Fisher Scientific) where fragment ions were analyzed by tandem mass spectrometry (MS/MS). Data were acquired using a Data-Dependent Acquisition (DDA) strategy comprised of a full MS1 scan at 120,000 resolution followed by sequential MS2 scans (Resolution = 15,000) in 3 second cycle time.

### Mass spectrometry data analysis

Data analysis was performed using the Max Quant and Perseus software packages (Cox and Mann, 2008). Peptide and protein identifications were generated by the Andromeda search engine after searching against uniprot human database (UP000005604) and *E. chaffeensis* protein databases (Taxonomy ID 943, GenBank Accessions no. CP000236). Peptide/protein quantitation was determined by MS1-based quantitation of chromatographic peak areas. Maxquant intensity data was imported into the Perseus (Tyanova et al., 2016) algorithm which was used to impute missing values and determine which proteins were differentially abundant across samples using a two-tailed T-test. Multiple hypothesis corrections were performed using the Benjamini-Hochberg correction.

### SDS-PAGE and Western blot analysis

Twenty micrograms of ECV membrane proteins were separated on polyacrylamide gels as described above. For the detection of *E*. *chaffeensis* Dnak and p28‐Omp 19 proteins, the above-described electrophoresed proteins were transferred onto a 0.45 µm nitrocellulose membrane (Thermo Fisher Scientific, Rockford, IL) and subjected to blotting using p28 monoclonal antibodies and polyclonal rabbit antisera raised against recombinant *E*. *chaffeensis* protein DnaK, respectively (Zhang et al., 2013). For the detection of host membrane proteins Rab5 and Lamin B, anti-rabbit Rab5 and anti-rabbit Lamin B antibodies (1:1000) (Cell Signaling Technology, 110 Danvers, MA) were used, respectively. Secondary antibodies conjugated with horseradish peroxidase (Sigma-Aldrich, St. Louis, MO, USA) were used. ECL Western blotting detection reagents (Amersham, Buckinghamshire, UK) were used for the signal detection.

### Confocal microscopy

Infected *E. chaffeensis* were plated onto an 8-well culture chamber slide and fixed with 4% paraformaldehyde for 15 min, and permeabilized with 0.1% triton-X-100 and then blocked with 3% BSA. The cells were incubated overnight at 4°C with *E. chaffeensis* specific anti-p28 Omp 19 monoclonal antibody, 18.1 (1:500 dilution) and Rab 5 (1:200) antibodies. The slides were washed with PBS and incubated 1h with Alexa Flour 594 goat anti-mouse or Alexa Flour goat anti-rabbit 488 secondary antibodies (Invitrogen). The slides were washed and mounted with DAPI Fluor mount (Southern Biotech, Birmingham, AL). Slides were visualized with a Carl Zeiss 700 laser scanning confocal microscopy (Carl Zeiss Optronics GmbH, Oberkochen, Germany) and images were acquired using ZEN software and analyzed using ImageJ software.

## Results

### *Ehrlichia*-containing phagosome (ECV) purification and verification

**Figure 1:**
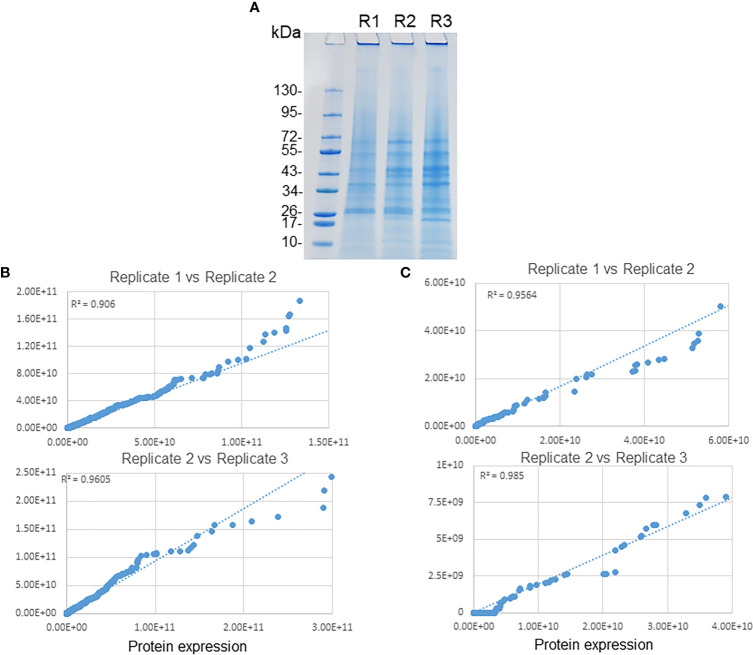
Replicates of phagosome membrane proteins of *E*. *chaffeensis*. **(A)** Purified phagosome membranes from three replicate samples were separated by SDS-PAGE followed by staining with colloidal blue stain. Scatter plots for the protein expression data comparison of replicate samples. Proteins associated with host phagosome membranes **(B)** and *E*. *chaffeensis* **(C)** among replicates revealed a higher degree of correlation.

We monitored the infection from 24 to 72 h by Diff-Quick staining. At 72 h, we observed the highest ECV numbers when compared to earlier time points in the HL-60 cells. We utilized effective cell lysis protocol followed by sucrose gradient ultracentrifugation to purify *E. chaffeensis* containing phagosomes from the infected HL-60 cells as described in our previous study ( (Zhang et al., 2021). Subsequently, the purified phagosomes were lysed and *Ehrlichia*-free phagosome membranes were recovered and subjected to proteome analysis. To obtain comprehensive coverage of phagosome proteome, we performed LC-MS/MS mass spectrometry analysis of the purified phagosome membrane fractions, and the data were used for database searches against human genome and *E. chaffeensis* genome databases. The raw spectrum data and search engine files were submitted in the Mass Spectrometry Interactive Virtual Environment (MassIVE) repository; accession number MassIVE MSV000090638 at https://massive.ucsd.edu/ProteoSAFe/dataset.jsp. To increase the phagosome protein identification, we used dual protein digestion and fractionated peptides to sub fractions and then separated on a C18 column with long gradient of 140 min. Subsequently, peptides were ionized using electrospray and detected using highly sensitive and high resolution orbitrap mass spectrometry. These experiments allowed the identification of large numbers of host and *Ehrlichia* proteins in the ECV membranes with a high degree of correlation between the three replicates and having more than 75% of protein identified by >2 unique peptides and a minimum of 10% sequence coverage. These analysis performed from three replicate samples (**Figure 1A**) resulted in the identification of 2,683 host proteins (**Table S2**) and 160 *E. chaffeensis* proteins (**Table S1**). A protein is regarded as expressed only when two or more unique peptides were detected. Seventy percent of *Ehrlichia* proteins had more than 10% sequence coverage (**Figure S1A**). Similarly, 81% of identified host proteins had more than 10% of sequence coverage (**Figure S1B**). The overall protein expression of both host and *Ehrlichia* proteins were compared to assess the reproducibility among the replicates (**Figures 1B, C**). The scattered plots of host phagosomal proteins expression (R² = >0.9) and *Ehrlichia* proteins expression (R² = >0.94) showed a very high degree of correlation among replicates (**Figures 1B, C**, respectively).

**Figure 2:**
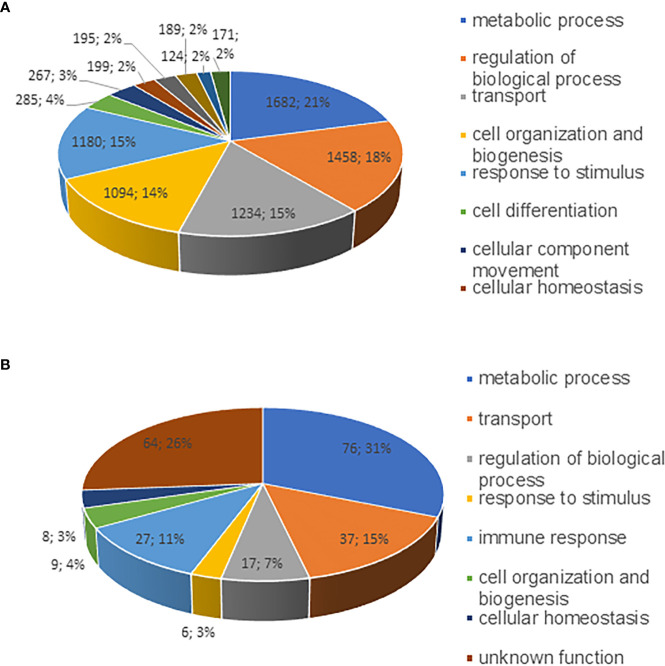
Pie charts representing proteins identified as various functional groups. Proteins identified from phagosome membranes were separated into as per their biological functional groups; **(A)** host membrane proteins and **(B)** *E*. *chaffeensis* proteins associated with the phagosome membrane.

Distribution of the identified proteins into different categories of biological process is shown in **Figure 2**. Gene ontology cellular component analysis showed that the predominant proteins from both host and bacterial origin belonged to host and bacterial membranes and membrane-associated proteins (**Figures S2A and B**). In the host proteome, a sum of 1,682 (21%), 1,458 (18%), 1,234 (15%), 1,094 (14%), and 1,180 (15%) proteins belonged to biological process category of metabolic process, regulation of biological process, transport processes, cellular organization, and response to stimulus, respectively (**Figure 2A**). The identified *E. chaffeensis* proteins included in the categories belonged to metabolic process; 76 (31%), transport processes; 37 (15%), and immune response; 27 (11%) (**Figure 2B**). The highly expressed proteins in the host phagosome membrane included several well-defined phagosome markers, such as several Rab proteins, calnexin, several endoplasmic reticulum proteins, lysosome-associated membrane glycoproteins (LAMP 1 and LAMP 2), several phosphoinositide lipids, actin-binding coat proteins belong to coronins. The highly expressed *E. chaffeensis* proteins within the host phagosome membrane included many outer membrane proteins, such as several p28 Omps, 120 kDa protein, Type I and Type V secretion system-associated proteins, lipoproteins, zinc and iron binding proteins, chaperon proteins, and efflux and preprotein transporter proteins.

### Validation of proteomics data

**Figure 3:**
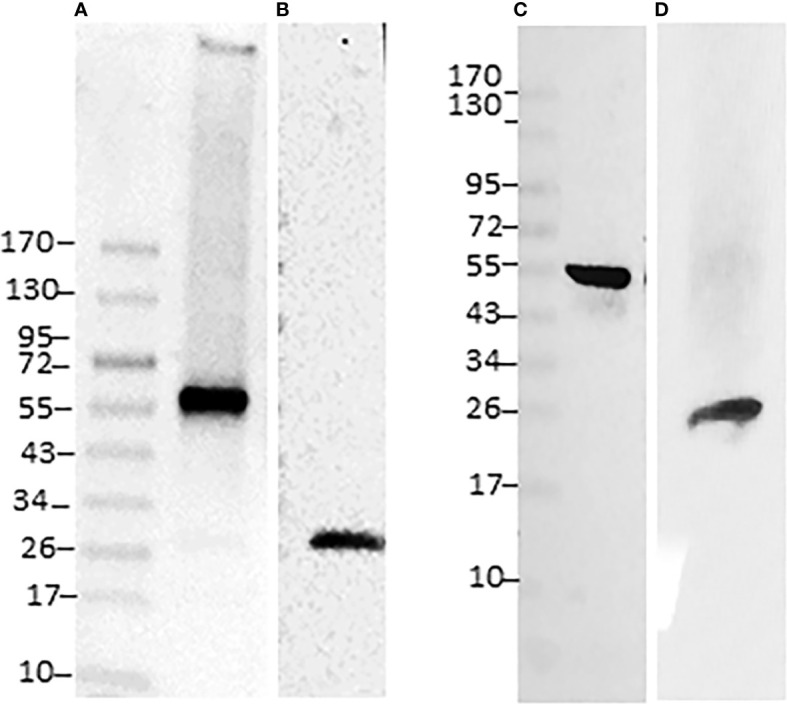
Immunoblotting of *E*. *chaffeensis* and host proteins identified in phagosome membranes. Twenty micrograms of proteins per lane were separated on a 4-20% SDS-PAGE gel, the resolved proteins were transferred to a nitrocellulose membrane and immunoblotted with *Ehrlichia* polyclonal antibodies specific to specific DnaK **(A)** and p28 monoclonal **(B)** or with host phagosome protein antibodies specific to lamin-B **(C)** and Rab5 **(D)**. Specific antigen-antibody interactions were visualized with horseradish peroxidase-conjugated secondary antibodies and detected using ECL.

To confirm the detection of selected proteins by proteomic datasets associated with host and pathogen, we performed Western blot analysis of purified proteins with two each of the host- and pathogen-specific specific antibodies, respectively. They included *Ehrlichia*-specific proteins DnaK (**Figure 3A**) and P28-Omp 19 (**Figure 3B**) and host-specific lamin-B (**Figure 3C**) and Rab5 (**Figure 3D**). Consistent with the proteome data, the antibodies recognized all four proteins in the phagosome membrane fractions.

### Proteome data further validated by confocal microscopy

**Figure 4:**
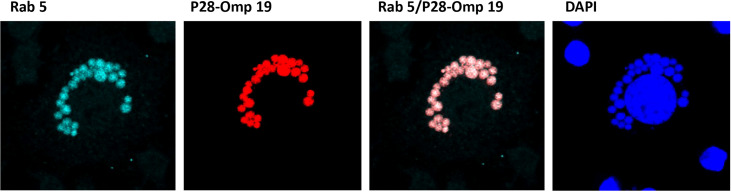
Confocal microscopy images of infected *E. chaffeensis.* Confocal microscopy was performed on *E. chaffeensis* infected cells with p28-Omp 19-specific monoclonal antibody 18.1 and phagosome membrane marker Rab5 antibody. Multiple images were collected and assessed for co-localization of *E. chaffeensis* p28-Omp and host Rab5 proteins.

To further confirm the translocation of *E. chaffeensis* proteins on to the ECV membrane, we performed confocal microscopy using *E. chaffeensis* p28-Omp 19-specific monoclonal antibody 18.1 (Li et al., 2001) and phagosome membrane-specific Rab5 antibody by following methods we previously described (Dedonder et al., 2012 and Zhang et al., 2021). Multiple confocal images were collected for *E. chaffeensis*-infected cultures grown to 48 h post infection and assessed; for co-localization (**Figure 4**). Both Rab5 and p28-Omp 19 antibody recognized the phagosomes containing *E. chaffeensis* inclusions. High degree of co-localization thresholds observed for Rab5 with p28-Omp, as estimated with the Pearson’s coefficient value of r = >0.4 for the multiple images assessed.

## Discussion

This study represents the first detailed analysis focused on defining the ECV membrane proteome. Our data were compared with the previously reported phagosomal membrane data in host vacuoles known to harbor bacterial pathogens, such as *Legionella pneumophila, Chlamydia trachomatis* and *Mycobacterium tuberculosis* (Clemens et al., 2000; Shevchuk et al., 2009; Li et al., 2010; Aeberhard et al., 2015; Bruckert and Kwaik, 2015). The majority of the phagosome proteins detected in the current study were very similar to the previously defined phagosome membranes of vacuoles containing bacterial pathogens (**Table S3**). The presence of highly expressed phagosome proteins in the current study is similar to the documentation of numerous proteins by previous studies, thus demonstrating that independent of the bacterial pathogens, phagosome membrane structures are predominantly similar (Bruckert and Kwaik, 2015) (**Table S4**). For example, as reported in the *Legionella*-containing vacuole membrane proteome (Bruckert and Kwaik, 2015), the ECV membrane is extensively constituted with ubiquitinated proteins. Quantitative proteomics and immunofluorescence microscopy analysis of *C. trachomatis* containing vacuoles (CCVs) identified 351 host cell proteins significantly enriched in the inclusion vacuole, of which 19 proteins were validated (Aeberhard et al., 2015). In another study, CCV membrane proteome analysis resulted in the identification of 2,231 host proteins and 440 C*. trachomatis* proteins (Herweg et al., 2015). Our current study detected similar numbers of host proteins (2,683) and lower numbers of bacterial proteins (160) as compared to the *C. trachomatis* study. We identified synaptogyrin-2 (SYNGR2), Ras-related protein Rab8A (Rab8A), Ras-related protein Rab3 (Rab3), syntaxin (STX) 1-8, STX 10-12 and STX 18, stromal interaction molecule 1 (STIM1L), and Vesicle-trafficking protein SEC22b as in CCV membrane (**Tables S2** and **S5**). Finding many host cell-derived phagosome proteins being common with *Ehrlichia-* and *Chlamydia*-infected host cells suggest that phagosome proteins for trafficking intracellularly for formation, infection and effectively undermining the host cellular defense factors are similar for these two obligate intraphagosomal bacteria. Previous studies, based on confocal microscopy and Western blot analysis, also reported that pathogen-containing vacuoles have large numbers of Ras-related proteins, cathepsin, annexin, calnexin and transferrin proteins localized to the bacterial-containing vacuoles (Li and Lostumbo, 2010; Aeberhard et al., 2015) (**Table S5**). Our finding of such proteins also on ECV membrane further demonstrate similarities for the pathogen-containing vacuole formations.

Several studies identified fewer phagosome membrane proteins for pathogen-containing phagosomes, probably because isolation of phagosomes in large quantities is challenging (Li et al., 2010; Herweg et al., 2015). Similarly, proteins identified for intracellular bacteria containing phagosome samples are typically lower than those obtained for pure bacterial cultures (Li and Lostumbo, 2010). To obtain large quantities of purified phagosomes, several technological challenges had to be met. We effectively lysed host cells and then purified the phagosomes be taking advantage of sucrose density gradient centrifugation method which we recently described (Zhang et al., 2021); it was adopted as per Bruckert and Kwaik, 2015. The purity was evaluated by imaging microscopy using phagosome membrane marker Rab5 antibody and *Ehrlichia*-specific p28 monoclonal antibody. Phagosome preparations were predominantly enriched with membrane or associated proteins as evidenced by cellular component analysis. With recent advances in the MS-based proteomic technologies (Trost et al., 2009; Wisniewski et al., 2009; Wisniewski et al., 2009), in depth analysis of both bacterial and host proteins associated with phagosome membranes was achieved and our current study represents the first for any rickettsial pathogen infecting a host.

We observed abundant expression of phagosome markers associated with ECV membrane. Among those Ras-related Rab proteins: Rab1, Rab2, Rab5, Rab7, Rab10 and Rab11 were predominant. Rab proteins are distinct molecular markers of phagosomes responsible for transport, recruitment, and fusion of vesicles (Zerial and McBride, 2001). Rab5 is the organizer of the protein machinery that assembles on early endosomes and endosomal fusion involved in transport, signaling, and progression of phagosomes to phagolysosomes (Vieira et al., 2003). Rab7 is expressed high in late endosomes and lysosomes (Garin et al., 2001; Stuart et al., 2007). Its presence on ECVs is previously confirmed by confocal and electron microscopy (Zhang et al., 2021). Rab5 and Rab7 expression levels change during phagosome maturation process. Rab7 and mannose-6 phosphate receptor (M6PR) proteins are characterized as late phagosome markers (Cheng et al., 2014). Rab2 and Rab10 play crucial roles in protein transport from the ER to the Golgi apparatus and phagolysosome formation respectively (Tisdale and Balch, 1996; Tisdale, 1999) and during phagosome maturation (Cardoso et al., 2010). The detection of transferrin, transferrin receptor, vacuolar-type H+-ATPase, and Rab5 confirm the previous evidence of the ECV is an early endosome (Rikihisa, 2017; Yan et al., 2018). Cathepsin D and lysosome-associated membrane proteins (LAMPs) are regarded as phagolysosome markers. The detection of Cathepsin D in our current study is unexpected if phagolysosomal fusion is not known to occur with *E. chaffeensis* infection. Cathepsin D has multiple functions, including its association with a plasma membrane (Sloane et al., 1986; Benes et al., 2008). The presence of this enzyme on the ECV membrane may likely suggest that a close association of lysosomes with ECVs. Indeed, we identified several subunits of vacuolar-type H+-ATPases which are known to function in acidification of functional phagosomes (Lukacs et al., 1990; Sturgill-Koszycki et al., 1994) and are required for phagosome maturation (Sturgill-Koszycki et al., 1994; Kinchen and Ravichandran, 2008). Further, we identified known phagosome proteins such as tubulins, syntaxin, protein transport protein Sec61 and calnexin that have been also previously reported by non-MS-based methods (Via et al., 1997). Thus, additional investigates are warranted regarding how the proteins such as Cathepsin D are associated with ECV if phagolysosome fusion is blocked by *E. chaffeensis* resides in an early endosomes, as previously suggested.

The ECV membrane proteome contained proteins crucial for stability and structure of the cell membrane and cytoskeleton reorganization; myosin-9, filamins, and tubulins, Talin1, Coronin-1, Ras GTPase-activating-like protein, and PLXNB2. Molecular chaperones are vital to cellular defense against general protein misfolding and facilitate the correct folding of imported proteins. Mannose 6 phosphate (M6P), protein transport protein Sec61, vesicle-trafficking protein SEC22b were present on the phagosome membrane, possibly implicating their role in transport of mitochondrial, endoplasmic reticulum and Golgi complex proteins to the phagosome membrane. Clathrins, annexins, and syntaxins are involved in intracellular trafficking of receptors, vesicle fusion and exocytosis (Bruckert and Kwaik, 2015; Herweg et al., 2015).

*Ehrlichia* proteins associated with host phagosome membrane included over 40 known bacterial membrane-associated proteins, which comprised of 21 OMP/P28 gene family proteins and several Type I and Type IV secretion system proteins. Several chaperon proteins, metal ion binding proteins, ankyrins and preprotein transporters were also among the proteins found in the ECV membranes. In our previous study, we purified *Ehrlichia* membranes, fractionated as total and immunogenic membrane proteins and then subjected to Liquid Chromatography-Tandem Mass Spectrometry and MALDI-TOF methods of fractionated proteins (Seo et al., 2008). That study identified 278 proteins in *E. chaffeensis* membrane fractions (Seo et al., 2008). Similarly, another study reported 37 *E*. *chaffeensis* outer membrane-expressed proteins by employing an affinity purification coupled with LC/MS/MS analysis (Ge and Rikihisa, 2007). This is the first report demonstrating the presence of p28-Omps on the ECV membrane. The p28-Omp protein expression on the ECV membrane was independently confirmed by confocal microscopy. The p28-Omp expression on the ECV membrane is not a surprising outcome considering the published extensive immunological data on the p28-Omps. Several investigators reported that the p28-Omps are among the major immunogenic outer membrane proteins in *E. chaffeensis*. Similarly, p28-Omp homologs in related *Ehrlichia* species; *E. canis* and *E. ruminantium* are also highly immunogenic and contain three highly dominant immunogenic epitopes (Reddy et al., 1998; Ohashi et al., 1998; McBride et al., 1999; Li et al., 2001). These membrane proteins are also identified as having porin function to facilitate nutrient transport for the bacteria from the host cytoplasm (Kumagai et al., 2008). Considering these data, the presence of p28-Omps with porin activity also on the ECV membrane may be of exceptional importance for *E. chaffeensis* in acquiring nutrients from host cell cytoplasm.

While many *Ehrlichia* membrane proteins are also found in the ECV membrane, we did observe few important differences in the ECV membranes vs *Ehrlichia* membranes. For example, cation/H antiporter protein, predicted phage proteins and alpha/beta hydrolases were only present on the *Ehrlichia* membrane proteome (Seo et al., 2008), but not in the ECV membrane proteome. We recently reported functionally active genes encoding some of these proteins as essential for the *E. chaffeensis in vivo* growth (Cheng et al., 2013; Wang et al., 2020). The current study documenting the presence of many *E. chaffeensis* proteins modifying the ECV membrane is novel. Our data demonstrating the ECV membrane modification by the pathogen is similar to *Chlamydia* containing vacuole (CCV) membrane where large numbers of the *Chlamydia* proteins are similarly detected. While it is interesting that 21 of 22 p28-Omps were found in both *E. chaffeensis* membrane (Seo et al., 2008) as well as ECV membranes (current study), it is not clear how these proteins contribute to the bacterial replication within a phagosome. Also unclear why only one p28-Omp paralogs (p28-Omp 4/OMP-T) is not detected in either bacterial membranes (Seo et al., 2008) or in ECV membranes (current study). The presence of several Type I and Type IV secretion system proteins, ankyrin repeat proteins, the 120 kDa membrane protein, and preprotein translocases is anticipated, as these proteins are important for the bacterial effector protein translocation. Several recent studies extensively documented the importance of Type I and Type IV secretion systems in the bacterial effector translocation and their contributions in altering host responses against the pathogen (Rikihisa, 2017; Byerly et al., 2021). An efflux transporter is among the essential *E. chaffeensis* proteins for the pathogen’s continued *in vivo* growth in a vertebrate host (Wang et al., 2020). Efflux transporters are commonly found in Gram-negative bacteria which function as the active efflux of several antibiotics and chemotherapeutic agents (Nikaido and Takatsuka, 2009). Our study represents the first description of several lipoproteins being translocated on the ECV membrane. In Gram-negative bacteria, surface expressed lipoproteins play important roles in the immune evasion and nutrient acquisition from infected host cells (Cole et al., 2021). *E. chaffeensis* lacks cholesterol biosynthesis pathway and is known to acquire host-membrane components within membrane-bound vacuoles (ECVs) (Lin et al., 2020). It is likely that some of the lipoproteins on the ECV membranes may contribute to the cholesterol trafficking to the pathogen. *In toto*, evidence demonstrating the presence of many *E. chaffeensis* proteins on the ECV membrane is novel and will facilitate in furthering investigations regarding protein trafficking from the pathogen to the host cell environment and their functional significance for the bacterial evasion. The data reported in this study will aid in elucidating pathogenic evasion mechanisms and in developing effective control methods and vaccine development.

## Data availability statement

Original datasets are available in a publicly accessible repository Mass Spectrometry Interactive Virtual Environment (MassIVE) with accession number MassIVE MSV000090638. This data can be found here: https://massive.ucsd.edu/ProteoSAFe/dataset.jsp.

## Author contributions

RG conceived and directed the research design and CK designed research plan, executed and performed the experiments, and data analysis. CK and RG prepared the manuscript. All authors contributed to the article and approved the submitted version.

## Supplementary material

Supplementary Figure 1 Distribution of proteins molecular weights, unique peptides, and sequence coverage of proteins associated with *E. chaffeensis* **(A)** and macrophage host phagosome membrane **(B)**.

Supplementary Figure 2
**A**. Enrichment of membrane proteins from the host. **B**. Enrichment of membrane proteins from *E. chaffeensis*
