# Supplementary material for: Proteome analysis of Ehrlichia chaffeensis containing phagosome membranes revealed the presence of numerous bacterial and host proteins
Source: Front Cell Infect Microbiol. 2022 Dec 23;12:1070356. doi: 10.3389/fcimb.2022.1070356 (PMC9816426; doi:10.3389/fcimb.2022.1070356)
Supplement: Supplementary Figure 1 — Distribution of proteins molecular weights, unique peptides, and sequence coverage of proteins associated with E. chaffeensis (A) and macrophage host phagosome membrane (B). [file DataSheet_1.pdf]

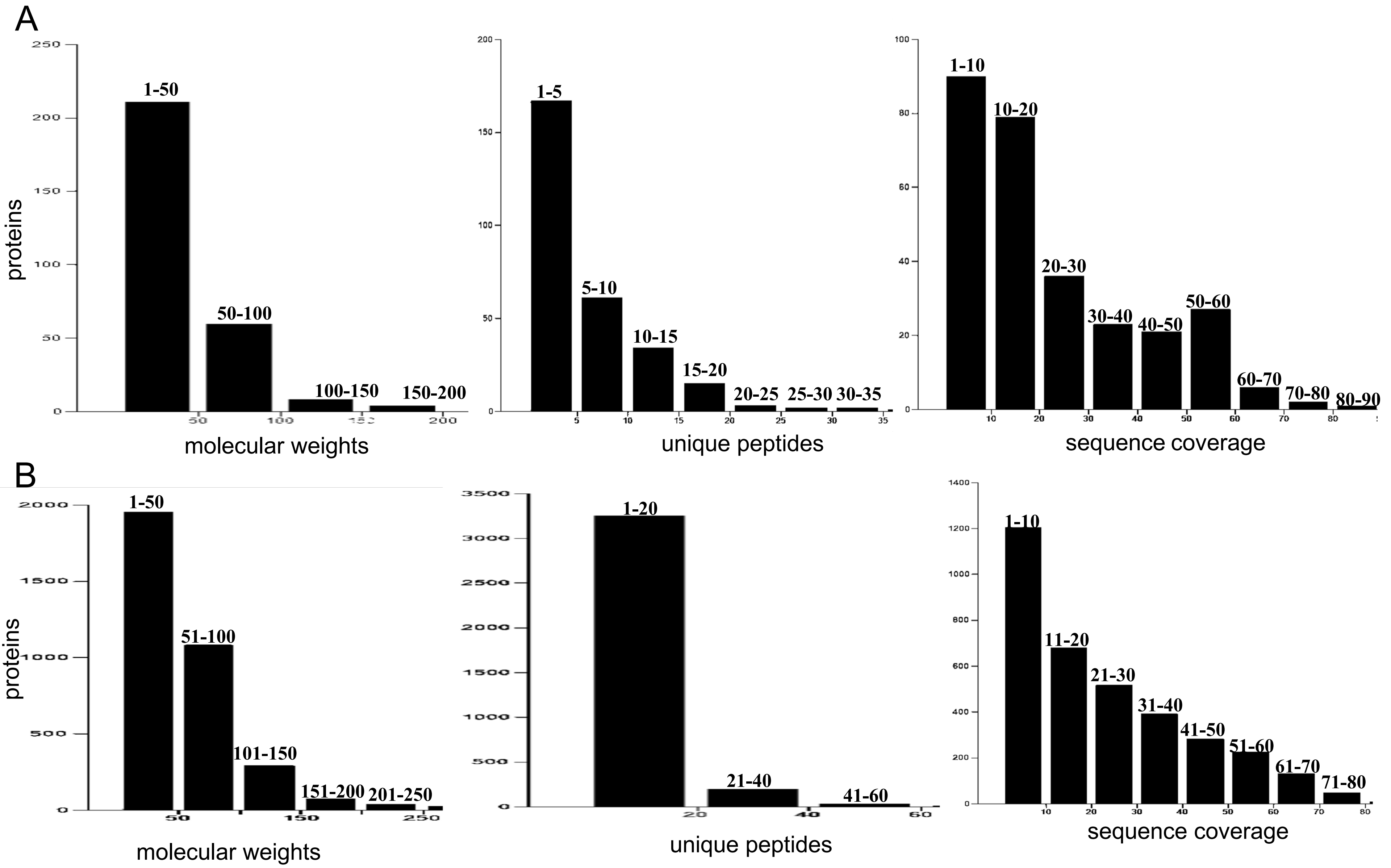

Fig. S1: Distribution of proteins molecular weights, unique peptides, and sequence coverage of proteins associated with *E. chaffeensis* (A) and macrophage host phagosome membrane (B).

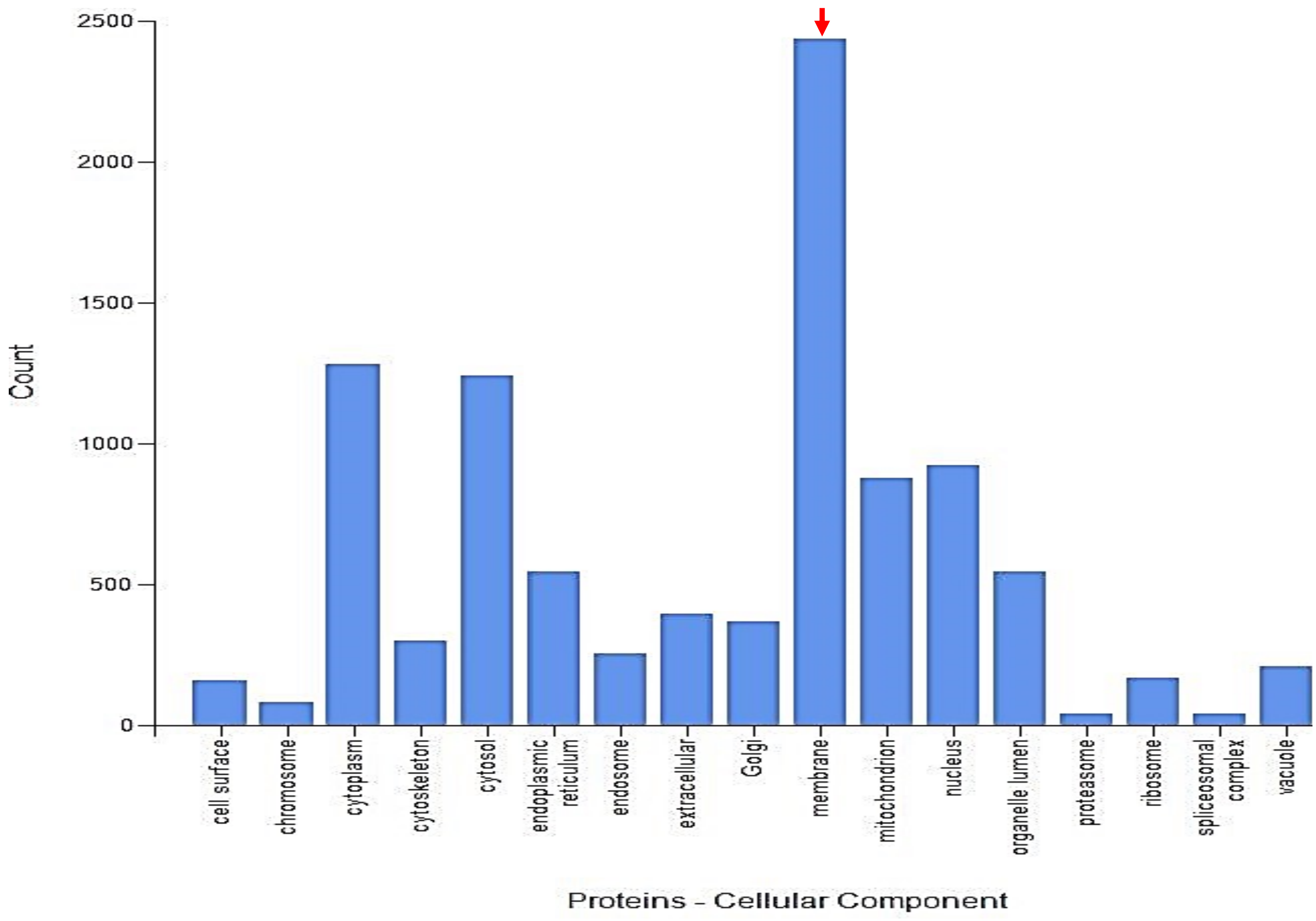

Fig. S2 A. Enrichment of membrane proteins from the host

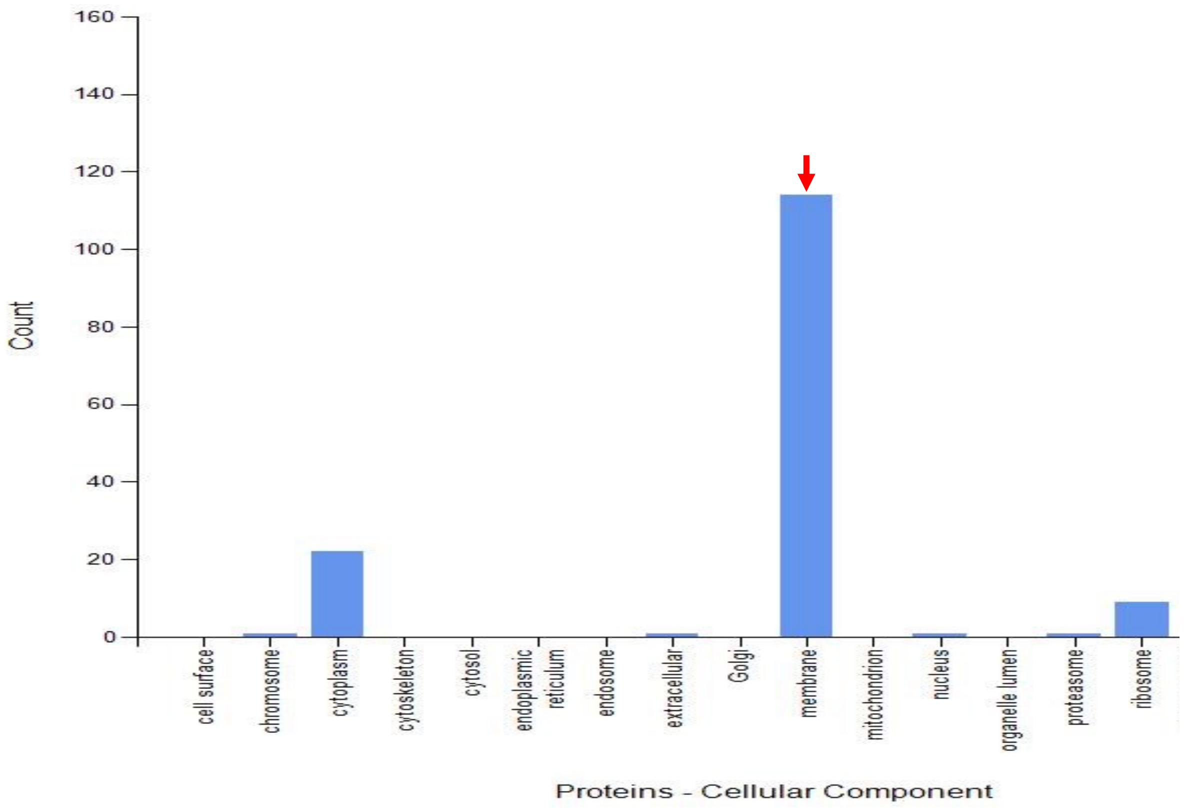

Fig.S2 B. Enrichment of membrane proteins from *E. chaffeensis*

Table S1. ***E. chaffeensis*** **proteins identified in phagosome membrane proteome (E. chaffeensis protein databases (Taxonomy ID 943, GenBank Accessions no. CP000236)**

Accession	Description	Coverage [%]	# Unique Peptides	MW [kDa]
O69197	Major outer membrane protein P28-1 OS=Ehrlichia chaffeensis OX=945 GN=p28-20 PE=4 SV=2	76	13	30
Q2GFM0	Major antigenic protein OS=Ehrlichia chaffeensis (strain ATCC CRL-10679 / Arkansas) OX=205920 GN=ECH_0976 PE=4 SV=1	71	11	23.1
H9BXB3	60 kDa chaperonin OS=Ehrlichia chaffeensis OX=945 PE=3 SV=1	68	23	41.7
Q2GHS0	Putative iron-binding protein OS=Ehrlichia chaffeensis (strain ATCC CRL-10679 / Arkansas) OX=205920 GN=ECH_0189 PE=4 SV=1	63	17	38.6
Q2GHG9	Disulfide oxidoreductase OS=Ehrlichia chaffeensis (strain ATCC CRL-10679 / Arkansas) OX=205920 GN=ECH_0293 PE=4 SV=1	61	14	27.5
Q2GGD8	Signal peptidase I OS=Ehrlichia chaffeensis (strain ATCC CRL-10679 / Arkansas) OX=205920 GN=lepB PE=3 SV=1	60	10	26.8
Q2GF78	Major outer membrane protein Omp-1N OS=Ehrlichia chaffeensis (strain ATCC CRL-10679 / Arkansas) OX=205920 GN=omp-1N PE=4 SV=1	59	12	32.1
O52105	28kDa outer membrane protein gene 16 OS=Ehrlichia chaffeensis OX=945 GN=omp-1D PE=4 SV=1	59	8	31.5
Q2GF56	Major outer membrane protein P28 OS=Ehrlichia chaffeensis (strain ATCC CRL-10679 / Arkansas) OX=205920 GN=p28 PE=4 SV=1	59	6	30.3
Q2GFR4	Putative lipoprotein OS=Ehrlichia chaffeensis (strain ATCC CRL-10679 / Arkansas) OX=205920 GN=ECH_0929 PE=4 SV=1	58	14	31.3
Q2GF76	Major outer membrane protein OMP-1Q OS=Ehrlichia chaffeensis (strain ATCC CRL-10679 / Arkansas) OX=205920 GN=omp-1Q PE=4 SV=1	57	14	33.4
Q9ACI8	Uncharacterized protein OS=Ehrlichia chaffeensis OX=945 PE=4 SV=1	56	4	14.3
Q2GHQ8	DnaJ domain protein OS=Ehrlichia chaffeensis (strain ATCC CRL-10679 / Arkansas) OX=205920 GN=ECH_0201 PE=4 SV=1	56	3	8.7
Q2GGM9	Uncharacterized protein OS=Ehrlichia chaffeensis (strain ATCC CRL-10679 / Arkansas) OX=205920 GN=ECH_0593 PE=4 SV=1	54	16	42
Q2GGA0	Peptidylprolyl isomerase OS=Ehrlichia chaffeensis (strain ATCC CRL-10679 / Arkansas) OX=205920 GN=ECH_0731 PE=4 SV=1	53	27	72.3
Q2GG97	Antioxidant, AhpC/Tsa family OS=Ehrlichia chaffeensis (strain ATCC CRL-10679 / Arkansas) OX=205920 GN=ECH_0734 PE=4 SV=1	53	7	23.3
Q2GF66	Major outer membrane protein OMP-1H OS=Ehrlichia chaffeensis (strain ATCC CRL-10679 / Arkansas) OX=205920 GN=omp-1H PE=4 SV=1	52	12	33
Q2GFC6	Outer membrane protein, OmpH family OS=Ehrlichia chaffeensis (strain ATCC CRL-10679 / Arkansas) OX=205920 GN=ECH_1072 PE=4 SV=1	52	8	21
Q2GGU3	Conserved domain protein OS=Ehrlichia chaffeensis (strain ATCC CRL-10679 / Arkansas) OX=205920 GN=ECH_0525 PE=4 SV=1	51	26	74
Q2GFH7	Putative outer membrane protein TolC OS=Ehrlichia chaffeensis (strain ATCC CRL-10679 / Arkansas) OX=205920 GN=ECH_1020 PE=4 SV=1	51	14	46.8

Q2GF59	Major outer membrane protein OMP-1E OS=Ehrlichia chaffeensis (strain ATCC CRL-10679 / Arkansas) OX=205920 GN=omp-1E PE=4 SV=1	50	6	30.5
Q2GFE6	Periplasmic serine endoprotease DegP-like OS=Ehrlichia chaffeensis (strain ATCC CRL-10679 / Arkansas) OX=205920 GN=ECH_1052 PE=3 SV=1	49	16	50.7
Q2GF73	Major outer membrane protein OMP-1U OS=Ehrlichia chaffeensis (strain ATCC CRL-10679 / Arkansas) OX=205920 GN=omp-1U PE=4 SV=1	49	11	33.6
Q2GG31	DNA-binding protein HU OS=Ehrlichia chaffeensis (strain ATCC CRL-10679 / Arkansas) OX=205920 GN=hup PE=4 SV=1	49	3	10.6
Q2GGU2	Conserved domain protein OS=Ehrlichia chaffeensis (strain ATCC CRL-10679 / Arkansas) OX=205920 GN=ECH_0526 PE=4 SV=1	48	18	55.5
Q2GF69	Major outer membrane protein OMP-1X OS=Ehrlichia chaffeensis (strain ATCC CRL-10679 / Arkansas) OX=205920 GN=omp-1X PE=4 SV=1	48	7	30.5
Q2GGZ6	Chaperone protein DnaK OS=Ehrlichia chaffeensis (strain ATCC CRL-10679 / Arkansas) OX=205920 GN=dnaK PE=2 SV=1	47	21	69
Q8GGU9	120 kDa outer membrane protein OS=Ehrlichia chaffeensis OX=945 PE=4 SV=1	47	9	52
Q2GH04	OmpA family protein OS=Ehrlichia chaffeensis (strain ATCC CRL-10679 / Arkansas) OX=205920 GN=ECH_0462 PE=3 SV=1	47	6	19.9
Q2GH86	Conserved domain protein OS=Ehrlichia chaffeensis (strain ATCC CRL-10679 / Arkansas) OX=205920 GN=ECH_0377 PE=4 SV=1	46	5	12
Q2GF62	Major outer membrane protein OMP-1C OS=Ehrlichia chaffeensis (strain ATCC CRL-10679 / Arkansas) OX=205920 GN=omp-1C PE=4 SV=1	46	5	30.3
Q8RPM3	VirB8 OS=Ehrlichia chaffeensis OX=945 GN=virB8 PE=4 SV=1	45	9	27.9
Q2GFX6	Uncharacterized protein OS=Ehrlichia chaffeensis (strain ATCC CRL-10679 / Arkansas) OX=205920 GN=ECH_0862 PE=4 SV=1	43	10	46.2
Q2GF70	Major outer membrane protein OMP-1W OS=Ehrlichia chaffeensis (strain ATCC CRL-10679 / Arkansas) OX=205920 GN=omp-1w PE=4 SV=1	43	10	31.6
Q2GGL1	Uncharacterized protein OS=Ehrlichia chaffeensis (strain ATCC CRL-10679 / Arkansas) OX=205920 GN=ECH_0612 PE=4 SV=1	42	5	23
Q2GGZ2	Signal recognition particle protein OS=Ehrlichia chaffeensis (strain ATCC CRL-10679 / Arkansas) OX=205920 GN=ffh PE=4 SV=1	41	14	49.2
Q2GF65	Major outer membrane protein OMP-1Z OS=Ehrlichia chaffeensis (strain ATCC CRL-10679 / Arkansas) OX=205920 GN=omp-1Z PE=4 SV=1	41	7	33
Q2GGC1	Uncharacterized protein OS=Ehrlichia chaffeensis (strain ATCC CRL-10679 / Arkansas) OX=205920 GN=ECH_0707 PE=4 SV=1	40	16	61.1
Q2GFD3	Dihydrolipoyllysine-residue succinyltransferase component of 2-oxoglutarate dehydrogenase complex OS=Ehrlichia chaffeensis (strain ATCC CRL-10679 / Arkansas) OX=205920 GN=sucB PE=3 SV=1	40	15	44.4
Q2GFM6	Type I secretion membrane fusion protein, HlyD family OS=Ehrlichia chaffeensis (strain ATCC CRL-10679 / Arkansas) OX=205920 GN=ECH_0970 PE=4 SV=1	40	14	56.8
Q2GH78	Quinone oxidoreductase OS=Ehrlichia chaffeensis (strain ATCC CRL-10679 / Arkansas) OX=205920 GN=qor PE=4 SV=1	40	10	35.7
Q2GFJ1	Outer membrane protein assembly factor BamD OS=Ehrlichia chaffeensis (strain ATCC CRL-10679 / Arkansas) OX=205920 GN=bamD PE=3 SV=1	40	10	29.4
O52102	Major outer membrane protein OMP-1A OS=Ehrlichia chaffeensis OX=945 GN=omp-1A PE=4 SV=2	40	9	32.9

Q9ACJ3	Major outer membrane protein OMP-1P OS=Ehrlichia chaffeensis OX=945 GN=omp-1P PE=4 SV=1	39	11	32.6
Q2GHE6	Succinate dehydrogenase iron-sulfur subunit OS=Ehrlichia chaffeensis (strain ATCC CRL-10679 / Arkansas) OX=205920 GN=ECH_0316 PE=3 SV=1	39	9	29.6
Q9L6Y5	Major outer membrane protein OMP-1Y OS=Ehrlichia chaffeensis OX=945 GN=p28-9 PE=4 SV=1	39	9	31.7
Q2GFC7	Outer membrane protein assembly factor BamA OS=Ehrlichia chaffeensis (strain ATCC CRL-10679 / Arkansas) OX=205920 GN=bamA PE=3 SV=1	38	24	87.5
Q9ACJ0	Major outer membrane protein OMP-1S OS=Ehrlichia chaffeensis OX=945 GN=omp-1S PE=4 SV=1	37	8	32
Q2GFD1	D-alanyl-D-alanine carboxypeptidase family protein OS=Ehrlichia chaffeensis (strain ATCC CRL-10679 / Arkansas) OX=205920 GN=ECH_1067 PE=3 SV=1	36	12	43.1
Q2GFE7	Protein HflC OS=Ehrlichia chaffeensis (strain ATCC CRL-10679 / Arkansas) OX=205920 GN=hflC PE=3 SV=1	36	10	33
Q9ACJ6	Major outer membrane protein OMP-1M OS=Ehrlichia chaffeensis OX=945 GN=omp-1M PE=4 SV=1	36	4	33.3
Q2GH80	Type I secretion system ATPase OS=Ehrlichia chaffeensis (strain ATCC CRL-10679 / Arkansas) OX=205920 GN=ECH_0383 PE=4 SV=1	34	14	64.4
Q2GGR3	Putative lipoprotein OS=Ehrlichia chaffeensis (strain ATCC CRL-10679 / Arkansas) OX=205920 GN=ECH_0558 PE=4 SV=1	34	9	66.2
Q2GFY4	Uncharacterized protein OS=Ehrlichia chaffeensis (strain ATCC CRL-10679 / Arkansas) OX=205920 GN=ECH_0854 PE=4 SV=1	34	6	21.4
Q2GFL6	Succinate--CoA ligase [ADP-forming] subunit alpha OS=Ehrlichia chaffeensis (strain ATCC CRL-10679 / Arkansas) OX=205920 GN=sucD PE=3 SV=1	34	6	30.5
O52107	28kDa outer membrane protein gene 18 OS=Ehrlichia chaffeensis OX=945 GN=omp-1F PE=4 SV=1	34	6	30.7
Q2GHA8	M23/M37 peptidase domain protein OS=Ehrlichia chaffeensis (strain ATCC CRL-10679 / Arkansas) OX=205920 GN=ECH_0355 PE=4 SV=1	33	5	23
Q2GHE7	Succinate dehydrogenase flavoprotein subunit OS=Ehrlichia chaffeensis (strain ATCC CRL-10679 / Arkansas) OX=205920 GN=sdhA PE=3 SV=1	32	14	65.8
Q2GI58	Type IV secretion system protein VirB9 OS=Ehrlichia chaffeensis (strain ATCC CRL-10679 / Arkansas) OX=205920 GN=virB9-1 PE=1 SV=1	32	7	31.7
Q2GI59	Type IV secretion system protein VirB10 OS=Ehrlichia chaffeensis (strain ATCC CRL-10679 / Arkansas) OX=205920 GN=virB10 PE=4 SV=1	31	9	48.5
Q2GFI9	Preprotein translocase, YajC subunit OS=Ehrlichia chaffeensis (strain ATCC CRL-10679 / Arkansas) OX=205920 GN=ECH_1008 PE=4 SV=1	31	3	13.2
Q2GHD6	Cytochrome C, membrane-bound OS=Ehrlichia chaffeensis (strain ATCC CRL-10679 / Arkansas) OX=205920 GN=cycM PE=4 SV=1	30	2	19.3
Q2GHT2	Uncharacterized protein OS=Ehrlichia chaffeensis (strain ATCC CRL-10679 / Arkansas) OX=205920 GN=ECH_0176 PE=4 SV=1	29	17	97.4
Q2GF95	Uncharacterized protein OS=Ehrlichia chaffeensis (strain ATCC CRL-10679 / Arkansas) OX=205920 GN=ECH_1104 PE=4 SV=1	29	12	59.8
Q2GFC8	Zinc metalloprotease OS=Ehrlichia chaffeensis (strain ATCC CRL-10679 / Arkansas) OX=205920 GN=ECH_1070 PE=3 SV=1	29	8	42.8
Q2GF72	Major outer membrane protein OMP-1V OS=Ehrlichia chaffeensis (strain ATCC CRL-10679 / Arkansas) OX=205920 GN=omp-1V PE=4 SV=1	29	7	30.8

O85358	28kDa outer membrane protein gene 14 OS=Ehrlichia chaffeensis OX=945 GN=omp-1B PE=4 SV=2	29	5	31
Q2GI44	2,3,4,5-tetrahydropyridine-2,6-dicarboxylate N-succinyltransferase OS=Ehrlichia chaffeensis (strain ATCC CRL-10679 / Arkansas) OX=205920 GN=dapD PE=3 SV=1	28	10	30.1
Q2GGI8	Uncharacterized protein OS=Ehrlichia chaffeensis (strain ATCC CRL-10679 / Arkansas) OX=205920 GN=ECH_0635 PE=4 SV=1	27	8	40.3
Q2GGT1	Antioxidant, AhpC/TSA family OS=Ehrlichia chaffeensis (strain ATCC CRL-10679 / Arkansas) OX=205920 GN=ECH_0539 PE=4 SV=1	27	3	16.9
Q2GFJ4	Cytochrome c oxidase subunit 2 OS=Ehrlichia chaffeensis (strain ATCC CRL-10679 / Arkansas) OX=205920 GN=ECH_1002 PE=3 SV=1	26	5	29.9
Q2GHP1	Type IV secretion system protein, VirB9 OS=Ehrlichia chaffeensis (strain ATCC CRL-10679 / Arkansas) OX=205920 GN=virB9-2 PE=4 SV=1	26	4	30.9
Q2GGU6	Ubiquinol-cytochrome c reductase, cytochrome c1 OS=Ehrlichia chaffeensis (strain ATCC CRL-10679 / Arkansas) OX=205920 GN=petC PE=4 SV=1	26	3	28.6
O85816	28kDa outer membrane protein gene 19 OS=Ehrlichia chaffeensis OX=945 PE=4 SV=1	26	2	30.3
Q2GGF8	Bifunctional protein PutA OS=Ehrlichia chaffeensis (strain ATCC CRL-10679 / Arkansas) OX=205920 GN=putA PE=3 SV=1	25	17	116.2
Q2GH94	Probable cytosol aminopeptidase OS=Ehrlichia chaffeensis (strain ATCC CRL-10679 / Arkansas) OX=205920 GN=pepA PE=3 SV=1	25	11	54.7
Q2GFE8	Protein HflK OS=Ehrlichia chaffeensis (strain ATCC CRL-10679 / Arkansas) OX=205920 GN=hflK PE=3 SV=1	25	7	40.8
Q2GHX7	Putative lipoprotein OS=Ehrlichia chaffeensis (strain ATCC CRL-10679 / Arkansas) OX=205920 GN=ECH_0128 PE=4 SV=1	25	5	30.1
Q2GGV9	Dihydrolipoyl dehydrogenase OS=Ehrlichia chaffeensis (strain ATCC CRL-10679 / Arkansas) OX=205920 GN=lpdA-1 PE=3 SV=1	24	6	49.7
Q2GGB5	Uncharacterized protein OS=Ehrlichia chaffeensis (strain ATCC CRL-10679 / Arkansas) OX=205920 GN=ECH_0715 PE=4 SV=1	23	8	61.2
Q2GHT0	Apolipoprotein N-acyltransferase OS=Ehrlichia chaffeensis (strain ATCC CRL-10679 / Arkansas) OX=205920 GN=cutE PE=3 SV=1	23	6	56.8
Q2GGJ8	Putative lipoprotein OS=Ehrlichia chaffeensis (strain ATCC CRL-10679 / Arkansas) OX=205920 GN=ECH_0625 PE=4 SV=1	23	4	15.3
Q2GGU8	Ubiquinol-cytochrome c reductase iron-sulfur subunit OS=Ehrlichia chaffeensis (strain ATCC CRL-10679 / Arkansas) OX=205920 GN=petA PE=4 SV=1	23	3	20.9
Q2GFD9	Uncharacterized protein OS=Ehrlichia chaffeensis (strain ATCC CRL-10679 / Arkansas) OX=205920 GN=ECH_1059 PE=4 SV=1	23	2	23.6
Q2GFA1	ATP-dependent zinc metalloprotease FtsH OS=Ehrlichia chaffeensis (strain ATCC CRL-10679 / Arkansas) OX=205920 GN=ftsH PE=3 SV=1	22	8	67.7
Q2GGW0	PQQ enzyme repeat family protein OS=Ehrlichia chaffeensis (strain ATCC CRL-10679 / Arkansas) OX=205920 GN=ECH_0508 PE=4 SV=1	22	5	42.8
Q2GG53	Membrane protein insertase YidC OS=Ehrlichia chaffeensis (strain ATCC CRL-10679 / Arkansas) OX=205920 GN=yidC PE=3 SV=1	21	9	66.9
Q2GI14	Glutamine synthetase, type I OS=Ehrlichia chaffeensis (strain ATCC CRL-10679 / Arkansas) OX=205920 GN=glnA PE=3 SV=1	21	7	53.4

Q2GFE1	Peptidase, M16 family OS=Ehrlichia chaffeensis (strain ATCC CRL-10679 / Arkansas) OX=205920 GN=ECH_1057 PE=4 SV=1	21	5	50
Q2GFF6	Uncharacterized protein OS=Ehrlichia chaffeensis (strain ATCC CRL-10679 / Arkansas) OX=205920 GN=ECH_1042 PE=4 SV=1	21	2	13.1
Q2GGF0	Arginine biosynthesis bifunctional protein ArgJ OS=Ehrlichia chaffeensis (strain ATCC CRL-10679 / Arkansas) OX=205920 GN=argJ PE=3 SV=1	20	7	45.5
Q2GGD3	Uncharacterized protein OS=Ehrlichia chaffeensis (strain ATCC CRL-10679 / Arkansas) OX=205920 GN=ECH_0695 PE=4 SV=1	20	5	33.4
Q2GGX5	Superoxide dismutase OS=Ehrlichia chaffeensis (strain ATCC CRL-10679 / Arkansas) OX=205920 GN=sodB PE=3 SV=1	20	2	23.5
Q2GHH0	Conserved domain protein OS=Ehrlichia chaffeensis (strain ATCC CRL-10679 / Arkansas) OX=205920 GN=ECH_0292 PE=4 SV=1	19	13	122.2
Q2GGE3	Ankyrin repeat protein OS=Ehrlichia chaffeensis (strain ATCC CRL-10679 / Arkansas) OX=205920 GN=ECH_0684 PE=4 SV=1	18	15	156.6
Q2GI88	Uncharacterized protein OS=Ehrlichia chaffeensis (strain ATCC CRL-10679 / Arkansas) OX=205920 GN=ECH_0010 PE=4 SV=1	18	8	38
Q2GGQ9	Transcription termination/antitermination protein NusA OS=Ehrlichia chaffeensis (strain ATCC CRL-10679 / Arkansas) OX=205920 GN=nusA PE=3 SV=1	18	7	57.4
Q2GFS6	Uncharacterized protein OS=Ehrlichia chaffeensis (strain ATCC CRL-10679 / Arkansas) OX=205920 GN=ECH_0916 PE=4 SV=1	18	6	46.5
Q2GGT8	Uncharacterized protein OS=Ehrlichia chaffeensis (strain ATCC CRL-10679 / Arkansas) OX=205920 GN=ECH_0531 PE=4 SV=1	18	2	19.1
Q2GHF8	Putative thiol:disulfide oxidoreductase OS=Ehrlichia chaffeensis (strain ATCC CRL-10679 / Arkansas) OX=205920 GN=ECH_0304 PE=4 SV=1	18	2	18.9
Q9ACI6	Preprotein translocase SecA subunit (Fragment) OS=Ehrlichia chaffeensis OX=945 GN=secA PE=4 SV=1	18	2	21.8
Q2GHT3	Malate dehydrogenase OS=Ehrlichia chaffeensis (strain ATCC CRL-10679 / Arkansas) OX=205920 GN=ECH_0175 PE=4 SV=1	17	10	83.1
Q8RPL9	VirD4 OS=Ehrlichia chaffeensis OX=945 GN=virD4 PE=4 SV=1	17	8	82.5
Q2GG12	Uncharacterized protein OS=Ehrlichia chaffeensis (strain ATCC CRL-10679 / Arkansas) OX=205920 GN=ECH_0825 PE=4 SV=1	17	4	42.3
Q2GHV6	Pyruvate dehydrogenase E1 component subunit beta OS=Ehrlichia chaffeensis (strain ATCC CRL-10679 / Arkansas) OX=205920 GN=ECH_0149 PE=4 SV=1	17	4	36.4
Q2GHD4	Uncharacterized protein OS=Ehrlichia chaffeensis (strain ATCC CRL-10679 / Arkansas) OX=205920 GN=ECH_0329 PE=4 SV=1	17	3	25.3
Q2GG93	Uncharacterized protein OS=Ehrlichia chaffeensis (strain ATCC CRL-10679 / Arkansas) OX=205920 GN=ECH_0739 PE=4 SV=1	17	3	30.4
Q2GH61	Putative ribosomal protein S1 OS=Ehrlichia chaffeensis (strain ATCC CRL-10679 / Arkansas) OX=205920 GN=ECH_0402 PE=4 SV=1	16	7	63.5
Q2GH62	Signal peptide peptidase SppA OS=Ehrlichia chaffeensis (strain ATCC CRL-10679 / Arkansas) OX=205920 GN=sppA PE=4 SV=1	16	6	32.3
Q2GG40	Uncharacterized protein OS=Ehrlichia chaffeensis (strain ATCC CRL-10679 / Arkansas) OX=205920 GN=ECH_0795 PE=4 SV=1	16	2	12.2

Q2GFJ9	ATP-dependent protease ATPase subunit HslU OS=Ehrlichia chaffeensis (strain ATCC CRL-10679 / Arkansas) OX=205920 GN=hslU PE=3 SV=1	15	7	54.7
Q2GFD0	C-type cytochrome family protein OS=Ehrlichia chaffeensis (strain ATCC CRL-10679 / Arkansas) OX=205920 GN=ECH_1068 PE=4 SV=1	15	5	46.2
Q2GHC8	Putative osmotically inducible protein OS=Ehrlichia chaffeensis (strain ATCC CRL-10679 / Arkansas) OX=205920 GN=ECH_0335 PE=4 SV=1	15	3	22.4
Q2GI31	1-acyl-sn-glycerol-3-phosphate acyltransferase family protein OS=Ehrlichia chaffeensis (strain ATCC CRL-10679 / Arkansas) OX=205920 GN=ECH_0072 PE=4 SV=1	15	3	26.8
Q2GI54	TPR domain protein OS=Ehrlichia chaffeensis (strain ATCC CRL-10679 / Arkansas) OX=205920 GN=ECH_0048 PE=4 SV=1	15	3	40.8
Q2GFI5	DNA-binding response regulator OS=Ehrlichia chaffeensis (strain ATCC CRL-10679 / Arkansas) OX=205920 GN=ECH_1012 PE=4 SV=1	15	3	29.9
Q2GG52	6,7-dimethyl-8-ribityllumazine synthase OS=Ehrlichia chaffeensis (strain ATCC CRL-10679 / Arkansas) OX=205920 GN=ribE-2 PE=3 SV=1	15	2	16.2
Q2GFA9	ATP synthase F0, B chain OS=Ehrlichia chaffeensis (strain ATCC CRL-10679 / Arkansas) OX=205920 GN=atpF PE=4 SV=1	15	2	18.4
Q2GHQ0	SurA domain protein OS=Ehrlichia chaffeensis (strain ATCC CRL-10679 / Arkansas) OX=205920 GN=ECH_0210 PE=4 SV=1	14	5	44.6
Q2GHU1	Tryptophan--tRNA ligase OS=Ehrlichia chaffeensis (strain ATCC CRL-10679 / Arkansas) OX=205920 GN=trpS PE=3 SV=1	14	4	37.4
Q2GHL0	Uncharacterized protein OS=Ehrlichia chaffeensis (strain ATCC CRL-10679 / Arkansas) OX=205920 GN=ECH_0252 PE=4 SV=1	14	3	40.8
Q2GGN5	Uncharacterized protein OS=Ehrlichia chaffeensis (strain ATCC CRL-10679 / Arkansas) OX=205920 GN=ECH_0587 PE=4 SV=1	14	2	17.1
Q2GHV5	Uncharacterized protein OS=Ehrlichia chaffeensis (strain ATCC CRL-10679 / Arkansas) OX=205920 GN=ECH_0150 PE=4 SV=1	13	5	75
Q2GHK1	Uncharacterized protein OS=Ehrlichia chaffeensis (strain ATCC CRL-10679 / Arkansas) OX=205920 GN=ECH_0261 PE=4 SV=1	13	2	28.5
Q2GHP0	Pyruvate dehydrogenase E1 component subunit alpha OS=Ehrlichia chaffeensis (strain ATCC CRL-10679 / Arkansas) OX=205920 GN=pdhA PE=4 SV=1	13	2	36.5
Q2GHR0	Uncharacterized protein OS=Ehrlichia chaffeensis (strain ATCC CRL-10679 / Arkansas) OX=205920 GN=ECH_0199 PE=4 SV=1	12	2	23.9
Q2GHB5	Uncharacterized protein OS=Ehrlichia chaffeensis (strain ATCC CRL-10679 / Arkansas) OX=205920 GN=ECH_0348 PE=4 SV=1	12	2	22.8
Q2GH67	Uncharacterized protein OS=Ehrlichia chaffeensis (strain ATCC CRL-10679 / Arkansas) OX=205920 GN=ECH_0396 PE=4 SV=1	12	2	26.3
Q2GGX2	Type IV secretion system protein VirB6 OS=Ehrlichia chaffeensis (strain ATCC CRL-10679 / Arkansas) OX=205920 GN=virB6 PE=4 SV=1	11	8	90.5
Q2GGV8	Efflux transporter, RND family, MFP subunit OS=Ehrlichia chaffeensis (strain ATCC CRL-10679 / Arkansas) OX=205920 GN=ECH_0510 PE=3 SV=1	11	3	40.1
Q2GH56	50S ribosomal protein L3 OS=Ehrlichia chaffeensis (strain ATCC CRL-10679 / Arkansas) OX=205920 GN=rplC PE=3 SV=1	11	2	25.1
Q2GHB6	Peptidase, M48 family OS=Ehrlichia chaffeensis (strain ATCC CRL-10679 / Arkansas) OX=205920 GN=ECH_0347 PE=3 SV=1	10	4	50.2

Q2GFE0	Peptidase, M16 family OS=Ehrlichia chaffeensis (strain ATCC CRL-10679 / Arkansas) OX=205920 GN=ECH_1058 PE=4 SV=1	10	3	50.6
Q2GHR6	Major facilitator family transporter OS=Ehrlichia chaffeensis (strain ATCC CRL-10679 / Arkansas) OX=205920 GN=ECH_0193 PE=4 SV=1	10	3	47
Q2GHV9	Putative glutathione-regulated potassium-efflux system protein KefB OS=Ehrlichia chaffeensis (strain ATCC CRL-10679 / Arkansas) OX=205920 GN=ECH_0146 PE=3 SV=1	9	4	62.2
Q2GHU7	Uncharacterized protein OS=Ehrlichia chaffeensis (strain ATCC CRL-10679 / Arkansas) OX=205920 GN=ECH_0159 PE=4 SV=1	9	3	54.3
Q9LB20	Major outer membrane protein P28-2 OS=Ehrlichia chaffeensis OX=945 GN=p28-21 PE=4 SV=1	9	2	31.8
Q2GFG1	Uncharacterized protein OS=Ehrlichia chaffeensis (strain ATCC CRL-10679 / Arkansas) OX=205920 GN=ECH_1037 PE=4 SV=1	8	8	140.5
Q2GGK5	NADH-quinone oxidoreductase OS=Ehrlichia chaffeensis (strain ATCC CRL-10679 / Arkansas) OX=205920 GN=nuoG PE=3 SV=1	8	4	75.9
Q2GHF6	TRAP transporter, 4TM/12TM fusion protein OS=Ehrlichia chaffeensis (strain ATCC CRL-10679 / Arkansas) OX=205920 GN=ECH_0306 PE=4 SV=1	7	7	97.6
Q2GGX3	Type IV secretion system protein VirB4 OS=Ehrlichia chaffeensis (strain ATCC CRL-10679 / Arkansas) OX=205920 GN=virB4-1 PE=4 SV=1	7	3	90.7
Q2GH11	Uncharacterized protein OS=Ehrlichia chaffeensis (strain ATCC CRL-10679 / Arkansas) OX=205920 GN=ECH_0454 PE=4 SV=1	7	2	34.5
Q2GH37	Protein translocase subunit SecY OS=Ehrlichia chaffeensis (strain ATCC CRL-10679 / Arkansas) OX=205920 GN=secY PE=3 SV=1	7	2	48.4
Q2GFJ3	Cytochrome c oxidase subunit 1 OS=Ehrlichia chaffeensis (strain ATCC CRL-10679 / Arkansas) OX=205920 GN=ctaD PE=3 SV=1	7	2	56.9
Q2GFB0	ATP synthase F0, B' subunit OS=Ehrlichia chaffeensis (strain ATCC CRL-10679 / Arkansas) OX=205920 GN=atpX PE=3 SV=1	7	2	19.6
Q2GFU8	Putative cell division protein FtsK OS=Ehrlichia chaffeensis (strain ATCC CRL-10679 / Arkansas) OX=205920 GN=ECH_0890 PE=3 SV=1	6	3	93.4
Q2GGY2	Diaminopimelate decarboxylase OS=Ehrlichia chaffeensis (strain ATCC CRL-10679 / Arkansas) OX=205920 GN=lysA PE=3 SV=1	6	2	46.9
Q2GHE9	ABC transporter, permease/ATP-binding protein OS=Ehrlichia chaffeensis (strain ATCC CRL-10679 / Arkansas) OX=205920 GN=ECH_0313 PE=4 SV=1	6	2	64.8
Q2GGX0	Type IV secretion system protein,VirB6 family OS=Ehrlichia chaffeensis (strain ATCC CRL-10679 / Arkansas) OX=205920 GN=ECH_0498 PE=4 SV=1	5	5	159
Q2GGE5	Uncharacterized protein OS=Ehrlichia chaffeensis (strain ATCC CRL-10679 / Arkansas) OX=205920 GN=ECH_0681 PE=4 SV=1	5	2	24.1
Q2GF85	Dehydrogenase, isocitrate/isopropylmalate family OS=Ehrlichia chaffeensis (strain ATCC CRL-10679 / Arkansas) OX=205920 GN=ECH_1114 PE=4 SV=1	5	2	54.1
Q2GFV3	Sensor histidine kinase OS=Ehrlichia chaffeensis (strain ATCC CRL-10679 / Arkansas) OX=205920 GN=ECH_0885 PE=4 SV=1	5	2	53.3
Q2GF93	Protein translocase subunit SecD OS=Ehrlichia chaffeensis (strain ATCC CRL-10679 / Arkansas) OX=205920 GN=secD PE=3 SV=1	5	2	55.8
A6MFP6	ECH0849-like protein OS=Ehrlichia chaffeensis OX=945 PE=4 SV=1	4	4	121.2

Q2GFU0	Lon protease OS=Ehrlichia chaffeensis (strain ATCC CRL-10679 / Arkansas) OX=205920 GN=lon PE=2 SV=1	4	3	90.2
Q2GGU7	Cytochrome b OS=Ehrlichia chaffeensis (strain ATCC CRL-10679 / Arkansas) OX=205920 GN=petB PE=3 SV=1	4	2	47
Q2GGX1	Type IV secretion system protein, VirB6 family OS=Ehrlichia chaffeensis (strain ATCC CRL-10679 / Arkansas) OX=205920 GN=ECH_0497 PE=1 SV=1	4	2	103.9
Q2GG05	2-oxoglutarate dehydrogenase, E1 component OS=Ehrlichia chaffeensis (strain ATCC CRL-10679 / Arkansas) OX=205920 GN=sucA PE=4 SV=1	3	2	103.2
Q2GFF7	Type IV secretion system protein VirB4 OS=Ehrlichia chaffeensis (strain ATCC CRL-10679 / Arkansas) OX=205920 GN=virB4-2 PE=4 SV=1	3	2	90.4
Q2GGH1	Ankyrin repeat protein OS=Ehrlichia chaffeensis (strain ATCC CRL-10679 / Arkansas) OX=205920 GN=ECH_0653 PE=4 SV=1	1	3	476.5

Table S2. Host phagosome membrane proteins identified in the phagosome membrane proteome (uniprot human database; GenBank Accessions no. UP000005604)

Accession	Description	Coverage [%]	# Unique Peptides	MW [kDa]
P05387	60S acidic ribosomal protein P2 OS=Homo sapiens GN=RPLP2 PE=1 SV=1	92	6	11.7
P04179	Superoxide dismutase [Mn], mitochondrial OS=Homo sapiens GN=SOD2 PE=1 SV=2	91	17	24.7
P35232	Prohibitin OS=Homo sapiens GN=PHB PE=1 SV=1	90	26	29.8
O43504	Ragulator complex protein LAMTOR5 OS=Homo sapiens GN=LAMTOR5 PE=1 SV=1	86	4	9.6
P07203	Glutathione peroxidase 1 OS=Homo sapiens GN=GPX1 PE=1 SV=4	85	13	22.1
P51149	Ras-related protein Rab-7a OS=Homo sapiens GN=RAB7A PE=1 SV=1	83	20	23.5
P80723	Brain acid soluble protein 1 OS=Homo sapiens GN=BASP1 PE=1 SV=2	83	16	22.7
P60709	Actin, cytoplasmic 1 OS=Homo sapiens GN=ACTB PE=1 SV=1	83	10	41.7
P07437	Tubulin beta chain OS=Homo sapiens GN=TUBB PE=1 SV=2	83	7	49.6
P68371	Tubulin beta-4B chain OS=Homo sapiens GN=TUBB4B PE=1 SV=1	82	3	49.8
O00299	Chloride intracellular channel protein 1 OS=Homo sapiens GN=CLIC1 PE=1 SV=4	80	16	26.9
P62834	Ras-related protein Rap-1A OS=Homo sapiens GN=RAP1A PE=1 SV=1	80	5	21
P61224	Ras-related protein Rap-1b OS=Homo sapiens GN=RAP1B PE=1 SV=1	80	5	20.8
P04040	Catalase OS=Homo sapiens GN=CAT PE=1 SV=3	79	39	59.7
P61106	Ras-related protein Rab-14 OS=Homo sapiens GN=RAB14 PE=1 SV=4	79	14	23.9
Q9Y6N5	Sulfide:quinone oxidoreductase, mitochondrial OS=Homo sapiens GN=SQRDL PE=1 SV=1	78	35	49.9
P33241	Lymphocyte-specific protein 1 OS=Homo sapiens GN=LSP1 PE=1 SV=1	78	18	37.2
O00161	Synaptosomal-associated protein 23 OS=Homo sapiens GN=SNAP23 PE=1 SV=1	78	15	23.3
P27797	Calreticulin OS=Homo sapiens GN=CALR PE=1 SV=1	77	28	48.1
P00387	NADH-cytochrome b5 reductase 3 OS=Homo sapiens GN=CYB5R3 PE=1 SV=3	77	19	34.2
O95182	NADH dehydrogenase [ubiquinone] 1 alpha subcomplex subunit 7 OS=Homo sapiens GN=NDUFA7 PE=1 SV=3	77	10	12.5
P61225	Ras-related protein Rap-2b OS=Homo sapiens GN=RAP2B PE=1 SV=1	77	8	20.5
P13796	Plastin-2 OS=Homo sapiens GN=LCP1 PE=1 SV=6	76	43	70.2
Q99714	3-hydroxyacyl-CoA dehydrogenase type-2 OS=Homo sapiens GN=HSD17B10 PE=1 SV=3	76	14	26.9

Q99623	Prohibitin-2 OS=Homo sapiens GN=PHB2 PE=1 SV=2	75	21	33.3
P21796	Voltage-dependent anion-selective channel protein 1 OS=Homo sapiens GN=VDAC1 PE=1 SV=2	75	17	30.8
P37802	Transgelin-2 OS=Homo sapiens GN=TAGLN2 PE=1 SV=3	75	13	22.4
Q6IAA8	Ragulator complex protein LAMTOR1 OS=Homo sapiens GN=LAMTOR1 PE=1 SV=2	75	8	17.7
Q9BQE3	Tubulin alpha-1C chain OS=Homo sapiens GN=TUBA1C PE=1 SV=1	75	2	49.9
P04350	Tubulin beta-4A chain OS=Homo sapiens GN=TUBB4A PE=1 SV=2	75	2	49.6
P07237	Protein disulfide-isomerase OS=Homo sapiens GN=P4HB PE=1 SV=3	74	46	57.1
O15144	Actin-related protein 2/3 complex subunit 2 OS=Homo sapiens GN=ARPC2 PE=1 SV=1	74	23	34.3
P52907	F-actin-capping protein subunit alpha-1 OS=Homo sapiens GN=CAPZA1 PE=1 SV=3	74	10	32.9
O75955	Flotillin-1 OS=Homo sapiens GN=FLOT1 PE=1 SV=3	73	26	47.3
O75947	ATP synthase subunit d, mitochondrial OS=Homo sapiens GN=ATP5H PE=1 SV=3	73	13	18.5
Q9UI09	NADH dehydrogenase [ubiquinone] 1 alpha subcomplex subunit 12 OS=Homo sapiens GN=NDUFA12 PE=1 SV=1	73	10	17.1
G8JLA2	Myosin light polypeptide 6 OS=Homo sapiens GN=MYL6 PE=1 SV=1	73	9	17.1
Q9Y3L5	Ras-related protein Rap-2c OS=Homo sapiens GN=RAP2C PE=1 SV=1	73	4	20.7
P10809	60 kDa heat shock protein, mitochondrial OS=Homo sapiens GN=HSPD1 PE=1 SV=2	72	45	61
P06576	ATP synthase subunit beta, mitochondrial OS=Homo sapiens GN=ATP5B PE=1 SV=3	72	31	56.5
P55084	Trifunctional enzyme subunit beta, mitochondrial OS=Homo sapiens GN=HADHB PE=1 SV=3	72	30	51.3
P49411	Elongation factor Tu, mitochondrial OS=Homo sapiens GN=TUFM PE=1 SV=2	72	29	49.5
P04406	Glyceraldehyde-3-phosphate dehydrogenase OS=Homo sapiens GN=GAPDH PE=1 SV=3	72	17	36
P04899	Guanine nucleotide-binding protein G(i) subunit alpha-2 OS=Homo sapiens GN=GNAI2 PE=1 SV=3	72	15	40.4
Q9GZT3	SRA stem-loop-interacting RNA-binding protein, mitochondrial OS=Homo sapiens GN=SLIRP PE=1 SV=1	72	10	12.3
P09382	Galectin-1 OS=Homo sapiens GN=LGALS1 PE=1 SV=2	72	7	14.7
O75438	NADH dehydrogenase [ubiquinone] 1 beta subcomplex subunit 1 OS=Homo sapiens GN=NDUFB1 PE=1 SV=1	72	5	7
P04843	Dolichyl-diphosphooligosaccharide--protein glycosyltransferase subunit 1 OS=Homo sapiens GN=RPN1 PE=1 SV=1	71	45	68.5
P43304	Glycerol-3-phosphate dehydrogenase, mitochondrial OS=Homo sapiens GN=GPD2 PE=1 SV=3	71	43	80.8
P26038	Moesin OS=Homo sapiens GN=MSN PE=1 SV=3	71	37	67.8
P30101	Protein disulfide-isomerase A3 OS=Homo sapiens GN=PDIA3 PE=1 SV=4	71	31	56.7

P21281	V-type proton ATPase subunit B, brain isoform OS=Homo sapiens GN=ATP6V1B2 PE=1 SV=3	71	27	56.5
Q9P0L0	Vesicle-associated membrane protein-associated protein A OS=Homo sapiens GN=VAPA PE=1 SV=3	71	17	27.9
P56385	ATP synthase subunit e, mitochondrial OS=Homo sapiens GN=ATP5I PE=1 SV=2	71	6	7.9
P34897	Serine hydroxymethyltransferase, mitochondrial OS=Homo sapiens GN=SHMT2 PE=1 SV=3	70	30	56
P04075	Fructose-bisphosphate aldolase A OS=Homo sapiens GN=ALDOA PE=1 SV=2	70	18	39.4
Q06830	Peroxiredoxin-1 OS=Homo sapiens GN=PRDX1 PE=1 SV=1	70	14	22.1
P84095	Rho-related GTP-binding protein RhoG OS=Homo sapiens GN=RHOG PE=1 SV=1	70	11	21.3
O95571	Persulfide dioxygenase ETHE1, mitochondrial OS=Homo sapiens GN=ETHE1 PE=1 SV=2	70	10	27.9
P06702	Protein S100-A9 OS=Homo sapiens GN=S100A9 PE=1 SV=1	70	6	13.2
P04080	Cystatin-B OS=Homo sapiens GN=CSTB PE=1 SV=2	70	4	11.1
O43707	Alpha-actinin-4 OS=Homo sapiens GN=ACTN4 PE=1 SV=2	69	41	104.8
P40939	Trifunctional enzyme subunit alpha, mitochondrial OS=Homo sapiens GN=HADHA PE=1 SV=2	69	41	82.9
P35908	Keratin, type II cytoskeletal 2 epidermal OS=Homo sapiens GN=KRT2 PE=1 SV=2	69	28	65.4
P06733	Alpha-enolase OS=Homo sapiens GN=ENO1 PE=1 SV=2	69	25	47.1
Q96HS1	Serine/threonine-protein phosphatase PGAM5, mitochondrial OS=Homo sapiens GN=PGAM5 PE=1 SV=2	69	17	32
A0A1P0AYU5	Sideroflexin OS=Homo sapiens GN=SFXN3 PE=1 SV=1	69	17	36
P54819	Adenylate kinase 2, mitochondrial OS=Homo sapiens GN=AK2 PE=1 SV=2	69	15	26.5
P60174	Triosephosphate isomerase OS=Homo sapiens GN=TPI1 PE=1 SV=3	69	15	30.8
Q9Y2Q3	Glutathione S-transferase kappa 1 OS=Homo sapiens GN=GSTK1 PE=1 SV=3	69	12	25.5
Q14697	Neutral alpha-glucosidase AB OS=Homo sapiens GN=GANAB PE=1 SV=3	69	11	106.8
P82909	28S ribosomal protein S36, mitochondrial OS=Homo sapiens GN=MRPS36 PE=1 SV=2	69	5	11.5
P62136	Serine/threonine-protein phosphatase PP1-alpha catalytic subunit OS=Homo sapiens GN=PPP1CA PE=1 SV=1	69	5	37.5
E9PKU7	Neutral alpha-glucosidase AB OS=Homo sapiens GN=GANAB PE=1 SV=1	69	3	96.5
P05164	Myeloperoxidase OS=Homo sapiens GN=MPO PE=1 SV=1	68	56	83.8
A8K968	Band 4.1-like protein 3 OS=Homo sapiens GN=EPB41L3 PE=1 SV=1	68	5	84.7
P35579	Myosin-9 OS=Homo sapiens GN=MYH9 PE=1 SV=4	67	165	226.4
P38606	V-type proton ATPase catalytic subunit A OS=Homo sapiens GN=ATP6V1A PE=1 SV=2	67	32	68.3
P23368	NAD-dependent malic enzyme, mitochondrial OS=Homo sapiens GN=ME2 PE=1 SV=1	67	30	65.4

P31930	Cytochrome b-c1 complex subunit 1, mitochondrial OS=Homo sapiens GN=UQCRC1 PE=1 SV=3	67	27	52.6
Q16718	NADH dehydrogenase [ubiquinone] 1 alpha subcomplex subunit 5 OS=Homo sapiens GN=NDUFA5 PE=1 SV=3	67	8	13.5
Q9HCN8	Stromal cell-derived factor 2-like protein 1 OS=Homo sapiens GN=SDF2L1 PE=1 SV=2	67	8	23.6
O75608	Acyl-protein thioesterase 1 OS=Homo sapiens GN=LYPLA1 PE=1 SV=1	67	7	24.7
P68366	Tubulin alpha-4A chain OS=Homo sapiens GN=TUBA4A PE=1 SV=1	67	7	49.9
P14854	Cytochrome c oxidase subunit 6B1 OS=Homo sapiens GN=COX6B1 PE=1 SV=2	67	6	10.2
P07919	Cytochrome b-c1 complex subunit 6, mitochondrial OS=Homo sapiens GN=UQCRH PE=1 SV=2	67	5	10.7
P05386	60S acidic ribosomal protein P1 OS=Homo sapiens GN=RPLP1 PE=1 SV=1	67	2	11.5
B0I1T2	Unconventional myosin-Ig OS=Homo sapiens GN=MYO1G PE=1 SV=2	66	59	116.4
O00116	Alkyldihydroxyacetonephosphate synthase, peroxisomal OS=Homo sapiens GN=AGPS PE=1 SV=1	66	37	72.9
P28331	NADH-ubiquinone oxidoreductase 75 kDa subunit, mitochondrial OS=Homo sapiens GN=NDUFS1 PE=1 SV=3	66	35	79.4
Q53H12	Acylglycerol kinase, mitochondrial OS=Homo sapiens GN=AGK PE=1 SV=2	66	23	47.1
P07355	Annexin A2 OS=Homo sapiens GN=ANXA2 PE=1 SV=2	66	22	38.6
P07954	Fumarate hydratase, mitochondrial OS=Homo sapiens GN=FH PE=1 SV=3	66	19	54.6
Q15738	Sterol-4-alpha-carboxylate 3-dehydrogenase, decarboxylating OS=Homo sapiens GN=NSDHL PE=1 SV=2	66	17	41.9
P30040	Endoplasmic reticulum resident protein 29 OS=Homo sapiens GN=ERP29 PE=1 SV=4	66	16	29
Q9BPW8	Protein NipSnap homolog 1 OS=Homo sapiens GN=NIPSNAP1 PE=1 SV=1	66	15	33.3
Q8N5N7	39S ribosomal protein L50, mitochondrial OS=Homo sapiens GN=MRPL50 PE=1 SV=2	66	8	18.3
P20339	Ras-related protein Rab-5A OS=Homo sapiens GN=RAB5A PE=1 SV=2	66	8	23.6
Q16891	MICOS complex subunit MIC60 OS=Homo sapiens GN=IMMT PE=1 SV=1	66	6	83.6
Q9BZE1	39S ribosomal protein L37, mitochondrial OS=Homo sapiens GN=MRPL37 PE=1 SV=2	65	23	48.1
Q02127	Dihydroorotate dehydrogenase (quinone), mitochondrial OS=Homo sapiens GN=DHODH PE=1 SV=3	65	21	42.8
P42765	3-ketoacyl-CoA thiolase, mitochondrial OS=Homo sapiens GN=ACAA2 PE=1 SV=2	65	17	41.9
P23396	40S ribosomal protein S3 OS=Homo sapiens GN=RPS3 PE=1 SV=2	65	14	26.7
O96008	Mitochondrial import receptor subunit TOM40 homolog OS=Homo sapiens GN=TOMM40 PE=1 SV=1	65	14	37.9
Q9Y3B7	39S ribosomal protein L11, mitochondrial OS=Homo sapiens GN=MRPL11 PE=1 SV=1	65	13	20.7
P08754	Guanine nucleotide-binding protein G(k) subunit alpha OS=Homo sapiens GN=GNAI3 PE=1 SV=3	65	12	40.5
P82663	28S ribosomal protein S25, mitochondrial OS=Homo sapiens GN=MRPS25 PE=1 SV=1	65	11	20.1

Q9Y2B0	Protein canopy homolog 2 OS=Homo sapiens GN=CNPY2 PE=1 SV=1	65	10	20.6
P62820	Ras-related protein Rab-1A OS=Homo sapiens GN=RAB1A PE=1 SV=3	65	6	22.7
Q96EL3	39S ribosomal protein L53, mitochondrial OS=Homo sapiens GN=MRPL53 PE=1 SV=1	65	5	12.1
Q9Y5J7	Mitochondrial import inner membrane translocase subunit Tim9 OS=Homo sapiens GN=TIMM9 PE=1 SV=1	65	5	10.4
C9J406	MICOS complex subunit MIC60 OS=Homo sapiens GN=IMMT PE=1 SV=1	65	3	73.2
P47985	Cytochrome b-c1 complex subunit Rieske, mitochondrial OS=Homo sapiens GN=UQCRFS1 PE=1 SV=2	65	2	29.6
Q15084	Protein disulfide-isomerase A6 OS=Homo sapiens GN=PDIA6 PE=1 SV=1	64	24	48.1
P49821	NADH dehydrogenase [ubiquinone] flavoprotein 1, mitochondrial OS=Homo sapiens GN=NDUFV1 PE=1 SV=4	64	23	50.8
P08559	Pyruvate dehydrogenase E1 component subunit alpha, somatic form, mitochondrial OS=Homo sapiens GN=PDHA1 PE=1 SV=3	64	22	43.3
P40926	Malate dehydrogenase, mitochondrial OS=Homo sapiens GN=MDH2 PE=1 SV=3	64	17	35.5
O94905	Erlin-2 OS=Homo sapiens GN=ERLIN2 PE=1 SV=1	64	15	37.8
P54920	Alpha-soluble NSF attachment protein OS=Homo sapiens GN=NAPA PE=1 SV=3	64	13	33.2
Q9BYD3	39S ribosomal protein L4, mitochondrial OS=Homo sapiens GN=MRPL4 PE=1 SV=1	64	12	34.9
P48047	ATP synthase subunit O, mitochondrial OS=Homo sapiens GN=ATP5O PE=1 SV=1	64	11	23.3
O00264	Membrane-associated progesterone receptor component 1 OS=Homo sapiens GN=PGRMC1 PE=1 SV=3	64	11	21.7
P27144	Adenylate kinase 4, mitochondrial OS=Homo sapiens GN=AK4 PE=1 SV=1	64	10	25.3
O14548	Cytochrome c oxidase subunit 7A-related protein, mitochondrial OS=Homo sapiens GN=COX7A2L PE=1 SV=2	64	7	12.6
P62263	40S ribosomal protein S14 OS=Homo sapiens GN=RPS14 PE=1 SV=3	64	5	16.3
Q9H0U4	Ras-related protein Rab-1B OS=Homo sapiens GN=RAB1B PE=1 SV=1	64	5	22.2
Q32Q12	Nucleoside diphosphate kinase OS=Homo sapiens GN=NME1-NME2 PE=1 SV=1	64	4	32.6
P13667	Protein disulfide-isomerase A4 OS=Homo sapiens GN=PDIA4 PE=1 SV=2	63	43	72.9
Q99798	Aconitate hydratase, mitochondrial OS=Homo sapiens GN=ACO2 PE=1 SV=2	63	40	85.4
P14618	Pyruvate kinase PKM OS=Homo sapiens GN=PKM PE=1 SV=4	63	29	57.9
P07948	Tyrosine-protein kinase Lyn OS=Homo sapiens GN=LYN PE=1 SV=3	63	29	58.5
P00367	Glutamate dehydrogenase 1, mitochondrial OS=Homo sapiens GN=GLUD1 PE=1 SV=2	63	28	61.4
P61158	Actin-related protein 3 OS=Homo sapiens GN=ACTR3 PE=1 SV=3	63	21	47.3
P08133	Annexin A6 OS=Homo sapiens GN=ANXA6 PE=1 SV=3	63	19	75.8
P08571	Monocyte differentiation antigen CD14 OS=Homo sapiens GN=CD14 PE=1 SV=2	63	18	40.1

P38117	Electron transfer flavoprotein subunit beta OS=Homo sapiens GN=ETFB PE=1 SV=3	63	17	27.8
P21964	Catechol O-methyltransferase OS=Homo sapiens GN=COMT PE=1 SV=2	63	16	30
Q9UHQ9	NADH-cytochrome b5 reductase 1 OS=Homo sapiens GN=CYB5R1 PE=1 SV=1	63	16	34.1
P14927	Cytochrome b-c1 complex subunit 7 OS=Homo sapiens GN=UQCRB PE=1 SV=2	63	10	13.5
Q9P0J0	NADH dehydrogenase [ubiquinone] 1 alpha subcomplex subunit 13 OS=Homo sapiens GN=NDUFA13 PE=1 SV=3	63	10	16.7
Q99497	Protein DJ-1 OS=Homo sapiens GN=PARK7 PE=1 SV=2	63	9	19.9
Q16540	39S ribosomal protein L23, mitochondrial OS=Homo sapiens GN=MRPL23 PE=1 SV=1	63	7	17.8
Q8IXM3	39S ribosomal protein L41, mitochondrial OS=Homo sapiens GN=MRPL41 PE=1 SV=1	63	6	15.4
Q9C002	Normal mucosa of esophagus-specific gene 1 protein OS=Homo sapiens GN=NMES1 PE=2 SV=1	63	6	9.6
P63218	Guanine nucleotide-binding protein G(I)/G(S)/G(O) subunit gamma-5 OS=Homo sapiens GN=GNG5 PE=1 SV=3	63	3	7.3
P21333	Filamin-A OS=Homo sapiens GN=FLNA PE=1 SV=4	62	111	280.6
P46940	Ras GTPase-activating-like protein IQGAP1 OS=Homo sapiens GN=IQGAP1 PE=1 SV=1	62	85	189.1
P51659	Peroxisomal multifunctional enzyme type 2 OS=Homo sapiens GN=HSD17B4 PE=1 SV=3	62	34	79.6
P35527	Keratin, type I cytoskeletal 9 OS=Homo sapiens GN=KRT9 PE=1 SV=3	62	27	62
Q96HE7	ERO1-like protein alpha OS=Homo sapiens GN=ERO1A PE=1 SV=2	62	22	54.4
E9PK25	Cofilin-1 OS=Homo sapiens GN=CFL1 PE=1 SV=1	62	20	22.7
P00558	Phosphoglycerate kinase 1 OS=Homo sapiens GN=PGK1 PE=1 SV=3	62	20	44.6
O43852	Calumenin OS=Homo sapiens GN=CALU PE=1 SV=2	62	14	37.1
O75558	Syntaxin-11 OS=Homo sapiens GN=STX11 PE=1 SV=1	62	14	33.2
A0A087WYF7	MICOS complex subunit OS=Homo sapiens GN=APOOL PE=1 SV=1	62	11	29.2
P18669	Phosphoglycerate mutase 1 OS=Homo sapiens GN=PGAM1 PE=1 SV=2	62	11	28.8
P61586	Transforming protein RhoA OS=Homo sapiens GN=RHOA PE=1 SV=1	62	10	21.8
Q14019	Coactosin-like protein OS=Homo sapiens GN=COTL1 PE=1 SV=3	62	8	15.9
Q13636	Ras-related protein Rab-31 OS=Homo sapiens GN=RAB31 PE=1 SV=1	62	8	21.6
Q9UHA4	Ragulator complex protein LAMTOR3 OS=Homo sapiens GN=LAMTOR3 PE=1 SV=1	62	5	13.6
Q9UBX3	Mitochondrial dicarboxylate carrier OS=Homo sapiens GN=SLC25A10 PE=1 SV=2	62	2	31.3
P12814	Alpha-actinin-1 OS=Homo sapiens GN=ACTN1 PE=1 SV=2	61	25	103
P78324	Tyrosine-protein phosphatase non-receptor type substrate 1 OS=Homo sapiens GN=SIRPA PE=1 SV=2	61	18	54.9

P11177	Pyruvate dehydrogenase E1 component subunit beta, mitochondrial OS=Homo sapiens GN=PDHB PE=1 SV=3	61	15	39.2
P13804	Electron transfer flavoprotein subunit alpha, mitochondrial OS=Homo sapiens GN=ETFA PE=1 SV=1	61	14	35.1
Q15907	Ras-related protein Rab-11B OS=Homo sapiens GN=RAB11B PE=1 SV=4	61	13	24.5
J3QRS3	Myosin regulatory light chain 12A OS=Homo sapiens GN=MYL12A PE=1 SV=1	61	12	20.4
Q13162	Peroxiredoxin-4 OS=Homo sapiens GN=PRDX4 PE=1 SV=1	61	12	30.5
P17568	NADH dehydrogenase [ubiquinone] 1 beta subcomplex subunit 7 OS=Homo sapiens GN=NDUFB7 PE=1 SV=4	61	10	16.4
P02794	Ferritin heavy chain OS=Homo sapiens GN=FTH1 PE=1 SV=2	61	9	21.2
P07737	Profilin-1 OS=Homo sapiens GN=PFN1 PE=1 SV=2	61	8	15
P51148	Ras-related protein Rab-5C OS=Homo sapiens GN=RAB5C PE=1 SV=2	61	8	23.5
A0A0G2JIF2	HLA class I histocompatibility antigen, A-3 alpha chain OS=Homo sapiens GN=HLA-A PE=1 SV=1	61	7	41.3
P62330	ADP-ribosylation factor 6 OS=Homo sapiens GN=ARF6 PE=1 SV=2	61	6	20.1
P61019	Ras-related protein Rab-2A OS=Homo sapiens GN=RAB2A PE=1 SV=1	61	6	23.5
Q5JPE7	Nodal modulator 2 OS=Homo sapiens GN=NOMO2 PE=1 SV=1	61	5	139.4
Q09666	Neuroblast differentiation-associated protein AHNAK OS=Homo sapiens GN=AHNAK PE=1 SV=2	60	173	628.7
P38646	Stress-70 protein, mitochondrial OS=Homo sapiens GN=HSPA9 PE=1 SV=2	60	45	73.6
P55072	Transitional endoplasmic reticulum ATPase OS=Homo sapiens GN=VCP PE=1 SV=4	60	39	89.3
O75489	NADH dehydrogenase [ubiquinone] iron-sulfur protein 3, mitochondrial OS=Homo sapiens GN=NDUFS3 PE=1 SV=1	60	21	30.2
Q9BRR6	ADP-dependent glucokinase OS=Homo sapiens GN=ADPGK PE=1 SV=1	60	19	54.1
Q14344	Guanine nucleotide-binding protein subunit alpha-13 OS=Homo sapiens GN=GNA13 PE=1 SV=2	60	18	44
Q12907	Vesicular integral-membrane protein VIP36 OS=Homo sapiens GN=LMAN2 PE=1 SV=1	60	17	40.2
Q9H9B4	Sideroflexin-1 OS=Homo sapiens GN=SFXN1 PE=1 SV=4	60	15	35.6
Q99828	Calcium and integrin-binding protein 1 OS=Homo sapiens GN=CIB1 PE=1 SV=4	60	11	21.7
Q9GZY6	Linker for activation of T-cells family member 2 OS=Homo sapiens GN=LAT2 PE=1 SV=1	60	10	26.5
Q9BYD1	39S ribosomal protein L13, mitochondrial OS=Homo sapiens GN=MRPL13 PE=1 SV=1	60	9	20.7
P61604	10 kDa heat shock protein, mitochondrial OS=Homo sapiens GN=HSPE1 PE=1 SV=2	60	8	10.9
Q9Y3D3	28S ribosomal protein S16, mitochondrial OS=Homo sapiens GN=MRPS16 PE=1 SV=1	60	6	15.3
Q9Y2Q5	Ragulator complex protein LAMTOR2 OS=Homo sapiens GN=LAMTOR2 PE=1 SV=1	60	6	13.5
P01040	Cystatin-A OS=Homo sapiens GN=CSTA PE=1 SV=1	60	4	11

P42704	Leucine-rich PPR motif-containing protein, mitochondrial OS=Homo sapiens GN=LRPPRC PE=1 SV=3	59	70	157.8
O60488	Long-chain-fatty-acid--CoA ligase 4 OS=Homo sapiens GN=ACSL4 PE=1 SV=2	59	32	79.1
O95573	Long-chain-fatty-acid--CoA ligase 3 OS=Homo sapiens GN=ACSL3 PE=1 SV=3	59	28	80.4
P31040	Succinate dehydrogenase [ubiquinone] flavoprotein subunit, mitochondrial OS=Homo sapiens GN=SDHA PE=1 SV=2	59	27	72.6
P13995	Bifunctional methylenetetrahydrofolate dehydrogenase/cyclohydrolase, mitochondrial OS=Homo sapiens GN=MTHFD2 PE=1 SV=2	59	14	37.9
Q9NUQ9	Protein FAM49B OS=Homo sapiens GN=FAM49B PE=1 SV=1	59	13	36.7
P62241	40S ribosomal protein S8 OS=Homo sapiens GN=RPS8 PE=1 SV=2	59	12	24.2
O00560	Syntenin-1 OS=Homo sapiens GN=SDCBP PE=1 SV=1	59	8	32.4
P30050	60S ribosomal protein L12 OS=Homo sapiens GN=RPL12 PE=1 SV=1	59	7	17.8
J3KNF8	Cytochrome b5 type B OS=Homo sapiens GN=CYB5B PE=1 SV=1	59	7	16.7
P62879	Guanine nucleotide-binding protein G(I)/G(S)/G(T) subunit beta-2 OS=Homo sapiens GN=GNB2 PE=1 SV=3	59	7	37.3
O14925	Mitochondrial import inner membrane translocase subunit Tim23 OS=Homo sapiens GN=TIMM23 PE=1 SV=1	59	7	21.9
P15153	Ras-related C3 botulinum toxin substrate 2 OS=Homo sapiens GN=RAC2 PE=1 SV=1	59	6	21.4
A0A075B746	28S ribosomal protein S21, mitochondrial OS=Homo sapiens GN=MRPS21 PE=1 SV=1	59	5	10.7
P62140	Serine/threonine-protein phosphatase PP1-beta catalytic subunit OS=Homo sapiens GN=PPP1CB PE=1 SV=3	59	5	37.2
P12236	ADP/ATP translocase 3 OS=Homo sapiens GN=SLC25A6 PE=1 SV=4	59	4	32.8
P14625	Endoplasmin OS=Homo sapiens GN=HSP90B1 PE=1 SV=1	58	53	92.4
P11021	78 kDa glucose-regulated protein OS=Homo sapiens GN=HSPA5 PE=1 SV=2	58	40	72.3
P27824	Calnexin OS=Homo sapiens GN=CANX PE=1 SV=2	58	36	67.5
O14672	Disintegrin and metalloproteinase domain-containing protein 10 OS=Homo sapiens GN=ADAM10 PE=1 SV=1	58	31	84.1
P04844	Dolichyl-diphosphooligosaccharide--protein glycosyltransferase subunit 2 OS=Homo sapiens GN=RPN2 PE=1 SV=3	58	29	69.2
Q9Y512	Sorting and assembly machinery component 50 homolog OS=Homo sapiens GN=SAMM50 PE=1 SV=3	58	23	51.9
O75083	WD repeat-containing protein 1 OS=Homo sapiens GN=WDR1 PE=1 SV=4	58	22	66.2
P00505	Aspartate aminotransferase, mitochondrial OS=Homo sapiens GN=GOT2 PE=1 SV=3	58	21	47.5
Q16795	NADH dehydrogenase [ubiquinone] 1 alpha subcomplex subunit 9, mitochondrial OS=Homo sapiens GN=NDUFA9 PE=1 SV=2	58	19	42.5
O75306	NADH dehydrogenase [ubiquinone] iron-sulfur protein 2, mitochondrial OS=Homo sapiens GN=NDUFS2 PE=1 SV=2	58	19	52.5

Q9UJZ1	Stomatin-like protein 2, mitochondrial OS=Homo sapiens GN=STOML2 PE=1 SV=1	58	19	38.5
Q9P015	39S ribosomal protein L15, mitochondrial OS=Homo sapiens GN=MRPL15 PE=1 SV=1	58	15	33.4
P05388	60S acidic ribosomal protein P0 OS=Homo sapiens GN=RPLP0 PE=1 SV=1	58	14	34.3
P21912	Succinate dehydrogenase [ubiquinone] iron-sulfur subunit, mitochondrial OS=Homo sapiens GN=SDHB PE=1 SV=3	58	14	31.6
O96000	NADH dehydrogenase [ubiquinone] 1 beta subcomplex subunit 10 OS=Homo sapiens GN=NDUFB10 PE=1 SV=3	58	12	20.8
Q9UFN0	Protein NipSnap homolog 3A OS=Homo sapiens GN=NIPSNAP3A PE=1 SV=2	58	11	28.4
P25786	Proteasome subunit alpha type-1 OS=Homo sapiens GN=PSMA1 PE=1 SV=1	58	10	29.5
P61981	14-3-3 protein gamma OS=Homo sapiens GN=YWHAG PE=1 SV=2	58	9	28.3
Q9HAV0	Guanine nucleotide-binding protein subunit beta-4 OS=Homo sapiens GN=GNB4 PE=1 SV=3	58	9	37.5
Q15365	Poly(rC)-binding protein 1 OS=Homo sapiens GN=PCBP1 PE=1 SV=2	58	9	37.5
O15511	Actin-related protein 2/3 complex subunit 5 OS=Homo sapiens GN=ARPC5 PE=1 SV=3	58	8	16.3
P11234	Ras-related protein Ral-B OS=Homo sapiens GN=RALB PE=1 SV=1	58	8	23.4
P61626	Lysozyme C OS=Homo sapiens GN=LYZ PE=1 SV=1	58	7	16.5
Q9BX68	Histidine triad nucleotide-binding protein 2, mitochondrial OS=Homo sapiens GN=HINT2 PE=1 SV=1	58	6	17.2
Q86Y39	NADH dehydrogenase [ubiquinone] 1 alpha subcomplex subunit 11 OS=Homo sapiens GN=NDUFA11 PE=1 SV=3	58	6	14.8
P10319	HLA class I histocompatibility antigen, B-58 alpha chain OS=Homo sapiens GN=HLA-B PE=1 SV=1	58	2	40.3
A0A140T9J9	HLA class I histocompatibility antigen, Cw-6 alpha chain OS=Homo sapiens GN=HLA-C PE=1 SV=1	58	2	41.6
P05107	Integrin beta-2 OS=Homo sapiens GN=ITGB2 PE=1 SV=2	58	2	84.7
A0A087WVQ6	Clathrin heavy chain OS=Homo sapiens GN=CLTC PE=1 SV=1	57	78	191.9
O95202	LETM1 and EF-hand domain-containing protein 1, mitochondrial OS=Homo sapiens GN=LETM1 PE=1 SV=1	57	41	83.3
Q13724	Mannosyl-oligosaccharide glucosidase OS=Homo sapiens GN=MOGS PE=1 SV=5	57	34	91.9
P25705	ATP synthase subunit alpha, mitochondrial OS=Homo sapiens GN=ATP5A1 PE=1 SV=1	57	32	59.7
Q15833	Syntaxin-binding protein 2 OS=Homo sapiens GN=STXBP2 PE=1 SV=2	57	30	66.4
Q9HDC9	Adipocyte plasma membrane-associated protein OS=Homo sapiens GN=APMAP PE=1 SV=2	57	24	46.5
P78371	T-complex protein 1 subunit beta OS=Homo sapiens GN=CCT2 PE=1 SV=4	57	24	57.5
Q13217	DnaJ homolog subfamily C member 3 OS=Homo sapiens GN=DNAJC3 PE=1 SV=1	57	23	57.5
A0A0C4DFL7	Lanosterol 14-alpha demethylase OS=Homo sapiens GN=CYP51A1 PE=1 SV=1	57	23	57.2

P24752	Acetyl-CoA acetyltransferase, mitochondrial OS=Homo sapiens GN=ACAT1 PE=1 SV=1	57	19	45.2
P45880	Voltage-dependent anion-selective channel protein 2 OS=Homo sapiens GN=VDAC2 PE=1 SV=2	57	12	31.5
P30044	Peroxiredoxin-5, mitochondrial OS=Homo sapiens GN=PRDX5 PE=1 SV=4	57	11	22.1
P26885	Peptidyl-prolyl cis-trans isomerase FKBP2 OS=Homo sapiens GN=FKBP2 PE=1 SV=2	57	6	15.6
P11233	Ras-related protein Ral-A OS=Homo sapiens GN=RALA PE=1 SV=1	57	6	23.6
P61204	ADP-ribosylation factor 3 OS=Homo sapiens GN=ARF3 PE=1 SV=2	57	5	20.6
A0A087X117	Nodal modulator 1 OS=Homo sapiens GN=NOMO1 PE=1 SV=1	57	3	139.4
P17927	Complement receptor type 1 OS=Homo sapiens GN=CR1 PE=1 SV=3	56	46	223.5
Q9Y4L1	Hypoxia up-regulated protein 1 OS=Homo sapiens GN=HYOU1 PE=1 SV=1	56	45	111.3
Q02218	2-oxoglutarate dehydrogenase, mitochondrial OS=Homo sapiens GN=OGDH PE=1 SV=3	56	43	115.9
Q14254	Flotillin-2 OS=Homo sapiens GN=FLOT2 PE=1 SV=2	56	24	47
P29350	Tyrosine-protein phosphatase non-receptor type 6 OS=Homo sapiens GN=PTPN6 PE=1 SV=1	56	24	67.5
O75746	Calcium-binding mitochondrial carrier protein Aralar1 OS=Homo sapiens GN=SLC25A12 PE=1 SV=2	56	20	74.7
Q9Y276	Mitochondrial chaperone BCS1 OS=Homo sapiens GN=BCS1L PE=1 SV=1	56	20	47.5
Q8NBS9	Thioredoxin domain-containing protein 5 OS=Homo sapiens GN=TXNDC5 PE=1 SV=2	56	20	47.6
Q6YN16	Hydroxysteroid dehydrogenase-like protein 2 OS=Homo sapiens GN=HSDL2 PE=1 SV=1	56	18	45.4
Q8N2K0	Monoacylglycerol lipase ABHD12 OS=Homo sapiens GN=ABHD12 PE=1 SV=2	56	17	45.1
P14598	Neutrophil cytosol factor 1 OS=Homo sapiens GN=NCF1 PE=1 SV=3	56	13	44.6
P08311	Cathepsin G OS=Homo sapiens GN=CTSG PE=1 SV=2	56	12	28.8
Q08257	Quinone oxidoreductase OS=Homo sapiens GN=CRYZ PE=1 SV=1	56	12	35.2
O75323	Protein NipSnap homolog 2 OS=Homo sapiens GN=GBAS PE=1 SV=1	56	11	33.7
Q99653	Calcineurin B homologous protein 1 OS=Homo sapiens GN=CHP1 PE=1 SV=3	56	9	22.4
P52566	Rho GDP-dissociation inhibitor 2 OS=Homo sapiens GN=ARHGDIB PE=1 SV=3	56	8	23
P62873	Guanine nucleotide-binding protein G(I)/G(S)/G(T) subunit beta-1 OS=Homo sapiens GN=GNB1 PE=1 SV=3	56	7	37.4
J3KQ48	Peptidyl-tRNA hydrolase 2, mitochondrial OS=Homo sapiens GN=PTRH2 PE=1 SV=1	56	7	19.3
Q6IBS0	Twinfilin-2 OS=Homo sapiens GN=TWF2 PE=1 SV=2	56	7	39.5
O75380	NADH dehydrogenase [ubiquinone] iron-sulfur protein 6, mitochondrial OS=Homo sapiens GN=NDUFS6 PE=1 SV=1	56	6	13.7
Q9NVJ2	ADP-ribosylation factor-like protein 8B OS=Homo sapiens GN=ARL8B PE=1 SV=1	56	5	21.5

Q9H4G4	Golgi-associated plant pathogenesis-related protein 1 OS=Homo sapiens GN=GLIPR2 PE=1 SV=3	56	5	17.2
P63000	Ras-related C3 botulinum toxin substrate 1 OS=Homo sapiens GN=RAC1 PE=1 SV=1	56	5	21.4
Q14061	Cytochrome c oxidase copper chaperone OS=Homo sapiens GN=COX17 PE=1 SV=2	56	3	6.9
Q9P0U1	Mitochondrial import receptor subunit TOM7 homolog OS=Homo sapiens GN=TOMM7 PE=1 SV=1	56	2	6.2
P16284	Platelet endothelial cell adhesion molecule OS=Homo sapiens GN=PECAM1 PE=1 SV=1	55	31	82.5
Q6NUK1	Calcium-binding mitochondrial carrier protein SCaMC-1 OS=Homo sapiens GN=SLC25A24 PE=1 SV=2	55	27	53.3
Q16134	Electron transfer flavoprotein-ubiquinone oxidoreductase, mitochondrial OS=Homo sapiens GN=ETFDH PE=1 SV=2	55	24	68.5
E7EQR4	Ezrin OS=Homo sapiens GN=EZR PE=1 SV=3	55	22	69.3
Q9BTV4	Transmembrane protein 43 OS=Homo sapiens GN=TMEM43 PE=1 SV=1	55	22	44.8
Q96I99	Succinate--CoA ligase [GDP-forming] subunit beta, mitochondrial OS=Homo sapiens GN=SUCLG2 PE=1 SV=2	55	20	46.5
P68104	Elongation factor 1-alpha 1 OS=Homo sapiens GN=EEF1A1 PE=1 SV=1	55	18	50.1
P04083	Annexin A1 OS=Homo sapiens GN=ANXA1 PE=1 SV=2	55	17	38.7
Q02978	Mitochondrial 2-oxoglutarate/malate carrier protein OS=Homo sapiens GN=SLC25A11 PE=1 SV=3	55	16	34
H0Y2W2	ATPase family AAA domain-containing protein 3A (Fragment) OS=Homo sapiens GN=ATAD3A PE=1 SV=1	55	15	64.3
P53701	Cytochrome c-type heme lyase OS=Homo sapiens GN=HCCS PE=1 SV=1	55	15	30.6
Q02338	D-beta-hydroxybutyrate dehydrogenase, mitochondrial OS=Homo sapiens GN=BDH1 PE=1 SV=3	55	13	38.1
O75477	Erlin-1 OS=Homo sapiens GN=ERLIN1 PE=1 SV=1	55	13	38.9
P13727	Bone marrow proteoglycan OS=Homo sapiens GN=PRG2 PE=1 SV=2	55	12	25.2
P61026	Ras-related protein Rab-10 OS=Homo sapiens GN=RAB10 PE=1 SV=1	55	10	22.5
P10606	Cytochrome c oxidase subunit 5B, mitochondrial OS=Homo sapiens GN=COX5B PE=1 SV=2	55	9	13.7
Q9BV40	Vesicle-associated membrane protein 8 OS=Homo sapiens GN=VAMP8 PE=1 SV=1	55	8	11.4
O75964	ATP synthase subunit g, mitochondrial OS=Homo sapiens GN=ATP5L PE=1 SV=3	55	7	11.4
O43920	NADH dehydrogenase [ubiquinone] iron-sulfur protein 5 OS=Homo sapiens GN=NDUFS5 PE=1 SV=3	55	7	12.5
P61769	Beta-2-microglobulin OS=Homo sapiens GN=B2M PE=1 SV=1	55	4	13.7
P12235	ADP/ATP translocase 1 OS=Homo sapiens GN=SLC25A4 PE=1 SV=4	55	2	33
P23786	Carnitine O-palmitoyltransferase 2, mitochondrial OS=Homo sapiens GN=CPT2 PE=1 SV=2	54	28	73.7
Q9UBV2	Protein sel-1 homolog 1 OS=Homo sapiens GN=SEL1L PE=1 SV=3	54	23	88.7
P49257	Protein ERGIC-53 OS=Homo sapiens GN=LMAN1 PE=1 SV=2	54	20	57.5

Q7L0Y3	Mitochondrial ribonuclease P protein 1 OS=Homo sapiens GN=TRMT10C PE=1 SV=2	54	18	47.3
P08758	Annexin A5 OS=Homo sapiens GN=ANXA5 PE=1 SV=2	54	15	35.9
Q9UL25	Ras-related protein Rab-21 OS=Homo sapiens GN=RAB21 PE=1 SV=3	54	13	24.3
P63244	Receptor of activated protein C kinase 1 OS=Homo sapiens GN=RACK1 PE=1 SV=3	54	13	35.1
P30084	Enoyl-CoA hydratase, mitochondrial OS=Homo sapiens GN=ECHS1 PE=1 SV=4	54	12	31.4
Q9HAV7	GrpE protein homolog 1, mitochondrial OS=Homo sapiens GN=GRPEL1 PE=1 SV=2	54	11	24.3
P51159	Ras-related protein Rab-27A OS=Homo sapiens GN=RAB27A PE=1 SV=3	54	11	24.9
C9JJ19	28S ribosomal protein S34, mitochondrial OS=Homo sapiens GN=MRPS34 PE=1 SV=2	54	10	26.3
Q13637	Ras-related protein Rab-32 OS=Homo sapiens GN=RAB32 PE=1 SV=3	54	10	25
Q9Y3D9	28S ribosomal protein S23, mitochondrial OS=Homo sapiens GN=MRPS23 PE=1 SV=2	54	8	21.8
Q9UL26	Ras-related protein Rab-22A OS=Homo sapiens GN=RAB22A PE=1 SV=2	54	5	21.8
Q9BPX5	Actin-related protein 2/3 complex subunit 5-like protein OS=Homo sapiens GN=ARPC5L PE=1 SV=1	54	4	16.9
P00441	Superoxide dismutase [Cu-Zn] OS=Homo sapiens GN=SOD1 PE=1 SV=2	54	4	15.9
F8W914	Reticulon OS=Homo sapiens GN=RTN4 PE=1 SV=1	54	2	37.1
P04264	Keratin, type II cytoskeletal 1 OS=Homo sapiens GN=KRT1 PE=1 SV=6	53	31	66
Q9NTJ5	Phosphatidylinositide phosphatase SAC1 OS=Homo sapiens GN=SACM1L PE=1 SV=2	53	29	66.9
A0A024R571	EH domain-containing protein 1 OS=Homo sapiens GN=EHD1 PE=1 SV=1	53	24	61.9
Q7L5N7	Lysophosphatidylcholine acyltransferase 2 OS=Homo sapiens GN=LPCAT2 PE=1 SV=1	53	24	60.2
O14975	Very long-chain acyl-CoA synthetase OS=Homo sapiens GN=SLC27A2 PE=1 SV=2	53	24	70.3
P13674	Prolyl 4-hydroxylase subunit alpha-1 OS=Homo sapiens GN=P4HA1 PE=1 SV=2	53	23	61
Q9UJS0	Calcium-binding mitochondrial carrier protein Aralar2 OS=Homo sapiens GN=SLC25A13 PE=1 SV=2	53	22	74.1
Q9Y305	Acyl-coenzyme A thioesterase 9, mitochondrial OS=Homo sapiens GN=ACOT9 PE=1 SV=2	53	21	49.9
A0A087WSV8	Nucleobindin 2, isoform CRA_b OS=Homo sapiens GN=NUCB2 PE=1 SV=1	53	19	50.2
E7ESZ7	NADH dehydrogenase [ubiquinone] 1 alpha subcomplex subunit 10, mitochondrial OS=Homo sapiens GN=NDUFA10 PE=1 SV=1	53	18	44.7
P09110	3-ketoacyl-CoA thiolase, peroxisomal OS=Homo sapiens GN=ACAA1 PE=1 SV=2	53	17	44.3
P07339	Cathepsin D OS=Homo sapiens GN=CTSD PE=1 SV=1	53	17	44.5
Q14847	LIM and SH3 domain protein 1 OS=Homo sapiens GN=LASP1 PE=1 SV=2	53	12	29.7
P00338	L-lactate dehydrogenase A chain OS=Homo sapiens GN=LDHA PE=1 SV=2	53	12	36.7

Q96EY8	Cob(I)yrinic acid a,c-diamide adenosyltransferase, mitochondrial OS=Homo sapiens GN=MMAB PE=1 SV=1	53	11	27.4
P27105	Erythrocyte band 7 integral membrane protein OS=Homo sapiens GN=STOM PE=1 SV=3	53	11	31.7
A0A087WYR3	Tumor protein D54 OS=Homo sapiens GN=TPD52L2 PE=1 SV=1	53	10	23.8
Q9Y6M9	NADH dehydrogenase [ubiquinone] 1 beta subcomplex subunit 9 OS=Homo sapiens GN=NDUFB9 PE=1 SV=3	53	9	21.8
A0A096LPI6	Uncharacterized protein (Fragment) OS=Homo sapiens PE=4 SV=2	53	9	30.5
P62805	Histone H4 OS=Homo sapiens GN=HIST1H4A PE=1 SV=2	53	8	11.4
Q9BUR5	MICOS complex subunit MIC26 OS=Homo sapiens GN=APOO PE=1 SV=1	53	7	22.3
P60953	Cell division control protein 42 homolog OS=Homo sapiens GN=CDC42 PE=1 SV=2	53	6	21.2
Q9Y5L4	Mitochondrial import inner membrane translocase subunit Tim13 OS=Homo sapiens GN=TIMM13 PE=1 SV=1	53	4	10.5
P03928	ATP synthase protein 8 OS=Homo sapiens GN=MT-ATP8 PE=1 SV=1	53	3	8
A0A140TA86	MICOS complex subunit MIC13 OS=Homo sapiens GN=C19orf70 PE=1 SV=1	53	3	15.4
O60220	Mitochondrial import inner membrane translocase subunit Tim8 A OS=Homo sapiens GN=TIMM8A PE=1 SV=1	53	3	11
A8MVG7	Integrin beta-2 OS=Homo sapiens GN=ITGB2 PE=1 SV=1	53	2	11.4
Q9NYU2	UDP-glucose:glycoprotein glucosyltransferase 1 OS=Homo sapiens GN=UGGT1 PE=1 SV=3	52	58	177.1
P36776	Lon protease homolog, mitochondrial OS=Homo sapiens GN=LONP1 PE=1 SV=2	52	47	106.4
Q969V3	Nicalin OS=Homo sapiens GN=NCLN PE=1 SV=2	52	27	62.9
P32189	Glycerol kinase OS=Homo sapiens GN=GK PE=1 SV=3	52	23	61.2
P41240	Tyrosine-protein kinase CSK OS=Homo sapiens GN=CSK PE=1 SV=1	52	18	50.7
Q9H2U2	Inorganic pyrophosphatase 2, mitochondrial OS=Homo sapiens GN=PPA2 PE=1 SV=2	52	16	37.9
Q9NX40	OCIA domain-containing protein 1 OS=Homo sapiens GN=OCIAD1 PE=1 SV=1	52	14	27.6
Q9H7Z7	Prostaglandin E synthase 2 OS=Homo sapiens GN=PTGES2 PE=1 SV=1	52	14	41.9
P54709	Sodium/potassium-transporting ATPase subunit beta-3 OS=Homo sapiens GN=ATP1B3 PE=1 SV=1	52	14	31.5
Q9HCU5	Prolactin regulatory element-binding protein OS=Homo sapiens GN=PREB PE=1 SV=2	52	13	45.4
Q9NPL8	Complex I assembly factor TIMMDC1, mitochondrial OS=Homo sapiens GN=TIMMDC1 PE=1 SV=2	52	12	32.2
P62258	14-3-3 protein epsilon OS=Homo sapiens GN=YWHAE PE=1 SV=1	52	11	29.2
P63104	14-3-3 protein zeta/delta OS=Homo sapiens GN=YWHAZ PE=1 SV=1	52	11	27.7
P36222	Chitinase-3-like protein 1 OS=Homo sapiens GN=CHI3L1 PE=1 SV=2	52	11	42.6
A0A0C4DG17	40S ribosomal protein SA OS=Homo sapiens GN=RPSA PE=1 SV=1	52	10	33.3

O15173	Membrane-associated progesterone receptor component 2 OS=Homo sapiens GN=PGRMC2 PE=1 SV=1	52	10	23.8
P30048	Thioredoxin-dependent peroxide reductase, mitochondrial OS=Homo sapiens GN=PRDX3 PE=1 SV=3	52	10	27.7
P05141	ADP/ATP translocase 2 OS=Homo sapiens GN=SLC25A5 PE=1 SV=7	52	9	32.8
F8WCF6	Actin-related protein 2/3 complex subunit 4 OS=Homo sapiens GN=ARPC4-TTLL3 PE=3 SV=1	52	8	21
Q5VST6	Protein ABHD17B OS=Homo sapiens GN=ABHD17B PE=1 SV=1	52	8	32.2
Q96AB3	Isochorismatase domain-containing protein 2 OS=Homo sapiens GN=ISOC2 PE=1 SV=1	52	6	22.3
Q9Y490	Talin-1 OS=Homo sapiens GN=TLN1 PE=1 SV=3	51	94	269.6
P05023	Sodium/potassium-transporting ATPase subunit alpha-1 OS=Homo sapiens GN=ATP1A1 PE=1 SV=1	51	40	112.8
K7ELL7	Glucosidase 2 subunit beta OS=Homo sapiens GN=PRKCSH PE=1 SV=1	51	24	60.2
Q9BTU6	Phosphatidylinositol 4-kinase type 2-alpha OS=Homo sapiens GN=PI4K2A PE=1 SV=1	51	21	54
Q92552	28S ribosomal protein S27, mitochondrial OS=Homo sapiens GN=MRPS27 PE=1 SV=3	51	18	47.6
O75439	Mitochondrial-processing peptidase subunit beta OS=Homo sapiens GN=PMPCB PE=1 SV=2	51	17	54.3
Q9H3M7	Thioredoxin-interacting protein OS=Homo sapiens GN=TXNIP PE=1 SV=1	51	17	43.6
P46781	40S ribosomal protein S9 OS=Homo sapiens GN=RPS9 PE=1 SV=3	51	15	22.6
Q92643	GPI-anchor transamidase OS=Homo sapiens GN=PIGK PE=1 SV=2	51	15	45.2
Q9GZT6	Coiled-coil domain-containing protein 90B, mitochondrial OS=Homo sapiens GN=CCDC90B PE=1 SV=2	51	13	29.5
P23284	Peptidyl-prolyl cis-trans isomerase B OS=Homo sapiens GN=PPIB PE=1 SV=2	51	13	23.7
Q53GQ0	Very-long-chain 3-oxoacyl-CoA reductase OS=Homo sapiens GN=HSD17B12 PE=1 SV=2	51	13	34.3
Q16762	Thiosulfate sulfurtransferase OS=Homo sapiens GN=TST PE=1 SV=4	51	12	33.4
Q53H82	Endoribonuclease LACTB2 OS=Homo sapiens GN=LACTB2 PE=1 SV=2	51	11	32.8
P62269	40S ribosomal protein S18 OS=Homo sapiens GN=RPS18 PE=1 SV=3	51	10	17.7
Q9Y277	Voltage-dependent anion-selective channel protein 3 OS=Homo sapiens GN=VDAC3 PE=1 SV=1	51	10	30.6
P50148	Guanine nucleotide-binding protein G(q) subunit alpha OS=Homo sapiens GN=GNAQ PE=1 SV=4	51	9	42.1
Q96A35	39S ribosomal protein L24, mitochondrial OS=Homo sapiens GN=MRPL24 PE=1 SV=1	51	8	24.9
P02792	Ferritin light chain OS=Homo sapiens GN=FTL PE=1 SV=2	51	8	20
Q8N183	Mimitin, mitochondrial OS=Homo sapiens GN=NDUFAF2 PE=1 SV=1	51	7	19.8
P62937	Peptidyl-prolyl cis-trans isomerase A OS=Homo sapiens GN=PPIA PE=1 SV=2	51	7	18
Q9BU61	NADH dehydrogenase [ubiquinone] 1 alpha subcomplex assembly factor 3 OS=Homo sapiens GN=NDUFAF3 PE=1 SV=1	51	6	20.3

P62888	60S ribosomal protein L30 OS=Homo sapiens GN=RPL30 PE=1 SV=2	51	5	12.8
Q9NS69	Mitochondrial import receptor subunit TOM22 homolog OS=Homo sapiens GN=TOMM22 PE=1 SV=3	51	5	15.5
O75531	Barrier-to-autointegration factor OS=Homo sapiens GN=BANF1 PE=1 SV=1	51	4	10.1
Q5TGZ0	MICOS complex subunit MIC10 OS=Homo sapiens GN=MINOS1 PE=1 SV=1	51	2	8.8
A0A0A0MT22	Protein tyrosine phosphatase, receptor type, C, isoform CRA_d OS=Homo sapiens GN=PTPRC PE=1 SV=1	50	70	147.4
P49748	Very long-chain specific acyl-CoA dehydrogenase, mitochondrial OS=Homo sapiens GN=ACADVL PE=1 SV=1	50	28	70.3
O94826	Mitochondrial import receptor subunit TOM70 OS=Homo sapiens GN=TOMM70 PE=1 SV=1	50	25	67.4
P11142	Heat shock cognate 71 kDa protein OS=Homo sapiens GN=HSPA8 PE=1 SV=1	50	24	70.9
P48735	Isocitrate dehydrogenase [NADP], mitochondrial OS=Homo sapiens GN=IDH2 PE=1 SV=2	50	23	50.9
P83111	Serine beta-lactamase-like protein LACTB, mitochondrial OS=Homo sapiens GN=LACTB PE=1 SV=2	50	23	60.7
P11413	Glucose-6-phosphate 1-dehydrogenase OS=Homo sapiens GN=G6PD PE=1 SV=4	50	22	59.2
P49368	T-complex protein 1 subunit gamma OS=Homo sapiens GN=CCT3 PE=1 SV=4	50	22	60.5
P50552	Vasodilator-stimulated phosphoprotein OS=Homo sapiens GN=VASP PE=1 SV=3	50	22	39.8
Q01518	Adenylyl cyclase-associated protein 1 OS=Homo sapiens GN=CAP1 PE=1 SV=5	50	20	51.9
P29401	Transketolase OS=Homo sapiens GN=TKT PE=1 SV=3	50	20	67.8
P05120	Plasminogen activator inhibitor 2 OS=Homo sapiens GN=SERPINB2 PE=1 SV=2	50	16	46.6
P36957	Dihydrolipoyllysine-residue succinyltransferase component of 2-oxoglutarate dehydrogenase complex, mitochondrial OS=Homo sapiens GN=DLST PE=1 SV=4	50	15	48.7
P62701	40S ribosomal protein S4, X isoform OS=Homo sapiens GN=RPS4X PE=1 SV=2	50	14	29.6
P18124	60S ribosomal protein L7 OS=Homo sapiens GN=RPL7 PE=1 SV=1	50	14	29.2
C9JRZ6	MICOS complex subunit OS=Homo sapiens GN=CHCHD3 PE=1 SV=1	50	14	26.7
E7EPT4	NADH dehydrogenase [ubiquinone] flavoprotein 2, mitochondrial OS=Homo sapiens GN=NDUFV2 PE=1 SV=1	50	12	27.9
Q9Y676	28S ribosomal protein S18b, mitochondrial OS=Homo sapiens GN=MRPS18B PE=1 SV=1	50	9	29.4
Q5HYB6	Epididymis luminal protein 189 OS=Homo sapiens GN=DKFZp686J1372 PE=1 SV=1	50	9	27.2
Q99807	5-demethoxyubiquinone hydroxylase, mitochondrial OS=Homo sapiens GN=COQ7 PE=1 SV=3	50	8	24.3
Q8N5K1	CDGSH iron-sulfur domain-containing protein 2 OS=Homo sapiens GN=CISD2 PE=1 SV=1	50	7	15.3
P01033	Metalloproteinase inhibitor 1 OS=Homo sapiens GN=TIMP1 PE=1 SV=1	50	7	23.2
Q92882	Osteoclast-stimulating factor 1 OS=Homo sapiens GN=OSTF1 PE=1 SV=2	50	7	23.8
P47755	F-actin-capping protein subunit alpha-2 OS=Homo sapiens GN=CAPZA2 PE=1 SV=3	50	6	32.9

Q13438	Protein OS-9 OS=Homo sapiens GN=OS9 PE=1 SV=1	50	6	75.5
L0R6Q1	SLC35A4 upstream open reading frame protein OS=Homo sapiens GN=SLC35A4 PE=3 SV=1	50	5	11.1
Q8N766	ER membrane protein complex subunit 1 OS=Homo sapiens GN=EMC1 PE=1 SV=1	49	36	111.7
P31146	Coronin-1A OS=Homo sapiens GN=CORO1A PE=1 SV=4	49	24	51
P13645	Keratin, type I cytoskeletal 10 OS=Homo sapiens GN=KRT10 PE=1 SV=6	49	22	58.8
Q15067	Peroxisomal acyl-coenzyme A oxidase 1 OS=Homo sapiens GN=ACOX1 PE=1 SV=3	49	22	74.4
P28838	Cytosol aminopeptidase OS=Homo sapiens GN=LAP3 PE=1 SV=3	49	20	56.1
Q8NE86	Calcium uniporter protein, mitochondrial OS=Homo sapiens GN=MCU PE=1 SV=1	49	19	39.8
P39656	Dolichyl-diphosphooligosaccharide--protein glycosyltransferase 48 kDa subunit OS=Homo sapiens GN=DDOST PE=1 SV=4	49	16	50.8
Q14165	Malectin OS=Homo sapiens GN=MLEC PE=1 SV=1	49	16	32.2
A0A087WT44	Heme oxygenase 2 OS=Homo sapiens GN=HMOX2 PE=1 SV=1	49	14	41.6
Q9P032	NADH dehydrogenase [ubiquinone] 1 alpha subcomplex assembly factor 4 OS=Homo sapiens GN=NDUFAF4 PE=1 SV=1	49	9	20.3
O00217	NADH dehydrogenase [ubiquinone] iron-sulfur protein 8, mitochondrial OS=Homo sapiens GN=NDUFS8 PE=1 SV=1	49	9	23.7
P39019	40S ribosomal protein S19 OS=Homo sapiens GN=RPS19 PE=1 SV=2	49	8	16.1
P30405	Peptidyl-prolyl cis-trans isomerase F, mitochondrial OS=Homo sapiens GN=PPIF PE=1 SV=1	49	6	22
P99999	Cytochrome c OS=Homo sapiens GN=CYCS PE=1 SV=2	49	5	11.7
Q6P161	39S ribosomal protein L54, mitochondrial OS=Homo sapiens GN=MRPL54 PE=1 SV=1	49	3	15.8
P13639	Elongation factor 2 OS=Homo sapiens GN=EEF2 PE=1 SV=4	48	33	95.3
Q9UGP8	Translocation protein SEC63 homolog OS=Homo sapiens GN=SEC63 PE=1 SV=2	48	32	87.9
Q12931	Heat shock protein 75 kDa, mitochondrial OS=Homo sapiens GN=TRAP1 PE=1 SV=3	48	28	80.1
Q8NBJ5	Procollagen galactosyltransferase 1 OS=Homo sapiens GN=COLGALT1 PE=1 SV=1	48	25	71.6
Q86UX7	Fermitin family homolog 3 OS=Homo sapiens GN=FERMT3 PE=1 SV=1	48	22	75.9
A0A0A0MTS2	Glucose-6-phosphate isomerase (Fragment) OS=Homo sapiens GN=GPI PE=1 SV=1	48	19	64.8
O75390	Citrate synthase, mitochondrial OS=Homo sapiens GN=CS PE=1 SV=2	48	18	51.7
P09622	Dihydrolipoyl dehydrogenase, mitochondrial OS=Homo sapiens GN=DLD PE=1 SV=2	48	18	54.1
Q10713	Mitochondrial-processing peptidase subunit alpha OS=Homo sapiens GN=PMPCA PE=1 SV=2	48	18	58.2
P37268	Squalene synthase OS=Homo sapiens GN=FDFT1 PE=1 SV=1	48	18	48.1

P61160	Actin-related protein 2 OS=Homo sapiens GN=ACTR2 PE=1 SV=1	48	17	44.7
Q9BS26	Endoplasmic reticulum resident protein 44 OS=Homo sapiens GN=ERP44 PE=1 SV=1	48	16	46.9
Q96CS3	FAS-associated factor 2 OS=Homo sapiens GN=FAF2 PE=1 SV=2	48	16	52.6
P55809	Succinyl-CoA:3-ketoacid coenzyme A transferase 1, mitochondrial OS=Homo sapiens GN=OXCT1 PE=1 SV=1	48	15	56.1
O14656	Torsin-1A OS=Homo sapiens GN=TOR1A PE=1 SV=1	48	12	37.8
Q9Y5K8	V-type proton ATPase subunit D OS=Homo sapiens GN=ATP6V1D PE=1 SV=1	48	11	28.2
P48060	Glioma pathogenesis-related protein 1 OS=Homo sapiens GN=GLIPR1 PE=1 SV=3	48	9	30.3
Q7Z4W1	L-xylulose reductase OS=Homo sapiens GN=DCXR PE=1 SV=2	48	9	25.9
P29966	Myristoylated alanine-rich C-kinase substrate OS=Homo sapiens GN=MARCKS PE=1 SV=4	48	9	31.5
P61088	Ubiquitin-conjugating enzyme E2 N OS=Homo sapiens GN=UBE2N PE=1 SV=1	48	7	17.1
P27348	14-3-3 protein theta OS=Homo sapiens GN=YWHAQ PE=1 SV=1	48	6	27.7
Q9Y2J2	Band 4.1-like protein 3 OS=Homo sapiens GN=EPB41L3 PE=1 SV=2	48	6	120.6
P20674	Cytochrome c oxidase subunit 5A, mitochondrial OS=Homo sapiens GN=COX5A PE=1 SV=2	48	6	16.8
O60830	Mitochondrial import inner membrane translocase subunit Tim17-B OS=Homo sapiens GN=TIMM17B PE=1 SV=1	48	6	18.3
O43678	NADH dehydrogenase [ubiquinone] 1 alpha subcomplex subunit 2 OS=Homo sapiens GN=NDUFA2 PE=1 SV=3	48	5	10.9
P61020	Ras-related protein Rab-5B OS=Homo sapiens GN=RAB5B PE=1 SV=1	48	5	23.7
Q13813	Spectrin alpha chain, non-erythrocytic 1 OS=Homo sapiens GN=SPTAN1 PE=1 SV=3	47	92	284.4
P02786	Transferrin receptor protein 1 OS=Homo sapiens GN=TFRC PE=1 SV=2	47	30	84.8
P48449	Lanosterol synthase OS=Homo sapiens GN=LSS PE=1 SV=1	47	26	83.3
P08240	Signal recognition particle receptor subunit alpha OS=Homo sapiens GN=SRPRA PE=1 SV=2	47	25	69.8
P50990	T-complex protein 1 subunit theta OS=Homo sapiens GN=CCT8 PE=1 SV=4	47	24	59.6
P24539	ATP synthase F(0) complex subunit B1, mitochondrial OS=Homo sapiens GN=ATP5F1 PE=1 SV=2	47	18	28.9
Q14554	Protein disulfide-isomerase A5 OS=Homo sapiens GN=PDIA5 PE=1 SV=1	47	18	59.6
P09769	Tyrosine-protein kinase Fgr OS=Homo sapiens GN=FGR PE=1 SV=2	47	15	59.4
Q9UBS4	DnaJ homolog subfamily B member 11 OS=Homo sapiens GN=DNAJB11 PE=1 SV=1	47	14	40.5
A0A0A0MSE2	Hydroxyacyl-coenzyme A dehydrogenase, mitochondrial OS=Homo sapiens GN=HADH PE=1 SV=1	47	13	42.1
Q99536	Synaptic vesicle membrane protein VAT-1 homolog OS=Homo sapiens GN=VAT1 PE=1 SV=2	47	13	41.9
Q9Y5M8	Signal recognition particle receptor subunit beta OS=Homo sapiens GN=SRPRB PE=1 SV=3	47	12	29.7

Q14257	Reticulocalbin-2 OS=Homo sapiens GN=RCN2 PE=1 SV=1	47	11	36.9
P01137	Transforming growth factor beta-1 OS=Homo sapiens GN=TGFB1 PE=1 SV=2	47	11	44.3
P50395	Rab GDP dissociation inhibitor beta OS=Homo sapiens GN=GDI2 PE=1 SV=2	47	10	50.6
O95721	Synaptosomal-associated protein 29 OS=Homo sapiens GN=SNAP29 PE=1 SV=1	47	9	29
P05109	Protein S100-A8 OS=Homo sapiens GN=S100A8 PE=1 SV=1	47	8	10.8
P62249	40S ribosomal protein S16 OS=Homo sapiens GN=RPS16 PE=1 SV=2	47	7	16.4
P00492	Hypoxanthine-guanine phosphoribosyltransferase OS=Homo sapiens GN=HPRT1 PE=1 SV=2	47	7	24.6
Q7Z5G4	Golgin subfamily A member 7 OS=Homo sapiens GN=GOLGA7 PE=1 SV=2	47	6	15.8
P30086	Phosphatidylethanolamine-binding protein 1 OS=Homo sapiens GN=PEBP1 PE=1 SV=3	47	6	21
P01116	GTPase KRas OS=Homo sapiens GN=KRAS PE=1 SV=1	47	3	21.6
E5KLJ5	Dynamin-like 120 kDa protein, mitochondrial OS=Homo sapiens GN=OPA1 PE=1 SV=1	46	43	117.7
J3KPF3	4F2 cell-surface antigen heavy chain OS=Homo sapiens GN=SLC3A2 PE=1 SV=1	46	33	68.1
O60568	Procollagen-lysine,2-oxoglutarate 5-dioxygenase 3 OS=Homo sapiens GN=PLOD3 PE=1 SV=1	46	25	84.7
P07900	Heat shock protein HSP 90-alpha OS=Homo sapiens GN=HSP90AA1 PE=1 SV=5	46	24	84.6
P08238	Heat shock protein HSP 90-beta OS=Homo sapiens GN=HSP90AB1 PE=1 SV=4	46	20	83.2
P23141	Liver carboxylesterase 1 OS=Homo sapiens GN=CES1 PE=1 SV=2	46	20	62.5
P22234	Multifunctional protein ADE2 OS=Homo sapiens GN=PAICS PE=1 SV=3	46	16	47
P17213	Bactericidal permeability-increasing protein OS=Homo sapiens GN=BPI PE=1 SV=4	46	15	53.9
Q9NYK5	39S ribosomal protein L39, mitochondrial OS=Homo sapiens GN=MRPL39 PE=1 SV=3	46	14	38.7
Q9NUJ1	Mycophenolic acid acyl-glucuronide esterase, mitochondrial OS=Homo sapiens GN=ABHD10 PE=1 SV=1	46	12	33.9
A0A087X2D5	39S ribosomal protein L45, mitochondrial OS=Homo sapiens GN=MRPL45 PE=1 SV=1	46	11	35.3
Q5T9A4	ATPase family AAA domain-containing protein 3B OS=Homo sapiens GN=ATAD3B PE=1 SV=1	46	11	72.5
P82673	28S ribosomal protein S35, mitochondrial OS=Homo sapiens GN=MRPS35 PE=1 SV=1	46	10	36.8
P56589	Peroxisomal biogenesis factor 3 OS=Homo sapiens GN=PEX3 PE=1 SV=1	46	10	42.1
P61313	60S ribosomal protein L15 OS=Homo sapiens GN=RPL15 PE=1 SV=2	46	9	24.1
P30041	Peroxiredoxin-6 OS=Homo sapiens GN=PRDX6 PE=1 SV=3	46	8	25
Q15286	Ras-related protein Rab-35 OS=Homo sapiens GN=RAB35 PE=1 SV=1	46	8	23
Q86Y82	Syntaxin-12 OS=Homo sapiens GN=STX12 PE=1 SV=1	46	8	31.6

P49795	Regulator of G-protein signaling 19 OS=Homo sapiens GN=RGS19 PE=1 SV=1	46	7	24.6
O14949	Cytochrome b-c1 complex subunit 8 OS=Homo sapiens GN=UQCRQ PE=1 SV=4	46	6	9.9
Q5VV89	Microsomal glutathione S-transferase 3 OS=Homo sapiens GN=MGST3 PE=1 SV=1	46	6	18.4
O95563	Mitochondrial pyruvate carrier 2 OS=Homo sapiens GN=MPC2 PE=1 SV=1	46	6	14.3
P68032	Actin, alpha cardiac muscle 1 OS=Homo sapiens GN=ACTC1 PE=1 SV=1	46	5	42
Q01469	Fatty acid-binding protein, epidermal OS=Homo sapiens GN=FABP5 PE=1 SV=3	46	4	15.2
P10599	Thioredoxin OS=Homo sapiens GN=TXN PE=1 SV=3	46	4	11.7
Q9BRT2	Ubiquinol-cytochrome-c reductase complex assembly factor 2 OS=Homo sapiens GN=UQCC2 PE=1 SV=1	46	4	14.9
P62244	40S ribosomal protein S15a OS=Homo sapiens GN=RPS15A PE=1 SV=2	46	2	14.8
Q01082	Spectrin beta chain, non-erythrocytic 1 OS=Homo sapiens GN=SPTBN1 PE=1 SV=2	45	78	274.4
O15031	Plexin-B2 OS=Homo sapiens GN=PLXNB2 PE=1 SV=3	45	60	205
P15144	Aminopeptidase N OS=Homo sapiens GN=ANPEP PE=1 SV=4	45	48	109.5
O00754	Lysosomal alpha-mannosidase OS=Homo sapiens GN=MAN2B1 PE=1 SV=3	45	30	113.7
O95831	Apoptosis-inducing factor 1, mitochondrial OS=Homo sapiens GN=AIFM1 PE=1 SV=1	45	23	66.9
O15254	Peroxisomal acyl-coenzyme A oxidase 3 OS=Homo sapiens GN=ACOX3 PE=1 SV=2	45	22	77.6
O43615	Mitochondrial import inner membrane translocase subunit TIM44 OS=Homo sapiens GN=TIMM44 PE=1 SV=2	45	20	51.3
P30837	Aldehyde dehydrogenase X, mitochondrial OS=Homo sapiens GN=ALDH1B1 PE=1 SV=3	45	17	57.2
Q9UHG3	Prenylcysteine oxidase 1 OS=Homo sapiens GN=PCYOX1 PE=1 SV=3	45	15	56.6
P18031	Tyrosine-protein phosphatase non-receptor type 1 OS=Homo sapiens GN=PTPN1 PE=1 SV=1	45	15	49.9
O15143	Actin-related protein 2/3 complex subunit 1B OS=Homo sapiens GN=ARPC1B PE=1 SV=3	45	13	40.9
B1AK88	Capping protein (Actin filament) muscle Z-line, beta, isoform CRA_d OS=Homo sapiens GN=CAPZB PE=1 SV=1	45	13	33.8
P07195	L-lactate dehydrogenase B chain OS=Homo sapiens GN=LDHB PE=1 SV=2	45	13	36.6
A8MXV4	Nucleoside diphosphate-linked moiety X motif 19 OS=Homo sapiens GN=NUDT19 PE=1 SV=1	45	13	42.2
Q8NBX0	Saccharopine dehydrogenase-like oxidoreductase OS=Homo sapiens GN=SCCPDH PE=1 SV=1	45	13	47.1
P43897	Elongation factor Ts, mitochondrial OS=Homo sapiens GN=TSFM PE=1 SV=2	45	12	35.4
Q9BWD1	Acetyl-CoA acetyltransferase, cytosolic OS=Homo sapiens GN=ACAT2 PE=1 SV=2	45	10	41.3
Q9UIJ7	GTP:AMP phosphotransferase AK3, mitochondrial OS=Homo sapiens GN=AK3 PE=1 SV=4	45	10	25.6

Q8TAE8	Growth arrest and DNA damage-inducible proteins-interacting protein 1 OS=Homo sapiens GN=GADD45GIP1 PE=1 SV=1	45	9	25.4
Q92597	Protein NDRG1 OS=Homo sapiens GN=NDRG1 PE=1 SV=1	45	9	42.8
H7BY58	Protein-L-isoaspartate O-methyltransferase OS=Homo sapiens GN=PCMT1 PE=1 SV=1	45	9	30.3
P51809	Vesicle-associated membrane protein 7 OS=Homo sapiens GN=VAMP7 PE=1 SV=3	45	9	24.9
Q7Z2W9	39S ribosomal protein L21, mitochondrial OS=Homo sapiens GN=MRPL21 PE=1 SV=2	45	8	22.8
P23919	Thymidylate kinase OS=Homo sapiens GN=DTYMK PE=1 SV=4	45	8	23.8
P67936	Tropomyosin alpha-4 chain OS=Homo sapiens GN=TPM4 PE=1 SV=3	45	7	28.5
Q16611	Bcl-2 homologous antagonist/killer OS=Homo sapiens GN=BAK1 PE=1 SV=1	45	6	23.4
Q9H3Z4	DnaJ homolog subfamily C member 5 OS=Homo sapiens GN=DNAJC5 PE=1 SV=1	45	6	22.1
O95716	Ras-related protein Rab-3D OS=Homo sapiens GN=RAB3D PE=1 SV=1	45	6	24.3
Q04837	Single-stranded DNA-binding protein, mitochondrial OS=Homo sapiens GN=SSBP1 PE=1 SV=1	45	6	17.2
P62979	Ubiquitin-40S ribosomal protein S27a OS=Homo sapiens GN=RPS27A PE=1 SV=2	45	6	18
P18859	ATP synthase-coupling factor 6, mitochondrial OS=Homo sapiens GN=ATP5J PE=1 SV=1	45	5	12.6
P61006	Ras-related protein Rab-8A OS=Homo sapiens GN=RAB8A PE=1 SV=1	45	5	23.7
Q9BUF5	Tubulin beta-6 chain OS=Homo sapiens GN=TUBB6 PE=1 SV=1	45	4	49.8
Q8N8J7	Uncharacterized protein C4orf32 OS=Homo sapiens GN=C4orf32 PE=2 SV=2	45	4	14.6
Q7Z4R8	UPF0669 protein C6orf120 OS=Homo sapiens GN=C6orf120 PE=1 SV=1	45	3	20.8
P11215	Integrin alpha-M OS=Homo sapiens GN=ITGAM PE=1 SV=2	44	44	127.1
P16615	Sarcoplasmic/endoplasmic reticulum calcium ATPase 2 OS=Homo sapiens GN=ATP2A2 PE=1 SV=1	44	34	114.7
O15228	Dihydroxyacetone phosphate acyltransferase OS=Homo sapiens GN=GNPAT PE=1 SV=1	44	25	77.1
P04839	Cytochrome b-245 heavy chain OS=Homo sapiens GN=CYBB PE=1 SV=2	44	22	65.3
Q53F23	HCG14925, isoform CRA_a (Fragment) OS=Homo sapiens GN=TBXAS1 PE=1 SV=1	44	18	60.6
Q9HCC0	Methylcrotonoyl-CoA carboxylase beta chain, mitochondrial OS=Homo sapiens GN=MCCC2 PE=1 SV=1	44	18	61.3
P36551	Oxygen-dependent coproporphyrinogen-III oxidase, mitochondrial OS=Homo sapiens GN=CPOX PE=1 SV=3	44	16	50.1
A0A087WZN1	Isocitrate dehydrogenase [NAD] subunit, mitochondrial OS=Homo sapiens GN=IDH3B PE=1 SV=1	44	15	42.4
P82650	28S ribosomal protein S22, mitochondrial OS=Homo sapiens GN=MRPS22 PE=1 SV=1	44	14	41.3
O43772	Mitochondrial carnitine/acylcarnitine carrier protein OS=Homo sapiens GN=SLC25A20 PE=1 SV=1	44	14	32.9
Q02878	60S ribosomal protein L6 OS=Homo sapiens GN=RPL6 PE=1 SV=3	44	13	32.7

P62424	60S ribosomal protein L7a OS=Homo sapiens GN=RPL7A PE=1 SV=2	44	12	30
Q9BXW7	Cat eye syndrome critical region protein 5 OS=Homo sapiens GN=CECR5 PE=1 SV=1	44	12	46.3
Q9Y394	Dehydrogenase/reductase SDR family member 7 OS=Homo sapiens GN=DHRS7 PE=1 SV=1	44	12	38.3
Q15080	Neutrophil cytosol factor 4 OS=Homo sapiens GN=NCF4 PE=1 SV=2	44	12	39
O60664	Perilipin-3 OS=Homo sapiens GN=PLIN3 PE=1 SV=3	44	12	47
Q16698	2,4-dienoyl-CoA reductase, mitochondrial OS=Homo sapiens GN=DECR1 PE=1 SV=1	44	11	36
A6NC48	ADP-ribosyl cyclase/cyclic ADP-ribose hydrolase 2 OS=Homo sapiens GN=BST1 PE=1 SV=1	44	11	37.5
Q9Y624	Junctional adhesion molecule A OS=Homo sapiens GN=F11R PE=1 SV=1	44	11	32.6
Q03405	Urokinase plasminogen activator surface receptor OS=Homo sapiens GN=PLAUR PE=1 SV=1	44	11	37
P31937	3-hydroxyisobutyrate dehydrogenase, mitochondrial OS=Homo sapiens GN=HIBADH PE=1 SV=2	44	10	35.3
Q13011	Delta(3,5)-Delta(2,4)-dienoyl-CoA isomerase, mitochondrial OS=Homo sapiens GN=ECH1 PE=1 SV=2	44	10	35.8
P10301	Ras-related protein R-Ras OS=Homo sapiens GN=RRAS PE=1 SV=1	44	8	23.5
Q9BVK6	Transmembrane emp24 domain-containing protein 9 OS=Homo sapiens GN=TMED9 PE=1 SV=2	44	8	27.3
O75396	Vesicle-trafficking protein SEC22b OS=Homo sapiens GN=SEC22B PE=1 SV=4	44	8	24.6
Q9Y3A6	Transmembrane emp24 domain-containing protein 5 OS=Homo sapiens GN=TMED5 PE=1 SV=1	44	7	26
P09211	Glutathione S-transferase P OS=Homo sapiens GN=GSTP1 PE=1 SV=2	44	6	23.3
P24158	Myeloblastin OS=Homo sapiens GN=PRTN3 PE=1 SV=3	44	6	27.8
Q99470	Stromal cell-derived factor 2 OS=Homo sapiens GN=SDF2 PE=1 SV=2	44	6	23
Q9NZ45	CDGSH iron-sulfur domain-containing protein 1 OS=Homo sapiens GN=CISD1 PE=1 SV=1	44	4	12.2
Q9BY43	Charged multivesicular body protein 4a OS=Homo sapiens GN=CHMP4A PE=1 SV=3	44	2	25.1
O60262	Guanine nucleotide-binding protein G(I)/G(S)/G(O) subunit gamma-7 OS=Homo sapiens GN=GNG7 PE=1 SV=1	44	2	7.5
Q13423	NAD(P) transhydrogenase, mitochondrial OS=Homo sapiens GN=NNT PE=1 SV=3	43	45	113.8
Q9BSJ8	Extended synaptotagmin-1 OS=Homo sapiens GN=ESYT1 PE=1 SV=1	43	38	122.8
Q9Y4W6	AFG3-like protein 2 OS=Homo sapiens GN=AFG3L2 PE=1 SV=2	43	34	88.5
Q9H845	Acyl-CoA dehydrogenase family member 9, mitochondrial OS=Homo sapiens GN=ACAD9 PE=1 SV=1	43	27	68.7
Q6PI48	Aspartate--tRNA ligase, mitochondrial OS=Homo sapiens GN=DARS2 PE=1 SV=1	43	22	73.5
P22307	Non-specific lipid-transfer protein OS=Homo sapiens GN=SCP2 PE=1 SV=2	43	22	59
P10515	Dihydrolipoyllysine-residue acetyltransferase component of pyruvate dehydrogenase complex, mitochondrial OS=Homo sapiens GN=DLAT PE=1 SV=3	43	19	69

Q16822	Phosphoenolpyruvate carboxykinase [GTP], mitochondrial OS=Homo sapiens GN=PCK2 PE=1 SV=3	43	19	70.7
Q9NVH1	DnaJ homolog subfamily C member 11 OS=Homo sapiens GN=DNAJC11 PE=1 SV=2	43	18	63.2
Q96JJ7	Protein disulfide-isomerase TMX3 OS=Homo sapiens GN=TMX3 PE=1 SV=2	43	17	51.8
Q00013	55 kDa erythrocyte membrane protein OS=Homo sapiens GN=MPP1 PE=1 SV=2	43	15	52.3
Q9H9J2	39S ribosomal protein L44, mitochondrial OS=Homo sapiens GN=MRPL44 PE=1 SV=1	43	13	37.5
Q8TBP6	Solute carrier family 25 member 40 OS=Homo sapiens GN=SLC25A40 PE=1 SV=1	43	13	38.1
J3KPT4	TraB domain-containing protein OS=Homo sapiens GN=TRABD PE=1 SV=1	43	11	42.7
Q9NRX2	39S ribosomal protein L17, mitochondrial OS=Homo sapiens GN=MRPL17 PE=1 SV=1	43	10	20
Q9H2W6	39S ribosomal protein L46, mitochondrial OS=Homo sapiens GN=MRPL46 PE=1 SV=1	43	10	31.7
Q07021	Complement component 1 Q subcomponent-binding protein, mitochondrial OS=Homo sapiens GN=C1QBP PE=1 SV=1	43	9	31.3
P13073	Cytochrome c oxidase subunit 4 isoform 1, mitochondrial OS=Homo sapiens GN=COX4I1 PE=1 SV=1	43	9	19.6
Q15006	ER membrane protein complex subunit 2 OS=Homo sapiens GN=EMC2 PE=1 SV=1	43	9	34.8
P40121	Macrophage-capping protein OS=Homo sapiens GN=CAPG PE=1 SV=2	43	9	38.5
P62081	40S ribosomal protein S7 OS=Homo sapiens GN=RPS7 PE=1 SV=1	43	6	22.1
O95168	NADH dehydrogenase [ubiquinone] 1 beta subcomplex subunit 4 OS=Homo sapiens GN=NDUFB4 PE=1 SV=3	43	6	15.2
P31946	14-3-3 protein beta/alpha OS=Homo sapiens GN=YWHAB PE=1 SV=3	43	5	28.1
Q8N4L2	Type 2 phosphatidylinositol 4,5-bisphosphate 4-phosphatase OS=Homo sapiens GN=TMEM55A PE=1 SV=1	43	5	28.1
B4DLN1	cDNA FLJ60124, highly similar to Mitochondrial dicarboxylate carrier OS=Homo sapiens PE=2 SV=1	43	4	48.1
P00167	Cytochrome b5 OS=Homo sapiens GN=CYB5A PE=1 SV=2	43	4	15.3
Q16864	V-type proton ATPase subunit F OS=Homo sapiens GN=ATP6V1F PE=1 SV=2	43	4	13.4
O00453	Leukocyte-specific transcript 1 protein OS=Homo sapiens GN=LST1 PE=1 SV=2	43	3	10.8
Q00325	Phosphate carrier protein, mitochondrial OS=Homo sapiens GN=SLC25A3 PE=1 SV=2	43	3	40.1
P05556	Integrin beta-1 OS=Homo sapiens GN=ITGB1 PE=1 SV=2	42	33	88.4
P50416	Carnitine O-palmitoyltransferase 1, liver isoform OS=Homo sapiens GN=CPT1A PE=1 SV=2	42	31	88.3
P54886	Delta-1-pyrroline-5-carboxylate synthase OS=Homo sapiens GN=ALDH18A1 PE=1 SV=2	42	30	87.2
O95140	Mitofusin-2 OS=Homo sapiens GN=MFN2 PE=1 SV=3	42	22	86.3
Q96RQ3	Methylcrotonoyl-CoA carboxylase subunit alpha, mitochondrial OS=Homo sapiens GN=MCCC1 PE=1 SV=3	42	20	80.4
P20700	Lamin-B1 OS=Homo sapiens GN=LMNB1 PE=1 SV=2	42	19	66.4

Q8IXI2	Mitochondrial Rho GTPase 1 OS=Homo sapiens GN=RHOT1 PE=1 SV=2	42	17	70.7
A1L0T0	Acetolactate synthase-like protein OS=Homo sapiens GN=ILVBL PE=1 SV=2	42	16	67.8
Q13510	Acid ceramidase OS=Homo sapiens GN=ASAH1 PE=1 SV=5	42	16	44.6
Q96DV4	39S ribosomal protein L38, mitochondrial OS=Homo sapiens GN=MRPL38 PE=1 SV=2	42	15	44.6
P22695	Cytochrome b-c1 complex subunit 2, mitochondrial OS=Homo sapiens GN=UQCRC2 PE=1 SV=3	42	15	48.4
P43490	Nicotinamide phosphoribosyltransferase OS=Homo sapiens GN=NAMPT PE=1 SV=1	42	15	55.5
Q9BXK5	Bcl-2-like protein 13 OS=Homo sapiens GN=BCL2L13 PE=1 SV=1	42	13	52.7
Q96AG4	Leucine-rich repeat-containing protein 59 OS=Homo sapiens GN=LRRC59 PE=1 SV=1	42	13	34.9
Q9Y265	RuvB-like 1 OS=Homo sapiens GN=RUVBL1 PE=1 SV=1	42	13	50.2
O96011	Peroxisomal membrane protein 11B OS=Homo sapiens GN=PEX11B PE=1 SV=1	42	11	28.4
J3KQY1	39S ribosomal protein L22, mitochondrial OS=Homo sapiens GN=MRPL22 PE=1 SV=1	42	10	26.5
P62917	60S ribosomal protein L8 OS=Homo sapiens GN=RPL8 PE=1 SV=2	42	10	28
Q9NTX5	Ethylmalonyl-CoA decarboxylase OS=Homo sapiens GN=ECHDC1 PE=1 SV=2	42	10	33.7
Q13501	Sequestosome-1 OS=Homo sapiens GN=SQSTM1 PE=1 SV=1	42	10	47.7
P20645	Cation-dependent mannose-6-phosphate receptor OS=Homo sapiens GN=M6PR PE=1 SV=1	42	9	31
Q96LJ7	Dehydrogenase/reductase SDR family member 1 OS=Homo sapiens GN=DHRS1 PE=1 SV=1	42	9	33.9
Q14696	LDLR chaperone MESD OS=Homo sapiens GN=MESDC2 PE=1 SV=2	42	9	26.1
Q9H061	Transmembrane protein 126A OS=Homo sapiens GN=TMEM126A PE=1 SV=1	42	8	21.5
Q9NQR4	Omega-amidase NIT2 OS=Homo sapiens GN=NIT2 PE=1 SV=1	42	7	30.6
Q9HBH0	Rho-related GTP-binding protein RhoF OS=Homo sapiens GN=RHOF PE=1 SV=1	42	7	23.6
P60981	Destrin OS=Homo sapiens GN=DSTN PE=1 SV=3	42	6	18.5
P24534	Elongation factor 1-beta OS=Homo sapiens GN=EEF1B2 PE=1 SV=3	42	6	24.7
Q5RI15	Cytochrome c oxidase protein 20 homolog OS=Homo sapiens GN=COX20 PE=1 SV=2	42	4	13.3
O60234	Glia maturation factor gamma OS=Homo sapiens GN=GMFG PE=1 SV=1	42	4	16.8
Q92930	Ras-related protein Rab-8B OS=Homo sapiens GN=RAB8B PE=1 SV=2	42	4	23.6
P68036	Ubiquitin-conjugating enzyme E2 L3 OS=Homo sapiens GN=UBE2L3 PE=1 SV=1	42	4	17.9
P61513	60S ribosomal protein L37a OS=Homo sapiens GN=RPL37A PE=1 SV=2	42	3	10.3
O95167	NADH dehydrogenase [ubiquinone] 1 alpha subcomplex subunit 3 OS=Homo sapiens GN=NDUFA3 PE=1 SV=1	42	3	9.3

Q86TS9	39S ribosomal protein L52, mitochondrial OS=Homo sapiens GN=MRPL52 PE=1 SV=2	42	2	13.7
Q9Y6C9	Mitochondrial carrier homolog 2 OS=Homo sapiens GN=MTCH2 PE=1 SV=1	42	2	33.3
P11717	Cation-independent mannose-6-phosphate receptor OS=Homo sapiens GN=IGF2R PE=1 SV=3	41	77	274.2
H7BXI1	Extended synaptotagmin-2 (Fragment) OS=Homo sapiens GN=ESYT2 PE=1 SV=1	41	28	97.9
P50570	Dynamin-2 OS=Homo sapiens GN=DNM2 PE=1 SV=2	41	27	98
P14780	Matrix metalloproteinase-9 OS=Homo sapiens GN=MMP9 PE=1 SV=3	41	22	78.4
O00186	Syntaxin-binding protein 3 OS=Homo sapiens GN=STXBP3 PE=1 SV=2	41	22	67.7
P14868	Aspartate--tRNA ligase, cytoplasmic OS=Homo sapiens GN=DARS PE=1 SV=2	41	18	57.1
P50991	T-complex protein 1 subunit delta OS=Homo sapiens GN=CCT4 PE=1 SV=4	41	17	57.9
P14317	Hematopoietic lineage cell-specific protein OS=Homo sapiens GN=HCLS1 PE=1 SV=3	41	16	54
Q5T4U5	Acyl-Coenzyme A dehydrogenase, C-4 to C-12 straight chain, isoform CRA_a OS=Homo sapiens GN=ACADM PE=1 SV=1	41	14	50.2
A0A0A0MTJ9	Neutral cholesterol ester hydrolase 1 OS=Homo sapiens GN=NCEH1 PE=1 SV=1	41	14	49.9
Q9BWS9	Chitinase domain-containing protein 1 OS=Homo sapiens GN=CHID1 PE=1 SV=1	41	13	44.9
Q8IXI1	Mitochondrial Rho GTPase 2 OS=Homo sapiens GN=RHOT2 PE=1 SV=2	41	13	68.1
P08574	Cytochrome c1, heme protein, mitochondrial OS=Homo sapiens GN=CYC1 PE=1 SV=3	41	12	35.4
P60842	Eukaryotic initiation factor 4A-I OS=Homo sapiens GN=EIF4A1 PE=1 SV=1	41	12	46.1
P42768	Wiskott-Aldrich syndrome protein OS=Homo sapiens GN=WAS PE=1 SV=4	41	12	52.9
Q9Y2S7	Polymerase delta-interacting protein 2 OS=Homo sapiens GN=POLDIP2 PE=1 SV=1	41	11	42
Q12846	Syntaxin-4 OS=Homo sapiens GN=STX4 PE=1 SV=2	41	11	34.2
Q92947	Glutaryl-CoA dehydrogenase, mitochondrial OS=Homo sapiens GN=GCDH PE=1 SV=1	41	10	48.1
P62993	Growth factor receptor-bound protein 2 OS=Homo sapiens GN=GRB2 PE=1 SV=1	41	10	25.2
O14828	Secretory carrier-associated membrane protein 3 OS=Homo sapiens GN=SCAMP3 PE=1 SV=3	41	10	38.3
Q9UDX5	Mitochondrial fission process protein 1 OS=Homo sapiens GN=MTFP1 PE=1 SV=1	41	8	18
P62277	40S ribosomal protein S13 OS=Homo sapiens GN=RPS13 PE=1 SV=2	41	7	17.2
O95169	NADH dehydrogenase [ubiquinone] 1 beta subcomplex subunit 8, mitochondrial OS=Homo sapiens GN=NDUFB8 PE=1 SV=1	41	7	21.8
O14818	Proteasome subunit alpha type-7 OS=Homo sapiens GN=PSMA7 PE=1 SV=1	41	7	27.9
Q9Y5U8	Mitochondrial pyruvate carrier 1 OS=Homo sapiens GN=MPC1 PE=1 SV=1	41	6	12.3

P51151	Ras-related protein Rab-9A OS=Homo sapiens GN=RAB9A PE=1 SV=1	41	6	22.8
P32969	60S ribosomal protein L9 OS=Homo sapiens GN=RPL9 PE=1 SV=1	41	5	21.9
P30273	High affinity immunoglobulin epsilon receptor subunit gamma OS=Homo sapiens GN=FCER1G PE=1 SV=1	41	4	9.7
Q9NPJ3	Acyl-coenzyme A thioesterase 13 OS=Homo sapiens GN=ACOT13 PE=1 SV=1	41	3	15
P18085	ADP-ribosylation factor 4 OS=Homo sapiens GN=ARF4 PE=1 SV=3	41	3	20.5
Q9HB66	Alternative protein MKKS OS=Homo sapiens GN=MKKS PE=1 SV=1	41	2	7.3
P15954	Cytochrome c oxidase subunit 7C, mitochondrial OS=Homo sapiens GN=COX7C PE=1 SV=1	41	2	7.2
Q8WUD1	Ras-related protein Rab-2B OS=Homo sapiens GN=RAB2B PE=1 SV=1	41	2	24.2
Q92835	Phosphatidylinositol 3,4,5-trisphosphate 5-phosphatase 1 OS=Homo sapiens GN=INPP5D PE=1 SV=2	40	30	133.2
O95782	AP-2 complex subunit alpha-1 OS=Homo sapiens GN=AP2A1 PE=1 SV=3	40	21	107.5
Q96A33	Coiled-coil domain-containing protein 47 OS=Homo sapiens GN=CCDC47 PE=1 SV=1	40	18	55.8
P36578	60S ribosomal protein L4 OS=Homo sapiens GN=RPL4 PE=1 SV=5	40	17	47.7
Q9UI12	V-type proton ATPase subunit H OS=Homo sapiens GN=ATP6V1H PE=1 SV=1	40	15	55.8
Q3ZCQ8	Mitochondrial import inner membrane translocase subunit TIM50 OS=Homo sapiens GN=TIMM50 PE=1 SV=2	40	14	39.6
Q92665	28S ribosomal protein S31, mitochondrial OS=Homo sapiens GN=MRPS31 PE=1 SV=3	40	13	45.3
P50213	Isocitrate dehydrogenase [NAD] subunit alpha, mitochondrial OS=Homo sapiens GN=IDH3A PE=1 SV=1	40	13	39.6
F5H5P2	Uncharacterized protein OS=Homo sapiens PE=4 SV=1	40	13	54.2
P30679	Guanine nucleotide-binding protein subunit alpha-15 OS=Homo sapiens GN=GNA15 PE=1 SV=2	40	12	43.5
Q9NNW7	Thioredoxin reductase 2, mitochondrial OS=Homo sapiens GN=TXNRD2 PE=1 SV=3	40	12	56.5
Q5HYK3	2-methoxy-6-polyprenyl-1,4-benzoquinol methylase, mitochondrial OS=Homo sapiens GN=COQ5 PE=1 SV=2	40	10	37.1
P25774	Cathepsin S OS=Homo sapiens GN=CTSS PE=1 SV=3	40	10	37.5
P51553	Isocitrate dehydrogenase [NAD] subunit gamma, mitochondrial OS=Homo sapiens GN=IDH3G PE=1 SV=1	40	9	42.8
Q96BS2	Calcineurin B homologous protein 3 OS=Homo sapiens GN=TESC PE=1 SV=3	40	8	24.7
P06748	Nucleophosmin OS=Homo sapiens GN=NPM1 PE=1 SV=2	40	8	32.6
Q8TC12	Retinol dehydrogenase 11 OS=Homo sapiens GN=RDH11 PE=1 SV=2	40	8	35.4
P28066	Proteasome subunit alpha type-5 OS=Homo sapiens GN=PSMA5 PE=1 SV=3	40	7	26.4
P18077	60S ribosomal protein L35a OS=Homo sapiens GN=RPL35A PE=1 SV=2	40	6	12.5
Q9NVT9	Armadillo repeat-containing protein 1 OS=Homo sapiens GN=ARMC1 PE=1 SV=1	40	6	31.3

O95816	BAG family molecular chaperone regulator 2 OS=Homo sapiens GN=BAG2 PE=1 SV=1	40	6	23.8
P49721	Proteasome subunit beta type-2 OS=Homo sapiens GN=PSMB2 PE=1 SV=1	40	6	22.8
P82932	28S ribosomal protein S6, mitochondrial OS=Homo sapiens GN=MRPS6 PE=1 SV=3	40	5	14.2
P25398	40S ribosomal protein S12 OS=Homo sapiens GN=RPS12 PE=1 SV=3	40	5	14.5
P56556	NADH dehydrogenase [ubiquinone] 1 alpha subcomplex subunit 6 OS=Homo sapiens GN=NDUFA6 PE=1 SV=3	40	5	17.9
Q15836	Vesicle-associated membrane protein 3 OS=Homo sapiens GN=VAMP3 PE=1 SV=3	40	4	11.3
Q13303	Voltage-gated potassium channel subunit beta-2 OS=Homo sapiens GN=KCNAB2 PE=1 SV=2	40	4	41
Q9NSE4	Isoleucine--tRNA ligase, mitochondrial OS=Homo sapiens GN=IARS2 PE=1 SV=2	39	34	113.7
P33121	Long-chain-fatty-acid--CoA ligase 1 OS=Homo sapiens GN=ACSL1 PE=1 SV=1	39	26	77.9
P33897	ATP-binding cassette sub-family D member 1 OS=Homo sapiens GN=ABCD1 PE=1 SV=2	39	22	82.9
P05362	Intercellular adhesion molecule 1 OS=Homo sapiens GN=ICAM1 PE=1 SV=2	39	19	57.8
Q9H3N1	Thioredoxin-related transmembrane protein 1 OS=Homo sapiens GN=TMX1 PE=1 SV=1	39	18	31.8
J3KN66	Torsin-1A-interacting protein 1 OS=Homo sapiens GN=TOR1AIP1 PE=1 SV=1	39	17	67.8
Q9Y4P3	Transducin beta-like protein 2 OS=Homo sapiens GN=TBL2 PE=1 SV=1	39	17	49.8
Q6UW02	Cytochrome P450 20A1 OS=Homo sapiens GN=CYP20A1 PE=1 SV=1	39	13	52.4
P11182	Lipoamide acyltransferase component of branched-chain alpha-keto acid dehydrogenase complex, mitochondrial OS=Homo sapiens GN=DBT PE=1 SV=3	39	13	53.5
P51398	28S ribosomal protein S29, mitochondrial OS=Homo sapiens GN=DAP3 PE=1 SV=1	39	12	45.5
E9PI68	Signal peptidase complex subunit 2 OS=Homo sapiens GN=SPCS2 PE=1 SV=1	39	11	28.5
F5GYQ1	V-type proton ATPase subunit d 1 OS=Homo sapiens GN=ATP6V0D1 PE=1 SV=1	39	10	44.6
P61247	40S ribosomal protein S3a OS=Homo sapiens GN=RPS3A PE=1 SV=2	39	9	29.9
Q8NBU5	ATPase family AAA domain-containing protein 1 OS=Homo sapiens GN=ATAD1 PE=1 SV=1	39	9	40.7
P49755	Transmembrane emp24 domain-containing protein 10 OS=Homo sapiens GN=TMED10 PE=1 SV=2	39	9	25
P27635	60S ribosomal protein L10 OS=Homo sapiens GN=RPL10 PE=1 SV=4	39	8	24.6
Q5QGZ9	C-type lectin domain family 12 member A OS=Homo sapiens GN=CLEC12A PE=1 SV=3	39	8	30.7
Q9UBQ7	Glyoxylate reductase/hydroxypyruvate reductase OS=Homo sapiens GN=GRHPR PE=1 SV=1	39	8	35.6
P08246	Neutrophil elastase OS=Homo sapiens GN=ELANE PE=1 SV=1	39	8	28.5
P00491	Purine nucleoside phosphorylase OS=Homo sapiens GN=PNP PE=1 SV=2	39	8	32.1
Q9H444	Charged multivesicular body protein 4b OS=Homo sapiens GN=CHMP4B PE=1 SV=1	39	7	24.9

Q9Y696	Chloride intracellular channel protein 4 OS=Homo sapiens GN=CLIC4 PE=1 SV=4	39	7	28.8
O94766	Galactosylgalactosylxylosylprotein 3-beta-glucuronosyltransferase 3 OS=Homo sapiens GN=B3GAT3 PE=1 SV=2	39	7	37.1
O75431	Metaxin-2 OS=Homo sapiens GN=MTX2 PE=1 SV=1	39	7	29.7
P67812	Signal peptidase complex catalytic subunit SEC11A OS=Homo sapiens GN=SEC11A PE=1 SV=1	39	7	20.6
Q56VL3	OCIA domain-containing protein 2 OS=Homo sapiens GN=OCIAD2 PE=1 SV=1	39	6	16.9
Q6P1L8	39S ribosomal protein L14, mitochondrial OS=Homo sapiens GN=MRPL14 PE=1 SV=1	39	5	15.9
E5RJR5	S-phase kinase-associated protein 1 OS=Homo sapiens GN=SKP1 PE=1 SV=1	39	5	18.7
P09669	Cytochrome c oxidase subunit 6C OS=Homo sapiens GN=COX6C PE=1 SV=2	39	4	8.8
P35268	60S ribosomal protein L22 OS=Homo sapiens GN=RPL22 PE=1 SV=2	39	3	14.8
O75348	V-type proton ATPase subunit G 1 OS=Homo sapiens GN=ATP6V1G1 PE=1 SV=3	39	3	13.7
Q93033	Immunoglobulin superfamily member 2 OS=Homo sapiens GN=CD101 PE=1 SV=2	38	29	115
Q96RP9	Elongation factor G, mitochondrial OS=Homo sapiens GN=GFM1 PE=1 SV=2	38	27	83.4
H3BN02	Integrin alpha-X OS=Homo sapiens GN=ITGAX PE=1 SV=1	38	26	128.5
P41250	Glycine--tRNA ligase OS=Homo sapiens GN=GARS PE=1 SV=3	38	20	83.1
Q9BW92	Threonine--tRNA ligase, mitochondrial OS=Homo sapiens GN=TARS2 PE=1 SV=1	38	18	81
Q96TA1	Niban-like protein 1 OS=Homo sapiens GN=FAM129B PE=1 SV=3	38	17	84.1
Q10471	Polypeptide N-acetylgalactosaminyltransferase 2 OS=Homo sapiens GN=GALNT2 PE=1 SV=1	38	17	64.7
Q9HC21	Mitochondrial thiamine pyrophosphate carrier OS=Homo sapiens GN=SLC25A19 PE=1 SV=1	38	16	35.5
A0A140T9T7	Antigen peptide transporter 1 OS=Homo sapiens GN=TAP1 PE=1 SV=1	38	15	87.1
Q13884	Beta-1-syntrophin OS=Homo sapiens GN=SNTB1 PE=1 SV=3	38	15	58
P39023	60S ribosomal protein L3 OS=Homo sapiens GN=RPL3 PE=1 SV=2	38	14	46.1
Q9BR76	Coronin-1B OS=Homo sapiens GN=CORO1B PE=1 SV=1	38	14	54.2
P82933	28S ribosomal protein S9, mitochondrial OS=Homo sapiens GN=MRPS9 PE=1 SV=2	38	12	45.8
Q8IYU8	Calcium uptake protein 2, mitochondrial OS=Homo sapiens GN=MICU2 PE=1 SV=2	38	12	49.6
Q70UQ0	Inhibitor of nuclear factor kappa-B kinase-interacting protein OS=Homo sapiens GN=IKBIP PE=1 SV=1	38	12	39.3
Q687X5	Metalloreductase STEAP4 OS=Homo sapiens GN=STEAP4 PE=1 SV=1	38	12	51.9
Q96NB2	Sideroflexin-2 OS=Homo sapiens GN=SFXN2 PE=1 SV=2	38	11	36.2
J3QLS3	28S ribosomal protein S7, mitochondrial OS=Homo sapiens GN=MRPS7 PE=1 SV=1	38	10	31.7

P43353	Aldehyde dehydrogenase family 3 member B1 OS=Homo sapiens GN=ALDH3B1 PE=1 SV=1	38	10	51.8
E7EMB3	Calmodulin OS=Homo sapiens GN=CALM2 PE=1 SV=1	38	10	21.7
Q96C19	EF-hand domain-containing protein D2 OS=Homo sapiens GN=EFHD2 PE=1 SV=1	38	10	26.7
Q9HBH5	Retinol dehydrogenase 14 OS=Homo sapiens GN=RDH14 PE=1 SV=1	38	10	36.8
B4DUT8	Calponin OS=Homo sapiens GN=CNN2 PE=1 SV=1	38	9	35.9
P46782	40S ribosomal protein S5 OS=Homo sapiens GN=RPS5 PE=1 SV=4	38	8	22.9
O95298	NADH dehydrogenase [ubiquinone] 1 subunit C2 OS=Homo sapiens GN=NDUFC2 PE=1 SV=1	38	8	14.2
O43819	Protein SCO2 homolog, mitochondrial OS=Homo sapiens GN=SCO2 PE=1 SV=3	38	8	29.8
Q7Z7H5	Transmembrane emp24 domain-containing protein 4 OS=Homo sapiens GN=TMED4 PE=1 SV=1	38	7	25.9
Q9NQ50	39S ribosomal protein L40, mitochondrial OS=Homo sapiens GN=MRPL40 PE=1 SV=1	38	6	24.5
A0A024RA52	Proteasome subunit alpha type OS=Homo sapiens GN=PSMA2 PE=1 SV=1	38	6	25.9
P61353	60S ribosomal protein L27 OS=Homo sapiens GN=RPL27 PE=1 SV=2	38	5	15.8
P20340	Ras-related protein Rab-6A OS=Homo sapiens GN=RAB6A PE=1 SV=3	38	5	23.6
Q99643	Succinate dehydrogenase cytochrome b560 subunit, mitochondrial OS=Homo sapiens GN=SDHC PE=1 SV=1	38	4	18.6
K7ELC2	40S ribosomal protein S15 OS=Homo sapiens GN=RPS15 PE=1 SV=1	38	3	17.7
P30049	ATP synthase subunit delta, mitochondrial OS=Homo sapiens GN=ATP5D PE=1 SV=2	38	3	17.5
P01111	GTPase NRas OS=Homo sapiens GN=NRAS PE=1 SV=1	38	3	21.2
Q9UDW1	Cytochrome b-c1 complex subunit 9 OS=Homo sapiens GN=UQCR10 PE=1 SV=3	38	2	7.3
Q6UW78	Ubiquinol-cytochrome-c reductase complex assembly factor 3 OS=Homo sapiens GN=UQCC3 PE=1 SV=2	38	2	10.1
Q9HD20	Manganese-transporting ATPase 13A1 OS=Homo sapiens GN=ATP13A1 PE=1 SV=2	37	36	132.9
P23634	Plasma membrane calcium-transporting ATPase 4 OS=Homo sapiens GN=ATP2B4 PE=1 SV=2	37	27	137.8
O60603	Toll-like receptor 2 OS=Homo sapiens GN=TLR2 PE=1 SV=1	37	25	89.8
Q9NRK6	ATP-binding cassette sub-family B member 10, mitochondrial OS=Homo sapiens GN=ABCB10 PE=1 SV=2	37	23	79.1
P16435	NADPH--cytochrome P450 reductase OS=Homo sapiens GN=POR PE=1 SV=2	37	23	76.6
P20020	Plasma membrane calcium-transporting ATPase 1 OS=Homo sapiens GN=ATP2B1 PE=1 SV=3	37	20	138.7
Q15046	Lysine--tRNA ligase OS=Homo sapiens GN=KARS PE=1 SV=3	37	18	68
Q10472	Polypeptide N-acetylgalactosaminyltransferase 1 OS=Homo sapiens GN=GALNT1 PE=1 SV=1	37	18	64.2
P17987	T-complex protein 1 subunit alpha OS=Homo sapiens GN=TCP1 PE=1 SV=1	37	18	60.3

Q9UH99	SUN domain-containing protein 2 OS=Homo sapiens GN=SUN2 PE=1 SV=3	37	17	80.3
Q9ULV4	Coronin-1C OS=Homo sapiens GN=CORO1C PE=1 SV=1	37	16	53.2
Q07065	Cytoskeleton-associated protein 4 OS=Homo sapiens GN=CKAP4 PE=1 SV=2	37	16	66
P35354	Prostaglandin G/H synthase 2 OS=Homo sapiens GN=PTGS2 PE=1 SV=2	37	15	69
P09543	2',3'-cyclic-nucleotide 3'-phosphodiesterase OS=Homo sapiens GN=CNP PE=1 SV=2	37	14	47.5
P26641	Elongation factor 1-gamma OS=Homo sapiens GN=EEF1G PE=1 SV=3	37	14	50.1
Q02818	Nucleobindin-1 OS=Homo sapiens GN=NUCB1 PE=1 SV=4	37	12	53.8
O14874	[3-methyl-2-oxobutanoate dehydrogenase [lipoamide]] kinase, mitochondrial OS=Homo sapiens GN=BCKDK PE=1 SV=2	37	10	46.3
P49406	39S ribosomal protein L19, mitochondrial OS=Homo sapiens GN=MRPL19 PE=1 SV=2	37	10	33.5
E7ETU7	39S ribosomal protein L3, mitochondrial OS=Homo sapiens GN=MRPL3 PE=1 SV=1	37	10	41.6
Q9BYD2	39S ribosomal protein L9, mitochondrial OS=Homo sapiens GN=MRPL9 PE=1 SV=2	37	10	30.2
Q13084	39S ribosomal protein L28, mitochondrial OS=Homo sapiens GN=MRPL28 PE=1 SV=4	37	9	30.1
H0Y368	Dolichol-phosphate mannosyltransferase subunit 1 (Fragment) OS=Homo sapiens GN=DPM1 PE=1 SV=1	37	9	33.3
P25325	3-mercaptopyruvate sulfurtransferase OS=Homo sapiens GN=MPST PE=1 SV=3	37	8	33.2
Q9NR28	Diablo homolog, mitochondrial OS=Homo sapiens GN=DIABLO PE=1 SV=1	37	8	27.1
Q8IY95	Transmembrane protein 192 OS=Homo sapiens GN=TMEM192 PE=1 SV=1	37	8	30.9
P62280	40S ribosomal protein S11 OS=Homo sapiens GN=RPS11 PE=1 SV=3	37	7	18.4
A8K878	Mesencephalic astrocyte-derived neurotrophic factor OS=Homo sapiens GN=MANF PE=1 SV=1	37	7	21.1
Q8WUY1	Protein THEM6 OS=Homo sapiens GN=THEM6 PE=1 SV=2	37	6	23.9
Q96AH8	Ras-related protein Rab-7b OS=Homo sapiens GN=RAB7B PE=2 SV=1	37	6	22.5
P51571	Translocon-associated protein subunit delta OS=Homo sapiens GN=SSR4 PE=1 SV=1	37	6	19
Q9Y3B3	Transmembrane emp24 domain-containing protein 7 OS=Homo sapiens GN=TMED7 PE=1 SV=2	37	6	25.2
Q6P587	Acylpyruvase FAHD1, mitochondrial OS=Homo sapiens GN=FAHD1 PE=1 SV=2	37	5	24.8
Q9UJU6	Drebrin-like protein OS=Homo sapiens GN=DBNL PE=1 SV=1	37	5	48.2
P62854	40S ribosomal protein S26 OS=Homo sapiens GN=RPS26 PE=1 SV=3	37	4	13
O00483	Cytochrome c oxidase subunit NDUFA4 OS=Homo sapiens GN=NDUFA4 PE=1 SV=1	37	3	9.4
P63167	Dynein light chain 1, cytoplasmic OS=Homo sapiens GN=DYNLL1 PE=1 SV=1	37	2	10.4
P60059	Protein transport protein Sec61 subunit gamma OS=Homo sapiens GN=SEC61G PE=1 SV=1	37	2	7.7

Q93084	Sarcoplasmic/endoplasmic reticulum calcium ATPase 3 OS=Homo sapiens GN=ATP2A3 PE=1 SV=2	36	28	113.9
Q13488	V-type proton ATPase 116 kDa subunit a isoform 3 OS=Homo sapiens GN=TCIRG1 PE=1 SV=3	36	24	92.9
A0A0A6YYL4	Coronin OS=Homo sapiens GN=CORO7-PAM16 PE=3 SV=1	36	22	114.1
Q05655	Protein kinase C delta type OS=Homo sapiens GN=PRKCD PE=1 SV=2	36	18	77.5
Q99832	T-complex protein 1 subunit eta OS=Homo sapiens GN=CCT7 PE=1 SV=2	36	14	59.3
P30533	Alpha-2-macroglobulin receptor-associated protein OS=Homo sapiens GN=LRPAP1 PE=1 SV=1	36	13	41.4
P53634	Dipeptidyl peptidase 1 OS=Homo sapiens GN=CTSC PE=1 SV=2	36	13	51.8
Q96DZ1	Endoplasmic reticulum lectin 1 OS=Homo sapiens GN=ERLEC1 PE=1 SV=1	36	13	54.8
P00738	Haptoglobin OS=Homo sapiens GN=HP PE=1 SV=1	36	13	45.2
Q9UQ80	Proliferation-associated protein 2G4 OS=Homo sapiens GN=PA2G4 PE=1 SV=3	36	13	43.8
Q4G0N4	NAD kinase 2, mitochondrial OS=Homo sapiens GN=NADK2 PE=1 SV=2	36	12	49.4
Q9HD33	39S ribosomal protein L47, mitochondrial OS=Homo sapiens GN=MRPL47 PE=1 SV=2	36	11	29.4
P46777	60S ribosomal protein L5 OS=Homo sapiens GN=RPL5 PE=1 SV=3	36	11	34.3
Q9Y606	tRNA pseudouridine synthase A, mitochondrial OS=Homo sapiens GN=PUS1 PE=1 SV=3	36	11	47.4
Q5T653	39S ribosomal protein L2, mitochondrial OS=Homo sapiens GN=MRPL2 PE=1 SV=2	36	10	33.3
P07858	Cathepsin B OS=Homo sapiens GN=CTSB PE=1 SV=3	36	10	37.8
P13489	Ribonuclease inhibitor OS=Homo sapiens GN=RNH1 PE=1 SV=2	36	10	49.9
O14773	Tripeptidyl-peptidase 1 OS=Homo sapiens GN=TPP1 PE=1 SV=2	36	10	61.2
Q8IVS2	Malonyl-CoA-acyl carrier protein transacylase, mitochondrial OS=Homo sapiens GN=MCAT PE=1 SV=2	36	9	42.9
P53597	Succinate--CoA ligase [ADP/GDP-forming] subunit alpha, mitochondrial OS=Homo sapiens GN=SUCLG1 PE=1 SV=4	36	9	36.2
Q9NWU1	3-oxoacyl-[acyl-carrier-protein] synthase, mitochondrial OS=Homo sapiens GN=OXSM PE=1 SV=1	36	8	48.8
Q8N2F6	Armadillo repeat-containing protein 10 OS=Homo sapiens GN=ARMC10 PE=1 SV=1	36	8	37.5
Q9P0I2	ER membrane protein complex subunit 3 OS=Homo sapiens GN=EMC3 PE=1 SV=3	36	7	29.9
O75208	Ubiquinone biosynthesis protein COQ9, mitochondrial OS=Homo sapiens GN=COQ9 PE=1 SV=1	36	7	35.5
Q07812	Apoptosis regulator BAX OS=Homo sapiens GN=BAX PE=1 SV=1	36	6	21.2
Q9NP72	Ras-related protein Rab-18 OS=Homo sapiens GN=RAB18 PE=1 SV=1	36	6	23
Q9BYC9	39S ribosomal protein L20, mitochondrial OS=Homo sapiens GN=MRPL20 PE=1 SV=1	36	5	17.4
Q9P0M9	39S ribosomal protein L27, mitochondrial OS=Homo sapiens GN=MRPL27 PE=1 SV=1	36	5	16.1

Q8N983	39S ribosomal protein L43, mitochondrial OS=Homo sapiens GN=MRPL43 PE=1 SV=1	36	5	23.4
Q9Y2R0	Cytochrome c oxidase assembly factor 3 homolog, mitochondrial OS=Homo sapiens GN=COA3 PE=1 SV=1	36	4	11.7
K7EK60	Formin-like protein 1 OS=Homo sapiens GN=FMNL1 PE=1 SV=1	36	3	74.6
O75368	SH3 domain-binding glutamic acid-rich-like protein OS=Homo sapiens GN=SH3BGRL PE=1 SV=1	36	3	12.8
Q9P1F3	Costars family protein ABRACL OS=Homo sapiens GN=ABRACL PE=1 SV=1	36	2	9.1
P53396	ATP-citrate synthase OS=Homo sapiens GN=ACLY PE=1 SV=3	35	29	120.8
O76031	ATP-dependent Clp protease ATP-binding subunit clpX-like, mitochondrial OS=Homo sapiens GN=CLPX PE=1 SV=2	35	21	69.2
O75027	ATP-binding cassette sub-family B member 7, mitochondrial OS=Homo sapiens GN=ABCB7 PE=1 SV=2	35	20	82.6
Q02809	Procollagen-lysine,2-oxoglutarate 5-dioxygenase 1 OS=Homo sapiens GN=PLOD1 PE=1 SV=2	35	19	83.5
Q86SF2	N-acetylgalactosaminyltransferase 7 OS=Homo sapiens GN=GALNT7 PE=1 SV=1	35	18	75.3
P17858	ATP-dependent 6-phosphofructokinase, liver type OS=Homo sapiens GN=PFKL PE=1 SV=6	35	15	85
O94925	Glutaminase kidney isoform, mitochondrial OS=Homo sapiens GN=GLS PE=1 SV=1	35	15	73.4
P15586	N-acetylglucosamine-6-sulfatase OS=Homo sapiens GN=GNS PE=1 SV=3	35	15	62
P52209	6-phosphogluconate dehydrogenase, decarboxylating OS=Homo sapiens GN=PGD PE=1 SV=3	35	14	53.1
Q14699	Raftlin OS=Homo sapiens GN=RFTN1 PE=1 SV=4	35	13	63.1
P40227	T-complex protein 1 subunit zeta OS=Homo sapiens GN=CCT6A PE=1 SV=3	35	13	58
P35613	Basigin OS=Homo sapiens GN=BSG PE=1 SV=2	35	11	42.2
Q9Y697	Cysteine desulfurase, mitochondrial OS=Homo sapiens GN=NFS1 PE=1 SV=3	35	11	50.2
Q6PCB8	Embigin OS=Homo sapiens GN=EMB PE=1 SV=1	35	11	36.9
Q9Y6M5	Zinc transporter 1 OS=Homo sapiens GN=SLC30A1 PE=1 SV=3	35	11	55.3
P53007	Tricarboxylate transport protein, mitochondrial OS=Homo sapiens GN=SLC25A1 PE=1 SV=2	35	10	34
O60884	DnaJ homolog subfamily A member 2 OS=Homo sapiens GN=DNAJA2 PE=1 SV=1	35	9	45.7
Q9H936	Mitochondrial glutamate carrier 1 OS=Homo sapiens GN=SLC25A22 PE=1 SV=1	35	9	34.4
Q9UNE7	E3 ubiquitin-protein ligase CHIP OS=Homo sapiens GN=STUB1 PE=1 SV=2	35	8	34.8
P50897	Palmitoyl-protein thioesterase 1 OS=Homo sapiens GN=PPT1 PE=1 SV=1	35	8	34.2
Q96A26	Protein FAM162A OS=Homo sapiens GN=FAM162A PE=1 SV=2	35	8	17.3
Q8NBN7	Retinol dehydrogenase 13 OS=Homo sapiens GN=RDH13 PE=1 SV=2	35	8	35.9
Q15019	Septin-2 OS=Homo sapiens GN=SEPT2 PE=1 SV=1	35	8	41.5

Q9BTZ2	Dehydrogenase/reductase SDR family member 4 OS=Homo sapiens GN=DHRS4 PE=1 SV=3	35	7	29.5
O00165	HCLS1-associated protein X-1 OS=Homo sapiens GN=HAX1 PE=1 SV=2	35	7	31.6
A0A0G2JNQ7	Leukocyte immunoglobulin-like receptor subfamily B member 2 OS=Homo sapiens GN=LILRB2 PE=1 SV=1	35	7	64.9
Q969Q5	Ras-related protein Rab-24 OS=Homo sapiens GN=RAB24 PE=1 SV=1	35	7	23.1
Q96D96	Voltage-gated hydrogen channel 1 OS=Homo sapiens GN=HVCN1 PE=1 SV=1	35	7	31.7
P62906	60S ribosomal protein L10a OS=Homo sapiens GN=RPL10A PE=1 SV=2	35	6	24.8
P12318	Low affinity immunoglobulin gamma Fc region receptor II-a OS=Homo sapiens GN=FCGR2A PE=1 SV=4	35	6	35
P16949	Stathmin OS=Homo sapiens GN=STMN1 PE=1 SV=3	35	6	17.3
Q13405	39S ribosomal protein L49, mitochondrial OS=Homo sapiens GN=MRPL49 PE=1 SV=1	35	5	19.2
P62829	60S ribosomal protein L23 OS=Homo sapiens GN=RPL23 PE=1 SV=1	35	5	14.9
Q16595	Frataxin, mitochondrial OS=Homo sapiens GN=FXN PE=1 SV=2	35	5	23.1
P69905	Hemoglobin subunit alpha OS=Homo sapiens GN=HBA1 PE=1 SV=2	35	5	15.2
P20338	Ras-related protein Rab-4A OS=Homo sapiens GN=RAB4A PE=1 SV=3	35	5	24.4
Q9NX14	NADH dehydrogenase [ubiquinone] 1 beta subcomplex subunit 11, mitochondrial OS=Homo sapiens GN=NDUFB11 PE=1 SV=1	35	4	17.3
Q6RW13	Type-1 angiotensin II receptor-associated protein OS=Homo sapiens GN=AGTRAP PE=1 SV=1	35	4	17.4
Q8NF50	Dedicator of cytokinesis protein 8 OS=Homo sapiens GN=DOCK8 PE=1 SV=3	34	55	238.4
Q92608	Dedicator of cytokinesis protein 2 OS=Homo sapiens GN=DOCK2 PE=1 SV=2	34	45	211.8
P33527	Multidrug resistance-associated protein 1 OS=Homo sapiens GN=ABCC1 PE=1 SV=3	34	38	171.5
Q8TCS8	Polyribonucleotide nucleotidyltransferase 1, mitochondrial OS=Homo sapiens GN=PNPT1 PE=1 SV=2	34	22	85.9
P06396	Gelsolin OS=Homo sapiens GN=GSN PE=1 SV=1	34	19	85.6
P28288	ATP-binding cassette sub-family D member 3 OS=Homo sapiens GN=ABCD3 PE=1 SV=1	34	16	75.4
Q8NCL4	Polypeptide N-acetylgalactosaminyltransferase 6 OS=Homo sapiens GN=GALNT6 PE=2 SV=2	34	15	71.1
Q9UHL4	Dipeptidyl peptidase 2 OS=Homo sapiens GN=DPP7 PE=1 SV=3	34	12	54.3
O95466	Formin-like protein 1 OS=Homo sapiens GN=FMNL1 PE=1 SV=3	34	12	121.8
Q9P035	Very-long-chain (3R)-3-hydroxyacyl-CoA dehydratase 3 OS=Homo sapiens GN=HACD3 PE=1 SV=2	34	12	43.1
Q9NV96	Cell cycle control protein 50A OS=Homo sapiens GN=TMEM30A PE=1 SV=1	34	11	40.7
P21283	V-type proton ATPase subunit C 1 OS=Homo sapiens GN=ATP6V1C1 PE=1 SV=4	34	11	43.9
O60711	Leupaxin OS=Homo sapiens GN=LPXN PE=1 SV=1	34	10	43.3

O15382	Branched-chain-amino-acid aminotransferase, mitochondrial OS=Homo sapiens GN=BCAT2 PE=1 SV=2	34	9	44.3
A0A0A6YYL6	Protein RPL17-C18orf32 OS=Homo sapiens GN=RPL17-C18orf32 PE=3 SV=1	34	7	26.4
Q15363	Transmembrane emp24 domain-containing protein 2 OS=Homo sapiens GN=TMED2 PE=1 SV=1	34	7	22.7
P13747	HLA class I histocompatibility antigen, alpha chain E OS=Homo sapiens GN=HLA-E PE=1 SV=3	34	6	40.1
Q15181	Inorganic pyrophosphatase OS=Homo sapiens GN=PPA1 PE=1 SV=2	34	6	32.6
Q8WUK0	Phosphatidylglycerophosphatase and protein-tyrosine phosphatase 1 OS=Homo sapiens GN=PTPMT1 PE=1 SV=1	34	6	22.8
P25789	Proteasome subunit alpha type-4 OS=Homo sapiens GN=PSMA4 PE=1 SV=1	34	6	29.5
P61009	Signal peptidase complex subunit 3 OS=Homo sapiens GN=SPCS3 PE=1 SV=1	34	6	20.3
Q04917	14-3-3 protein eta OS=Homo sapiens GN=YWHAH PE=1 SV=4	34	5	28.2
P55957	BH3-interacting domain death agonist OS=Homo sapiens GN=BID PE=1 SV=1	34	4	22
A0A0C4DG91	Nucleoside diphosphate kinase OS=Homo sapiens GN=NME6 PE=1 SV=1	34	4	22
Q9Y2Q9	28S ribosomal protein S28, mitochondrial OS=Homo sapiens GN=MRPS28 PE=1 SV=1	34	3	20.8
Q9Y6H1	Coiled-coil-helix-coiled-coil-helix domain-containing protein 2 OS=Homo sapiens GN=CHCHD2 PE=1 SV=1	34	3	15.5
G3V2N0	Guanine nucleotide-binding protein subunit gamma OS=Homo sapiens GN=GNG2 PE=1 SV=1	34	3	12.4
O60888	Protein CutA OS=Homo sapiens GN=CUTA PE=1 SV=2	34	3	19.1
P31949	Protein S100-A11 OS=Homo sapiens GN=S100A11 PE=1 SV=2	34	3	11.7
Q9Y5U9	Immediate early response 3-interacting protein 1 OS=Homo sapiens GN=IER3IP1 PE=1 SV=1	34	2	9
P56181	NADH dehydrogenase [ubiquinone] flavoprotein 3, mitochondrial OS=Homo sapiens GN=NDUFV3 PE=1 SV=2	34	2	11.9
P07814	Bifunctional glutamate/proline--tRNA ligase OS=Homo sapiens GN=EPRS PE=1 SV=5	33	34	170.5
O75976	Carboxypeptidase D OS=Homo sapiens GN=CPD PE=1 SV=2	33	33	152.8
P46459	Vesicle-fusing ATPase OS=Homo sapiens GN=NSF PE=1 SV=3	33	21	82.5
Q8TDW0	Volume-regulated anion channel subunit LRRC8C OS=Homo sapiens GN=LRRC8C PE=1 SV=2	33	19	92.4
Q9NPH3	Interleukin-1 receptor accessory protein OS=Homo sapiens GN=IL1RAP PE=1 SV=2	33	18	65.4
O14786	Neuropilin-1 OS=Homo sapiens GN=NRP1 PE=1 SV=3	33	18	103.1
O75844	CAAX prenyl protease 1 homolog OS=Homo sapiens GN=ZMPSTE24 PE=1 SV=2	33	13	54.8
P49189	4-trimethylaminobutyraldehyde dehydrogenase OS=Homo sapiens GN=ALDH9A1 PE=1 SV=3	33	12	53.8
O75427	Leucine-rich repeat and calponin homology domain-containing protein 4 OS=Homo sapiens GN=LRCH4 PE=1 SV=2	33	12	73.4
Q9NZ01	Very-long-chain enoyl-CoA reductase OS=Homo sapiens GN=TECR PE=1 SV=1	33	12	36

Q9UJ83	2-hydroxyacyl-CoA lyase 1 OS=Homo sapiens GN=HACL1 PE=1 SV=2	33	11	63.7
P51572	B-cell receptor-associated protein 31 OS=Homo sapiens GN=BCAP31 PE=1 SV=3	33	11	28
P36542	ATP synthase subunit gamma, mitochondrial OS=Homo sapiens GN=ATP5C1 PE=1 SV=1	33	10	33
Q9Y673	Dolichyl-phosphate beta-glucosyltransferase OS=Homo sapiens GN=ALG5 PE=1 SV=1	33	10	36.9
Q9H939	Proline-serine-threonine phosphatase-interacting protein 2 OS=Homo sapiens GN=PSTPIP2 PE=1 SV=4	33	9	38.8
J3KQJ1	Sulfatase-modifying factor 2 OS=Homo sapiens GN=SUMF2 PE=1 SV=1	33	9	35.9
H0YKD8	60S ribosomal protein L28 OS=Homo sapiens GN=RPL28 PE=1 SV=1	33	8	19.1
O14745	Na(+)/H(+) exchange regulatory cofactor NHE-RF1 OS=Homo sapiens GN=SLC9A3R1 PE=1 SV=4	33	8	38.8
Q9BYC8	39S ribosomal protein L32, mitochondrial OS=Homo sapiens GN=MRPL32 PE=1 SV=1	33	7	21.4
O43488	Aflatoxin B1 aldehyde reductase member 2 OS=Homo sapiens GN=AKR7A2 PE=1 SV=3	33	7	39.6
A0A0G2JM57	Leukocyte immunoglobulin-like receptor subfamily B member 4 OS=Homo sapiens GN=LILRB4 PE=1 SV=1	33	7	49.1
G3V5Z7	Proteasome subunit alpha type OS=Homo sapiens GN=PSMA6 PE=1 SV=1	33	7	28.1
Q9H0U6	39S ribosomal protein L18, mitochondrial OS=Homo sapiens GN=MRPL18 PE=1 SV=1	33	6	20.6
Q8N9N7	Leucine-rich repeat-containing protein 57 OS=Homo sapiens GN=LRRC57 PE=1 SV=1	33	6	26.7
Q9Y3Q3	Transmembrane emp24 domain-containing protein 3 OS=Homo sapiens GN=TMED3 PE=1 SV=1	33	6	24.8
P46783	40S ribosomal protein S10 OS=Homo sapiens GN=RPS10 PE=1 SV=1	33	5	18.9
P46778	60S ribosomal protein L21 OS=Homo sapiens GN=RPL21 PE=1 SV=2	33	4	18.6
P13284	Gamma-interferon-inducible lysosomal thiol reductase OS=Homo sapiens GN=IFI30 PE=1 SV=3	33	4	27.9
P31994	Low affinity immunoglobulin gamma Fc region receptor II-b OS=Homo sapiens GN=FCGR2B PE=1 SV=2	33	4	34
H0YIV9	Uncharacterized protein (Fragment) OS=Homo sapiens PE=3 SV=1	33	4	18.7
O15235	28S ribosomal protein S12, mitochondrial OS=Homo sapiens GN=MRPS12 PE=1 SV=1	33	3	15.2
P06753	Tropomyosin alpha-3 chain OS=Homo sapiens GN=TPM3 PE=1 SV=2	33	3	32.9
P62857	40S ribosomal protein S28 OS=Homo sapiens GN=RPS28 PE=1 SV=1	33	2	7.8
P07996	Thrombospondin-1 OS=Homo sapiens GN=THBS1 PE=1 SV=2	32	30	129.3
P18206	Vinculin OS=Homo sapiens GN=VCL PE=1 SV=4	32	26	123.7
P08648	Integrin alpha-5 OS=Homo sapiens GN=ITGA5 PE=1 SV=2	32	22	114.5
Q92556	Engulfment and cell motility protein 1 OS=Homo sapiens GN=ELMO1 PE=1 SV=2	32	17	83.8
P82675	28S ribosomal protein S5, mitochondrial OS=Homo sapiens GN=MRPS5 PE=1 SV=2	32	14	48

A0A0G2JHC2	Phostensin OS=Homo sapiens GN=PPP1R18 PE=1 SV=1	32	13	68
O00330	Pyruvate dehydrogenase protein X component, mitochondrial OS=Homo sapiens GN=PDHX PE=1 SV=3	32	13	54.1
Q9Y230	RuvB-like 2 OS=Homo sapiens GN=RUVBL2 PE=1 SV=3	32	13	51.1
P06865	Beta-hexosaminidase subunit alpha OS=Homo sapiens GN=HEXA PE=1 SV=2	32	12	60.7
P07686	Beta-hexosaminidase subunit beta OS=Homo sapiens GN=HEXB PE=1 SV=3	32	12	63.1
Q15118	[Pyruvate dehydrogenase (acetyl-transferring)] kinase isozyme 1, mitochondrial OS=Homo sapiens GN=PDK1 PE=1 SV=1	32	11	49.2
Q9H0R6	Glutamyl-tRNA(Gln) amidotransferase subunit A, mitochondrial OS=Homo sapiens GN=QRSL1 PE=1 SV=2	32	11	57.4
P31689	DnaJ homolog subfamily A member 1 OS=Homo sapiens GN=DNAJA1 PE=1 SV=2	32	10	44.8
P35914	Hydroxymethylglutaryl-CoA lyase, mitochondrial OS=Homo sapiens GN=HMGCL PE=1 SV=2	32	10	34.3
P40925	Malate dehydrogenase, cytoplasmic OS=Homo sapiens GN=MDH1 PE=1 SV=4	32	10	36.4
Q8NBL1	Protein O-glucosyltransferase 1 OS=Homo sapiens GN=POGLUT1 PE=1 SV=1	32	10	46.2
Q8IZ81	ELMO domain-containing protein 2 OS=Homo sapiens GN=ELMOD2 PE=1 SV=1	32	9	34.9
E7EPK1	Septin-7 OS=Homo sapiens GN=SEPT7 PE=1 SV=2	32	9	50.7
Q86WV6	Stimulator of interferon genes protein OS=Homo sapiens GN=TMEM173 PE=1 SV=1	32	9	42.2
Q00059	Transcription factor A, mitochondrial OS=Homo sapiens GN=TFAM PE=1 SV=1	32	9	29.1
P32121	Beta-arrestin-2 OS=Homo sapiens GN=ARRB2 PE=1 SV=2	32	8	46.1
P36543	V-type proton ATPase subunit E 1 OS=Homo sapiens GN=ATP6V1E1 PE=1 SV=1	32	8	26.1
P15880	40S ribosomal protein S2 OS=Homo sapiens GN=RPS2 PE=1 SV=2	32	7	31.3
J3QQ67	60S ribosomal protein L18 (Fragment) OS=Homo sapiens GN=RPL18 PE=1 SV=1	32	7	21.8
Q16740	ATP-dependent Clp protease proteolytic subunit, mitochondrial OS=Homo sapiens GN=CLPP PE=1 SV=1	32	7	30.2
Q96FZ7	Charged multivesicular body protein 6 OS=Homo sapiens GN=CHMP6 PE=1 SV=3	32	7	23.5
Q9NY25	C-type lectin domain family 5 member A OS=Homo sapiens GN=CLEC5A PE=1 SV=1	32	7	21.5
P42126	Enoyl-CoA delta isomerase 1, mitochondrial OS=Homo sapiens GN=ECI1 PE=1 SV=1	32	7	32.8
P09972	Fructose-bisphosphate aldolase C OS=Homo sapiens GN=ALDOC PE=1 SV=2	32	7	39.4
P01584	Interleukin-1 beta OS=Homo sapiens GN=IL1B PE=1 SV=2	32	7	30.7
O75915	PRA1 family protein 3 OS=Homo sapiens GN=ARL6IP5 PE=1 SV=1	32	7	21.6
Q9GZY8	Mitochondrial fission factor OS=Homo sapiens GN=MFF PE=1 SV=1	32	6	38.4

O43181	NADH dehydrogenase [ubiquinone] iron-sulfur protein 4, mitochondrial OS=Homo sapiens GN=NDUFS4 PE=1 SV=1	32	6	20.1
P55209	Nucleosome assembly protein 1-like 1 OS=Homo sapiens GN=NAP1L1 PE=1 SV=1	32	6	45.3
Q9BY50	Signal peptidase complex catalytic subunit SEC11C OS=Homo sapiens GN=SEC11C PE=1 SV=3	32	6	21.5
Q5VYY1	Ankyrin repeat domain-containing protein 22 OS=Homo sapiens GN=ANKRD22 PE=2 SV=1	32	5	21.8
Q9ULZ3	Apoptosis-associated speck-like protein containing a CARD OS=Homo sapiens GN=PYCARD PE=1 SV=2	32	5	21.6
P26583	High mobility group protein B2 OS=Homo sapiens GN=HMGB2 PE=1 SV=2	32	5	24
P78368	Casein kinase I isoform gamma-2 OS=Homo sapiens GN=CSNK1G2 PE=1 SV=1	32	4	47.4
Q9NR31	GTP-binding protein SAR1a OS=Homo sapiens GN=SAR1A PE=1 SV=1	32	4	22.4
Q99720	Sigma non-opioid intracellular receptor 1 OS=Homo sapiens GN=SIGMAR1 PE=1 SV=1	32	4	25.1
Q9Y6A9	Signal peptidase complex subunit 1 OS=Homo sapiens GN=SPCS1 PE=1 SV=4	32	4	11.8
Q9Y3D5	28S ribosomal protein S18c, mitochondrial OS=Homo sapiens GN=MRPS18C PE=1 SV=1	32	3	15.8
Q9Y5J6	Mitochondrial import inner membrane translocase subunit Tim10 B OS=Homo sapiens GN=TIMM10B PE=1 SV=1	32	3	11.6
Q99595	Mitochondrial import inner membrane translocase subunit Tim17-A OS=Homo sapiens GN=TIMM17A PE=1 SV=1	32	3	18
Q9H299	SH3 domain-binding glutamic acid-rich-like protein 3 OS=Homo sapiens GN=SH3BGRL3 PE=1 SV=1	32	3	10.4
O14957	Cytochrome b-c1 complex subunit 10 OS=Homo sapiens GN=UQCR11 PE=3 SV=1	32	2	6.6
P13640	Metallothionein-1G OS=Homo sapiens GN=MT1G PE=1 SV=2	32	2	6.1
P58546	Myotrophin OS=Homo sapiens GN=MTPN PE=1 SV=2	32	2	12.9
O15439	Multidrug resistance-associated protein 4 OS=Homo sapiens GN=ABCC4 PE=1 SV=3	31	30	149.4
Q92896	Golgi apparatus protein 1 OS=Homo sapiens GN=GLG1 PE=1 SV=2	31	29	134.5
Q8WUM4	Programmed cell death 6-interacting protein OS=Homo sapiens GN=PDCD6IP PE=1 SV=1	31	25	96
Q7KZF4	Staphylococcal nuclease domain-containing protein 1 OS=Homo sapiens GN=SND1 PE=1 SV=1	31	21	101.9
Q969N2	GPI transamidase component PIG-T OS=Homo sapiens GN=PIGT PE=1 SV=1	31	16	65.7
Q15392	Delta(24)-sterol reductase OS=Homo sapiens GN=DHCR24 PE=1 SV=2	31	15	60.1
P31948	Stress-induced-phosphoprotein 1 OS=Homo sapiens GN=STIP1 PE=1 SV=1	31	15	62.6
P19878	Neutrophil cytosol factor 2 OS=Homo sapiens GN=NCF2 PE=1 SV=2	31	13	59.7
P08670	Vimentin OS=Homo sapiens GN=VIM PE=1 SV=4	31	13	53.6
A0A024R6I7	Alpha-1-antitrypsin OS=Homo sapiens GN=SERPINA1 PE=1 SV=1	31	12	46.7

Q13740	CD166 antigen OS=Homo sapiens GN=ALCAM PE=1 SV=2	31	12	65.1
Q96KA5	Cleft lip and palate transmembrane protein 1-like protein OS=Homo sapiens GN=CLPTM1L PE=1 SV=1	31	12	62.2
Q15904	V-type proton ATPase subunit S1 OS=Homo sapiens GN=ATP6AP1 PE=1 SV=2	31	12	52
P43007	Neutral amino acid transporter A OS=Homo sapiens GN=SLC1A4 PE=1 SV=1	31	11	55.7
Q9C0E8	Protein lunapark OS=Homo sapiens GN=LNP PE=1 SV=2	31	11	47.7
Q9Y2Z4	Tyrosine--tRNA ligase, mitochondrial OS=Homo sapiens GN=YARS2 PE=1 SV=2	31	11	53.2
A0A087X1S7	CD48 antigen OS=Homo sapiens GN=CD48 PE=1 SV=1	31	10	28.8
Q9UKU7	Isobutyryl-CoA dehydrogenase, mitochondrial OS=Homo sapiens GN=ACAD8 PE=1 SV=1	31	9	45
P30740	Leukocyte elastase inhibitor OS=Homo sapiens GN=SERPINB1 PE=1 SV=1	31	9	42.7
Q9NUI1	Peroxisomal 2,4-dienoyl-CoA reductase OS=Homo sapiens GN=DECR2 PE=1 SV=1	31	9	30.8
Q13492	Phosphatidylinositol-binding clathrin assembly protein OS=Homo sapiens GN=PICALM PE=1 SV=2	31	9	70.7
P15291	Beta-1,4-galactosyltransferase 1 OS=Homo sapiens GN=B4GALT1 PE=1 SV=5	31	8	43.9
Q8IWE4	DCN1-like protein 3 OS=Homo sapiens GN=DCUN1D3 PE=1 SV=1	31	8	34.3
P17931	Galectin-3 OS=Homo sapiens GN=LGALS3 PE=1 SV=5	31	7	26.1
P10620	Microsomal glutathione S-transferase 1 OS=Homo sapiens GN=MGST1 PE=1 SV=1	31	7	17.6
Q14197	Peptidyl-tRNA hydrolase ICT1, mitochondrial OS=Homo sapiens GN=MRPL58 PE=1 SV=1	31	7	23.6
Q8N490	Probable hydrolase PNKD OS=Homo sapiens GN=PNKD PE=1 SV=2	31	7	42.8
Q8IUW5	RELT-like protein 1 OS=Homo sapiens GN=RELL1 PE=1 SV=1	31	7	29.3
O15145	Actin-related protein 2/3 complex subunit 3 OS=Homo sapiens GN=ARPC3 PE=1 SV=3	31	6	20.5
Q9H6E4	Coiled-coil domain-containing protein 134 OS=Homo sapiens GN=CCDC134 PE=1 SV=1	31	6	26.5
P33316	Deoxyuridine 5'-triphosphate nucleotidohydrolase, mitochondrial OS=Homo sapiens GN=DUT PE=1 SV=4	31	6	26.5
Q9BSF4	Mitochondrial import inner membrane translocase subunit Tim29 OS=Homo sapiens GN=TIMM29 PE=1 SV=2	31	6	29.2
A0A087X1Z3	Proteasome activator complex subunit 2 OS=Homo sapiens GN=PSME2 PE=1 SV=1	31	6	29.1
P83731	60S ribosomal protein L24 OS=Homo sapiens GN=RPL24 PE=1 SV=1	31	5	17.8
Q96HY6	DDRGK domain-containing protein 1 OS=Homo sapiens GN=DDRGK1 PE=1 SV=2	31	5	35.6
Q8NCW5	NAD(P)H-hydrate epimerase OS=Homo sapiens GN=NAXE PE=1 SV=2	31	5	31.7
Q9BUL8	Programmed cell death protein 10 OS=Homo sapiens GN=PDCD10 PE=1 SV=1	31	5	24.7
Q14141	Septin-6 OS=Homo sapiens GN=SEPT6 PE=1 SV=4	31	5	49.7

P43307	Translocon-associated protein subunit alpha OS=Homo sapiens GN=SSR1 PE=1 SV=3	31	5	32.2
Q9GZY4	Cytochrome c oxidase assembly factor 1 homolog OS=Homo sapiens GN=COA1 PE=1 SV=1	31	4	16.7
Q9Y287	Integral membrane protein 2B OS=Homo sapiens GN=ITM2B PE=1 SV=1	31	4	30.3
O95881	Thioredoxin domain-containing protein 12 OS=Homo sapiens GN=TXNDC12 PE=1 SV=1	31	4	19.2
A0A0A0MTI5	Acyl-CoA-binding protein OS=Homo sapiens GN=DBI PE=1 SV=1	31	3	15.9
P56381	ATP synthase subunit epsilon, mitochondrial OS=Homo sapiens GN=ATP5E PE=1 SV=2	31	2	5.8
Q9HD89	Resistin OS=Homo sapiens GN=RETN PE=1 SV=1	31	2	11.4
O00160	Unconventional myosin-If OS=Homo sapiens GN=MYO1F PE=1 SV=3	30	28	124.8
P20701	Integrin alpha-L OS=Homo sapiens GN=ITGAL PE=1 SV=3	30	24	128.7
V9GYM8	Rho guanine nucleotide exchange factor 2 OS=Homo sapiens GN=ARHGEF2 PE=1 SV=1	30	24	116
M0QZR4	Rho guanine nucleotide exchange factor 1 OS=Homo sapiens GN=ARHGEF1 PE=1 SV=1	30	23	108.3
Q93050	V-type proton ATPase 116 kDa subunit a isoform 1 OS=Homo sapiens GN=ATP6V0A1 PE=1 SV=3	30	20	96.4
Q7Z2K6	Endoplasmic reticulum metallopeptidase 1 OS=Homo sapiens GN=ERMP1 PE=1 SV=2	30	19	100.2
O94804	Serine/threonine-protein kinase 10 OS=Homo sapiens GN=STK10 PE=1 SV=1	30	19	112.1
Q96EY7	Pentatricopeptide repeat domain-containing protein 3, mitochondrial OS=Homo sapiens GN=PTCD3 PE=1 SV=3	30	16	78.5
Q12797	Aspartyl/asparaginyl beta-hydroxylase OS=Homo sapiens GN=ASPH PE=1 SV=3	30	15	85.8
Q9HA77	Probable cysteine--tRNA ligase, mitochondrial OS=Homo sapiens GN=CARS2 PE=1 SV=1	30	15	62.2
Q96JB5	CDK5 regulatory subunit-associated protein 3 OS=Homo sapiens GN=CDK5RAP3 PE=1 SV=2	30	13	56.9
A0A1C7CYX9	Dihydropyrimidinase-related protein 2 OS=Homo sapiens GN=DPYSL2 PE=1 SV=1	30	12	73.5
Q96CW1	AP-2 complex subunit mu OS=Homo sapiens GN=AP2M1 PE=1 SV=2	30	11	49.6
Q9H9P8	L-2-hydroxyglutarate dehydrogenase, mitochondrial OS=Homo sapiens GN=L2HGDH PE=1 SV=3	30	11	50.3
Q6DD88	Atlastin-3 OS=Homo sapiens GN=ATL3 PE=1 SV=1	30	10	60.5
Q13057	Bifunctional coenzyme A synthase OS=Homo sapiens GN=COASY PE=1 SV=4	30	10	62.3
P16150	Leukosialin OS=Homo sapiens GN=SPN PE=1 SV=1	30	10	40.3
P23219	Prostaglandin G/H synthase 1 OS=Homo sapiens GN=PTGS1 PE=1 SV=2	30	10	68.6
P15309	Prostatic acid phosphatase OS=Homo sapiens GN=ACPP PE=1 SV=3	30	10	44.5
P04066	Tissue alpha-L-fucosidase OS=Homo sapiens GN=FUCA1 PE=1 SV=4	30	10	53.7
Q9Y399	28S ribosomal protein S2, mitochondrial OS=Homo sapiens GN=MRPS2 PE=1 SV=1	30	9	33.2

Q6IAN0	Dehydrogenase/reductase SDR family member 7B OS=Homo sapiens GN=DHRS7B PE=1 SV=2	30	9	35.1
P42167	Lamina-associated polypeptide 2, isoforms beta/gamma OS=Homo sapiens GN=TMPO PE=1 SV=2	30	9	50.6
Q13177	Serine/threonine-protein kinase PAK 2 OS=Homo sapiens GN=PAK2 PE=1 SV=3	30	9	58
O14734	Acyl-coenzyme A thioesterase 8 OS=Homo sapiens GN=ACOT8 PE=1 SV=1	30	8	35.9
Q15293	Reticulocalbin-1 OS=Homo sapiens GN=RCN1 PE=1 SV=1	30	8	38.9
X5DQS5	Cell adhesion molecule 1 (Fragment) OS=Homo sapiens GN=CADM1 PE=1 SV=1	30	7	48.6
Q68D91	Metallo-beta-lactamase domain-containing protein 2 OS=Homo sapiens GN=MBLAC2 PE=1 SV=3	30	7	31.4
P67809	Nuclease-sensitive element-binding protein 1 OS=Homo sapiens GN=YBX1 PE=1 SV=3	30	7	35.9
Q5W0Z9	Probable palmitoyltransferase ZDHHC20 OS=Homo sapiens GN=ZDHHC20 PE=1 SV=1	30	7	42.3
O15126	Secretory carrier-associated membrane protein 1 OS=Homo sapiens GN=SCAMP1 PE=1 SV=2	30	7	37.9
O75351	Vacuolar protein sorting-associated protein 4B OS=Homo sapiens GN=VPS4B PE=1 SV=2	30	7	49.3
O75251	NADH dehydrogenase [ubiquinone] iron-sulfur protein 7, mitochondrial OS=Homo sapiens GN=NDUFS7 PE=1 SV=3	30	6	23.5
P08567	Pleckstrin OS=Homo sapiens GN=PLEK PE=1 SV=3	30	6	40.1
Q9NUM4	Transmembrane protein 106B OS=Homo sapiens GN=TMEM106B PE=1 SV=2	30	6	31.1
P49207	60S ribosomal protein L34 OS=Homo sapiens GN=RPL34 PE=1 SV=3	30	5	13.3
Q5TDF0	Cancer-related nucleoside-triphosphatase OS=Homo sapiens GN=NTPCR PE=1 SV=1	30	5	25.1
Q9UJ68	Mitochondrial peptide methionine sulfoxide reductase OS=Homo sapiens GN=MSRA PE=1 SV=1	30	5	26.1
P51970	NADH dehydrogenase [ubiquinone] 1 alpha subcomplex subunit 8 OS=Homo sapiens GN=NDUFA8 PE=1 SV=3	30	5	20.1
P35241	Radixin OS=Homo sapiens GN=RDX PE=1 SV=1	30	5	68.5
H0YF29	UPF0598 protein C8orf82 (Fragment) OS=Homo sapiens GN=C8orf82 PE=1 SV=1	30	5	28.6
B7Z4C8	60S ribosomal protein L31 OS=Homo sapiens GN=RPL31 PE=1 SV=1	30	4	15.1
F8W727	60S ribosomal protein L32 OS=Homo sapiens GN=RPL32 PE=1 SV=1	30	4	18
Q04760	Lactoylglutathione lyase OS=Homo sapiens GN=GLO1 PE=1 SV=4	30	4	20.8
O75340	Programmed cell death protein 6 OS=Homo sapiens GN=PDCD6 PE=1 SV=1	30	4	21.9
Q9Y291	28S ribosomal protein S33, mitochondrial OS=Homo sapiens GN=MRPS33 PE=1 SV=1	30	3	12.6
Q7Z7F7	39S ribosomal protein L55, mitochondrial OS=Homo sapiens GN=MRPL55 PE=1 SV=1	30	3	15.1
Q96PE7	Methylmalonyl-CoA epimerase, mitochondrial OS=Homo sapiens GN=MCEE PE=1 SV=1	30	3	18.7
O95139	NADH dehydrogenase [ubiquinone] 1 beta subcomplex subunit 6 OS=Homo sapiens GN=NDUFB6 PE=1 SV=3	30	3	15.5

P39210	Protein Mpv17 OS=Homo sapiens GN=MPV17 PE=1 SV=1	30	3	19.7
P63172	Dynein light chain Tctex-type 1 OS=Homo sapiens GN=DYNLT1 PE=1 SV=1	30	2	12.4
Q96I45	Transmembrane protein 141 OS=Homo sapiens GN=TMEM141 PE=1 SV=1	30	2	11.9
P49327	Fatty acid synthase OS=Homo sapiens GN=FASN PE=1 SV=3	29	49	273.3
P13612	Integrin alpha-4 OS=Homo sapiens GN=ITGA4 PE=1 SV=3	29	25	114.8
P46977	Dolichyl-diphosphooligosaccharide--protein glycosyltransferase subunit STT3A OS=Homo sapiens GN=STT3A PE=1 SV=2	29	22	80.5
B7ZM99	MTHFD1L protein OS=Homo sapiens GN=MTHFD1L PE=1 SV=1	29	22	105.8
Q5JRX3	Presequence protease, mitochondrial OS=Homo sapiens GN=PITRM1 PE=1 SV=3	29	20	117.3
Q5JTZ9	Alanine--tRNA ligase, mitochondrial OS=Homo sapiens GN=AARS2 PE=1 SV=1	29	19	107.3
G0XQ39	STIM1L OS=Homo sapiens GN=STIM1 PE=1 SV=1	29	16	88.6
P48960	CD97 antigen OS=Homo sapiens GN=CD97 PE=1 SV=4	29	15	91.8
Q2M385	Macrophage-expressed gene 1 protein OS=Homo sapiens GN=MPEG1 PE=2 SV=1	29	14	78.5
Q9NZW5	MAGUK p55 subfamily member 6 OS=Homo sapiens GN=MPP6 PE=1 SV=2	29	13	61.1
P23469	Receptor-type tyrosine-protein phosphatase epsilon OS=Homo sapiens GN=PTPRE PE=1 SV=1	29	13	80.6
X5CMH5	Antigen peptide transporter 2 OS=Homo sapiens GN=TAP2 PE=1 SV=1	29	12	77.6
Q96S66	Chloride channel CLIC-like protein 1 OS=Homo sapiens GN=CLCC1 PE=1 SV=1	29	12	62
P63010	AP-2 complex subunit beta OS=Homo sapiens GN=AP2B1 PE=1 SV=1	29	10	104.5
A0A0A0MRK6	Metaxin 1, isoform CRA_b OS=Homo sapiens GN=MTX1 PE=1 SV=1	29	10	51.4
O75695	Protein XRP2 OS=Homo sapiens GN=RP2 PE=1 SV=4	29	10	39.6
Q9NUQ2	1-acyl-sn-glycerol-3-phosphate acyltransferase epsilon OS=Homo sapiens GN=AGPAT5 PE=1 SV=3	29	9	42
Q9Y2Z9	Ubiquinone biosynthesis monooxygenase COQ6, mitochondrial OS=Homo sapiens GN=COQ6 PE=1 SV=2	29	9	50.8
Q9BYD6	39S ribosomal protein L1, mitochondrial OS=Homo sapiens GN=MRPL1 PE=1 SV=2	29	8	36.9
O75648	Mitochondrial tRNA-specific 2-thiouridylase 1 OS=Homo sapiens GN=TRMU PE=1 SV=2	29	8	47.7
Q96E11	Ribosome-recycling factor, mitochondrial OS=Homo sapiens GN=MRRF PE=1 SV=1	29	7	29.3
O15400	Syntaxin-7 OS=Homo sapiens GN=STX7 PE=1 SV=4	29	7	29.8
Q9NYL9	Tropomodulin-3 OS=Homo sapiens GN=TMOD3 PE=1 SV=1	29	7	39.6
P55899	IgG receptor FcRn large subunit p51 OS=Homo sapiens GN=FCGRT PE=1 SV=1	29	6	39.7
Q9HBH1	Peptide deformylase, mitochondrial OS=Homo sapiens GN=PDF PE=1 SV=1	29	6	27

Q9NVA1	Ubiquinol-cytochrome-c reductase complex assembly factor 1 OS=Homo sapiens GN=UQCC1 PE=1 SV=3	29	6	34.6
P17900	Ganglioside GM2 activator OS=Homo sapiens GN=GM2A PE=1 SV=4	29	5	20.8
P09429	High mobility group protein B1 OS=Homo sapiens GN=HMGB1 PE=1 SV=3	29	5	24.9
H0YI09	Methyltransferase-like protein 7A (Fragment) OS=Homo sapiens GN=METTL7A PE=1 SV=1	29	5	28.3
P82664	28S ribosomal protein S10, mitochondrial OS=Homo sapiens GN=MRPS10 PE=1 SV=2	29	4	23
E7EQ34	Golgi SNAP receptor complex member 2 OS=Homo sapiens GN=GOSR2 PE=3 SV=2	29	4	26.8
Q8N4Q1	Mitochondrial intermembrane space import and assembly protein 40 OS=Homo sapiens GN=CHCHD4 PE=1 SV=1	29	4	16
P84103	Serine/arginine-rich splicing factor 3 OS=Homo sapiens GN=SRSF3 PE=1 SV=1	29	4	19.3
P10768	S-formylglutathione hydrolase OS=Homo sapiens GN=ESD PE=1 SV=2	29	4	31.4
O60783	28S ribosomal protein S14, mitochondrial OS=Homo sapiens GN=MRPS14 PE=1 SV=1	29	3	15.1
P62847	40S ribosomal protein S24 OS=Homo sapiens GN=RPS24 PE=1 SV=1	29	3	15.4
I3L504	Eukaryotic translation initiation factor 5A-1 OS=Homo sapiens GN=EIF5A PE=1 SV=1	29	3	20.5
Q9H1K1	Iron-sulfur cluster assembly enzyme ISCU, mitochondrial OS=Homo sapiens GN=ISCU PE=1 SV=2	29	3	18
O43676	NADH dehydrogenase [ubiquinone] 1 beta subcomplex subunit 3 OS=Homo sapiens GN=NDUFB3 PE=1 SV=3	29	3	11.4
Q13232	Nucleoside diphosphate kinase 3 OS=Homo sapiens GN=NME3 PE=1 SV=2	29	3	19
Q96EX1	Small integral membrane protein 12 OS=Homo sapiens GN=SMIM12 PE=1 SV=3	29	3	10.8
P62942	Peptidyl-prolyl cis-trans isomerase FKBP1A OS=Homo sapiens GN=FKBP1A PE=1 SV=2	29	2	11.9
Q14204	Cytoplasmic dynein 1 heavy chain 1 OS=Homo sapiens GN=DYNC1H1 PE=1 SV=5	28	99	532.1
Q9NR97	Toll-like receptor 8 OS=Homo sapiens GN=TLR8 PE=1 SV=1	28	24	119.8
Q4KMQ2	Anoctamin-6 OS=Homo sapiens GN=ANO6 PE=1 SV=2	28	21	106.1
Q8IXB1	DnaJ homolog subfamily C member 10 OS=Homo sapiens GN=DNAJC10 PE=1 SV=2	28	17	91
Q8IYB8	ATP-dependent RNA helicase SUPV3L1, mitochondrial OS=Homo sapiens GN=SUPV3L1 PE=1 SV=1	28	16	87.9
Q9Y3Z3	Deoxynucleoside triphosphate triphosphohydrolase SAMHD1 OS=Homo sapiens GN=SAMHD1 PE=1 SV=2	28	13	72.2
Q99805	Transmembrane 9 superfamily member 2 OS=Homo sapiens GN=TM9SF2 PE=1 SV=1	28	13	75.7
Q99442	Translocation protein SEC62 OS=Homo sapiens GN=SEC62 PE=1 SV=1	28	12	45.8
Q9NP92	39S ribosomal protein S30, mitochondrial OS=Homo sapiens GN=MRPS30 PE=1 SV=2	28	11	50.3
P30153	Serine/threonine-protein phosphatase 2A 65 kDa regulatory subunit A alpha isoform OS=Homo sapiens GN=PPP2R1A PE=1 SV=4	28	11	65.3

P23381	Tryptophan--tRNA ligase, cytoplasmic OS=Homo sapiens GN=WARS PE=1 SV=2	28	10	53.1
Q10567	AP-1 complex subunit beta-1 OS=Homo sapiens GN=AP1B1 PE=1 SV=2	28	9	104.6
O75787	Renin receptor OS=Homo sapiens GN=ATP6AP2 PE=1 SV=2	28	9	39
Q9NWS8	Required for meiotic nuclear division protein 1 homolog OS=Homo sapiens GN=RMND1 PE=1 SV=2	28	9	51.6
Q8N4Q0	Prostaglandin reductase 3 OS=Homo sapiens GN=ZADH2 PE=1 SV=1	28	8	40.1
Q06136	3-ketodihydrosphingosine reductase OS=Homo sapiens GN=KDSR PE=1 SV=1	28	7	36.2
Q96CB9	5-methylcytosine rRNA methyltransferase NSUN4 OS=Homo sapiens GN=NSUN4 PE=1 SV=2	28	7	43.1
Q9H857	5'-nucleotidase domain-containing protein 2 OS=Homo sapiens GN=NT5DC2 PE=1 SV=1	28	7	60.7
Q06323	Proteasome activator complex subunit 1 OS=Homo sapiens GN=PSME1 PE=1 SV=1	28	7	28.7
Q07960	Rho GTPase-activating protein 1 OS=Homo sapiens GN=ARHGAP1 PE=1 SV=1	28	7	50.4
B4E0Y9	Serine/threonine-protein kinase 26 OS=Homo sapiens GN=STK26 PE=1 SV=1	28	7	49.2
Q9Y6I9	Testis-expressed protein 264 OS=Homo sapiens GN=TEX264 PE=1 SV=1	28	7	34.2
O14657	Torsin-1B OS=Homo sapiens GN=TOR1B PE=1 SV=2	28	7	38
P57088	Transmembrane protein 33 OS=Homo sapiens GN=TMEM33 PE=1 SV=2	28	7	28
P00403	Cytochrome c oxidase subunit 2 OS=Homo sapiens GN=MT-CO2 PE=1 SV=1	28	6	25.5
O95976	Immunoglobulin superfamily member 6 OS=Homo sapiens GN=IGSF6 PE=2 SV=2	28	6	27
Q9Y2C4	Nuclease EXOG, mitochondrial OS=Homo sapiens GN=EXOG PE=1 SV=2	28	6	41.1
Q5T440	Putative transferase CAF17, mitochondrial OS=Homo sapiens GN=IBA57 PE=1 SV=1	28	6	38.1
Q5HYI8	Rab-like protein 3 OS=Homo sapiens GN=RABL3 PE=1 SV=1	28	6	26.4
P05026	Sodium/potassium-transporting ATPase subunit beta-1 OS=Homo sapiens GN=ATP1B1 PE=1 SV=1	28	6	35
Q9BUB7	Transmembrane protein 70, mitochondrial OS=Homo sapiens GN=TMEM70 PE=1 SV=2	28	6	29
Q9NRG7	Epimerase family protein SDR39U1 OS=Homo sapiens GN=SDR39U1 PE=1 SV=2	28	5	34.7
Q9NPA0	ER membrane protein complex subunit 7 OS=Homo sapiens GN=EMC7 PE=1 SV=1	28	5	26.5
Q9H2D1	Mitochondrial folate transporter/carrier OS=Homo sapiens GN=SLC25A32 PE=1 SV=2	28	5	35.4
Q6UW68	Transmembrane protein 205 OS=Homo sapiens GN=TMEM205 PE=1 SV=1	28	5	21.2
Q7Z7H8	39S ribosomal protein L10, mitochondrial OS=Homo sapiens GN=MRPL10 PE=1 SV=3	28	4	29.3
Q9NZZ3	Charged multivesicular body protein 5 OS=Homo sapiens GN=CHMP5 PE=1 SV=1	28	4	24.6
A0A0G2JMN6	Leukocyte immunoglobulin-like receptor subfamily A member 6 OS=Homo sapiens GN=LILRA6 PE=1 SV=1	28	4	54.3

Q4U2R6	39S ribosomal protein L51, mitochondrial OS=Homo sapiens GN=MRPL51 PE=1 SV=1	28	3	15.1
Q86YS6	Ras-related protein Rab-43 OS=Homo sapiens GN=RAB43 PE=1 SV=1	28	3	23.3
Q3ZAQ7	Vacuolar ATPase assembly integral membrane protein VMA21 OS=Homo sapiens GN=VMA21 PE=1 SV=1	28	3	11.3
P46939	Utrophin OS=Homo sapiens GN=UTRN PE=1 SV=2	27	68	394.2
Q9NZM1	Myoferlin OS=Homo sapiens GN=MYOF PE=1 SV=1	27	37	234.6
E9PEJ6	Phospholipid-transporting ATPase OS=Homo sapiens GN=ATP11A PE=1 SV=1	27	27	135.8
P53621	Coatomer subunit alpha OS=Homo sapiens GN=COPA PE=1 SV=2	27	26	138.3
Q9UIQ6	Leucyl-cystinyl aminopeptidase OS=Homo sapiens GN=LNPEP PE=1 SV=3	27	24	117.3
Q9Y2G3	Probable phospholipid-transporting ATPase IF OS=Homo sapiens GN=ATP11B PE=1 SV=2	27	23	134.1
Q9HD45	Transmembrane 9 superfamily member 3 OS=Homo sapiens GN=TM9SF3 PE=1 SV=2	27	19	67.8
P98171	Rho GTPase-activating protein 4 OS=Homo sapiens GN=ARHGAP4 PE=1 SV=2	27	18	105
P11678	Eosinophil peroxidase OS=Homo sapiens GN=EPX PE=1 SV=2	27	15	81
P16278	Beta-galactosidase OS=Homo sapiens GN=GLB1 PE=1 SV=2	27	13	76
Q7KYR7	Butyrophilin subfamily 2 member A1 OS=Homo sapiens GN=BTN2A1 PE=1 SV=3	27	13	59.6
O43175	D-3-phosphoglycerate dehydrogenase OS=Homo sapiens GN=PHGDH PE=1 SV=4	27	13	56.6
Q15758	Neutral amino acid transporter B(0) OS=Homo sapiens GN=SLC1A5 PE=1 SV=2	27	12	56.6
Q9P2R7	Succinate--CoA ligase [ADP-forming] subunit beta, mitochondrial OS=Homo sapiens GN=SUCLA2 PE=1 SV=3	27	12	50.3
Q9P0J1	[Pyruvate dehydrogenase [acetyl-transferring]]-phosphatase 1, mitochondrial OS=Homo sapiens GN=PDP1 PE=1 SV=3	27	11	61
Q9BT22	Chitobiosyldiphosphodolichol beta-mannosyltransferase OS=Homo sapiens GN=ALG1 PE=1 SV=2	27	11	52.5
P01730	T-cell surface glycoprotein CD4 OS=Homo sapiens GN=CD4 PE=1 SV=1	27	11	51.1
Q9NRZ7	1-acyl-sn-glycerol-3-phosphate acyltransferase gamma OS=Homo sapiens GN=AGPAT3 PE=1 SV=1	27	10	43.4
Q9NRG9	Aladin OS=Homo sapiens GN=AAAS PE=1 SV=1	27	10	59.5
Q96ER9	Coiled-coil domain-containing protein 51 OS=Homo sapiens GN=CCDC51 PE=1 SV=2	27	10	45.8
Q02790	Peptidyl-prolyl cis-trans isomerase FKBP4 OS=Homo sapiens GN=FKBP4 PE=1 SV=3	27	10	51.8
Q96BW9	Phosphatidate cytidylyltransferase, mitochondrial OS=Homo sapiens GN=TAMM41 PE=1 SV=2	27	10	51
M0QWZ7	Serine--tRNA ligase, mitochondrial OS=Homo sapiens GN=SARS2 PE=1 SV=1	27	9	58.1
Q9Y6W5	Wiskott-Aldrich syndrome protein family member 2 OS=Homo sapiens GN=WASF2 PE=1 SV=3	27	9	54.3
A0A087X2I1	26S protease regulatory subunit 10B OS=Homo sapiens GN=PSMC6 PE=1 SV=1	27	8	45.8

Q02543	60S ribosomal protein L18a OS=Homo sapiens GN=RPL18A PE=1 SV=2	27	8	20.7
Q12849	G-rich sequence factor 1 OS=Homo sapiens GN=GRSF1 PE=1 SV=3	27	8	53.1
Q9H078	Caseinolytic peptidase B protein homolog OS=Homo sapiens GN=CLPB PE=1 SV=1	27	7	78.7
Q92820	Gamma-glutamyl hydrolase OS=Homo sapiens GN=GGH PE=1 SV=2	27	7	35.9
Q9BQP7	Mitochondrial genome maintenance exonuclease 1 OS=Homo sapiens GN=MGME1 PE=1 SV=1	27	7	39.4
Q9Y619	Mitochondrial ornithine transporter 1 OS=Homo sapiens GN=SLC25A15 PE=1 SV=1	27	7	32.7
Q9BYN8	28S ribosomal protein S26, mitochondrial OS=Homo sapiens GN=MRPS26 PE=1 SV=1	27	6	24.2
Q9UBR2	Cathepsin Z OS=Homo sapiens GN=CTSZ PE=1 SV=1	27	6	33.8
Q13347	Eukaryotic translation initiation factor 3 subunit I OS=Homo sapiens GN=EIF3I PE=1 SV=1	27	6	36.5
Q330K2	NADH dehydrogenase (ubiquinone) complex I, assembly factor 6 OS=Homo sapiens GN=NDUFAF6 PE=1 SV=2	27	6	38.2
Q8WW59	SPRY domain-containing protein 4 OS=Homo sapiens GN=SPRYD4 PE=1 SV=2	27	6	23.1
O15120	1-acyl-sn-glycerol-3-phosphate acyltransferase beta OS=Homo sapiens GN=AGPAT2 PE=1 SV=1	27	5	30.9
Q5QPA5	39S ribosomal protein S18a, mitochondrial (Fragment) OS=Homo sapiens GN=MRPS18A PE=1 SV=9	27	5	29.6
P26373	60S ribosomal protein L13 OS=Homo sapiens GN=RPL13 PE=1 SV=4	27	5	24.2
P09601	Heme oxygenase 1 OS=Homo sapiens GN=HMOX1 PE=1 SV=1	27	5	32.8
Q969H8	Myeloid-derived growth factor OS=Homo sapiens GN=MYDGF PE=1 SV=1	27	5	18.8
A0A0J9YXF2	Paraoxonase 2, isoform CRA_a OS=Homo sapiens GN=PON2 PE=1 SV=1	27	5	41.5
Q9HBL7	Plasminogen receptor (KT) OS=Homo sapiens GN=PLGRKT PE=1 SV=1	27	5	17.2
D6RER5	Septin-11 OS=Homo sapiens GN=SEPT11 PE=1 SV=1	27	4	49.8
O15389	Sialic acid-binding Ig-like lectin 5 OS=Homo sapiens GN=SIGLEC5 PE=1 SV=1	27	4	60.7
O43752	Syntaxin-6 OS=Homo sapiens GN=STX6 PE=1 SV=1	27	4	29.2
Q9Y3U8	60S ribosomal protein L36 OS=Homo sapiens GN=RPL36 PE=1 SV=3	27	3	12.2
Q5STX8	Allograft inflammatory factor 1 (Fragment) OS=Homo sapiens GN=AIF1 PE=1 SV=1	27	3	18
A0A0U1RRH7	Histone H2A OS=Homo sapiens PE=3 SV=1	27	3	18.5
Q8N4V1	Membrane magnesium transporter 1 OS=Homo sapiens GN=MMGT1 PE=1 SV=1	27	2	14.7
Q13464	Rho-associated protein kinase 1 OS=Homo sapiens GN=ROCK1 PE=1 SV=1	26	28	158.1
P51828	Adenylate cyclase type 7 OS=Homo sapiens GN=ADCY7 PE=2 SV=1	26	19	120.2
Q9BXX0	EMILIN-2 OS=Homo sapiens GN=EMILIN2 PE=1 SV=3	26	18	115.6

Q86UT6	NLR family member X1 OS=Homo sapiens GN=NLRX1 PE=1 SV=1	26	18	107.5
Q7L576	Cytoplasmic FMR1-interacting protein 1 OS=Homo sapiens GN=CYFIP1 PE=1 SV=1	26	17	145.1
Q9H2K8	Serine/threonine-protein kinase TAO3 OS=Homo sapiens GN=TAOK3 PE=1 SV=2	26	17	105.3
R4GMU1	GDH/6PGL endoplasmic bifunctional protein OS=Homo sapiens GN=H6PD PE=1 SV=1	26	15	90.1
P06737	Glycogen phosphorylase, liver form OS=Homo sapiens GN=PYGL PE=1 SV=4	26	15	97.1
Q14644	Ras GTPase-activating protein 3 OS=Homo sapiens GN=RASA3 PE=1 SV=3	26	15	95.6
Q9NP80	Calcium-independent phospholipase A2-gamma OS=Homo sapiens GN=PNPLA8 PE=1 SV=1	26	14	88.4
P54136	Arginine--tRNA ligase, cytoplasmic OS=Homo sapiens GN=RARS PE=1 SV=2	26	12	75.3
O00429	Dynamin-1-like protein OS=Homo sapiens GN=DNM1L PE=1 SV=2	26	12	81.8
A0A0G2JJD3	HLA-B associated transcript 5, isoform CRA_b OS=Homo sapiens GN=ABHD16A PE=1 SV=1	26	12	67.5
P12956	X-ray repair cross-complementing protein 6 OS=Homo sapiens GN=XRCC6 PE=1 SV=2	26	11	69.8
P51648	Fatty aldehyde dehydrogenase OS=Homo sapiens GN=ALDH3A2 PE=1 SV=1	26	10	54.8
Q96S52	GPI transamidase component PIG-S OS=Homo sapiens GN=PIGS PE=1 SV=3	26	10	61.6
Q9UHX3	Adhesion G protein-coupled receptor E2 OS=Homo sapiens GN=ADGRE2 PE=1 SV=2	26	9	90.4
P45954	Short/branched chain specific acyl-CoA dehydrogenase, mitochondrial OS=Homo sapiens GN=ACADSB PE=1 SV=1	26	9	47.5
P22626	Heterogeneous nuclear ribonucleoproteins A2/B1 OS=Homo sapiens GN=HNRNPA2B1 PE=1 SV=2	26	8	37.4
Q9NXW2	DnaJ homolog subfamily B member 12 OS=Homo sapiens GN=DNAJB12 PE=1 SV=4	26	7	41.8
Q9BVV7	Mitochondrial import inner membrane translocase subunit Tim21 OS=Homo sapiens GN=TIMM21 PE=1 SV=1	26	7	28.2
Q96I59	Probable asparagine--tRNA ligase, mitochondrial OS=Homo sapiens GN=NARS2 PE=1 SV=3	26	7	54.1
A0A087WZM2	Ribonuclease T2 OS=Homo sapiens GN=RNASET2 PE=1 SV=1	26	7	34.9
Q9UGN4	CMRF35-like molecule 8 OS=Homo sapiens GN=CD300A PE=1 SV=2	26	6	33.2
Q969X5	Endoplasmic reticulum-Golgi intermediate compartment protein 1 OS=Homo sapiens GN=ERGIC1 PE=1 SV=1	26	6	32.6
P28676	Grancalcin OS=Homo sapiens GN=GCA PE=1 SV=2	26	6	24
Q8N386	Leucine-rich repeat-containing protein 25 OS=Homo sapiens GN=LRRC25 PE=1 SV=2	26	6	33.2
Q9BUP3	Oxidoreductase HTATIP2 OS=Homo sapiens GN=HTATIP2 PE=1 SV=2	26	6	27
Q8WWV3	Reticulon-4-interacting protein 1, mitochondrial OS=Homo sapiens GN=RTN4IP1 PE=1 SV=2	26	6	43.6
Q9UIB8	SLAM family member 5 OS=Homo sapiens GN=CD84 PE=1 SV=1	26	6	38.8
P84098	60S ribosomal protein L19 OS=Homo sapiens GN=RPL19 PE=1 SV=1	26	5	23.5

A0A087WUW9	ADP-ribosylation factor-like protein 15 OS=Homo sapiens GN=ARL15 PE=1 SV=1	26	5	22.9
Q9HB71	Calcyclin-binding protein OS=Homo sapiens GN=CACYBP PE=1 SV=2	26	5	26.2
A6NI73	Leukocyte immunoglobulin-like receptor subfamily A member 5 OS=Homo sapiens GN=LILRA5 PE=1 SV=1	26	5	32.7
Q5VWZ2	Lysophospholipase-like protein 1 OS=Homo sapiens GN=LYPLAL1 PE=1 SV=3	26	5	26.3
Q9UMS0	NFU1 iron-sulfur cluster scaffold homolog, mitochondrial OS=Homo sapiens GN=NFU1 PE=1 SV=2	26	5	28.4
Q9BQ69	O-acetyl-ADP-ribose deacetylase MACROD1 OS=Homo sapiens GN=MACROD1 PE=1 SV=2	26	5	35.5
A0A087WYT3	Prostaglandin E synthase 3 OS=Homo sapiens GN=PTGES3 PE=1 SV=1	26	5	19.1
Q92520	Protein FAM3C OS=Homo sapiens GN=FAM3C PE=1 SV=1	26	5	24.7
Q9BSH4	Translational activator of cytochrome c oxidase 1 OS=Homo sapiens GN=TACO1 PE=1 SV=1	26	5	32.5
P21291	Cysteine and glycine-rich protein 1 OS=Homo sapiens GN=CSRP1 PE=1 SV=3	26	4	20.6
G3V3D1	Epididymal secretory protein E1 (Fragment) OS=Homo sapiens GN=NPC2 PE=1 SV=1	26	4	23.7
P08708	40S ribosomal protein S17 OS=Homo sapiens GN=RPS17 PE=1 SV=2	26	3	15.5
P60866	40S ribosomal protein S20 OS=Homo sapiens GN=RPS20 PE=1 SV=1	26	3	13.4
Q4VC31	Coiled-coil domain-containing protein 58 OS=Homo sapiens GN=CCDC58 PE=1 SV=1	26	3	16.6
I3L0E3	HCG1984214, isoform CRA_a OS=Homo sapiens GN=hCG_1984214 PE=4 SV=1	26	3	25.7
Q4G0I0	Protein CCSMST1 OS=Homo sapiens GN=CCSMST1 PE=2 SV=1	26	3	15
A8MWD9	Putative small nuclear ribonucleoprotein G-like protein 15 OS=Homo sapiens GN=SNRPGP15 PE=5 SV=2	26	2	8.5
Q14643	Inositol 1,4,5-trisphosphate receptor type 1 OS=Homo sapiens GN=ITPR1 PE=1 SV=3	25	49	313.7
Q8TEM1	Nuclear pore membrane glycoprotein 210 OS=Homo sapiens GN=NUP210 PE=1 SV=3	25	31	205
P19440	Gamma-glutamyltranspeptidase 1 OS=Homo sapiens GN=GGT1 PE=1 SV=2	25	14	61.4
P32942	Intercellular adhesion molecule 3 OS=Homo sapiens GN=ICAM3 PE=1 SV=2	25	14	59.5
Q96AQ6	Pre-B-cell leukemia transcription factor-interacting protein 1 OS=Homo sapiens GN=PBXIP1 PE=1 SV=1	25	14	80.6
P11940	Polyadenylate-binding protein 1 OS=Homo sapiens GN=PABPC1 PE=1 SV=2	25	13	70.6
Q6NUQ4	Transmembrane protein 214 OS=Homo sapiens GN=TMEM214 PE=1 SV=2	25	13	77.1
P31939	Bifunctional purine biosynthesis protein PURH OS=Homo sapiens GN=ATIC PE=1 SV=3	25	12	64.6
P17813	Endoglin OS=Homo sapiens GN=ENG PE=1 SV=2	25	12	70.5
P07099	Epoxide hydrolase 1 OS=Homo sapiens GN=EPHX1 PE=1 SV=1	25	11	52.9
Q92542	Nicastrin OS=Homo sapiens GN=NCSTN PE=1 SV=2	25	11	78.4

O43242	26S proteasome non-ATPase regulatory subunit 3 OS=Homo sapiens GN=PSMD3 PE=1 SV=2	25	10	60.9
Q15437	Protein transport protein Sec23B OS=Homo sapiens GN=SEC23B PE=1 SV=2	25	10	86.4
P48643	T-complex protein 1 subunit epsilon OS=Homo sapiens GN=CCT5 PE=1 SV=1	25	10	59.6
P23526	Adenosylhomocysteinase OS=Homo sapiens GN=AHCY PE=1 SV=4	25	9	47.7
Q9H3K2	Growth hormone-inducible transmembrane protein OS=Homo sapiens GN=GHITM PE=1 SV=2	25	9	37.2
Q6NVY1	3-hydroxyisobutyryl-CoA hydrolase, mitochondrial OS=Homo sapiens GN=HIBCH PE=1 SV=2	25	8	43.5
Q9H553	Alpha-1,3/1,6-mannosyltransferase ALG2 OS=Homo sapiens GN=ALG2 PE=1 SV=1	25	8	47.1
P17050	Alpha-N-acetylgalactosaminidase OS=Homo sapiens GN=NAGA PE=1 SV=2	25	8	46.5
P61978	Heterogeneous nuclear ribonucleoprotein K OS=Homo sapiens GN=HNRNPK PE=1 SV=1	25	8	50.9
P42785	Lysosomal Pro-X carboxypeptidase OS=Homo sapiens GN=PRCP PE=1 SV=1	25	8	55.8
Q9Y336	Sialic acid-binding Ig-like lectin 9 OS=Homo sapiens GN=SIGLEC9 PE=1 SV=2	25	8	50.1
P22830	Ferrochelatase, mitochondrial OS=Homo sapiens GN=FECH PE=1 SV=2	25	7	47.8
Q99571	P2X purinoceptor 4 OS=Homo sapiens GN=P2RX4 PE=1 SV=2	25	7	43.3
Q92575	UBX domain-containing protein 4 OS=Homo sapiens GN=UBXN4 PE=1 SV=2	25	7	56.7
Q9NX20	39S ribosomal protein L16, mitochondrial OS=Homo sapiens GN=MRPL16 PE=1 SV=1	25	6	28.4
J3KS52	CMRF35-like molecule 1 OS=Homo sapiens GN=CD300LF PE=1 SV=1	25	6	33.8
Q9NZJ6	Ubiquinone biosynthesis O-methyltransferase, mitochondrial OS=Homo sapiens GN=COQ3 PE=1 SV=3	25	6	41
Q8NBZ7	UDP-glucuronic acid decarboxylase 1 OS=Homo sapiens GN=UXS1 PE=1 SV=1	25	6	47.5
J3QTA6	MICOS complex subunit OS=Homo sapiens GN=CHCHD6 PE=1 SV=1	25	5	28.8
P49720	Proteasome subunit beta type-3 OS=Homo sapiens GN=PSMB3 PE=1 SV=2	25	5	22.9
P32970	CD70 antigen OS=Homo sapiens GN=CD70 PE=1 SV=2	25	4	21.1
Q16854	Deoxyguanosine kinase, mitochondrial OS=Homo sapiens GN=DGUOK PE=1 SV=2	25	4	32
O43402	ER membrane protein complex subunit 8 OS=Homo sapiens GN=EMC8 PE=1 SV=1	25	4	23.8
O14494	Phospholipid phosphatase 1 OS=Homo sapiens GN=PLPP1 PE=1 SV=1	25	4	32.1
O15498	Synaptobrevin homolog YKT6 OS=Homo sapiens GN=YKT6 PE=1 SV=1	25	4	22.4
E9PR17	CD59 glycoprotein OS=Homo sapiens GN=CD59 PE=1 SV=1	25	3	14.5
O43324	Eukaryotic translation elongation factor 1 epsilon-1 OS=Homo sapiens GN=EEF1E1 PE=1 SV=1	25	3	19.8
B8ZZA8	Glutaminase kidney isoform, mitochondrial OS=Homo sapiens GN=GLS PE=1 SV=1	25	3	18.6

P61018	Ras-related protein Rab-4B OS=Homo sapiens GN=RAB4B PE=1 SV=1	25	3	23.6
S4R2Z7	39S ribosomal protein L42, mitochondrial OS=Homo sapiens GN=MRPL42 PE=1 SV=1	25	2	17.9
Q9NRR8	CDC42 small effector protein 1 OS=Homo sapiens GN=CDC42SE1 PE=1 SV=1	25	2	8.9
Q9BW72	HIG1 domain family member 2A, mitochondrial OS=Homo sapiens GN=HIGD2A PE=1 SV=1	25	2	11.5
P12314	High affinity immunoglobulin gamma Fc receptor I OS=Homo sapiens GN=FCGR1A PE=1 SV=2	25	2	42.6
Q8NI22	Multiple coagulation factor deficiency protein 2 OS=Homo sapiens GN=MCFD2 PE=1 SV=1	25	2	16.4
Q8TBQ9	Protein kish-A OS=Homo sapiens GN=TMEM167A PE=1 SV=1	25	2	8.1
Q86UP2	Kinectin OS=Homo sapiens GN=KTN1 PE=1 SV=1	24	25	156.2
A0A0G2JH68	Protein diaphanous homolog 1 OS=Homo sapiens GN=DIAPH1 PE=1 SV=1	24	23	141.3
Q9H7F0	Probable cation-transporting ATPase 13A3 OS=Homo sapiens GN=ATP13A3 PE=1 SV=4	24	19	138
Q86UE4	Protein LYRIC OS=Homo sapiens GN=MTDH PE=1 SV=2	24	13	63.8
P11169	Solute carrier family 2, facilitated glucose transporter member 3 OS=Homo sapiens GN=SLC2A3 PE=1 SV=1	24	12	53.9
Q9ULC5	Long-chain-fatty-acid--CoA ligase 5 OS=Homo sapiens GN=ACSL5 PE=1 SV=1	24	11	75.9
O15269	Serine palmitoyltransferase 1 OS=Homo sapiens GN=SPTLC1 PE=1 SV=1	24	11	52.7
P50995	Annexin A11 OS=Homo sapiens GN=ANXA11 PE=1 SV=1	24	10	54.4
Q02252	Methylmalonate-semialdehyde dehydrogenase [acylating], mitochondrial OS=Homo sapiens GN=ALDH6A1 PE=1 SV=2	24	10	57.8
P43250	G protein-coupled receptor kinase 6 OS=Homo sapiens GN=GRK6 PE=1 SV=2	24	9	65.9
Q96N66	Lysophospholipid acyltransferase 7 OS=Homo sapiens GN=MBOAT7 PE=1 SV=2	24	9	52.7
Q96G23	Ceramide synthase 2 OS=Homo sapiens GN=CERS2 PE=1 SV=1	24	8	44.8
Q9NXH8	Torsin-4A OS=Homo sapiens GN=TOR4A PE=1 SV=2	24	8	46.9
A0A087WUL9	26S proteasome non-ATPase regulatory subunit 13 OS=Homo sapiens GN=PSMD13 PE=1 SV=1	24	7	42.7
Q96EY1	DnaJ homolog subfamily A member 3, mitochondrial OS=Homo sapiens GN=DNAJA3 PE=1 SV=2	24	7	52.5
Q3SXM5	Inactive hydroxysteroid dehydrogenase-like protein 1 OS=Homo sapiens GN=HSDL1 PE=1 SV=3	24	7	37
P11172	Uridine 5'-monophosphate synthase OS=Homo sapiens GN=UMPS PE=1 SV=1	24	7	52.2
M0QYS1	60S ribosomal protein L13a (Fragment) OS=Homo sapiens GN=RPL13A PE=1 SV=2	24	6	24.2
Q9HA72	Calcium homeostasis modulator protein 2 OS=Homo sapiens GN=CALHM2 PE=2 SV=1	24	6	36.2
Q13137	Calcium-binding and coiled-coil domain-containing protein 2 OS=Homo sapiens GN=CALCOCO2 PE=1 SV=1	24	6	52.2
A0A0G2JPR9	Leukocyte immunoglobulin-like receptor subfamily A member 6 OS=Homo sapiens GN=LILRA6 PE=1 SV=1	24	6	71

Q8IX19	Mast cell-expressed membrane protein 1 OS=Homo sapiens GN=MCEMP1 PE=1 SV=1	24	6	21.2
P25788	Proteasome subunit alpha type-3 OS=Homo sapiens GN=PSMA3 PE=1 SV=2	24	6	28.4
O15127	Secretory carrier-associated membrane protein 2 OS=Homo sapiens GN=SCAMP2 PE=1 SV=2	24	6	36.6
Q9H7M9	V-type immunoglobulin domain-containing suppressor of T-cell activation OS=Homo sapiens GN=C10orf54 PE=1 SV=3	24	6	33.9
P50914	60S ribosomal protein L14 OS=Homo sapiens GN=RPL14 PE=1 SV=4	24	5	23.4
O14561	Acyl carrier protein, mitochondrial OS=Homo sapiens GN=NDUFAB1 PE=1 SV=3	24	5	17.4
Q9H488	GDP-fucose protein O-fucosyltransferase 1 OS=Homo sapiens GN=POFUT1 PE=1 SV=1	24	5	43.9
J3KQE5	GTP-binding nuclear protein Ran (Fragment) OS=Homo sapiens GN=RAN PE=1 SV=1	24	5	26.8
A0A0A0MSW4	Phosphatidylinositol transfer protein beta isoform OS=Homo sapiens GN=PITPNB PE=1 SV=1	24	5	31.6
Q9Y279	V-set and immunoglobulin domain-containing protein 4 OS=Homo sapiens GN=VSIG4 PE=1 SV=1	24	5	44
Q9BSH5	Haloacid dehalogenase-like hydrolase domain-containing protein 3 OS=Homo sapiens GN=HDHD3 PE=1 SV=1	24	4	28
P12004	Proliferating cell nuclear antigen OS=Homo sapiens GN=PCNA PE=1 SV=1	24	4	28.8
P20618	Proteasome subunit beta type-1 OS=Homo sapiens GN=PSMB1 PE=1 SV=2	24	4	26.5
Q8TCC3	39S ribosomal protein L30, mitochondrial OS=Homo sapiens GN=MRPL30 PE=1 SV=1	24	3	18.5
P10415	Apoptosis regulator Bcl-2 OS=Homo sapiens GN=BCL2 PE=1 SV=2	24	3	26.2
A0A024R161	Guanine nucleotide-binding protein subunit gamma OS=Homo sapiens GN=DNAJC25-GNG10 PE=3 SV=1	24	3	16.5
Q9Y3D6	Mitochondrial fission 1 protein OS=Homo sapiens GN=FIS1 PE=1 SV=2	24	3	16.9
P37108	Signal recognition particle 14 kDa protein OS=Homo sapiens GN=SRP14 PE=1 SV=2	24	3	14.6
Q9P2X0	Dolichol-phosphate mannosyltransferase subunit 3 OS=Homo sapiens GN=DPM3 PE=1 SV=2	24	2	10.1
I3L0M9	Elongin-B (Fragment) OS=Homo sapiens GN=ELOB PE=1 SV=1	24	2	15.6
Q96KF7	Small integral membrane protein 8 OS=Homo sapiens GN=SMIM8 PE=1 SV=2	24	2	11.1
Q9P0S9	Transmembrane protein 14C OS=Homo sapiens GN=TMEM14C PE=1 SV=1	24	2	11.6
P55160	Nck-associated protein 1-like OS=Homo sapiens GN=NCKAP1L PE=1 SV=3	23	19	128.1
Q8WWI5	Choline transporter-like protein 1 OS=Homo sapiens GN=SLC44A1 PE=1 SV=1	23	15	73.3
Q14974	Importin subunit beta-1 OS=Homo sapiens GN=KPNB1 PE=1 SV=2	23	15	97.1
O43776	Asparagine--tRNA ligase, cytoplasmic OS=Homo sapiens GN=NARS PE=1 SV=1	23	10	62.9
P08236	Beta-glucuronidase OS=Homo sapiens GN=GUSB PE=1 SV=2	23	10	74.7
P11117	Lysosomal acid phosphatase OS=Homo sapiens GN=ACP2 PE=1 SV=3	23	9	48.3

O95497	Pantetheinase OS=Homo sapiens GN=VNN1 PE=1 SV=2	23	9	57
Q969Z0	Protein TBRG4 OS=Homo sapiens GN=TBRG4 PE=1 SV=1	23	9	70.7
O75381	Peroxisomal membrane protein PEX14 OS=Homo sapiens GN=PEX14 PE=1 SV=1	23	8	41.2
P50452	Serpin B8 OS=Homo sapiens GN=SERPINB8 PE=1 SV=2	23	8	42.7
P82914	28S ribosomal protein S15, mitochondrial OS=Homo sapiens GN=MRPS15 PE=1 SV=1	23	7	29.8
P06280	Alpha-galactosidase A OS=Homo sapiens GN=GLA PE=1 SV=1	23	7	48.7
Q7Z4H8	KDEL motif-containing protein 2 OS=Homo sapiens GN=KDELC2 PE=1 SV=2	23	7	58.5
P20138	Myeloid cell surface antigen CD33 OS=Homo sapiens GN=CD33 PE=1 SV=2	23	7	39.8
Q8NC51	Plasminogen activator inhibitor 1 RNA-binding protein OS=Homo sapiens GN=SERBP1 PE=1 SV=2	23	7	44.9
Q9NUQ7	Ufm1-specific protease 2 OS=Homo sapiens GN=UFSP2 PE=1 SV=3	23	7	53.2
O95498	Vascular non-inflammatory molecule 2 OS=Homo sapiens GN=VNN2 PE=1 SV=3	23	7	58.5
O43681	ATPase ASNA1 OS=Homo sapiens GN=ASNA1 PE=1 SV=2	23	6	38.8
Q99747	Gamma-soluble NSF attachment protein OS=Homo sapiens GN=NAPG PE=1 SV=1	23	6	34.7
Q9NXS2	Glutaminyl-peptide cyclotransferase-like protein OS=Homo sapiens GN=QPCTL PE=1 SV=2	23	6	42.9
Q8WWC4	m-AAA protease-interacting protein 1, mitochondrial OS=Homo sapiens GN=MAIP1 PE=1 SV=1	23	6	32.5
P50454	Serpin H1 OS=Homo sapiens GN=SERPINH1 PE=1 SV=2	23	6	46.4
Q8N0X4	Citrate lyase subunit beta-like protein, mitochondrial OS=Homo sapiens GN=CLYBL PE=1 SV=2	23	5	37.3
A0A087WT12	Glutathione peroxidase OS=Homo sapiens GN=GPX4 PE=1 SV=1	23	5	26.9
Q15526	Surfeit locus protein 1 OS=Homo sapiens GN=SURF1 PE=1 SV=1	23	5	33.3
Q8NG11	Tetraspanin-14 OS=Homo sapiens GN=TSPAN14 PE=1 SV=1	23	5	30.7
Q9Y320	Thioredoxin-related transmembrane protein 2 OS=Homo sapiens GN=TMX2 PE=1 SV=1	23	5	34
A0A087X266	Transmembrane protein 120A OS=Homo sapiens GN=TMEM120A PE=1 SV=1	23	5	42.9
P20333	Tumor necrosis factor receptor superfamily member 1B OS=Homo sapiens GN=TNFRSF1B PE=1 SV=3	23	5	48.3
P46776	60S ribosomal protein L27a OS=Homo sapiens GN=RPL27A PE=1 SV=2	23	4	16.6
Q13155	Aminoacyl tRNA synthase complex-interacting multifunctional protein 2 OS=Homo sapiens GN=AIMP2 PE=1 SV=2	23	4	35.3
M0QXB4	Coatomer protein complex, subunit epsilon, isoform CRA_g OS=Homo sapiens GN=COPE PE=1 SV=1	23	4	36.9
Q9NQX7	Integral membrane protein 2C OS=Homo sapiens GN=ITM2C PE=1 SV=1	23	4	30.2
Q15691	Microtubule-associated protein RP/EB family member 1 OS=Homo sapiens GN=MAPRE1 PE=1 SV=3	23	4	30

Q15366	Poly(rC)-binding protein 2 OS=Homo sapiens GN=PCBP2 PE=1 SV=1	23	4	38.6
Q9NX57	Ras-related protein Rab-20 OS=Homo sapiens GN=RAB20 PE=1 SV=1	23	4	26.3
Q5JU69	Torsin-2A OS=Homo sapiens GN=TOR2A PE=2 SV=1	23	4	35.7
A0A0B4J2C3	Translationally-controlled tumor protein OS=Homo sapiens GN=TPT1 PE=1 SV=1	23	4	22.6
Q86T03	Type 1 phosphatidylinositol 4,5-bisphosphate 4-phosphatase OS=Homo sapiens GN=TMEM55B PE=1 SV=1	23	4	29.5
O95372	Acyl-protein thioesterase 2 OS=Homo sapiens GN=LYPLA2 PE=1 SV=1	23	3	24.7
Q01151	CD83 antigen OS=Homo sapiens GN=CD83 PE=1 SV=1	23	3	23
Q8IUX1	Complex I assembly factor TMEM126B, mitochondrial OS=Homo sapiens GN=TMEM126B PE=1 SV=2	23	3	25.9
P56537	Eukaryotic translation initiation factor 6 OS=Homo sapiens GN=EIF6 PE=1 SV=1	23	3	26.6
Q9Y4H4	G-protein-signaling modulator 3 OS=Homo sapiens GN=GPSM3 PE=1 SV=1	23	3	17.9
B1ANH2	Guanylate kinase (Fragment) OS=Homo sapiens GN=GUK1 PE=1 SV=1	23	3	27.6
P62072	Mitochondrial import inner membrane translocase subunit Tim10 OS=Homo sapiens GN=TIMM10 PE=1 SV=1	23	3	10.3
O43557	Tumor necrosis factor ligand superfamily member 14 OS=Homo sapiens GN=TNFSF14 PE=1 SV=2	23	3	26.3
P63220	40S ribosomal protein S21 OS=Homo sapiens GN=RPS21 PE=1 SV=1	23	2	9.1
O95164	Ubiquitin-like protein 3 OS=Homo sapiens GN=UBL3 PE=1 SV=1	23	2	13.1
Q15149	Plectin OS=Homo sapiens GN=PLEC PE=1 SV=3	22	79	531.5
Q07954	Prolow-density lipoprotein receptor-related protein 1 OS=Homo sapiens GN=LRP1 PE=1 SV=2	22	73	504.3
Q92673	Sortilin-related receptor OS=Homo sapiens GN=SORL1 PE=1 SV=2	22	33	248.3
A3KMH1	von Willebrand factor A domain-containing protein 8 OS=Homo sapiens GN=VWA8 PE=1 SV=2	22	33	214.7
P35606	Coatomer subunit beta' OS=Homo sapiens GN=COPB2 PE=1 SV=2	22	15	102.4
P22102	Trifunctional purine biosynthetic protein adenosine-3 OS=Homo sapiens GN=GART PE=1 SV=1	22	15	107.7
A0A1B0GUZ7	Protein EFR3 homolog A OS=Homo sapiens GN=EFR3A PE=1 SV=1	22	13	96.1
A0A087WXI0	Ras-related protein Rab-44 OS=Homo sapiens GN=RAB44 PE=1 SV=1	22	13	110.8
Q7L1W4	Volume-regulated anion channel subunit LRRC8D OS=Homo sapiens GN=LRRC8D PE=1 SV=1	22	13	98.1
Q9BZF1	Oxysterol-binding protein-related protein 8 OS=Homo sapiens GN=OSBPL8 PE=1 SV=3	22	12	101.1
Q8WVX9	Fatty acyl-CoA reductase 1 OS=Homo sapiens GN=FAR1 PE=1 SV=1	22	11	59.3
Q99572	P2X purinoceptor 7 OS=Homo sapiens GN=P2RX7 PE=1 SV=4	22	10	68.5
O96005	Cleft lip and palate transmembrane protein 1 OS=Homo sapiens GN=CLPTM1 PE=1 SV=1	22	9	76

P15848	Arylsulfatase B OS=Homo sapiens GN=ARSB PE=1 SV=1	22	8	59.6
Q96CU9	FAD-dependent oxidoreductase domain-containing protein 1 OS=Homo sapiens GN=FOXRED1 PE=1 SV=2	22	8	53.8
Q8N2G8	GH3 domain-containing protein OS=Homo sapiens GN=GHDC PE=1 SV=2	22	8	57.5
P36897	TGF-beta receptor type-1 OS=Homo sapiens GN=TGFBR1 PE=1 SV=1	22	8	55.9
Q92604	Acyl-CoA:lysophosphatidylglycerol acyltransferase 1 OS=Homo sapiens GN=LPGAT1 PE=1 SV=1	22	7	43.1
Q10469	Alpha-1,6-mannosyl-glycoprotein 2-beta-N-acetylglucosaminyltransferase OS=Homo sapiens GN=MGAT2 PE=1 SV=1	22	7	51.5
Q9Y679	Ancient ubiquitous protein 1 OS=Homo sapiens GN=AUP1 PE=1 SV=1	22	7	53
O94973	AP-2 complex subunit alpha-2 OS=Homo sapiens GN=AP2A2 PE=1 SV=2	22	7	103.9
P49590	Probable histidine--tRNA ligase, mitochondrial OS=Homo sapiens GN=HARS2 PE=1 SV=1	22	7	56.9
Q00765	Receptor expression-enhancing protein 5 OS=Homo sapiens GN=REEP5 PE=1 SV=3	22	7	21.5
Q9UI08	Ena/VASP-like protein OS=Homo sapiens GN=EVL PE=1 SV=2	22	6	44.6
Q96C36	Pyrroline-5-carboxylate reductase 2 OS=Homo sapiens GN=PYCR2 PE=1 SV=1	22	6	33.6
Q96LW7	Caspase recruitment domain-containing protein 19 OS=Homo sapiens GN=CARD19 PE=1 SV=1	22	5	25.6
Q9NWW5	Ceroid-lipofuscinosis neuronal protein 6 OS=Homo sapiens GN=CLN6 PE=1 SV=1	22	5	35.9
Q9H1A3	Methyltransferase-like protein 9 OS=Homo sapiens GN=METTL9 PE=2 SV=1	22	5	36.5
Q13445	Transmembrane emp24 domain-containing protein 1 OS=Homo sapiens GN=TMED1 PE=1 SV=1	22	5	25.2
Q6PI78	Transmembrane protein 65 OS=Homo sapiens GN=TMEM65 PE=1 SV=2	22	5	25.5
P82912	28S ribosomal protein S11, mitochondrial OS=Homo sapiens GN=MRPS11 PE=1 SV=2	22	4	20.6
Q9BTT0	Acidic leucine-rich nuclear phosphoprotein 32 family member E OS=Homo sapiens GN=ANP32E PE=1 SV=1	22	4	30.7
Q9NX47	E3 ubiquitin-protein ligase MARCH5 OS=Homo sapiens GN=MARCH5 PE=1 SV=1	22	4	31.2
P37235	Hippocalcin-like protein 1 OS=Homo sapiens GN=HPCAL1 PE=1 SV=3	22	4	22.3
P13761	HLA class II histocompatibility antigen, DRB1-7 beta chain OS=Homo sapiens GN=HLA-DRB1 PE=1 SV=1	22	4	29.8
Q96DA6	Mitochondrial import inner membrane translocase subunit TIM14 OS=Homo sapiens GN=DNAJC19 PE=1 SV=3	22	4	12.5
Q92982	Ninjurin-1 OS=Homo sapiens GN=NINJ1 PE=1 SV=2	22	4	16.3
P09668	Pro-cathepsin H OS=Homo sapiens GN=CTSH PE=1 SV=4	22	4	37.4
P17152	Transmembrane protein 11, mitochondrial OS=Homo sapiens GN=TMEM11 PE=1 SV=1	22	4	21.5
Q12981	Vesicle transport protein SEC20 OS=Homo sapiens GN=BNIP1 PE=1 SV=3	22	4	26.1
O95292	Vesicle-associated membrane protein-associated protein B/C OS=Homo sapiens GN=VAPB PE=1 SV=3	22	4	27.2

Q8N0U8	Vitamin K epoxide reductase complex subunit 1-like protein 1 OS=Homo sapiens GN=VKORC1L1 PE=1 SV=2	22	4	19.8
Q9Y478	5'-AMP-activated protein kinase subunit beta-1 OS=Homo sapiens GN=PRKAB1 PE=1 SV=4	22	3	30.4
Q9ULY5	C-type lectin domain family 4 member E OS=Homo sapiens GN=CLEC4E PE=1 SV=1	22	3	25.1
Q5J8M3	ER membrane protein complex subunit 4 OS=Homo sapiens GN=EMC4 PE=1 SV=2	22	3	20.1
P34810	Macrosialin OS=Homo sapiens GN=CD68 PE=1 SV=2	22	3	37.4
A0A0B4J1S4	Selenoprotein F OS=Homo sapiens GN=SELENOF PE=1 SV=1	22	3	18
A0A087WVA1	Selenoprotein T OS=Homo sapiens GN=SELENOT PE=1 SV=1	22	3	22.3
A6NMH8	Tetraspanin OS=Homo sapiens GN=CD81 PE=1 SV=1	22	3	29.8
B1AKR6	Dynein light chain roadblock-type 1 OS=Homo sapiens GN=DYNLRB1 PE=1 SV=1	22	2	16.2
P60468	Protein transport protein Sec61 subunit beta OS=Homo sapiens GN=SEC61B PE=1 SV=2	22	2	10
P49458	Signal recognition particle 9 kDa protein OS=Homo sapiens GN=SRP9 PE=1 SV=2	22	2	10.1
Q99757	Thioredoxin, mitochondrial OS=Homo sapiens GN=TXN2 PE=1 SV=2	22	2	18.4
Q9H0R3	Transmembrane protein 222 OS=Homo sapiens GN=TMEM222 PE=1 SV=2	22	2	23.2
D6RCM8	Tumor necrosis factor alpha-induced protein 8 OS=Homo sapiens GN=TNFAIP8 PE=1 SV=1	22	2	23.2
O75379	Vesicle-associated membrane protein 4 OS=Homo sapiens GN=VAMP4 PE=1 SV=2	22	2	16.4
Q5JRA6	Melanoma inhibitory activity protein 3 OS=Homo sapiens GN=MIA3 PE=1 SV=1	21	30	213.6
O60486	Plexin-C1 OS=Homo sapiens GN=PLXNC1 PE=1 SV=1	21	23	175.6
P23458	Tyrosine-protein kinase JAK1 OS=Homo sapiens GN=JAK1 PE=1 SV=2	21	18	133.2
P33176	Kinesin-1 heavy chain OS=Homo sapiens GN=KIF5B PE=1 SV=1	21	15	109.6
A2RUF3	SLIT-ROBO Rho GTPase activating protein 2 OS=Homo sapiens GN=SRGAP2 PE=1 SV=1	21	15	120.8
P56192	Methionine--tRNA ligase, cytoplasmic OS=Homo sapiens GN=MARS PE=1 SV=2	21	14	101.1
P22314	Ubiquitin-like modifier-activating enzyme 1 OS=Homo sapiens GN=UBA1 PE=1 SV=3	21	14	117.8
P51798	H(+)/Cl(-) exchange transporter 7 OS=Homo sapiens GN=CLCN7 PE=1 SV=2	21	12	88.6
P78536	Disintegrin and metalloproteinase domain-containing protein 17 OS=Homo sapiens GN=ADAM17 PE=1 SV=1	21	11	93
Q9Y639	Neuroplastin OS=Homo sapiens GN=NPTN PE=1 SV=2	21	11	44.4
Q14739	Lamin-B receptor OS=Homo sapiens GN=LBR PE=1 SV=2	21	10	70.7
Q9H300	Presenilins-associated rhomboid-like protein, mitochondrial OS=Homo sapiens GN=PARL PE=1 SV=2	21	10	42.2
Q9BXP2	Solute carrier family 12 member 9 OS=Homo sapiens GN=SLC12A9 PE=1 SV=1	21	10	96

Q03169	Tumor necrosis factor alpha-induced protein 2 OS=Homo sapiens GN=TNFAIP2 PE=2 SV=2	21	10	72.6
Q01813	ATP-dependent 6-phosphofructokinase, platelet type OS=Homo sapiens GN=PFKP PE=1 SV=2	21	8	85.5
A0A087WU53	Magnesium transporter protein 1 OS=Homo sapiens GN=MAGT1 PE=1 SV=1	21	8	41.5
P21953	2-oxoisovalerate dehydrogenase subunit beta, mitochondrial OS=Homo sapiens GN=BCKDHB PE=1 SV=2	21	7	43.1
Q9Y376	Calcium-binding protein 39 OS=Homo sapiens GN=CAB39 PE=1 SV=1	21	7	39.8
X6R8A1	Carboxypeptidase OS=Homo sapiens GN=CTSA PE=1 SV=1	21	7	56.2
O60861	Growth arrest-specific protein 7 OS=Homo sapiens GN=GAS7 PE=1 SV=3	21	7	54.3
Q8TCT9	Minor histocompatibility antigen H13 OS=Homo sapiens GN=HM13 PE=1 SV=1	21	7	41.5
P41218	Myeloid cell nuclear differentiation antigen OS=Homo sapiens GN=MNDA PE=1 SV=1	21	7	45.8
Q8TD55	Pleckstrin homology domain-containing family O member 2 OS=Homo sapiens GN=PLEKHO2 PE=1 SV=1	21	7	53.3
P62195	26S protease regulatory subunit 8 OS=Homo sapiens GN=PSMC5 PE=1 SV=1	21	6	45.6
P21589	5'-nucleotidase OS=Homo sapiens GN=NT5E PE=1 SV=1	21	6	63.3
P28907	ADP-ribosyl cyclase/cyclic ADP-ribose hydrolase 1 OS=Homo sapiens GN=CD38 PE=1 SV=2	21	6	34.3
P00966	Argininosuccinate synthase OS=Homo sapiens GN=ASS1 PE=1 SV=2	21	6	46.5
Q96CX2	BTB/POZ domain-containing protein KCTD12 OS=Homo sapiens GN=KCTD12 PE=1 SV=1	21	6	35.7
Q16581	C3a anaphylatoxin chemotactic receptor OS=Homo sapiens GN=C3AR1 PE=1 SV=2	21	6	53.8
Q9BWH2	FUN14 domain-containing protein 2 OS=Homo sapiens GN=FUNDC2 PE=1 SV=2	21	6	20.7
P12268	Inosine-5'-monophosphate dehydrogenase 2 OS=Homo sapiens GN=IMPDH2 PE=1 SV=2	21	6	55.8
P12931	Proto-oncogene tyrosine-protein kinase Src OS=Homo sapiens GN=SRC PE=1 SV=3	21	6	59.8
A0A0A6YY92	Adenylosuccinate lyase OS=Homo sapiens GN=ADSL PE=1 SV=1	21	5	56.2
P78417	Glutathione S-transferase omega-1 OS=Homo sapiens GN=GSTO1 PE=1 SV=2	21	5	27.5
B5MDU6	Lipid droplet-associated hydrolase OS=Homo sapiens GN=LDAH PE=1 SV=1	21	5	42.6
Q15800	Methylsterol monooxygenase 1 OS=Homo sapiens GN=MSMO1 PE=1 SV=1	21	5	35.2
H0Y886	NADH dehydrogenase [ubiquinone] 1 beta subcomplex subunit 5, mitochondrial (Fragment) OS=Homo sapiens GN=NDUFB5 PE=1 SV=1	21	5	23.5
Q96MH6	Transmembrane protein 68 OS=Homo sapiens GN=TMEM68 PE=2 SV=2	21	5	37.4
P62913	60S ribosomal protein L11 OS=Homo sapiens GN=RPL11 PE=1 SV=2	21	4	20.2
P27695	DNA-(apurinic or apyrimidinic site) lyase OS=Homo sapiens GN=APEX1 PE=1 SV=2	21	4	35.5
Q8WU67	Phospholipase ABHD3 OS=Homo sapiens GN=ABHD3 PE=1 SV=2	21	4	46

Q9BT09	Protein canopy homolog 3 OS=Homo sapiens GN=CNPY3 PE=1 SV=1	21	4	30.7
O00764	Pyridoxal kinase OS=Homo sapiens GN=PDXK PE=1 SV=1	21	4	35.1
P62851	40S ribosomal protein S25 OS=Homo sapiens GN=RPS25 PE=1 SV=1	21	3	13.7
P56377	AP-1 complex subunit sigma-2 OS=Homo sapiens GN=AP1S2 PE=1 SV=1	21	3	18.6
Q96GK7	Fumarylacetoacetate hydrolase domain-containing protein 2A OS=Homo sapiens GN=FAHD2A PE=1 SV=1	21	3	34.6
Q99436	Proteasome subunit beta type-7 OS=Homo sapiens GN=PSMB7 PE=1 SV=1	21	3	29.9
Q9H5K3	Protein O-mannose kinase OS=Homo sapiens GN=POMK PE=1 SV=1	21	3	40
Q6UWD8	Transmembrane protein C16orf54 OS=Homo sapiens GN=C16orf54 PE=1 SV=1	21	3	24.3
J3KSJ5	Uncharacterized protein C17orf62 OS=Homo sapiens GN=C17orf62 PE=1 SV=1	21	3	21.3
E9PFN4	Anion exchange protein OS=Homo sapiens GN=SLC4A7 PE=1 SV=1	21	2	135.2
P01034	Cystatin-C OS=Homo sapiens GN=CST3 PE=1 SV=1	21	2	15.8
Q86SX6	Glutaredoxin-related protein 5, mitochondrial OS=Homo sapiens GN=GLRX5 PE=1 SV=2	21	2	16.6
O14737	Programmed cell death protein 5 OS=Homo sapiens GN=PDCD5 PE=1 SV=3	21	2	14.3
K7ES93	TYRO protein tyrosine kinase-binding protein OS=Homo sapiens GN=TYROBP PE=1 SV=1	21	2	15.6
H3BN98	Uncharacterized protein (Fragment) OS=Homo sapiens PE=4 SV=2	21	2	27.2
Q8TCJ2	Dolichyl-diphosphooligosaccharide--protein glycosyltransferase subunit STT3B OS=Homo sapiens GN=STT3B PE=1 SV=1	20	17	93.6
Q5JWF2	Guanine nucleotide-binding protein G(s) subunit alpha isoforms XLas OS=Homo sapiens GN=GNAS PE=1 SV=2	20	17	111
Q70J99	Protein unc-13 homolog D OS=Homo sapiens GN=UNC13D PE=1 SV=1	20	14	123.2
Q9UHW9	Solute carrier family 12 member 6 OS=Homo sapiens GN=SLC12A6 PE=1 SV=2	20	14	127.5
P49588	Alanine--tRNA ligase, cytoplasmic OS=Homo sapiens GN=AARS PE=1 SV=2	20	13	106.7
Q96TA2	ATP-dependent zinc metalloprotease YME1L1 OS=Homo sapiens GN=YME1L1 PE=1 SV=2	20	13	86.4
P53618	Coatomer subunit beta OS=Homo sapiens GN=COPB1 PE=1 SV=3	20	13	107.1
Q8IWT6	Volume-regulated anion channel subunit LRRC8A OS=Homo sapiens GN=LRRC8A PE=1 SV=1	20	12	94.1
P13010	X-ray repair cross-complementing protein 5 OS=Homo sapiens GN=XRCC5 PE=1 SV=3	20	10	82.7
Q9BY15	Adhesion G protein-coupled receptor E3 OS=Homo sapiens GN=ADGRE3 PE=2 SV=2	20	9	72.6
Q9UHD8	Septin-9 OS=Homo sapiens GN=SEPT9 PE=1 SV=2	20	9	65.4
Q4G176	Acyl-CoA synthetase family member 3, mitochondrial OS=Homo sapiens GN=ACSF3 PE=1 SV=3	20	8	64.1
A0A0U1RRK1	Calcium uptake protein 1, mitochondrial OS=Homo sapiens GN=MICU1 PE=1 SV=1	20	8	54.9

Q6ZRP7	Sulfhydryl oxidase 2 OS=Homo sapiens GN=QSOX2 PE=1 SV=3	20	8	77.5
Q15120	[Pyruvate dehydrogenase (acetyl-transferring)] kinase isozyme 3, mitochondrial OS=Homo sapiens GN=PDK3 PE=1 SV=1	20	7	46.9
O00232	26S proteasome non-ATPase regulatory subunit 12 OS=Homo sapiens GN=PSMD12 PE=1 SV=3	20	7	52.9
P00390	Glutathione reductase, mitochondrial OS=Homo sapiens GN=GSR PE=1 SV=2	20	7	56.2
A0A0G2JIW1	Heat shock 70 kDa protein 1B OS=Homo sapiens GN=HSPA1B PE=1 SV=1	20	7	70.1
A0A0A0MTH3	Integrin-linked protein kinase OS=Homo sapiens GN=ILK PE=1 SV=1	20	7	54.6
P04181	Ornithine aminotransferase, mitochondrial OS=Homo sapiens GN=OAT PE=1 SV=1	20	7	48.5
J9JIE6	Calcium load-activated calcium channel OS=Homo sapiens GN=TMCO1 PE=1 SV=1	20	6	27.1
Q96KP4	Cytosolic non-specific dipeptidase OS=Homo sapiens GN=CNDP2 PE=1 SV=2	20	6	52.8
O43567	E3 ubiquitin-protein ligase RNF13 OS=Homo sapiens GN=RNF13 PE=1 SV=1	20	6	42.8
Q8NC42	E3 ubiquitin-protein ligase RNF149 OS=Homo sapiens GN=RNF149 PE=2 SV=2	20	6	43.1
Q6P1Q0	LETM1 domain-containing protein 1 OS=Homo sapiens GN=LETMD1 PE=1 SV=1	20	6	41.8
P51688	N-sulphoglucosamine sulphohydrolase OS=Homo sapiens GN=SGSH PE=1 SV=1	20	6	56.7
O95674	Phosphatidate cytidylyltransferase 2 OS=Homo sapiens GN=CDS2 PE=1 SV=1	20	6	51.4
A0A0A0MSV9	Tapasin OS=Homo sapiens GN=TAPBP PE=1 SV=1	20	6	53.9
Q9HC07	Transmembrane protein 165 OS=Homo sapiens GN=TMEM165 PE=1 SV=1	20	6	34.9
O00767	Acyl-CoA desaturase OS=Homo sapiens GN=SCD PE=1 SV=2	20	5	41.5
Q9NUU6	Inactive ubiquitin thioesterase FAM105A OS=Homo sapiens GN=FAM105A PE=2 SV=1	20	5	42.2
A0A0G2JLV7	Leukocyte-associated immunoglobulin-like receptor 1 OS=Homo sapiens GN=LAIR1 PE=1 SV=1	20	5	31.5
J3QQX2	Rho GDP-dissociation inhibitor 1 OS=Homo sapiens GN=ARHGDIA PE=1 SV=1	20	5	25.8
O75436	Vacuolar protein sorting-associated protein 26A OS=Homo sapiens GN=VPS26A PE=1 SV=2	20	5	38.1
Q9H0V9	VIP36-like protein OS=Homo sapiens GN=LMAN2L PE=1 SV=1	20	5	39.7
B3KY94	CDP-diacylglycerol--inositol 3-phosphatidyltransferase OS=Homo sapiens GN=CDIPT PE=1 SV=1	20	4	25.9
Q16775	Hydroxyacylglutathione hydrolase, mitochondrial OS=Homo sapiens GN=HAGH PE=1 SV=2	20	4	33.8
Q16563	Synaptophysin-like protein 1 OS=Homo sapiens GN=SYPL1 PE=1 SV=1	20	4	28.5
Q53S58	Transmembrane protein 177 OS=Homo sapiens GN=TMEM177 PE=2 SV=1	20	4	33.7
P58335	Anthrax toxin receptor 2 OS=Homo sapiens GN=ANTXR2 PE=1 SV=5	20	3	53.6
P59665	Neutrophil defensin 1 OS=Homo sapiens GN=DEFA1 PE=1 SV=1	20	3	10.2

P28070	Proteasome subunit beta type-4 OS=Homo sapiens GN=PSMB4 PE=1 SV=4	20	3	29.2
O15258	Protein RER1 OS=Homo sapiens GN=RER1 PE=1 SV=1	20	3	22.9
P26447	Protein S100-A4 OS=Homo sapiens GN=S100A4 PE=1 SV=1	20	3	11.7
Q01105	Protein SET OS=Homo sapiens GN=SET PE=1 SV=3	20	3	33.5
Q5W111	SPRY domain-containing protein 7 OS=Homo sapiens GN=SPRYD7 PE=1 SV=2	20	3	21.7
P32856	Syntaxin-2 OS=Homo sapiens GN=STX2 PE=1 SV=3	20	3	33.3
Q15121	Astrocytic phosphoprotein PEA-15 OS=Homo sapiens GN=PEA15 PE=1 SV=2	20	2	15
Q9BV81	ER membrane protein complex subunit 6 OS=Homo sapiens GN=EMC6 PE=1 SV=1	20	2	12
P10153	Non-secretory ribonuclease OS=Homo sapiens GN=RNASE2 PE=1 SV=2	20	2	18.3
O60499	Syntaxin-10 OS=Homo sapiens GN=STX10 PE=1 SV=1	20	2	28.1
P60604	Ubiquitin-conjugating enzyme E2 G2 OS=Homo sapiens GN=UBE2G2 PE=1 SV=1	20	2	18.6
Q14571	Inositol 1,4,5-trisphosphate receptor type 2 OS=Homo sapiens GN=ITPR2 PE=1 SV=2	19	30	307.9
O75165	DnaJ homolog subfamily C member 13 OS=Homo sapiens GN=DNAJC13 PE=1 SV=5	19	27	254.3
O00410	Importin-5 OS=Homo sapiens GN=IPO5 PE=1 SV=4	19	15	123.6
P19367	Hexokinase-1 OS=Homo sapiens GN=HK1 PE=1 SV=3	19	13	102.4
Q9BZQ8	Protein Niban OS=Homo sapiens GN=FAM129A PE=1 SV=1	19	12	103.1
Q8NCN5	Pyruvate dehydrogenase phosphatase regulatory subunit, mitochondrial OS=Homo sapiens GN=PDPR PE=1 SV=2	19	12	99.3
A0A0D9SF53	ATP-dependent RNA helicase DDX3X OS=Homo sapiens GN=DDX3X PE=1 SV=1	19	11	81.4
P26639	Threonine--tRNA ligase, cytoplasmic OS=Homo sapiens GN=TARS PE=1 SV=3	19	11	83.4
Q9H3S1	Semaphorin-4A OS=Homo sapiens GN=SEMA4A PE=1 SV=2	19	9	83.5
O15270	Serine palmitoyltransferase 2 OS=Homo sapiens GN=SPTLC2 PE=1 SV=1	19	9	62.9
P46199	Translation initiation factor IF-2, mitochondrial OS=Homo sapiens GN=MTIF2 PE=1 SV=2	19	9	81.3
P11279	Lysosome-associated membrane glycoprotein 1 OS=Homo sapiens GN=LAMP1 PE=1 SV=3	19	8	44.9
O00231	26S proteasome non-ATPase regulatory subunit 11 OS=Homo sapiens GN=PSMD11 PE=1 SV=3	19	7	47.4
Q32NB8	CDP-diacylglycerol--glycerol-3-phosphate 3-phosphatidyltransferase, mitochondrial OS=Homo sapiens GN=PGS1 PE=2 SV=1	19	7	62.7
P60228	Eukaryotic translation initiation factor 3 subunit E OS=Homo sapiens GN=EIF3E PE=1 SV=1	19	7	52.2
O95864	Fatty acid desaturase 2 OS=Homo sapiens GN=FADS2 PE=1 SV=1	19	7	52.2

J3KTA1	F-box and leucine-rich repeat protein 20, isoform CRA_a OS=Homo sapiens GN=FBXL20 PE=1 SV=1	19	7	48.5
Q86UL3	Glycerol-3-phosphate acyltransferase 4 OS=Homo sapiens GN=GPAT4 PE=1 SV=1	19	7	52
Q8NF37	Lysophosphatidylcholine acyltransferase 1 OS=Homo sapiens GN=LPCAT1 PE=1 SV=2	19	7	59.1
Q14108	Lysosome membrane protein 2 OS=Homo sapiens GN=SCARB2 PE=1 SV=2	19	7	54.3
Q658P3	Metalloreductase STEAP3 OS=Homo sapiens GN=STEAP3 PE=1 SV=2	19	7	54.6
O15427	Monocarboxylate transporter 4 OS=Homo sapiens GN=SLC16A3 PE=1 SV=1	19	7	49.4
Q7Z3D6	UPF0317 protein C14orf159, mitochondrial OS=Homo sapiens GN=C14orf159 PE=1 SV=2	19	7	66.4
P56937	3-keto-steroid reductase OS=Homo sapiens GN=HSD17B7 PE=1 SV=1	19	6	38.2
Q9BXS5	AP-1 complex subunit mu-1 OS=Homo sapiens GN=AP1M1 PE=1 SV=3	19	6	48.6
P13473	Lysosome-associated membrane glycoprotein 2 OS=Homo sapiens GN=LAMP2 PE=1 SV=2	19	6	44.9
Q6P4A7	Sideroflexin-4 OS=Homo sapiens GN=SFXN4 PE=1 SV=1	19	6	38
O75563	Src kinase-associated phosphoprotein 2 OS=Homo sapiens GN=SKAP2 PE=1 SV=1	19	6	41.2
Q8NFQ8	Torsin-1A-interacting protein 2 OS=Homo sapiens GN=TOR1AIP2 PE=1 SV=1	19	6	51.2
O43516	WAS/WASL-interacting protein family member 1 OS=Homo sapiens GN=WIPF1 PE=1 SV=3	19	6	51.2
P62753	40S ribosomal protein S6 OS=Homo sapiens GN=RPS6 PE=1 SV=1	19	5	28.7
Q9H665	IGF-like family receptor 1 OS=Homo sapiens GN=IGFLR1 PE=1 SV=1	19	5	37.9
P48059	LIM and senescent cell antigen-like-containing domain protein 1 OS=Homo sapiens GN=LIMS1 PE=1 SV=4	19	5	37.2
O15162	Phospholipid scramblase 1 OS=Homo sapiens GN=PLSCR1 PE=1 SV=1	19	5	35
Q9NWQ8	Phosphoprotein associated with glycosphingolipid-enriched microdomains 1 OS=Homo sapiens GN=PAG1 PE=1 SV=2	19	5	47
Q7L3T8	Probable proline--tRNA ligase, mitochondrial OS=Homo sapiens GN=PARS2 PE=1 SV=1	19	5	53.2
P22087	rRNA 2'-O-methyltransferase fibrillarin OS=Homo sapiens GN=FBL PE=1 SV=2	19	5	33.8
E9PSI1	Transmembrane 9 superfamily member OS=Homo sapiens PE=3 SV=1	19	5	92.3
P13498	Cytochrome b-245 light chain OS=Homo sapiens GN=CYBA PE=1 SV=3	19	4	21
Q9HBI0	Gamma-parvin OS=Homo sapiens GN=PARVG PE=1 SV=1	19	4	37.5
Q96S97	Myeloid-associated differentiation marker OS=Homo sapiens GN=MYADM PE=1 SV=2	19	4	35.3
P28062	Proteasome subunit beta type-8 OS=Homo sapiens GN=PSMB8 PE=1 SV=3	19	4	30.3
Q14761	Protein tyrosine phosphatase receptor type C-associated protein OS=Homo sapiens GN=PTPRCAP PE=1 SV=1	19	4	21.2
O00241	Signal-regulatory protein beta-1 OS=Homo sapiens GN=SIRPB1 PE=1 SV=5	19	4	43.2

Q8WVQ1	Soluble calcium-activated nucleotidase 1 OS=Homo sapiens GN=CANT1 PE=1 SV=1	19	4	44.8
Q9NQE9	Histidine triad nucleotide-binding protein 3 OS=Homo sapiens GN=HINT3 PE=1 SV=1	19	3	20.3
P22794	Protein EVI2A OS=Homo sapiens GN=EVI2A PE=2 SV=2	19	3	26.2
P10124	Serglycin OS=Homo sapiens GN=SRGN PE=1 SV=3	19	3	17.6
Q86X83	COMM domain-containing protein 2 OS=Homo sapiens GN=COMMD2 PE=1 SV=2	19	2	22.7
P61803	Dolichyl-diphosphooligosaccharide--protein glycosyltransferase subunit DAD1 OS=Homo sapiens GN=DAD1 PE=1 SV=3	19	2	12.5
Q8WZ82	Esterase OVCA2 OS=Homo sapiens GN=OVCA2 PE=1 SV=1	19	2	24.4
Q9UMX5	Neudesin OS=Homo sapiens GN=NENF PE=1 SV=1	19	2	18.8
O14966	Ras-related protein Rab-7L1 OS=Homo sapiens GN=RAB29 PE=1 SV=1	19	2	23.1
Q9NQC3	Reticulon-4 OS=Homo sapiens GN=RTN4 PE=1 SV=2	19	2	129.9
Q96IX5	Up-regulated during skeletal muscle growth protein 5 OS=Homo sapiens GN=USMG5 PE=1 SV=1	19	2	6.5
P27708	CAD protein OS=Homo sapiens GN=CAD PE=1 SV=3	18	25	242.8
Q9Y4D7	Plexin-D1 OS=Homo sapiens GN=PLXND1 PE=1 SV=3	18	23	211.9
Q9P2J5	Leucine--tRNA ligase, cytoplasmic OS=Homo sapiens GN=LARS PE=1 SV=2	18	17	134.4
Q9Y2H6	Fibronectin type-III domain-containing protein 3A OS=Homo sapiens GN=FNDC3A PE=1 SV=4	18	14	131.8
Q00653	Nuclear factor NF-kappa-B p100 subunit OS=Homo sapiens GN=NFKB2 PE=1 SV=4	18	10	96.7
O43747	AP-1 complex subunit gamma-1 OS=Homo sapiens GN=AP1G1 PE=1 SV=5	18	9	91.3
O95470	Sphingosine-1-phosphate lyase 1 OS=Homo sapiens GN=SGPL1 PE=1 SV=3	18	8	63.5
Q8N6G5	Chondroitin sulfate N-acetylgalactosaminyltransferase 2 OS=Homo sapiens GN=CSGALNACT2 PE=1 SV=1	18	7	62.5
O75879	Glutamyl-tRNA(Gln) amidotransferase subunit B, mitochondrial OS=Homo sapiens GN=GATB PE=1 SV=1	18	7	61.8
P43405	Tyrosine-protein kinase SYK OS=Homo sapiens GN=SYK PE=1 SV=1	18	7	72
P04062	Glucosylceramidase OS=Homo sapiens GN=GBA PE=1 SV=3	18	6	59.7
Q9NX62	Inositol monophosphatase 3 OS=Homo sapiens GN=IMPAD1 PE=1 SV=1	18	6	38.7
O75874	Isocitrate dehydrogenase [NADP] cytoplasmic OS=Homo sapiens GN=IDH1 PE=1 SV=2	18	6	46.6
O43586	Proline-serine-threonine phosphatase-interacting protein 1 OS=Homo sapiens GN=PSTPIP1 PE=1 SV=1	18	6	47.6
P27918	Properdin OS=Homo sapiens GN=CFP PE=1 SV=2	18	6	51.2
Q8NBN3	Transmembrane protein 87A OS=Homo sapiens GN=TMEM87A PE=1 SV=3	18	6	63.4
P13861	cAMP-dependent protein kinase type II-alpha regulatory subunit OS=Homo sapiens GN=PRKAR2A PE=1 SV=2	18	5	45.5

A0A0A0MR51	Fatty acid desaturase 1 OS=Homo sapiens GN=FADS1 PE=1 SV=1	18	5	57.8
P50502	Hsc70-interacting protein OS=Homo sapiens GN=ST13 PE=1 SV=2	18	5	41.3
I3L0B8	Solute carrier family 25 member 52 OS=Homo sapiens GN=SLC25A52 PE=1 SV=1	18	5	34.9
Q9P2W9	Syntaxin-18 OS=Homo sapiens GN=STX18 PE=1 SV=1	18	5	38.7
P46109	Crk-like protein OS=Homo sapiens GN=CRKL PE=1 SV=1	18	4	33.8
Q9BQ95	Evolutionarily conserved signaling intermediate in Toll pathway, mitochondrial OS=Homo sapiens GN=ECSIT PE=1 SV=1	18	4	49.1
Q96ND0	Protein FAM210A OS=Homo sapiens GN=FAM210A PE=1 SV=2	18	4	30.8
Q99426	Tubulin-folding cofactor B OS=Homo sapiens GN=TBCB PE=1 SV=2	18	4	27.3
O60725	Protein-S-isoprenylcysteine O-methyltransferase OS=Homo sapiens GN=ICMT PE=1 SV=1	18	3	31.9
A0A087WWM0	Trafficking protein particle complex subunit 3 OS=Homo sapiens GN=TRAPPC3 PE=1 SV=1	18	3	21.2
P78537	Biogenesis of lysosome-related organelles complex 1 subunit 1 OS=Homo sapiens GN=BLOC1S1 PE=1 SV=2	18	2	17.3
O95415	Brain protein I3 OS=Homo sapiens GN=BRI3 PE=2 SV=1	18	2	13.6
F8W8Z9	Mitochondrial import receptor subunit TOM5 homolog OS=Homo sapiens GN=TOMM5 PE=1 SV=1	18	2	11
Q8N292	Protein GAPT OS=Homo sapiens GN=GAPT PE=1 SV=1	18	2	17.9
P60903	Protein S100-A10 OS=Homo sapiens GN=S100A10 PE=1 SV=2	18	2	11.2
Q04941	Proteolipid protein 2 OS=Homo sapiens GN=PLP2 PE=1 SV=1	18	2	16.7
Q92614	Unconventional myosin-XVIIIa OS=Homo sapiens GN=MYO18A PE=1 SV=3	17	26	233
P41252	Isoleucine--tRNA ligase, cytoplasmic OS=Homo sapiens GN=IARS PE=1 SV=2	17	16	144.4
Q13576	Ras GTPase-activating-like protein IQGAP2 OS=Homo sapiens GN=IQGAP2 PE=1 SV=4	17	16	180.5
Q13393	Phospholipase D1 OS=Homo sapiens GN=PLD1 PE=1 SV=1	17	13	124.1
Q96A65	Exocyst complex component 4 OS=Homo sapiens GN=EXOC4 PE=1 SV=1	17	12	110.4
Q13443	Disintegrin and metalloproteinase domain-containing protein 9 OS=Homo sapiens GN=ADAM9 PE=1 SV=1	17	11	90.5
Q13200	26S proteasome non-ATPase regulatory subunit 2 OS=Homo sapiens GN=PSMD2 PE=1 SV=3	17	10	100.1
O43292	Glycosylphosphatidylinositol anchor attachment 1 protein OS=Homo sapiens GN=GPAA1 PE=1 SV=3	17	10	67.6
C9JIZ6	Prosaposin OS=Homo sapiens GN=PSAP PE=1 SV=2	17	10	58.4
B4DR61	Protein transport protein Sec61 subunit alpha isoform 1 OS=Homo sapiens GN=SEC61A1 PE=1 SV=1	17	10	52.9
P18433	Receptor-type tyrosine-protein phosphatase alpha OS=Homo sapiens GN=PTPRA PE=1 SV=2	17	10	90.5
Q8NCG7	Sn1-specific diacylglycerol lipase beta OS=Homo sapiens GN=DAGLB PE=1 SV=2	17	10	73.7

Q9Y5S1	Transient receptor potential cation channel subfamily V member 2 OS=Homo sapiens GN=TRPV2 PE=1 SV=1	17	10	85.9
J3KNA0	Mitochondrial inner membrane protein OXA1L OS=Homo sapiens GN=OXA1L PE=1 SV=1	17	9	55.2
P11171	Protein 4.1 OS=Homo sapiens GN=EPB41 PE=1 SV=4	17	9	97
Q969S9	Ribosome-releasing factor 2, mitochondrial OS=Homo sapiens GN=GFM2 PE=1 SV=1	17	9	86.5
Q03252	Lamin-B2 OS=Homo sapiens GN=LMNB2 PE=1 SV=4	17	8	69.9
O76094	Signal recognition particle subunit SRP72 OS=Homo sapiens GN=SRP72 PE=1 SV=3	17	8	74.6
Q9UBM7	7-dehydrocholesterol reductase OS=Homo sapiens GN=DHCR7 PE=1 SV=1	17	7	54.5
Q99808	Equilibrative nucleoside transporter 1 OS=Homo sapiens GN=SLC29A1 PE=1 SV=3	17	7	50.2
P50336	Protoporphyrinogen oxidase OS=Homo sapiens GN=PPOX PE=1 SV=1	17	7	50.7
Q15008	26S proteasome non-ATPase regulatory subunit 6 OS=Homo sapiens GN=PSMD6 PE=1 SV=1	17	6	45.5
Q8IZP0	Abl interactor 1 OS=Homo sapiens GN=ABI1 PE=1 SV=4	17	6	55
Q8TB61	Adenosine 3'-phospho 5'-phosphosulfate transporter 1 OS=Homo sapiens GN=SLC35B2 PE=1 SV=1	17	6	47.5
P26572	Alpha-1,3-mannosyl-glycoprotein 2-beta-N-acetylglucosaminyltransferase OS=Homo sapiens GN=MGAT1 PE=1 SV=2	17	6	50.8
Q8NHH9	Atlastin-2 OS=Homo sapiens GN=ATL2 PE=1 SV=2	17	6	66.2
P14384	Carboxypeptidase M OS=Homo sapiens GN=CPM PE=1 SV=2	17	6	50.5
Q6YP21	Kynurenine--oxoglutarate transaminase 3 OS=Homo sapiens GN=KYAT3 PE=1 SV=1	17	6	51.4
Q14318	Peptidyl-prolyl cis-trans isomerase FKBP8 OS=Homo sapiens GN=FKBP8 PE=1 SV=2	17	6	44.5
P48651	Phosphatidylserine synthase 1 OS=Homo sapiens GN=PTDSS1 PE=1 SV=1	17	6	55.5
P61011	Signal recognition particle 54 kDa protein OS=Homo sapiens GN=SRP54 PE=1 SV=1	17	6	55.7
Q13277	Syntaxin-3 OS=Homo sapiens GN=STX3 PE=1 SV=3	17	6	33.1
Q9UKJ1	Paired immunoglobulin-like type 2 receptor alpha OS=Homo sapiens GN=PILRA PE=1 SV=3	17	5	34
Q9H6R6	Palmitoyltransferase ZDHHC6 OS=Homo sapiens GN=ZDHHC6 PE=1 SV=1	17	5	47.6
O14763	Tumor necrosis factor receptor superfamily member 10B OS=Homo sapiens GN=TNFRSF10B PE=1 SV=2	17	5	47.8
A8MUS3	60S ribosomal protein L23a OS=Homo sapiens GN=RPL23A PE=1 SV=1	17	4	21.9
Q8WY22	BRI3-binding protein OS=Homo sapiens GN=BRI3BP PE=1 SV=1	17	4	27.8
Q8ND76	Cyclin-Y OS=Homo sapiens GN=CCNY PE=1 SV=2	17	4	39.3
P50402	Emerin OS=Homo sapiens GN=EMD PE=1 SV=1	17	4	29
Q7LGA3	Heparan sulfate 2-O-sulfotransferase 1 OS=Homo sapiens GN=HS2ST1 PE=1 SV=1	17	4	41.9

O75352	Mannose-P-dolichol utilization defect 1 protein OS=Homo sapiens GN=MPDU1 PE=1 SV=2	17	4	26.6
Q9NZD8	Maspardin OS=Homo sapiens GN=SPG21 PE=1 SV=1	17	4	34.9
Q8NET5	NFAT activation molecule 1 OS=Homo sapiens GN=NFAM1 PE=1 SV=1	17	4	29.7
Q96RD7	Pannexin-1 OS=Homo sapiens GN=PANX1 PE=1 SV=4	17	4	48
Q9Y5Y5	Peroxisomal membrane protein PEX16 OS=Homo sapiens GN=PEX16 PE=1 SV=2	17	4	38.6
H7C2N1	Prothymosin alpha (Fragment) OS=Homo sapiens GN=PTMA PE=1 SV=1	17	4	15.8
O43464	Serine protease HTRA2, mitochondrial OS=Homo sapiens GN=HTRA2 PE=1 SV=2	17	4	48.8
Q8TD22	Sideroflexin-5 OS=Homo sapiens GN=SFXN5 PE=1 SV=1	17	4	37.1
Q8NBK3	Sulfatase-modifying factor 1 OS=Homo sapiens GN=SUMF1 PE=1 SV=3	17	4	40.5
O60784	Target of Myb protein 1 OS=Homo sapiens GN=TOM1 PE=1 SV=2	17	4	53.8
Q8NBM4	Ubiquitin-associated domain-containing protein 2 OS=Homo sapiens GN=UBAC2 PE=1 SV=1	17	4	38.9
Q96EL2	28S ribosomal protein S24, mitochondrial OS=Homo sapiens GN=MRPS24 PE=1 SV=1	17	3	19
Q96GC5	39S ribosomal protein L48, mitochondrial OS=Homo sapiens GN=MRPL48 PE=1 SV=2	17	3	23.9
P40199	Carcinoembryonic antigen-related cell adhesion molecule 6 OS=Homo sapiens GN=CEACAM6 PE=1 SV=3	17	3	37.2
P68431	Histone H3.1 OS=Homo sapiens GN=HIST1H3A PE=1 SV=2	17	3	15.4
F5H189	LYR motif-containing protein 4 OS=Homo sapiens GN=LYRM4 PE=1 SV=3	17	3	15.2
Q969V5	Mitochondrial ubiquitin ligase activator of NFKB 1 OS=Homo sapiens GN=MUL1 PE=1 SV=1	17	3	39.8
Q9H6K4	Optic atrophy 3 protein OS=Homo sapiens GN=OPA3 PE=1 SV=1	17	3	20
O75880	Protein SCO1 homolog, mitochondrial OS=Homo sapiens GN=SCO1 PE=1 SV=1	17	3	33.8
Q14088	Ras-related protein Rab-33A OS=Homo sapiens GN=RAB33A PE=1 SV=2	17	3	26.6
O00743	Serine/threonine-protein phosphatase 6 catalytic subunit OS=Homo sapiens GN=PPP6C PE=1 SV=1	17	3	35.1
A0A087WUM0	SYNJ2BP-COX16 readthrough (Fragment) OS=Homo sapiens GN=SYNJ2BP-COX16 PE=4 SV=1	17	3	20.8
P53999	Activated RNA polymerase II transcriptional coactivator p15 OS=Homo sapiens GN=SUB1 PE=1 SV=3	17	2	14.4
Q53S33	BolA-like protein 3 OS=Homo sapiens GN=BOLA3 PE=1 SV=1	17	2	12.1
Q9BY32	Inosine triphosphate pyrophosphatase OS=Homo sapiens GN=ITPA PE=1 SV=2	17	2	21.4
Q13907	Isopentenyl-diphosphate Delta-isomerase 1 OS=Homo sapiens GN=IDI1 PE=1 SV=2	17	2	26.3
A6NCE7	Microtubule-associated proteins 1A/1B light chain 3 beta 2 OS=Homo sapiens GN=MAP1LC3B2 PE=2 SV=1	17	2	14.6
A8MVS5	Protein HIDE1 OS=Homo sapiens GN=HIDE1 PE=2 SV=2	17	2	24.7

Q5BJH7	Protein YIF1B OS=Homo sapiens GN=YIF1B PE=1 SV=1	17	2	34.4
Q6P9G4	Transmembrane protein 154 OS=Homo sapiens GN=TMEM154 PE=1 SV=2	17	2	20.5
F8VXU5	Vacuolar protein sorting-associated protein 29 OS=Homo sapiens GN=VPS29 PE=1 SV=1	17	2	24
Q96AJ9	Vesicle transport through interaction with t-SNAREs homolog 1A OS=Homo sapiens GN=VTI1A PE=1 SV=2	17	2	25.2
Q9P265	Disco-interacting protein 2 homolog B OS=Homo sapiens GN=DIP2B PE=1 SV=3	16	16	171.4
P06213	Insulin receptor OS=Homo sapiens GN=INSR PE=1 SV=4	16	16	156.2
A0A0G2JPP5	Protein scribble homolog OS=Homo sapiens GN=SCRIB PE=1 SV=1	16	16	177.6
Q86VP6	Cullin-associated NEDD8-dissociated protein 1 OS=Homo sapiens GN=CAND1 PE=1 SV=2	16	14	136.3
O94874	E3 UFM1-protein ligase 1 OS=Homo sapiens GN=UFL1 PE=1 SV=2	16	11	89.5
Q8NE01	Metal transporter CNNM3 OS=Homo sapiens GN=CNNM3 PE=1 SV=1	16	10	76.1
Q96QK1	Vacuolar protein sorting-associated protein 35 OS=Homo sapiens GN=VPS35 PE=1 SV=2	16	10	91.6
P05067	Amyloid beta A4 protein OS=Homo sapiens GN=APP PE=1 SV=3	16	9	86.9
P25098	Beta-adrenergic receptor kinase 1 OS=Homo sapiens GN=GRK2 PE=1 SV=2	16	9	79.5
O60645	Exocyst complex component 3 OS=Homo sapiens GN=EXOC3 PE=1 SV=2	16	9	86.8
P47897	Glutamine--tRNA ligase OS=Homo sapiens GN=QARS PE=1 SV=1	16	9	87.7
Q6IC98	GRAM domain-containing protein 4 OS=Homo sapiens GN=GRAMD4 PE=1 SV=1	16	9	66.4
P02545	Prelamin-A/C OS=Homo sapiens GN=LMNA PE=1 SV=1	16	8	74.1
Q92544	Transmembrane 9 superfamily member 4 OS=Homo sapiens GN=TM9SF4 PE=1 SV=2	16	8	74.5
Q9H269	Vacuolar protein sorting-associated protein 16 homolog OS=Homo sapiens GN=VPS16 PE=1 SV=2	16	8	94.6
P61221	ATP-binding cassette sub-family E member 1 OS=Homo sapiens GN=ABCE1 PE=1 SV=1	16	7	67.3
Q96K12	Fatty acyl-CoA reductase 2 OS=Homo sapiens GN=FAR2 PE=2 SV=1	16	7	59.4
Q96II8	Leucine-rich repeat and calponin homology domain-containing protein 3 OS=Homo sapiens GN=LRCH3 PE=1 SV=2	16	7	86
P03915	NADH-ubiquinone oxidoreductase chain 5 OS=Homo sapiens GN=MT-ND5 PE=1 SV=2	16	7	67
Q8NHP8	Putative phospholipase B-like 2 OS=Homo sapiens GN=PLBD2 PE=1 SV=2	16	7	65.4
Q9C037	E3 ubiquitin-protein ligase TRIM4 OS=Homo sapiens GN=TRIM4 PE=1 SV=2	16	6	57.4
O60506	Heterogeneous nuclear ribonucleoprotein Q OS=Homo sapiens GN=SYNCRIP PE=1 SV=2	16	6	69.6
P53990	IST1 homolog OS=Homo sapiens GN=IST1 PE=1 SV=1	16	6	39.7
D6RE79	Major facilitator superfamily domain-containing protein 10 OS=Homo sapiens GN=MFSD10 PE=1 SV=1	16	6	50.4

P35998	26S protease regulatory subunit 7 OS=Homo sapiens GN=PSMC2 PE=1 SV=3	16	5	48.6
H7C5E2	B-cell receptor-associated protein 29 (Fragment) OS=Homo sapiens GN=BCAP29 PE=1 SV=1	16	5	34
Q08334	Interleukin-10 receptor subunit beta OS=Homo sapiens GN=IL10RB PE=1 SV=2	16	5	37
Q15418	Ribosomal protein S6 kinase alpha-1 OS=Homo sapiens GN=RPS6KA1 PE=1 SV=2	16	5	82.7
Q96GQ5	RUS1 family protein C16orf58 OS=Homo sapiens GN=C16orf58 PE=1 SV=2	16	5	51
Q9Y286	Sialic acid-binding Ig-like lectin 7 OS=Homo sapiens GN=SIGLEC7 PE=1 SV=1	16	5	51.1
O15121	Sphingolipid delta(4)-desaturase DES1 OS=Homo sapiens GN=DEGS1 PE=1 SV=1	16	5	37.8
P37837	Transaldolase OS=Homo sapiens GN=TALDO1 PE=1 SV=2	16	5	37.5
Q8N1S5	Zinc transporter ZIP11 OS=Homo sapiens GN=SLC39A11 PE=2 SV=3	16	5	35.4
P27701	CD82 antigen OS=Homo sapiens GN=CD82 PE=1 SV=1	16	4	29.6
P09497	Clathrin light chain B OS=Homo sapiens GN=CLTB PE=1 SV=1	16	4	25.2
Q01459	Di-N-acetylchitobiase OS=Homo sapiens GN=CTBS PE=1 SV=1	16	4	43.7
H0YBZ2	HLA class II histocompatibility antigen gamma chain (Fragment) OS=Homo sapiens GN=CD74 PE=1 SV=1	16	4	35.1
Q15722	Leukotriene B4 receptor 1 OS=Homo sapiens GN=LTB4R PE=1 SV=2	16	4	37.5
Q9NX46	Poly(ADP-ribose) glycohydrolase ARH3 OS=Homo sapiens GN=ADPRHL2 PE=1 SV=1	16	4	38.9
P60891	Ribose-phosphate pyrophosphokinase 1 OS=Homo sapiens GN=PRPS1 PE=1 SV=2	16	4	34.8
P25942	Tumor necrosis factor receptor superfamily member 5 OS=Homo sapiens GN=CD40 PE=1 SV=1	16	4	30.6
J3KR55	Tyrosine-protein phosphatase non-receptor type 7 (Fragment) OS=Homo sapiens GN=PTPN7 PE=1 SV=1	16	4	49.1
F5GYJ8	Ubiquitin thioesterase OTUB1 OS=Homo sapiens GN=OTUB1 PE=1 SV=1	16	4	32.3
P62266	40S ribosomal protein S23 OS=Homo sapiens GN=RPS23 PE=1 SV=3	16	3	15.8
Q9H4A6	Golgi phosphoprotein 3 OS=Homo sapiens GN=GOLPH3 PE=1 SV=1	16	3	33.8
O14910	Protein lin-7 homolog A OS=Homo sapiens GN=LIN7A PE=1 SV=2	16	3	26
Q9Y385	Ubiquitin-conjugating enzyme E2 J1 OS=Homo sapiens GN=UBE2J1 PE=1 SV=2	16	3	35.2
P42766	60S ribosomal protein L35 OS=Homo sapiens GN=RPL35 PE=1 SV=2	16	2	14.5
Q9NVZ3	Adaptin ear-binding coat-associated protein 2 OS=Homo sapiens GN=NECAP2 PE=1 SV=1	16	2	28.3
Q9UII2	ATPase inhibitor, mitochondrial OS=Homo sapiens GN=ATPIF1 PE=1 SV=1	16	2	12.2
U3KQB3	Casein kinase I isoform gamma-1 OS=Homo sapiens GN=CSNK1G1 PE=1 SV=1	16	2	54.4
Q5SRQ6	Casein kinase II subunit beta OS=Homo sapiens GN=CSNK2B PE=1 SV=2	16	2	26.9

A0A0G2JH66	HLA class I histocompatibility antigen, alpha chain F OS=Homo sapiens GN=HLA-F PE=1 SV=1	16	2	50.4
Q86U28	Iron-sulfur cluster assembly 2 homolog, mitochondrial OS=Homo sapiens GN=ISCA2 PE=1 SV=2	16	2	16.5
P61758	Prefoldin subunit 3 OS=Homo sapiens GN=VBP1 PE=1 SV=3	16	2	22.6
Q9H098	Protein FAM107B OS=Homo sapiens GN=FAM107B PE=1 SV=1	16	2	15.5
O95295	SNARE-associated protein Snapin OS=Homo sapiens GN=SNAPIN PE=1 SV=1	16	2	14.9
D3DVS1	Transmembrane protein 128 OS=Homo sapiens GN=TMEM128 PE=1 SV=1	16	2	18.8
P06241	Tyrosine-protein kinase Fyn OS=Homo sapiens GN=FYN PE=1 SV=3	16	2	60.7
P78527	DNA-dependent protein kinase catalytic subunit OS=Homo sapiens GN=PRKDC PE=1 SV=3	15	48	468.8
A0A0A0MRV0	Ribosome-binding protein 1 OS=Homo sapiens GN=RRBP1 PE=1 SV=1	15	14	152.4
P04920	Anion exchange protein 2 OS=Homo sapiens GN=SLC4A2 PE=1 SV=4	15	13	136.9
O15118	Niemann-Pick C1 protein OS=Homo sapiens GN=NPC1 PE=1 SV=2	15	13	142.1
P29597	Non-receptor tyrosine-protein kinase TYK2 OS=Homo sapiens GN=TYK2 PE=1 SV=3	15	13	133.6
Q9ULQ1	Two pore calcium channel protein 1 OS=Homo sapiens GN=TPCN1 PE=1 SV=3	15	10	94.1
Q6PJF5	Inactive rhomboid protein 2 OS=Homo sapiens GN=RHBDF2 PE=1 SV=2	15	9	96.6
P15498	Proto-oncogene vav OS=Homo sapiens GN=VAV1 PE=1 SV=4	15	9	98.3
Q9UPT5	Exocyst complex component 7 OS=Homo sapiens GN=EXOC7 PE=1 SV=3	15	8	83.3
P52790	Hexokinase-3 OS=Homo sapiens GN=HK3 PE=1 SV=2	15	8	99
P98155	Very low-density lipoprotein receptor OS=Homo sapiens GN=VLDLR PE=1 SV=1	15	8	96
Q6L8Q7	2',5'-phosphodiesterase 12 OS=Homo sapiens GN=PDE12 PE=1 SV=2	15	7	67.3
Q9UKM7	Endoplasmic reticulum mannosyl-oligosaccharide 1,2-alpha-mannosidase OS=Homo sapiens GN=MAN1B1 PE=1 SV=2	15	7	79.5
Q9UQ90	Paraplegin OS=Homo sapiens GN=SPG7 PE=1 SV=2	15	7	88.2
Q9UNF0	Protein kinase C and casein kinase substrate in neurons protein 2 OS=Homo sapiens GN=PACSIN2 PE=1 SV=2	15	7	55.7
O95747	Serine/threonine-protein kinase OSR1 OS=Homo sapiens GN=OXSR1 PE=1 SV=1	15	7	58
Q6PML9	Zinc transporter 9 OS=Homo sapiens GN=SLC30A9 PE=1 SV=1	15	7	63.5
Q9NZK5	Adenosine deaminase CECR1 OS=Homo sapiens GN=CECR1 PE=1 SV=2	15	6	58.9
Q9Y6G9	Cytoplasmic dynein 1 light intermediate chain 1 OS=Homo sapiens GN=DYNC1LI1 PE=1 SV=3	15	6	56.5
A0A0A0MT83	Isovaleryl-CoA dehydrogenase, mitochondrial OS=Homo sapiens GN=IVD PE=1 SV=1	15	6	46.6
P13647	Keratin, type II cytoskeletal 5 OS=Homo sapiens GN=KRT5 PE=1 SV=3	15	6	62.3

Q96GW9	Methionine--tRNA ligase, mitochondrial OS=Homo sapiens GN=MARS2 PE=1 SV=2	15	6	66.5
O15091	Mitochondrial ribonuclease P protein 3 OS=Homo sapiens GN=KIAA0391 PE=1 SV=2	15	6	67.3
Q8TB96	T-cell immunomodulatory protein OS=Homo sapiens GN=ITFG1 PE=1 SV=1	15	6	68.1
Q6NXT6	Transmembrane anterior posterior transformation protein 1 homolog OS=Homo sapiens GN=TAPT1 PE=1 SV=1	15	6	64.2
O75718	Cartilage-associated protein OS=Homo sapiens GN=CRTAP PE=1 SV=1	15	5	46.5
Q7L9B9	Endonuclease/exonuclease/phosphatase family domain-containing protein 1 OS=Homo sapiens GN=EEPD1 PE=1 SV=2	15	5	62.4
P14324	Farnesyl pyrophosphate synthase OS=Homo sapiens GN=FDPS PE=1 SV=4	15	5	48.2
P16402	Histone H1.3 OS=Homo sapiens GN=HIST1H1D PE=1 SV=2	15	5	22.3
O95822	Malonyl-CoA decarboxylase, mitochondrial OS=Homo sapiens GN=MLYCD PE=1 SV=3	15	5	55
H0Y8C3	Mitochondrial carrier homolog 1 (Fragment) OS=Homo sapiens GN=MTCH1 PE=1 SV=1	15	5	43.1
Q8NBM8	Prenylcysteine oxidase-like OS=Homo sapiens GN=PCYOX1L PE=1 SV=2	15	5	54.6
P48595	Serpin B10 OS=Homo sapiens GN=SERPINB10 PE=1 SV=1	15	5	45.4
P10644	cAMP-dependent protein kinase type I-alpha regulatory subunit OS=Homo sapiens GN=PRKAR1A PE=1 SV=1	15	4	43
M0R1T5	Charged multivesicular body protein 2a (Fragment) OS=Homo sapiens GN=CHMP2A PE=1 SV=1	15	4	24.9
O75907	Diacylglycerol O-acyltransferase 1 OS=Homo sapiens GN=DGAT1 PE=1 SV=2	15	4	55.2
Q9NR77	Peroxisomal membrane protein 2 OS=Homo sapiens GN=PXMP2 PE=1 SV=3	15	4	22.2
A0A0U1RRM4	Polypyrimidine tract-binding protein 1 OS=Homo sapiens GN=PTBP1 PE=1 SV=1	15	4	62.4
O15260	Surfeit locus protein 4 OS=Homo sapiens GN=SURF4 PE=1 SV=3	15	4	30.4
Q9H1E5	Thioredoxin-related transmembrane protein 4 OS=Homo sapiens GN=TMX4 PE=1 SV=1	15	4	38.9
P49407	Beta-arrestin-1 OS=Homo sapiens GN=ARRB1 PE=1 SV=2	15	3	47
A0A0C4DGQ5	Calpain small subunit 1 OS=Homo sapiens GN=CAPNS1 PE=1 SV=1	15	3	33.8
Q53TN4	Cytochrome b reductase 1 OS=Homo sapiens GN=CYBRD1 PE=1 SV=1	15	3	31.6
Q8WVC6	Dephospho-CoA kinase domain-containing protein OS=Homo sapiens GN=DCAKD PE=1 SV=1	15	3	26.5
E5RI87	E3 ubiquitin-protein ligase RNF130 OS=Homo sapiens GN=RNF130 PE=1 SV=1	15	3	46.5
Q07108	Early activation antigen CD69 OS=Homo sapiens GN=CD69 PE=1 SV=1	15	3	22.5
E9PRY8	Elongation factor 1-delta OS=Homo sapiens GN=EEF1D PE=1 SV=1	15	3	76.5
Q07820	Induced myeloid leukemia cell differentiation protein Mcl-1 OS=Homo sapiens GN=MCL1 PE=1 SV=3	15	3	37.3
O94903	Proline synthase co-transcribed bacterial homolog protein OS=Homo sapiens GN=PROSC PE=1 SV=1	15	3	30.3

Q8NFV4	Protein ABHD11 OS=Homo sapiens GN=ABHD11 PE=1 SV=1	15	3	34.7
Q96GS6	Protein ABHD17A OS=Homo sapiens GN=ABHD17A PE=1 SV=1	15	3	34
H3BU86	STX16-NPEPL1 readthrough (NMD candidate) OS=Homo sapiens GN=STX16-NPEPL1 PE=3 SV=1	15	3	43.2
Q9UNK0	Syntaxin-8 OS=Homo sapiens GN=STX8 PE=1 SV=2	15	3	26.9
G8JLH6	Tetraspanin (Fragment) OS=Homo sapiens GN=CD9 PE=1 SV=1	15	3	25.4
Q9UGM6	Tryptophan--tRNA ligase, mitochondrial OS=Homo sapiens GN=WARS2 PE=1 SV=1	15	3	40.1
Q9BRG1	Vacuolar protein-sorting-associated protein 25 OS=Homo sapiens GN=VPS25 PE=1 SV=1	15	3	20.7
Q15056	Eukaryotic translation initiation factor 4H OS=Homo sapiens GN=EIF4H PE=1 SV=5	15	2	27.4
O75629	Protein CREG1 OS=Homo sapiens GN=CREG1 PE=1 SV=1	15	2	24.1
P32322	Pyrroline-5-carboxylate reductase 1, mitochondrial OS=Homo sapiens GN=PYCR1 PE=1 SV=2	15	2	33.3
Q96AX2	Ras-related protein Rab-37 OS=Homo sapiens GN=RAB37 PE=1 SV=3	15	2	24.8
P62318	Small nuclear ribonucleoprotein Sm D3 OS=Homo sapiens GN=SNRPD3 PE=1 SV=1	15	2	13.9
O43396	Thioredoxin-like protein 1 OS=Homo sapiens GN=TXNL1 PE=1 SV=3	15	2	32.2
Q86SZ2	Trafficking protein particle complex subunit 6B OS=Homo sapiens GN=TRAPPC6B PE=1 SV=1	15	2	18
Q96PC5	Melanoma inhibitory activity protein 2 OS=Homo sapiens GN=MIA2 PE=1 SV=4	14	19	159.7
Q8TCU6	Phosphatidylinositol 3,4,5-trisphosphate-dependent Rac exchanger 1 protein OS=Homo sapiens GN=PREX1 PE=1 SV=3	14	17	186.1
Q6P179	Endoplasmic reticulum aminopeptidase 2 OS=Homo sapiens GN=ERAP2 PE=1 SV=2	14	12	110.4
O00462	Beta-mannosidase OS=Homo sapiens GN=MANBA PE=2 SV=3	14	10	100.8
Q9NZ08	Endoplasmic reticulum aminopeptidase 1 OS=Homo sapiens GN=ERAP1 PE=1 SV=3	14	10	107.2
Q8IY17	Neuropathy target esterase OS=Homo sapiens GN=PNPLA6 PE=1 SV=2	14	10	149.9
B4E2Q0	Calcium-transporting ATPase OS=Homo sapiens GN=ATP2C1 PE=1 SV=1	14	9	104.6
O75116	Rho-associated protein kinase 2 OS=Homo sapiens GN=ROCK2 PE=1 SV=4	14	9	160.8
Q96FS4	Signal-induced proliferation-associated protein 1 OS=Homo sapiens GN=SIPA1 PE=1 SV=1	14	9	112.1
P10253	Lysosomal alpha-glucosidase OS=Homo sapiens GN=GAA PE=1 SV=4	14	8	105.3
O14744	Protein arginine N-methyltransferase 5 OS=Homo sapiens GN=PRMT5 PE=1 SV=4	14	8	72.6
Q9Y487	V-type proton ATPase 116 kDa subunit a isoform 2 OS=Homo sapiens GN=ATP6V0A2 PE=1 SV=2	14	8	98
P55884	Eukaryotic translation initiation factor 3 subunit B OS=Homo sapiens GN=EIF3B PE=1 SV=3	14	7	92.4
P28799	Granulins OS=Homo sapiens GN=GRN PE=1 SV=2	14	7	63.5

Q9Y3L3	SH3 domain-binding protein 1 OS=Homo sapiens GN=SH3BP1 PE=1 SV=3	14	7	75.7
P11166	Solute carrier family 2, facilitated glucose transporter member 1 OS=Homo sapiens GN=SLC2A1 PE=1 SV=2	14	7	54
Q96DM3	Uncharacterized protein C18orf8 OS=Homo sapiens GN=C18orf8 PE=1 SV=2	14	7	74.9
P20073	Annexin A7 OS=Homo sapiens GN=ANXA7 PE=1 SV=3	14	6	52.7
Q8NI60	Atypical kinase COQ8A, mitochondrial OS=Homo sapiens GN=COQ8A PE=1 SV=1	14	6	71.9
H7BY55	Complement decay-accelerating factor OS=Homo sapiens GN=CD55 PE=1 SV=2	14	6	58.9
P30038	Delta-1-pyrroline-5-carboxylate dehydrogenase, mitochondrial OS=Homo sapiens GN=ALDH4A1 PE=1 SV=3	14	6	61.7
Q96JJ3	Engulfment and cell motility protein 2 OS=Homo sapiens GN=ELMO2 PE=1 SV=2	14	6	82.6
O75387	Large neutral amino acids transporter small subunit 3 OS=Homo sapiens GN=SLC43A1 PE=1 SV=1	14	6	61.4
Q9BU23	Lipase maturation factor 2 OS=Homo sapiens GN=LMF2 PE=1 SV=2	14	6	79.6
P34910	Protein EVI2B OS=Homo sapiens GN=EVI2B PE=1 SV=2	14	6	48.6
Q15629	Translocating chain-associated membrane protein 1 OS=Homo sapiens GN=TRAM1 PE=1 SV=3	14	6	43
Q96Q11	CCA tRNA nucleotidyltransferase 1, mitochondrial OS=Homo sapiens GN=TRNT1 PE=1 SV=2	14	5	50.1
Q7KZN9	Cytochrome c oxidase assembly protein COX15 homolog OS=Homo sapiens GN=COX15 PE=1 SV=1	14	5	46
O00214	Galectin-8 OS=Homo sapiens GN=LGALS8 PE=1 SV=4	14	5	35.8
P15260	Interferon gamma receptor 1 OS=Homo sapiens GN=IFNGR1 PE=1 SV=1	14	5	54.4
A0A0A0MT64	NADPH:adrenodoxin oxidoreductase, mitochondrial OS=Homo sapiens GN=FDXR PE=1 SV=1	14	5	58.2
Q9Y3I0	tRNA-splicing ligase RtcB homolog OS=Homo sapiens GN=RTCB PE=1 SV=1	14	5	55.2
Q99943	1-acyl-sn-glycerol-3-phosphate acyltransferase alpha OS=Homo sapiens GN=AGPAT1 PE=1 SV=2	14	4	31.7
P17980	26S protease regulatory subunit 6A OS=Homo sapiens GN=PSMC3 PE=1 SV=3	14	4	49.2
Q6NUM9	All-trans-retinol 13,14-reductase OS=Homo sapiens GN=RETSAT PE=1 SV=2	14	4	66.8
I3L448	ATP synthase mitochondrial F1 complex assembly factor 1 OS=Homo sapiens GN=ATPAF1 PE=1 SV=1	14	4	38.8
P31785	Cytokine receptor common subunit gamma OS=Homo sapiens GN=IL2RG PE=1 SV=1	14	4	42.3
P46734	Dual specificity mitogen-activated protein kinase kinase 3 OS=Homo sapiens GN=MAP2K3 PE=1 SV=2	14	4	39.3
Q8TB36	Ganglioside-induced differentiation-associated protein 1 OS=Homo sapiens GN=GDAP1 PE=1 SV=3	14	4	41.3
P19256	Lymphocyte function-associated antigen 3 OS=Homo sapiens GN=CD58 PE=1 SV=1	14	4	28.1
Q7Z434	Mitochondrial antiviral-signaling protein OS=Homo sapiens GN=MAVS PE=1 SV=2	14	4	56.5
Q15223	Nectin-1 OS=Homo sapiens GN=NECTIN1 PE=1 SV=3	14	4	57.1

P49768	Presenilin-1 OS=Homo sapiens GN=PSEN1 PE=1 SV=1	14	4	52.6
H7C2I1	Protein arginine N-methyltransferase 1 OS=Homo sapiens GN=PRMT1 PE=1 SV=1	14	4	42.4
Q6PK18	2-oxoglutarate and iron-dependent oxygenase domain-containing protein 3 OS=Homo sapiens GN=OGFOD3 PE=1 SV=2	14	3	35.6
Q9NZE8	39S ribosomal protein L35, mitochondrial OS=Homo sapiens GN=MRPL35 PE=1 SV=3	14	3	21.5
Q9UQN3	Charged multivesicular body protein 2b OS=Homo sapiens GN=CHMP2B PE=1 SV=1	14	3	23.9
Q9Y6N1	Cytochrome c oxidase assembly protein COX11, mitochondrial OS=Homo sapiens GN=COX11 PE=1 SV=3	14	3	31.4
Q15049	Membrane protein MLC1 OS=Homo sapiens GN=MLC1 PE=1 SV=5	14	3	41.1
Q99733	Nucleosome assembly protein 1-like 4 OS=Homo sapiens GN=NAP1L4 PE=1 SV=1	14	3	42.8
P41231	P2Y purinoceptor 2 OS=Homo sapiens GN=P2RY2 PE=2 SV=4	14	3	42.2
F8W6M3	Palmitoyltransferase OS=Homo sapiens GN=ZDHHC3 PE=1 SV=1	14	3	37.8
Q9NQH7	Probable Xaa-Pro aminopeptidase 3 OS=Homo sapiens GN=XPNPEP3 PE=1 SV=1	14	3	57
P28074	Proteasome subunit beta type-5 OS=Homo sapiens GN=PSMB5 PE=1 SV=3	14	3	28.5
Q8N5M9	Protein jagunal homolog 1 OS=Homo sapiens GN=JAGN1 PE=1 SV=1	14	3	21.1
Q9UN36	Protein NDRG2 OS=Homo sapiens GN=NDRG2 PE=1 SV=2	14	3	40.8
C9JA93	TBC1 domain family member 15 (Fragment) OS=Homo sapiens GN=TBC1D15 PE=1 SV=1	14	3	32.1
Q96B77	Transmembrane protein 186 OS=Homo sapiens GN=TMEM186 PE=2 SV=1	14	3	24.9
Q969Z4	Tumor necrosis factor receptor superfamily member 19L OS=Homo sapiens GN=RELT PE=1 SV=1	14	3	46.1
E9PPM1	Tumor protein p53-inducible protein 11 OS=Homo sapiens GN=TP53I11 PE=1 SV=1	14	3	26
P47914	60S ribosomal protein L29 OS=Homo sapiens GN=RPL29 PE=1 SV=2	14	2	17.7
Q10589	Bone marrow stromal antigen 2 OS=Homo sapiens GN=BST2 PE=1 SV=1	14	2	19.8
P49006	MARCKS-related protein OS=Homo sapiens GN=MARCKSL1 PE=1 SV=2	14	2	19.5
Q9NRP0	Oligosaccharyltransferase complex subunit OSTC OS=Homo sapiens GN=OSTC PE=1 SV=1	14	2	16.8
Q9Y6E0	Serine/threonine-protein kinase 24 OS=Homo sapiens GN=STK24 PE=1 SV=1	14	2	49.3
H0Y9X1	Translation machinery-associated protein 16 (Fragment) OS=Homo sapiens GN=TMA16 PE=1 SV=1	14	2	27.7
P42356	Phosphatidylinositol 4-kinase alpha OS=Homo sapiens GN=PI4KA PE=1 SV=4	13	21	236.7
O94759	Transient receptor potential cation channel subfamily M member 2 OS=Homo sapiens GN=TRPM2 PE=1 SV=2	13	12	171.1
Q00722	1-phosphatidylinositol 4,5-bisphosphate phosphodiesterase beta-2 OS=Homo sapiens GN=PLCB2 PE=1 SV=2	13	10	133.9
Q96KP1	Exocyst complex component 2 OS=Homo sapiens GN=EXOC2 PE=1 SV=1	13	10	104

P23229	Integrin alpha-6 OS=Homo sapiens GN=ITGA6 PE=1 SV=5	13	10	126.5
Q15031	Probable leucine--tRNA ligase, mitochondrial OS=Homo sapiens GN=LARS2 PE=1 SV=2	13	9	101.9
O00461	Golgi integral membrane protein 4 OS=Homo sapiens GN=GOLIM4 PE=1 SV=1	13	8	81.8
O75326	Semaphorin-7A OS=Homo sapiens GN=SEMA7A PE=1 SV=1	13	8	74.8
Q8IU68	Transmembrane channel-like protein 8 OS=Homo sapiens GN=TMC8 PE=1 SV=1	13	8	81.6
D3DTX6	Neurabin-2 OS=Homo sapiens GN=PPP1R9B PE=1 SV=1	13	7	89.3
Q32P28	Prolyl 3-hydroxylase 1 OS=Homo sapiens GN=P3H1 PE=1 SV=2	13	7	83.3
E7EVH7	Uncharacterized protein OS=Homo sapiens PE=4 SV=1	13	7	83.6
O00400	Acetyl-coenzyme A transporter 1 OS=Homo sapiens GN=SLC33A1 PE=1 SV=1	13	6	60.9
Q9Y2L9	Leucine-rich repeat and calponin homology domain-containing protein 1 OS=Homo sapiens GN=LRCH1 PE=1 SV=3	13	6	80.8
P40763	Signal transducer and activator of transcription 3 OS=Homo sapiens GN=STAT3 PE=1 SV=2	13	6	88
Q9NS93	Transmembrane 7 superfamily member 3 OS=Homo sapiens GN=TM7SF3 PE=2 SV=1	13	6	64.1
Q9BVC6	Transmembrane protein 109 OS=Homo sapiens GN=TMEM109 PE=1 SV=1	13	6	26.2
P49961	Ectonucleoside triphosphate diphosphohydrolase 1 OS=Homo sapiens GN=ENTPD1 PE=1 SV=1	13	5	57.9
H0Y5K5	Endoplasmic reticulum-Golgi intermediate compartment protein 3 (Fragment) OS=Homo sapiens GN=ERGIC3 PE=1 SV=1	13	5	44.6
O60476	Mannosyl-oligosaccharide 1,2-alpha-mannosidase IB OS=Homo sapiens GN=MAN1A2 PE=1 SV=1	13	5	73
P03905	NADH-ubiquinone oxidoreductase chain 4 OS=Homo sapiens GN=MT-ND4 PE=1 SV=1	13	5	51.5
Q5T160	Probable arginine--tRNA ligase, mitochondrial OS=Homo sapiens GN=RARS2 PE=1 SV=1	13	5	65.5
Q5SWX8	Protein odr-4 homolog OS=Homo sapiens GN=ODR4 PE=1 SV=1	13	5	51.1
Q99519	Sialidase-1 OS=Homo sapiens GN=NEU1 PE=1 SV=1	13	5	45.4
P62191	26S protease regulatory subunit 4 OS=Homo sapiens GN=PSMC1 PE=1 SV=1	13	4	49.2
A0A0C4DFZ2	Arylsulfatase A OS=Homo sapiens GN=ARSA PE=1 SV=1	13	4	53.8
Q6KCM7	Calcium-binding mitochondrial carrier protein SCaMC-2 OS=Homo sapiens GN=SLC25A25 PE=1 SV=1	13	4	52.6
Q99467	CD180 antigen OS=Homo sapiens GN=CD180 PE=1 SV=2	13	4	74.1
Q13409	Cytoplasmic dynein 1 intermediate chain 2 OS=Homo sapiens GN=DYNC1I2 PE=1 SV=3	13	4	71.4
P32249	G-protein coupled receptor 183 OS=Homo sapiens GN=GPR183 PE=1 SV=3	13	4	41.2
Q9NQS5	G-protein coupled receptor 84 OS=Homo sapiens GN=GPR84 PE=2 SV=1	13	4	43.7

O60832	H/ACA ribonucleoprotein complex subunit 4 OS=Homo sapiens GN=DKC1 PE=1 SV=3	13	4	57.6
P09651	Heterogeneous nuclear ribonucleoprotein A1 OS=Homo sapiens GN=HNRNPA1 PE=1 SV=5	13	4	38.7
P20933	N(4)-(beta-N-acetylglucosaminyl)-L-asparaginase OS=Homo sapiens GN=AGA PE=1 SV=2	13	4	37.2
Q9BZW8	Natural killer cell receptor 2B4 OS=Homo sapiens GN=CD244 PE=1 SV=2	13	4	41.6
Q92968	Peroxisomal membrane protein PEX13 OS=Homo sapiens GN=PEX13 PE=1 SV=2	13	4	44.1
Q9NUL7	Probable ATP-dependent RNA helicase DDX28 OS=Homo sapiens GN=DDX28 PE=1 SV=2	13	4	59.5
Q08209	Serine/threonine-protein phosphatase 2B catalytic subunit alpha isoform OS=Homo sapiens GN=PPP3CA PE=1 SV=1	13	4	58.7
O43493	Trans-Golgi network integral membrane protein 2 OS=Homo sapiens GN=TGOLN2 PE=1 SV=2	13	4	51.1
Q99766	ATP synthase subunit s, mitochondrial OS=Homo sapiens GN=ATP5S PE=1 SV=3	13	3	24.9
Q9NW81	ATP synthase subunit s-like protein OS=Homo sapiens GN=ATP5SL PE=1 SV=3	13	3	29.2
Q5VW32	BRO1 domain-containing protein BROX OS=Homo sapiens GN=BROX PE=1 SV=1	13	3	46.4
Q7LBR1	Charged multivesicular body protein 1b OS=Homo sapiens GN=CHMP1B PE=1 SV=1	13	3	22.1
A0A0R4J2F2	Claudin domain-containing protein 1 OS=Homo sapiens GN=CLDND1 PE=1 SV=1	13	3	31
Q9Y240	C-type lectin domain family 11 member A OS=Homo sapiens GN=CLEC11A PE=1 SV=1	13	3	35.7
Q9BW60	Elongation of very long chain fatty acids protein 1 OS=Homo sapiens GN=ELOVL1 PE=1 SV=1	13	3	32.6
E9PI62	Mitochondrial GTPase 1 OS=Homo sapiens PE=3 SV=1	13	3	37.2
Q16539	Mitogen-activated protein kinase 14 OS=Homo sapiens GN=MAPK14 PE=1 SV=3	13	3	41.3
Q15743	Ovarian cancer G-protein coupled receptor 1 OS=Homo sapiens GN=GPR68 PE=1 SV=1	13	3	41.1
P28072	Proteasome subunit beta type-6 OS=Homo sapiens GN=PSMB6 PE=1 SV=4	13	3	25.3
Q9BSR8	Protein YIPF4 OS=Homo sapiens GN=YIPF4 PE=1 SV=1	13	3	27.1
P49247	Ribose-5-phosphate isomerase OS=Homo sapiens GN=RPIA PE=1 SV=3	13	3	33.2
Q13148	TAR DNA-binding protein 43 OS=Homo sapiens GN=TARDBP PE=1 SV=1	13	3	44.7
Q9H0E2	Toll-interacting protein OS=Homo sapiens GN=TOLLIP PE=1 SV=1	13	3	30.3
P25445	Tumor necrosis factor receptor superfamily member 6 OS=Homo sapiens GN=FAS PE=1 SV=1	13	3	37.7
Q96GC9	Vacuole membrane protein 1 OS=Homo sapiens GN=VMP1 PE=1 SV=1	13	3	46.2
J3KQN4	60S ribosomal protein L36a OS=Homo sapiens GN=RPL36A PE=1 SV=1	13	2	16.4
P61163	Alpha-centractin OS=Homo sapiens GN=ACTR1A PE=1 SV=1	13	2	42.6
P14209	CD99 antigen OS=Homo sapiens GN=CD99 PE=1 SV=1	13	2	18.8

Q96E22	Dehydrodolichyl diphosphate synthase complex subunit NUS1 OS=Homo sapiens GN=NUS1 PE=1 SV=1	13	2	33.2
P02042	Hemoglobin subunit delta OS=Homo sapiens GN=HBD PE=1 SV=2	13	2	16
Q9UJJ9	N-acetylglucosamine-1-phosphotransferase subunit gamma OS=Homo sapiens GN=GNPTG PE=1 SV=1	13	2	34
Q9NZQ7	Programmed cell death 1 ligand 1 OS=Homo sapiens GN=CD274 PE=1 SV=1	13	2	33.3
Q01081	Splicing factor U2AF 35 kDa subunit OS=Homo sapiens GN=U2AF1 PE=1 SV=3	13	2	27.9
Q6ZWK4	Uncharacterized protein C1orf186 OS=Homo sapiens GN=C1orf186 PE=2 SV=1	13	2	19.4
O75923	Dysferlin OS=Homo sapiens GN=DYSF PE=1 SV=1	12	14	237.1
Q00839	Heterogeneous nuclear ribonucleoprotein U OS=Homo sapiens GN=HNRNPU PE=1 SV=6	12	9	90.5
Q9P2B2	Prostaglandin F2 receptor negative regulator OS=Homo sapiens GN=PTGFRN PE=1 SV=2	12	9	98.5
Q16706	Alpha-mannosidase 2 OS=Homo sapiens GN=MAN2A1 PE=1 SV=2	12	8	131.1
Q92598	Heat shock protein 105 kDa OS=Homo sapiens GN=HSPH1 PE=1 SV=1	12	7	96.8
A0A0A0MSJ9	Ras GTPase-activating protein 2 OS=Homo sapiens GN=RASA2 PE=1 SV=1	12	7	96.9
O00159	Unconventional myosin-Ic OS=Homo sapiens GN=MYO1C PE=1 SV=4	12	7	121.6
P07384	Calpain-1 catalytic subunit OS=Homo sapiens GN=CAPN1 PE=1 SV=1	12	6	81.8
Q9H4E7	Differentially expressed in FDCP 6 homolog OS=Homo sapiens GN=DEF6 PE=1 SV=1	12	6	73.9
Q68DH5	LMBR1 domain-containing protein 2 OS=Homo sapiens GN=LMBRD2 PE=1 SV=1	12	6	81.1
A0A087WZ62	Mannosyltransferase OS=Homo sapiens PE=3 SV=1	12	6	95.9
Q6ZUJ8	Phosphoinositide 3-kinase adapter protein 1 OS=Homo sapiens GN=PIK3AP1 PE=1 SV=2	12	6	90.3
Q96B97	SH3 domain-containing kinase-binding protein 1 OS=Homo sapiens GN=SH3KBP1 PE=1 SV=2	12	6	73.1
Q9UHB9	Signal recognition particle subunit SRP68 OS=Homo sapiens GN=SRP68 PE=1 SV=2	12	6	70.7
P54577	Tyrosine--tRNA ligase, cytoplasmic OS=Homo sapiens GN=YARS PE=1 SV=4	12	6	59.1
Q9NYY8	FAST kinase domain-containing protein 2, mitochondrial OS=Homo sapiens GN=FASTKD2 PE=1 SV=1	12	5	81.4
P48723	Heat shock 70 kDa protein 13 OS=Homo sapiens GN=HSPA13 PE=1 SV=1	12	5	51.9
O43566	Regulator of G-protein signaling 14 OS=Homo sapiens GN=RGS14 PE=1 SV=4	12	5	61.4
F8WF69	Clathrin light chain OS=Homo sapiens GN=CLTA PE=1 SV=1	12	4	27.8
O75063	Glycosaminoglycan xylosylkinase OS=Homo sapiens GN=FAM20B PE=1 SV=1	12	4	46.4
K7ER00	Phenylalanine--tRNA ligase alpha subunit OS=Homo sapiens GN=FARSA PE=1 SV=1	12	4	62.4
P78356	Phosphatidylinositol 5-phosphate 4-kinase type-2 beta OS=Homo sapiens GN=PIP4K2B PE=1 SV=1	12	4	47.3

A0A0C4DG49	Poliovirus receptor OS=Homo sapiens GN=PVR PE=1 SV=1	12	4	45.3
Q9H0X4	Protein FAM234A OS=Homo sapiens GN=FAM234A PE=1 SV=1	12	4	59.6
P17252	Protein kinase C alpha type OS=Homo sapiens GN=PRKCA PE=1 SV=4	12	4	76.7
Q13043	Serine/threonine-protein kinase 4 OS=Homo sapiens GN=STK4 PE=1 SV=2	12	4	55.6
Q8IYJ3	Synaptotagmin-like protein 1 OS=Homo sapiens GN=SYTL1 PE=1 SV=1	12	4	61.8
P42081	T-lymphocyte activation antigen CD86 OS=Homo sapiens GN=CD86 PE=1 SV=2	12	4	37.7
P00749	Urokinase-type plasminogen activator OS=Homo sapiens GN=PLAU PE=1 SV=2	12	4	48.5
P51665	26S proteasome non-ATPase regulatory subunit 7 OS=Homo sapiens GN=PSMD7 PE=1 SV=2	12	3	37
P20160	Azurocidin OS=Homo sapiens GN=AZU1 PE=1 SV=3	12	3	26.9
P68400	Casein kinase II subunit alpha OS=Homo sapiens GN=CSNK2A1 PE=1 SV=1	12	3	45.1
Q11206	CMP-N-acetylneuraminate-beta-galactosamide-alpha-2,3-sialyltransferase 4 OS=Homo sapiens GN=ST3GAL4 PE=2 SV=1	12	3	38
O00303	Eukaryotic translation initiation factor 3 subunit F OS=Homo sapiens GN=EIF3F PE=1 SV=1	12	3	37.5
O00182	Galectin-9 OS=Homo sapiens GN=LGALS9 PE=1 SV=2	12	3	39.5
Q16543	Hsp90 co-chaperone Cdc37 OS=Homo sapiens GN=CDC37 PE=1 SV=1	12	3	44.4
Q15404	Ras suppressor protein 1 OS=Homo sapiens GN=RSU1 PE=1 SV=3	12	3	31.5
E9PGQ0	Solute carrier family 25 member 44 OS=Homo sapiens GN=SLC25A44 PE=1 SV=3	12	3	36.3
J3QLM1	StAR-related lipid transfer protein 3 OS=Homo sapiens GN=STARD3 PE=1 SV=1	12	3	52.5
O43760	Synaptogyrin-2 OS=Homo sapiens GN=SYNGR2 PE=1 SV=1	12	3	24.8
Q9NP79	Vacuolar protein sorting-associated protein VTA1 homolog OS=Homo sapiens GN=VTA1 PE=1 SV=1	12	3	33.9
Q9NPF2	Carbohydrate sulfotransferase 11 OS=Homo sapiens GN=CHST11 PE=1 SV=1	12	2	41.5
E7EVJ5	Cytoplasmic FMR1-interacting protein 2 OS=Homo sapiens GN=CYFIP2 PE=1 SV=1	12	2	142.5
Q8WXX5	DnaJ homolog subfamily C member 9 OS=Homo sapiens GN=DNAJC9 PE=1 SV=1	12	2	29.9
Q9NY12	H/ACA ribonucleoprotein complex subunit 1 OS=Homo sapiens GN=GAR1 PE=1 SV=1	12	2	22.3
Q9Y584	Mitochondrial import inner membrane translocase subunit Tim22 OS=Homo sapiens GN=TIMM22 PE=1 SV=2	12	2	20
P68402	Platelet-activating factor acetylhydrolase IB subunit beta OS=Homo sapiens GN=PAFAH1B2 PE=1 SV=1	12	2	25.6
O95070	Protein YIF1A OS=Homo sapiens GN=YIF1A PE=1 SV=2	12	2	32
Q9BRF8	Serine/threonine-protein phosphatase CPPED1 OS=Homo sapiens GN=CPPED1 PE=1 SV=3	12	2	35.5
O60493	Sorting nexin-3 OS=Homo sapiens GN=SNX3 PE=1 SV=3	12	2	18.8

Q9UN37	Vacuolar protein sorting-associated protein 4A OS=Homo sapiens GN=VPS4A PE=1 SV=1	12	2	48.9
Q9ULH0	Kinase D-interacting substrate of 220 kDa OS=Homo sapiens GN=KIDINS220 PE=1 SV=3	11	15	196.4
Q9UBG0	C-type mannose receptor 2 OS=Homo sapiens GN=MRC2 PE=1 SV=2	11	10	166.6
Q0VD83	Apolipoprotein B receptor OS=Homo sapiens GN=APOBR PE=1 SV=2	11	8	114.8
P16070	CD44 antigen OS=Homo sapiens GN=CD44 PE=1 SV=3	11	8	81.5
P55786	Puromycin-sensitive aminopeptidase OS=Homo sapiens GN=NPEPPS PE=1 SV=2	11	8	103.2
Q93034	Cullin-5 OS=Homo sapiens GN=CUL5 PE=1 SV=4	11	7	90.9
P33992	DNA replication licensing factor MCM5 OS=Homo sapiens GN=MCM5 PE=1 SV=5	11	7	82.2
O15117	FYN-binding protein OS=Homo sapiens GN=FYB PE=1 SV=2	11	7	85.3
O14964	Hepatocyte growth factor-regulated tyrosine kinase substrate OS=Homo sapiens GN=HGS PE=1 SV=1	11	7	86.1
A0A087X0X3	Heterogeneous nuclear ribonucleoprotein M OS=Homo sapiens GN=HNRNPM PE=1 SV=1	11	7	77.5
O00291	Huntingtin-interacting protein 1 OS=Homo sapiens GN=HIP1 PE=1 SV=5	11	7	116.1
Q14764	Major vault protein OS=Homo sapiens GN=MVP PE=1 SV=4	11	7	99.3
P53985	Monocarboxylate transporter 1 OS=Homo sapiens GN=SLC16A1 PE=1 SV=3	11	7	53.9
P32418	Sodium/calcium exchanger 1 OS=Homo sapiens GN=SLC8A1 PE=1 SV=3	11	7	108.5
Q7Z2W4	Zinc finger CCCH-type antiviral protein 1 OS=Homo sapiens GN=ZC3HAV1 PE=1 SV=3	11	7	101.4
Q8IWA5	Choline transporter-like protein 2 OS=Homo sapiens GN=SLC44A2 PE=1 SV=3	11	6	80.1
Q14258	E3 ubiquitin/ISG15 ligase TRIM25 OS=Homo sapiens GN=TRIM25 PE=1 SV=2	11	6	70.9
Q08AM6	Protein VAC14 homolog OS=Homo sapiens GN=VAC14 PE=1 SV=1	11	6	87.9
Q8N392	Rho GTPase-activating protein 18 OS=Homo sapiens GN=ARHGAP18 PE=1 SV=3	11	6	74.9
Q8N697	Solute carrier family 15 member 4 OS=Homo sapiens GN=SLC15A4 PE=1 SV=1	11	6	62
Q4KMP7	TBC1 domain family member 10B OS=Homo sapiens GN=TBC1D10B PE=1 SV=3	11	6	87.1
Q9NUB1	Acetyl-coenzyme A synthetase 2-like, mitochondrial OS=Homo sapiens GN=ACSS1 PE=1 SV=2	11	5	74.8
Q86TM6	E3 ubiquitin-protein ligase synoviolin OS=Homo sapiens GN=SYVN1 PE=1 SV=2	11	5	67.6
Q16658	Fascin OS=Homo sapiens GN=FSCN1 PE=1 SV=3	11	5	54.5
Q01650	Large neutral amino acids transporter small subunit 1 OS=Homo sapiens GN=SLC7A5 PE=1 SV=2	11	5	55
Q6UWP7	Lysocardiolipin acyltransferase 1 OS=Homo sapiens GN=LCLAT1 PE=1 SV=1	11	5	48.9
Q6P1A2	Lysophospholipid acyltransferase 5 OS=Homo sapiens GN=LPCAT3 PE=1 SV=1	11	5	56

Q92692	Nectin-2 OS=Homo sapiens GN=NECTIN2 PE=1 SV=1	11	5	57.7
Q5TEJ8	Protein THEMIS2 OS=Homo sapiens GN=THEMIS2 PE=1 SV=1	11	5	72
Q9H1C4	Protein unc-93 homolog B1 OS=Homo sapiens GN=UNC93B1 PE=1 SV=2	11	5	66.6
Q15052	Rho guanine nucleotide exchange factor 6 OS=Homo sapiens GN=ARHGEF6 PE=1 SV=2	11	5	87.4
Q969Y2	tRNA modification GTPase GTPBP3, mitochondrial OS=Homo sapiens GN=GTPBP3 PE=1 SV=2	11	5	52
Q14642	Type I inositol 1,4,5-trisphosphate 5-phosphatase OS=Homo sapiens GN=INPP5A PE=1 SV=1	11	5	47.8
Q8IYS2	Uncharacterized protein KIAA2013 OS=Homo sapiens GN=KIAA2013 PE=1 SV=1	11	5	69.1
Q7L591	Docking protein 3 OS=Homo sapiens GN=DOK3 PE=1 SV=2	11	4	53.3
Q2TAA5	GDP-Man:Man(3)GlcNAc(2)-PP-Dol alpha-1,2-mannosyltransferase OS=Homo sapiens GN=ALG11 PE=1 SV=2	11	4	55.6
O15554	Intermediate conductance calcium-activated potassium channel protein 4 OS=Homo sapiens GN=KCNN4 PE=1 SV=1	11	4	47.7
Q96AQ8	Mitochondrial calcium uniporter regulator 1 OS=Homo sapiens GN=MCUR1 PE=1 SV=1	11	4	39.7
Q9HB40	Retinoid-inducible serine carboxypeptidase OS=Homo sapiens GN=SCPEP1 PE=1 SV=1	11	4	50.8
Q8WVM8	Sec1 family domain-containing protein 1 OS=Homo sapiens GN=SCFD1 PE=1 SV=4	11	4	72.3
Q5T5C7	Serine--tRNA ligase, cytoplasmic OS=Homo sapiens GN=SARS PE=1 SV=1	11	4	61.3
Q96LC7	Sialic acid-binding Ig-like lectin 10 OS=Homo sapiens GN=SIGLEC10 PE=1 SV=3	11	4	76.5
Q9NRA2	Sialin OS=Homo sapiens GN=SLC17A5 PE=1 SV=2	11	4	54.6
Q92536	Y+L amino acid transporter 2 OS=Homo sapiens GN=SLC7A6 PE=1 SV=3	11	4	56.8
Q9UH62	Armadillo repeat-containing X-linked protein 3 OS=Homo sapiens GN=ARMCX3 PE=1 SV=1	11	3	42.5
P48509	CD151 antigen OS=Homo sapiens GN=CD151 PE=1 SV=3	11	3	28.3
Q5ZPR3	CD276 antigen OS=Homo sapiens GN=CD276 PE=1 SV=1	11	3	57.2
Q9Y295	Developmentally-regulated GTP-binding protein 1 OS=Homo sapiens GN=DRG1 PE=1 SV=1	11	3	40.5
O60496	Docking protein 2 OS=Homo sapiens GN=DOK2 PE=1 SV=2	11	3	45.4
O75521	Enoyl-CoA delta isomerase 2, mitochondrial OS=Homo sapiens GN=ECI2 PE=1 SV=4	11	3	43.6
P41091	Eukaryotic translation initiation factor 2 subunit 3 OS=Homo sapiens GN=EIF2S3 PE=1 SV=3	11	3	51.1
B3KS98	Eukaryotic translation initiation factor 3 subunit H OS=Homo sapiens GN=EIF3S3 PE=1 SV=1	11	3	41.6
A4D1E9	GTP-binding protein 10 OS=Homo sapiens GN=GTPBP10 PE=1 SV=1	11	3	42.9
P07910	Heterogeneous nuclear ribonucleoproteins C1/C2 OS=Homo sapiens GN=HNRNPC PE=1 SV=4	11	3	33.7
P16401	Histone H1.5 OS=Homo sapiens GN=HIST1H1B PE=1 SV=3	11	3	22.6

Q9UIJ5	Palmitoyltransferase ZDHHC2 OS=Homo sapiens GN=ZDHHC2 PE=1 SV=1	11	3	42
Q8N2U9	PQ-loop repeat-containing protein 1 OS=Homo sapiens GN=PQLC1 PE=1 SV=1	11	3	30.5
F6WQW2	Ran-specific GTPase-activating protein OS=Homo sapiens GN=RANBP1 PE=1 SV=1	11	3	31.9
Q96I51	RCC1-like G exchanging factor-like protein OS=Homo sapiens GN=RCC1L PE=1 SV=2	11	3	50
Q9HC36	rRNA methyltransferase 3, mitochondrial OS=Homo sapiens GN=MRM3 PE=1 SV=2	11	3	47
J3KTL2	Serine/arginine-rich-splicing factor 1 OS=Homo sapiens GN=SRSF1 PE=1 SV=1	11	3	28.3
Q96CQ1	Solute carrier family 25 member 36 OS=Homo sapiens GN=SLC25A36 PE=1 SV=1	11	3	34.3
Q6DKK2	Tetratricopeptide repeat protein 19, mitochondrial OS=Homo sapiens GN=TTC19 PE=1 SV=4	11	3	42.4
Q96QE5	Transcription elongation factor, mitochondrial OS=Homo sapiens GN=TEFM PE=1 SV=1	11	3	41.7
P23258	Tubulin gamma-1 chain OS=Homo sapiens GN=TUBG1 PE=1 SV=2	11	3	51.1
Q9NT62	Ubiquitin-like-conjugating enzyme ATG3 OS=Homo sapiens GN=ATG3 PE=1 SV=1	11	3	35.8
Q969T9	WW domain-binding protein 2 OS=Homo sapiens GN=WBP2 PE=1 SV=1	11	3	28.1
Q9NQW7	Xaa-Pro aminopeptidase 1 OS=Homo sapiens GN=XPNPEP1 PE=1 SV=3	11	3	69.9
Q8NEW0	Zinc transporter 7 OS=Homo sapiens GN=SLC30A7 PE=2 SV=1	11	3	41.6
O43427	Acidic fibroblast growth factor intracellular-binding protein OS=Homo sapiens GN=FIBP PE=1 SV=3	11	2	41.9
P14550	Alcohol dehydrogenase [NADP(+)] OS=Homo sapiens GN=AKR1A1 PE=1 SV=3	11	2	36.6
Q8TCZ2	CD99 antigen-like protein 2 OS=Homo sapiens GN=CD99L2 PE=1 SV=1	11	2	28
P09417	Dihydropteridine reductase OS=Homo sapiens GN=QDPR PE=1 SV=2	11	2	25.8
P38919	Eukaryotic initiation factor 4A-III OS=Homo sapiens GN=EIF4A3 PE=1 SV=4	11	2	46.8
Q92522	Histone H1x OS=Homo sapiens GN=H1FX PE=1 SV=1	11	2	22.5
A0A0G2JMH6	HLA class II histocompatibility antigen, DR alpha chain OS=Homo sapiens GN=HLA-DRA PE=1 SV=1	11	2	28.6
P02533	Keratin, type I cytoskeletal 14 OS=Homo sapiens GN=KRT14 PE=1 SV=4	11	2	51.5
P02750	Leucine-rich alpha-2-glycoprotein OS=Homo sapiens GN=LRG1 PE=1 SV=2	11	2	38.2
Q7L9L4	MOB kinase activator 1B OS=Homo sapiens GN=MOB1B PE=1 SV=3	11	2	25.1
P41247	Patatin-like phospholipase domain-containing protein 4 OS=Homo sapiens GN=PNPLA4 PE=2 SV=3	11	2	28
Q5JUQ0	Protein FAM78A OS=Homo sapiens GN=FAM78A PE=2 SV=1	11	2	31.9
O14521	Succinate dehydrogenase [ubiquinone] cytochrome b small subunit, mitochondrial OS=Homo sapiens GN=SDHD PE=1 SV=1	11	2	17
P13726	Tissue factor OS=Homo sapiens GN=F3 PE=1 SV=1	11	2	33

Q9NX00	Transmembrane protein 160 OS=Homo sapiens GN=TMEM160 PE=1 SV=1	11	2	19.6
P42345	Serine/threonine-protein kinase mTOR OS=Homo sapiens GN=MTOR PE=1 SV=1	10	16	288.7
Q96BY6	Dedicator of cytokinesis protein 10 OS=Homo sapiens GN=DOCK10 PE=1 SV=3	10	14	249.4
Q8TF62	Probable phospholipid-transporting ATPase IM OS=Homo sapiens GN=ATP8B4 PE=1 SV=3	10	9	135.8
P26640	Valine--tRNA ligase OS=Homo sapiens GN=VARS PE=1 SV=4	10	9	140.4
O95427	GPI ethanolamine phosphate transferase 1 OS=Homo sapiens GN=PIGN PE=1 SV=1	10	7	105.7
Q9H330	Transmembrane protein 245 OS=Homo sapiens GN=TMEM245 PE=1 SV=2	10	7	100.9
O43264	Centromere/kinetochore protein zw10 homolog OS=Homo sapiens GN=ZW10 PE=1 SV=3	10	6	88.8
Q9UBC2	Epidermal growth factor receptor substrate 15-like 1 OS=Homo sapiens GN=EPS15L1 PE=1 SV=1	10	6	94.2
J3KMZ9	Low-density lipoprotein receptor (Fragment) OS=Homo sapiens GN=LDLR PE=1 SV=1	10	6	104.6
B0YIW6	Archain 1, isoform CRA_a OS=Homo sapiens GN=ARCN1 PE=1 SV=1	10	5	61.6
Q9P2E5	Chondroitin sulfate glucuronyltransferase OS=Homo sapiens GN=CHPF2 PE=2 SV=2	10	5	85.9
Q92611	ER degradation-enhancing alpha-mannosidase-like protein 1 OS=Homo sapiens GN=EDEM1 PE=1 SV=1	10	5	73.7
Q6P1M0	Long-chain fatty acid transport protein 4 OS=Homo sapiens GN=SLC27A4 PE=1 SV=1	10	5	72
Q9C0B5	Palmitoyltransferase ZDHHC5 OS=Homo sapiens GN=ZDHHC5 PE=1 SV=2	10	5	77.5
Q9NSD9	Phenylalanine--tRNA ligase beta subunit OS=Homo sapiens GN=FARSB PE=1 SV=3	10	5	66.1
E7ETB3	Aspartyl aminopeptidase OS=Homo sapiens GN=DNPEP PE=1 SV=2	10	4	54.5
B7ZL88	COX18 protein OS=Homo sapiens GN=COX18 PE=1 SV=1	10	4	37.1
P15509	Granulocyte-macrophage colony-stimulating factor receptor subunit alpha OS=Homo sapiens GN=CSF2RA PE=1 SV=1	10	4	46.2
Q9UK23	N-acetylglucosamine-1-phosphodiester alpha-N-acetylglucosaminidase OS=Homo sapiens GN=NAGPA PE=1 SV=2	10	4	56
O00567	Nucleolar protein 56 OS=Homo sapiens GN=NOP56 PE=1 SV=4	10	4	66
P05771	Protein kinase C beta type OS=Homo sapiens GN=PRKCB PE=1 SV=4	10	4	76.8
H3BMF4	Protein spinster homolog 1 OS=Homo sapiens GN=SPNS1 PE=1 SV=1	10	4	61.1
Q9UGQ3	Solute carrier family 2, facilitated glucose transporter member 6 OS=Homo sapiens GN=SLC2A6 PE=1 SV=2	10	4	54.5
Q7Z4G4	tRNA (guanine(10)-N2)-methyltransferase homolog OS=Homo sapiens GN=TRMT11 PE=1 SV=1	10	4	53.4
P49753	Acyl-coenzyme A thioesterase 2, mitochondrial OS=Homo sapiens GN=ACOT2 PE=1 SV=6	10	3	53.2
Q9BW91	ADP-ribose pyrophosphatase, mitochondrial OS=Homo sapiens GN=NUDT9 PE=1 SV=1	10	3	39.1

Q6JBY9	CapZ-interacting protein OS=Homo sapiens GN=RCSD1 PE=1 SV=1	10	3	44.5
Q9Y375	Complex I intermediate-associated protein 30, mitochondrial OS=Homo sapiens GN=NDUFAF1 PE=1 SV=2	10	3	37.7
Q6UXH1	Cysteine-rich with EGF-like domain protein 2 OS=Homo sapiens GN=CRELD2 PE=1 SV=1	10	3	38.2
P05198	Eukaryotic translation initiation factor 2 subunit 1 OS=Homo sapiens GN=EIF2S1 PE=1 SV=3	10	3	36.1
D6RBW1	Eukaryotic translation initiation factor 4E OS=Homo sapiens GN=EIF4E PE=1 SV=1	10	3	28.5
Q86VR2	Protein FAM134C OS=Homo sapiens GN=FAM134C PE=1 SV=1	10	3	51.4
Q8NBI5	Solute carrier family 43 member 3 OS=Homo sapiens GN=SLC43A3 PE=1 SV=2	10	3	54.5
Q16881	Thioredoxin reductase 1, cytoplasmic OS=Homo sapiens GN=TXNRD1 PE=1 SV=3	10	3	70.9
Q15125	3-beta-hydroxysteroid-Delta(8),Delta(7)-isomerase OS=Homo sapiens GN=EBP PE=1 SV=3	10	2	26.3
Q5TEU4	Arginine-hydroxylase NDUFAF5, mitochondrial OS=Homo sapiens GN=NDUFAF5 PE=1 SV=1	10	2	38.9
Q13867	Bleomycin hydrolase OS=Homo sapiens GN=BLMH PE=1 SV=1	10	2	52.5
A0A024QZP7	Cell division cycle 2, G1 to S and G2 to M, isoform CRA_a OS=Homo sapiens GN=CDC2 PE=1 SV=1	10	2	34.1
Q9Y6K0	Choline/ethanolaminephosphotransferase 1 OS=Homo sapiens GN=CEPT1 PE=1 SV=1	10	2	46.5
F8WCZ3	Coiled-coil domain-containing protein 115 OS=Homo sapiens GN=CCDC115 PE=1 SV=1	10	2	21.1
Q9BUN8	Derlin-1 OS=Homo sapiens GN=DERL1 PE=1 SV=1	10	2	28.8
O14972	Down syndrome critical region protein 3 OS=Homo sapiens GN=DSCR3 PE=2 SV=1	10	2	33
Q96DT0	Galectin-12 OS=Homo sapiens GN=LGALS12 PE=1 SV=1	10	2	37.5
Q9UBK5	Hematopoietic cell signal transducer OS=Homo sapiens GN=HCST PE=1 SV=1	10	2	9.5
Q14213	Interleukin-27 subunit beta OS=Homo sapiens GN=EBI3 PE=1 SV=2	10	2	25.4
Q16719	Kynureninase OS=Homo sapiens GN=KYNU PE=1 SV=1	10	2	52.3
Q6NUT3	Major facilitator superfamily domain-containing protein 12 OS=Homo sapiens GN=MFSD12 PE=1 SV=2	10	2	52
A0A087WZT2	Methyltransferase-like protein 7B OS=Homo sapiens GN=METTL7B PE=1 SV=1	10	2	31.3
Q9H019	Mitochondrial fission regulator 1-like OS=Homo sapiens GN=MTFR1L PE=1 SV=2	10	2	31.9
Q15388	Mitochondrial import receptor subunit TOM20 homolog OS=Homo sapiens GN=TOMM20 PE=1 SV=1	10	2	16.3
O95772	MLN64 N-terminal domain homolog OS=Homo sapiens GN=STARD3NL PE=1 SV=1	10	2	26.6
A0A087WV17	Osteoclast-associated immunoglobulin-like receptor OS=Homo sapiens GN=OSCAR PE=1 SV=1	10	2	30.8
P51575	P2X purinoceptor 1 OS=Homo sapiens GN=P2RX1 PE=1 SV=1	10	2	45
Q9UBY8	Protein CLN8 OS=Homo sapiens GN=CLN8 PE=1 SV=3	10	2	32.8

E7EQY1	Protein FAM136A OS=Homo sapiens GN=FAM136A PE=1 SV=1	10	2	26.8
Q96CM3	RNA pseudouridylate synthase domain-containing protein 4 OS=Homo sapiens GN=RPUSD4 PE=1 SV=1	10	2	42.2
A0A087X1N8	Serpin B6 OS=Homo sapiens GN=SERPINB6 PE=1 SV=1	10	2	44.8
Q8IXU6	Solute carrier family 35 member F2 OS=Homo sapiens GN=SLC35F2 PE=1 SV=1	10	2	41.2
A0A0A0MSY7	Thymidine kinase 2, mitochondrial OS=Homo sapiens GN=TK2 PE=1 SV=1	10	2	33.3
Q9Y296	Trafficking protein particle complex subunit 4 OS=Homo sapiens GN=TRAPPC4 PE=1 SV=1	10	2	24.3
Q5BJF2	Transmembrane protein 97 OS=Homo sapiens GN=TMEM97 PE=1 SV=1	10	2	20.8
Q5LJA9	Ubiquitin carboxyl-terminal hydrolase (Fragment) OS=Homo sapiens GN=UCHL5 PE=1 SV=1	10	2	41.7
Q04323	UBX domain-containing protein 1 OS=Homo sapiens GN=UBXN1 PE=1 SV=2	10	2	33.3
Q9NZ43	Vesicle transport protein USE1 OS=Homo sapiens GN=USE1 PE=1 SV=2	10	2	29.4
A6NIW2	Dedicator of cytokinesis protein 11 OS=Homo sapiens GN=DOCK11 PE=1 SV=2	9	15	237.9
Q7L2E3	Putative ATP-dependent RNA helicase DHX30 OS=Homo sapiens GN=DHX30 PE=1 SV=1	9	8	133.9
Q6P2E9	Enhancer of mRNA-decapping protein 4 OS=Homo sapiens GN=EDC4 PE=1 SV=1	9	7	151.6
Q8NB49	Phospholipid-transporting ATPase IG OS=Homo sapiens GN=ATP11C PE=1 SV=3	9	7	129.4
B8ZZD4	Tax1-binding protein 1 OS=Homo sapiens GN=TAX1BP1 PE=1 SV=1	9	7	93.6
Q6JQN1	Acyl-CoA dehydrogenase family member 10 OS=Homo sapiens GN=ACAD10 PE=1 SV=1	9	6	118.8
Q13574	Diacylglycerol kinase zeta OS=Homo sapiens GN=DGKZ PE=1 SV=3	9	6	124.1
Q53EP0	Fibronectin type III domain-containing protein 3B OS=Homo sapiens GN=FNDC3B PE=1 SV=2	9	6	132.8
Q8TBA6	Golgin subfamily A member 5 OS=Homo sapiens GN=GOLGA5 PE=1 SV=3	9	6	83
Q8WUM9	Sodium-dependent phosphate transporter 1 OS=Homo sapiens GN=SLC20A1 PE=1 SV=1	9	6	73.7
A0A0A0MSI8	Exocyst complex component 5 OS=Homo sapiens GN=EXOC5 PE=1 SV=1	9	5	82.1
Q7L8L6	FAST kinase domain-containing protein 5, mitochondrial OS=Homo sapiens GN=FASTKD5 PE=1 SV=1	9	5	86.5
Q9P107	GEM-interacting protein OS=Homo sapiens GN=GMIP PE=1 SV=2	9	5	106.6
Q8TEQ8	GPI ethanolamine phosphate transferase 3 OS=Homo sapiens GN=PIGO PE=1 SV=3	9	5	118.6
P19338	Nucleolin OS=Homo sapiens GN=NCL PE=1 SV=3	9	5	76.6
Q68EM7	Rho GTPase-activating protein 17 OS=Homo sapiens GN=ARHGAP17 PE=1 SV=1	9	5	95.4
P19634	Sodium/hydrogen exchanger 1 OS=Homo sapiens GN=SLC9A1 PE=1 SV=2	9	5	90.7
P53794	Sodium/myo-inositol cotransporter OS=Homo sapiens GN=SLC5A3 PE=1 SV=2	9	5	79.6

Q9UH65	Switch-associated protein 70 OS=Homo sapiens GN=SWAP70 PE=1 SV=1	9	5	69
P38435	Vitamin K-dependent gamma-carboxylase OS=Homo sapiens GN=GGCX PE=1 SV=2	9	5	87.5
Q86YZ3	Hornerin OS=Homo sapiens GN=HRNR PE=1 SV=2	9	4	282.2
Q01581	Hydroxymethylglutaryl-CoA synthase, cytoplasmic OS=Homo sapiens GN=HMGCS1 PE=1 SV=2	9	4	57.3
P20592	Interferon-induced GTP-binding protein Mx2 OS=Homo sapiens GN=MX2 PE=1 SV=1	9	4	82
Q6PCB7	Long-chain fatty acid transport protein 1 OS=Homo sapiens GN=SLC27A1 PE=2 SV=1	9	4	71.1
X6R3N0	Long-chain fatty acid transport protein 3 OS=Homo sapiens GN=SLC27A3 PE=1 SV=1	9	4	87.3
P15529	Membrane cofactor protein OS=Homo sapiens GN=CD46 PE=1 SV=3	9	4	43.7
Q9BTX1	Nucleoporin NDC1 OS=Homo sapiens GN=NDC1 PE=1 SV=2	9	4	76.3
Q9BVG9	Phosphatidylserine synthase 2 OS=Homo sapiens GN=PTDSS2 PE=1 SV=1	9	4	56.2
Q9Y289	Sodium-dependent multivitamin transporter OS=Homo sapiens GN=SLC5A6 PE=2 SV=2	9	4	68.6
O00391	Sulfhydryl oxidase 1 OS=Homo sapiens GN=QSOX1 PE=1 SV=3	9	4	82.5
Q8N442	Translation factor GUF1, mitochondrial OS=Homo sapiens GN=GUF1 PE=1 SV=1	9	4	74.3
E7EUC7	UTP--glucose-1-phosphate uridylyltransferase OS=Homo sapiens GN=UGP2 PE=1 SV=1	9	4	57.8
Q04446	1,4-alpha-glucan-branching enzyme OS=Homo sapiens GN=GBE1 PE=1 SV=3	9	3	80.4
P43686	26S protease regulatory subunit 6B OS=Homo sapiens GN=PSMC4 PE=1 SV=2	9	3	47.3
P48556	26S proteasome non-ATPase regulatory subunit 8 OS=Homo sapiens GN=PSMD8 PE=1 SV=2	9	3	39.6
P02649	Apolipoprotein E OS=Homo sapiens GN=APOE PE=1 SV=1	9	3	36.1
Q8N5I2	Arrestin domain-containing protein 1 OS=Homo sapiens GN=ARRDC1 PE=1 SV=1	9	3	46
O00478	Butyrophilin subfamily 3 member A3 OS=Homo sapiens GN=BTN3A3 PE=1 SV=1	9	3	65
P21730	C5a anaphylatoxin chemotactic receptor 1 OS=Homo sapiens GN=C5AR1 PE=1 SV=2	9	3	39.3
Q9BV79	Enoyl-[acyl-carrier-protein] reductase, mitochondrial OS=Homo sapiens GN=MECR PE=1 SV=2	9	3	40.4
Q53EU6	Glycerol-3-phosphate acyltransferase 3 OS=Homo sapiens GN=GPAT3 PE=1 SV=2	9	3	48.7
Q14103	Heterogeneous nuclear ribonucleoprotein D0 OS=Homo sapiens GN=HNRNPD PE=1 SV=1	9	3	38.4
O15229	Kynurenine 3-monooxygenase OS=Homo sapiens GN=KMO PE=1 SV=2	9	3	55.8
Q08722	Leukocyte surface antigen CD47 OS=Homo sapiens GN=CD47 PE=1 SV=1	9	3	35.2
Q9BQA1	Methylosome protein 50 OS=Homo sapiens GN=WDR77 PE=1 SV=1	9	3	36.7
Q9H4K7	Mitochondrial ribosome-associated GTPase 2 OS=Homo sapiens GN=MTG2 PE=1 SV=1	9	3	43.9

P03886	NADH-ubiquinone oxidoreductase chain 1 OS=Homo sapiens GN=MT-ND1 PE=1 SV=1	9	3	35.6
Q86YC3	Negative regulator of reactive oxygen species OS=Homo sapiens GN=NRROS PE=1 SV=1	9	3	76.3
Q9H490	Phosphatidylinositol glycan anchor biosynthesis class U protein OS=Homo sapiens GN=PIGU PE=1 SV=3	9	3	50
Q96G03	Phosphoglucomutase-2 OS=Homo sapiens GN=PGM2 PE=1 SV=4	9	3	68.2
J3KTA4	Probable ATP-dependent RNA helicase DDX5 OS=Homo sapiens GN=DDX5 PE=1 SV=1	9	3	69
Q7LDG7	RAS guanyl-releasing protein 2 OS=Homo sapiens GN=RASGRP2 PE=1 SV=1	9	3	69.2
Q96DB5	Regulator of microtubule dynamics protein 1 OS=Homo sapiens GN=RMDN1 PE=1 SV=1	9	3	35.8
O76021	Ribosomal L1 domain-containing protein 1 OS=Homo sapiens GN=RSL1D1 PE=1 SV=3	9	3	54.9
P51812	Ribosomal protein S6 kinase alpha-3 OS=Homo sapiens GN=RPS6KA3 PE=1 SV=1	9	3	83.7
H3BPE7	RNA-binding protein FUS OS=Homo sapiens GN=FUS PE=1 SV=1	9	3	53.5
Q96QD8	Sodium-coupled neutral amino acid transporter 2 OS=Homo sapiens GN=SLC38A2 PE=1 SV=2	9	3	56
Q8WUX1	Sodium-coupled neutral amino acid transporter 5 OS=Homo sapiens GN=SLC38A5 PE=1 SV=1	9	3	51.4
Q7L5N1	COP9 signalosome complex subunit 6 OS=Homo sapiens GN=COPS6 PE=1 SV=1	9	2	36.1
P61073	C-X-C chemokine receptor type 4 OS=Homo sapiens GN=CXCR4 PE=1 SV=1	9	2	39.7
Q00535	Cyclin-dependent-like kinase 5 OS=Homo sapiens GN=CDK5 PE=1 SV=3	9	2	33.3
P49184	Deoxyribonuclease-1-like 1 OS=Homo sapiens GN=DNASE1L1 PE=1 SV=1	9	2	33.9
Q99961	Endophilin-A2 OS=Homo sapiens GN=SH3GL1 PE=1 SV=1	9	2	41.5
A0A087WU02	Endoplasmic reticulum-Golgi intermediate compartment protein 2 OS=Homo sapiens GN=ERGIC2 PE=1 SV=1	9	2	43.4
Q7L2H7	Eukaryotic translation initiation factor 3 subunit M OS=Homo sapiens GN=EIF3M PE=1 SV=1	9	2	42.5
P31942	Heterogeneous nuclear ribonucleoprotein H3 OS=Homo sapiens GN=HNRNPH3 PE=1 SV=2	9	2	36.9
Q9UJ70	N-acetyl-D-glucosamine kinase OS=Homo sapiens GN=NAGK PE=1 SV=4	9	2	37.4
Q9NXA8	NAD-dependent protein deacylase sirtuin-5, mitochondrial OS=Homo sapiens GN=SIRT5 PE=1 SV=2	9	2	33.9
O95178	NADH dehydrogenase [ubiquinone] 1 beta subcomplex subunit 2, mitochondrial OS=Homo sapiens GN=NDUFB2 PE=1 SV=1	9	2	12.1
Q9HAN9	Nicotinamide/nicotinic acid mononucleotide adenylyltransferase 1 OS=Homo sapiens GN=NMNAT1 PE=1 SV=1	9	2	31.9
Q9GZT8	NIF3-like protein 1 OS=Homo sapiens GN=NIF3L1 PE=1 SV=2	9	2	41.9
O43808	Peroxisomal membrane protein PMP34 OS=Homo sapiens GN=SLC25A17 PE=1 SV=1	9	2	34.5
F5H2B5	Phospholipase D4 OS=Homo sapiens GN=PLD4 PE=1 SV=1	9	2	56.3

Q9H4B0	Probable tRNA N6-adenosine threonylcarbamoyltransferase, mitochondrial OS=Homo sapiens GN=OSGEPL1 PE=2 SV=2	9	2	45.1
P28065	Proteasome subunit beta type-9 OS=Homo sapiens GN=PSMB9 PE=1 SV=2	9	2	23.3
Q15436	Protein transport protein Sec23A OS=Homo sapiens GN=SEC23A PE=1 SV=2	9	2	86.1
E7EQR8	Protein YIPF3 OS=Homo sapiens GN=YIPF3 PE=1 SV=1	9	2	38.9
Q969E2	Secretory carrier-associated membrane protein 4 OS=Homo sapiens GN=SCAMP4 PE=1 SV=1	9	2	25.7
Q00796	Sorbitol dehydrogenase OS=Homo sapiens GN=SORD PE=1 SV=4	9	2	38.3
Q9Y5W9	Sorting nexin-11 OS=Homo sapiens GN=SNX11 PE=1 SV=2	9	2	30.4
A0A0A0MR05	Target of rapamycin complex subunit LST8 OS=Homo sapiens GN=MLST8 PE=1 SV=1	9	2	36
Q9H813	Transmembrane protein 206 OS=Homo sapiens GN=TMEM206 PE=1 SV=1	9	2	40
Q96HV5	Transmembrane protein 41A OS=Homo sapiens GN=TMEM41A PE=1 SV=1	9	2	29.6
Q5BJD5	Transmembrane protein 41B OS=Homo sapiens GN=TMEM41B PE=1 SV=1	9	2	32.5
Q5VWC8	Very-long-chain (3R)-3-hydroxyacyl-CoA dehydratase 4 OS=Homo sapiens GN=HACD4 PE=1 SV=1	9	2	27.5
Q96N67	Dedicator of cytokinesis protein 7 OS=Homo sapiens GN=DOCK7 PE=1 SV=4	8	11	242.4
P01024	Complement C3 OS=Homo sapiens GN=C3 PE=1 SV=2	8	10	187
Q96RT1	Erbin OS=Homo sapiens GN=ERBIN PE=1 SV=2	8	9	158.2
O60449	Lymphocyte antigen 75 OS=Homo sapiens GN=LY75 PE=1 SV=3	8	9	198.2
Q13045	Protein flightless-1 homolog OS=Homo sapiens GN=FLII PE=1 SV=2	8	7	144.7
P55011	Solute carrier family 12 member 2 OS=Homo sapiens GN=SLC12A2 PE=1 SV=1	8	7	131.4
F5H2F4	C-1-tetrahydrofolate synthase, cytoplasmic OS=Homo sapiens GN=MTHFD1 PE=1 SV=1	8	6	110.5
K7EM85	Rho GTPase-activating protein 45 OS=Homo sapiens GN=ARHGAP45 PE=1 SV=1	8	6	127.5
Q9ULT0	Tetratricopeptide repeat protein 7A OS=Homo sapiens GN=TTC7A PE=1 SV=3	8	6	96.1
Q9BZH6	WD repeat-containing protein 11 OS=Homo sapiens GN=WDR11 PE=1 SV=1	8	6	136.6
C9JIF9	Acylamino-acid-releasing enzyme OS=Homo sapiens GN=APEH PE=1 SV=1	8	5	81.6
Q06481	Amyloid-like protein 2 OS=Homo sapiens GN=APLP2 PE=1 SV=2	8	5	86.9
Q5H8A4	GPI ethanolamine phosphate transferase 2 OS=Homo sapiens GN=PIGG PE=1 SV=1	8	5	108.1
Q96AA3	Protein RFT1 homolog OS=Homo sapiens GN=RFT1 PE=1 SV=1	8	5	60.3
P17812	CTP synthase 1 OS=Homo sapiens GN=CTPS1 PE=1 SV=2	8	4	66.6
Q13618	Cullin-3 OS=Homo sapiens GN=CUL3 PE=1 SV=2	8	4	88.9

O14681	Etoposide-induced protein 2.4 homolog OS=Homo sapiens GN=EI24 PE=1 SV=4	8	4	38.9
P30825	High affinity cationic amino acid transporter 1 OS=Homo sapiens GN=SLC7A1 PE=1 SV=1	8	4	67.6
Q99797	Mitochondrial intermediate peptidase OS=Homo sapiens GN=MIPEP PE=1 SV=2	8	4	80.6
P42224	Signal transducer and activator of transcription 1-alpha/beta OS=Homo sapiens GN=STAT1 PE=1 SV=2	8	4	87.3
Q8N0X7	Spartin OS=Homo sapiens GN=SPG20 PE=1 SV=1	8	4	72.8
Q15025	TNFAIP3-interacting protein 1 OS=Homo sapiens GN=TNIP1 PE=1 SV=2	8	4	71.8
Q14956	Transmembrane glycoprotein NMB OS=Homo sapiens GN=GPNMB PE=1 SV=2	8	4	63.9
Q3MIX3	Uncharacterized aarF domain-containing protein kinase 5 OS=Homo sapiens GN=ADCK5 PE=1 SV=2	8	4	65.9
A0A087WXL6	Vacuolar protein sorting 11 (Yeast), isoform CRA_a OS=Homo sapiens GN=VPS11 PE=1 SV=1	8	4	107.7
Q86Y34	Adhesion G protein-coupled receptor G3 OS=Homo sapiens GN=ADGRG3 PE=1 SV=1	8	3	60.8
Q7Z6M3	Allergin-1 OS=Homo sapiens GN=MILR1 PE=1 SV=2	8	3	38.7
Q9UPY5	Cystine/glutamate transporter OS=Homo sapiens GN=SLC7A11 PE=1 SV=1	8	3	55.4
P49902	Cytosolic purine 5'-nucleotidase OS=Homo sapiens GN=NT5C2 PE=1 SV=1	8	3	64.9
Q8N465	D-2-hydroxyglutarate dehydrogenase, mitochondrial OS=Homo sapiens GN=D2HGDH PE=1 SV=3	8	3	56.4
Q8TBM8	DnaJ homolog subfamily B member 14 OS=Homo sapiens GN=DNAJB14 PE=1 SV=1	8	3	42.5
U3KQS2	Glucose-6-phosphate exchanger SLC37A4 OS=Homo sapiens GN=SLC37A4 PE=1 SV=2	8	3	48.9
P0CG08	Golgi pH regulator B OS=Homo sapiens GN=GPR89B PE=1 SV=1	8	3	52.9
Q8IWB1	Inositol 1,4,5-trisphosphate receptor-interacting protein OS=Homo sapiens GN=ITPRIP PE=1 SV=1	8	3	62
O43813	LanC-like protein 1 OS=Homo sapiens GN=LANCL1 PE=1 SV=1	8	3	45.3
A0A0G2JMC9	Leukocyte immunoglobulin-like receptor subfamily A member 2 OS=Homo sapiens GN=LILRA2 PE=1 SV=1	8	3	53.4
Q643R3	Lysophospholipid acyltransferase LPCAT4 OS=Homo sapiens GN=LPCAT4 PE=1 SV=1	8	3	57.2
Q4ZIN3	Membralin OS=Homo sapiens GN=TMEM259 PE=1 SV=1	8	3	67.8
Q8NHP6	Motile sperm domain-containing protein 2 OS=Homo sapiens GN=MOSPD2 PE=1 SV=1	8	3	59.7
P26196	Probable ATP-dependent RNA helicase DDX6 OS=Homo sapiens GN=DDX6 PE=1 SV=2	8	3	54.4
Q9NQL2	Ras-related GTP-binding protein D OS=Homo sapiens GN=RRAGD PE=1 SV=1	8	3	45.6
Q9BXS9	Solute carrier family 26 member 6 OS=Homo sapiens GN=SLC26A6 PE=1 SV=1	8	3	82.9
Q9UMX0	Ubiquilin-1 OS=Homo sapiens GN=UBQLN1 PE=1 SV=2	8	3	62.5
Q7L1V2	Vacuolar fusion protein MON1 homolog B OS=Homo sapiens GN=MON1B PE=1 SV=1	8	3	59.2

Q9P2A4	ABI gene family member 3 OS=Homo sapiens GN=ABI3 PE=1 SV=2	8	2	39
Q96A54	Adiponectin receptor protein 1 OS=Homo sapiens GN=ADIPOR1 PE=1 SV=1	8	2	42.6
O00170	AH receptor-interacting protein OS=Homo sapiens GN=AIP PE=1 SV=2	8	2	37.6
Q12904	Aminoacyl tRNA synthase complex-interacting multifunctional protein 1 OS=Homo sapiens GN=AIMP1 PE=1 SV=2	8	2	34.3
P09525	Annexin A4 OS=Homo sapiens GN=ANXA4 PE=1 SV=4	8	2	35.9
A0A087WW23	Arachidonate 5-lipoxygenase-activating protein OS=Homo sapiens GN=ALOX5AP PE=1 SV=1	8	2	24.2
Q8N5M1	ATP synthase mitochondrial F1 complex assembly factor 2 OS=Homo sapiens GN=ATPAF2 PE=1 SV=1	8	2	32.8
P00846	ATP synthase subunit a OS=Homo sapiens GN=MT-ATP6 PE=1 SV=1	8	2	24.8
Q9Y256	CAAX prenyl protease 2 OS=Homo sapiens GN=RCE1 PE=1 SV=1	8	2	35.8
P08962	CD63 antigen OS=Homo sapiens GN=CD63 PE=1 SV=2	8	2	25.6
P00395	Cytochrome c oxidase subunit 1 OS=Homo sapiens GN=MT-CO1 PE=1 SV=1	8	2	57
Q8WVM0	Dimethyladenosine transferase 1, mitochondrial OS=Homo sapiens GN=TFB1M PE=1 SV=1	8	2	39.5
Q9BSW2	EF-hand calcium-binding domain-containing protein 4B OS=Homo sapiens GN=CRACR2A PE=1 SV=1	8	2	45.6
Q9H223	EH domain-containing protein 4 OS=Homo sapiens GN=EHD4 PE=1 SV=1	8	2	61.1
P21462	fMet-Leu-Phe receptor OS=Homo sapiens GN=FPR1 PE=1 SV=3	8	2	38.4
Q9NZC3	Glycerophosphodiester phosphodiesterase 1 OS=Homo sapiens GN=GDE1 PE=1 SV=1	8	2	37.7
P52597	Heterogeneous nuclear ribonucleoprotein F OS=Homo sapiens GN=HNRNPF PE=1 SV=3	8	2	45.6
Q07666	KH domain-containing, RNA-binding, signal transduction-associated protein 1 OS=Homo sapiens GN=KHDRBS1 PE=1 SV=1	8	2	48.2
Q5SVS4	Kidney mitochondrial carrier protein 1 OS=Homo sapiens GN=SLC25A30 PE=1 SV=1	8	2	32.5
P04156	Major prion protein OS=Homo sapiens GN=PRNP PE=1 SV=1	8	2	27.6
Q969M1	Mitochondrial import receptor subunit TOM40B OS=Homo sapiens GN=TOMM40L PE=1 SV=1	8	2	33.9
P28482	Mitogen-activated protein kinase 1 OS=Homo sapiens GN=MAPK1 PE=1 SV=3	8	2	41.4
Q495G5	MMAA protein OS=Homo sapiens GN=MMAA PE=1 SV=1	8	2	47.1
Q86WC4	Osteopetrosis-associated transmembrane protein 1 OS=Homo sapiens GN=OSTM1 PE=1 SV=1	8	2	37.2
I3L1I5	Putative uncharacterized protein LOC100996504 OS=Homo sapiens PE=5 SV=1	8	2	41.2
Q9Y3F4	Serine-threonine kinase receptor-associated protein OS=Homo sapiens GN=STRAP PE=1 SV=1	8	2	38.4
Q96GZ6	Solute carrier family 41 member 3 OS=Homo sapiens GN=SLC41A3 PE=1 SV=2	8	2	54.7

A0PJW6	Transmembrane protein 223 OS=Homo sapiens GN=TMEM223 PE=1 SV=1	8	2	22
Q8N0Z8	tRNA pseudouridine synthase-like 1 OS=Homo sapiens GN=PUSL1 PE=1 SV=1	8	2	33.2
H7BYZ3	Uncharacterized protein OS=Homo sapiens PE=4 SV=1	8	2	36.9
Q14152	Eukaryotic translation initiation factor 3 subunit A OS=Homo sapiens GN=EIF3A PE=1 SV=1	7	9	166.5
Q5VZU9	Tripeptidyl-peptidase 2 OS=Homo sapiens GN=TPP2 PE=1 SV=1	7	8	139.7
Q86YS7	C2 domain-containing protein 5 OS=Homo sapiens GN=C2CD5 PE=1 SV=1	7	6	110.4
Q9BZQ6	ER degradation-enhancing alpha-mannosidase-like protein 3 OS=Homo sapiens GN=EDEM3 PE=1 SV=2	7	6	104.6
Q8N1F8	Serine/threonine-protein kinase 11-interacting protein OS=Homo sapiens GN=STK11IP PE=1 SV=3	7	6	121.3
Q8NE71	ATP-binding cassette sub-family F member 1 OS=Homo sapiens GN=ABCF1 PE=1 SV=2	7	5	95.9
Q8N370	Large neutral amino acids transporter small subunit 4 OS=Homo sapiens GN=SLC43A2 PE=1 SV=1	7	5	62.7
Q14BN4	Sarcolemmal membrane-associated protein OS=Homo sapiens GN=SLMAP PE=1 SV=1	7	5	95.1
Q7Z403	Transmembrane channel-like protein 6 OS=Homo sapiens GN=TMC6 PE=1 SV=2	7	5	90
Q9H4A4	Aminopeptidase B OS=Homo sapiens GN=RNPEP PE=1 SV=2	7	4	72.5
Q9Y678	Coatomer subunit gamma-1 OS=Homo sapiens GN=COPG1 PE=1 SV=1	7	4	97.7
A0A0B4J1V8	HCG2039996 OS=Homo sapiens GN=PPAN-P2RY11 PE=3 SV=1	7	4	87.9
Q86WA8	Lon protease homolog 2, peroxisomal OS=Homo sapiens GN=LONP2 PE=1 SV=1	7	4	94.6
Q6P4Q7	Metal transporter CNNM4 OS=Homo sapiens GN=CNNM4 PE=1 SV=3	7	4	86.6
Q6DN12	Multiple C2 and transmembrane domain-containing protein 2 OS=Homo sapiens GN=MCTP2 PE=1 SV=3	7	4	99.5
Q9BYT8	Neurolysin, mitochondrial OS=Homo sapiens GN=NLN PE=1 SV=1	7	4	80.6
Q9NR30	Nucleolar RNA helicase 2 OS=Homo sapiens GN=DDX21 PE=1 SV=5	7	4	87.3
Q9C0H2	Protein tweety homolog 3 OS=Homo sapiens GN=TTYH3 PE=1 SV=3	7	4	57.5
Q9H3U1	Protein unc-45 homolog A OS=Homo sapiens GN=UNC45A PE=1 SV=1	7	4	103
P49754	Vacuolar protein sorting-associated protein 41 homolog OS=Homo sapiens GN=VPS41 PE=1 SV=3	7	4	98.5
O43865	Adenosylhomocysteinase 2 OS=Homo sapiens GN=AHCYL1 PE=1 SV=2	7	3	58.9
A2A2G4	Alpha-1,3-glucosyltransferase OS=Homo sapiens GN=ALG6 PE=1 SV=1	7	3	58.3
Q7Z5R6	Amyloid beta A4 precursor protein-binding family B member 1-interacting protein OS=Homo sapiens GN=APBB1IP PE=1 SV=1	7	3	73.1
A0A0A0MT11	Calcium/calmodulin-dependent protein kinase type II subunit gamma OS=Homo sapiens GN=CAMK2G PE=1 SV=1	7	3	62.6

P52429	Diacylglycerol kinase epsilon OS=Homo sapiens GN=DGKE PE=1 SV=1	7	3	63.9
Q9UHN1	DNA polymerase subunit gamma-2, mitochondrial OS=Homo sapiens GN=POLG2 PE=1 SV=1	7	3	54.9
Q02750	Dual specificity mitogen-activated protein kinase kinase 1 OS=Homo sapiens GN=MAP2K1 PE=1 SV=2	7	3	43.4
B0QY89	Eukaryotic translation initiation factor 3 subunit L OS=Homo sapiens GN=EIF3L PE=1 SV=1	7	3	70.9
Q8TED4	Glucose-6-phosphate exchanger SLC37A2 OS=Homo sapiens GN=SLC37A2 PE=2 SV=2	7	3	54.4
P34932	Heat shock 70 kDa protein 4 OS=Homo sapiens GN=HSPA4 PE=1 SV=4	7	3	94.3
Q8NC56	LEM domain-containing protein 2 OS=Homo sapiens GN=LEMD2 PE=1 SV=1	7	3	56.9
P49279	Natural resistance-associated macrophage protein 1 OS=Homo sapiens GN=SLC11A1 PE=2 SV=1	7	3	59.8
Q14435	Polypeptide N-acetylgalactosaminyltransferase 3 OS=Homo sapiens GN=GALNT3 PE=1 SV=2	7	3	72.6
Q7L592	Protein arginine methyltransferase NDUFAF7, mitochondrial OS=Homo sapiens GN=NDUFAF7 PE=1 SV=1	7	3	49.2
Q9UGH3	Solute carrier family 23 member 2 OS=Homo sapiens GN=SLC23A2 PE=1 SV=1	7	3	70.3
Q13838	Spliceosome RNA helicase DDX39B OS=Homo sapiens GN=DDX39B PE=1 SV=1	7	3	49
Q9NX78	Transmembrane protein 260 OS=Homo sapiens GN=TMEM260 PE=2 SV=3	7	3	79.5
P45974	Ubiquitin carboxyl-terminal hydrolase 5 OS=Homo sapiens GN=USP5 PE=1 SV=2	7	3	95.7
P12955	Xaa-Pro dipeptidase OS=Homo sapiens GN=PEPD PE=1 SV=3	7	3	54.5
Q9BRK5	45 kDa calcium-binding protein OS=Homo sapiens GN=SDF4 PE=1 SV=1	7	2	41.8
Q9UQ53	Alpha-1,3-mannosyl-glycoprotein 4-beta-N-acetylglucosaminyltransferase B OS=Homo sapiens GN=MGAT4B PE=1 SV=1	7	2	63.2
Q13286	Battenin OS=Homo sapiens GN=CLN3 PE=1 SV=1	7	2	47.6
P54687	Branched-chain-amino-acid aminotransferase, cytosolic OS=Homo sapiens GN=BCAT1 PE=1 SV=3	7	2	42.9
Q96D31	Calcium release-activated calcium channel protein 1 OS=Homo sapiens GN=ORAI1 PE=1 SV=2	7	2	32.6
Q9NWR8	Calcium uniporter regulatory subunit MCUb, mitochondrial OS=Homo sapiens GN=MCUB PE=1 SV=2	7	2	39.1
O14595	Carboxy-terminal domain RNA polymerase II polypeptide A small phosphatase 2 OS=Homo sapiens GN=CTDSP2 PE=1 SV=2	7	2	30.6
Q9BSQ5	Cerebral cavernous malformations 2 protein OS=Homo sapiens GN=CCM2 PE=1 SV=1	7	2	48.8
Q9Y3E7	Charged multivesicular body protein 3 OS=Homo sapiens GN=CHMP3 PE=1 SV=3	7	2	25.1
A6NI79	Coiled-coil domain-containing protein 69 OS=Homo sapiens GN=CCDC69 PE=1 SV=1	7	2	34.8
Q8NDZ4	Deleted in autism protein 1 OS=Homo sapiens GN=C3orf58 PE=1 SV=1	7	2	49.5
O00115	Deoxyribonuclease-2-alpha OS=Homo sapiens GN=DNASE2 PE=1 SV=2	7	2	39.6

A0A0J9YX62	DnaJ homolog subfamily B member 6 OS=Homo sapiens GN=DNAJB6 PE=1 SV=1	7	2	36.6
Q13561	Dynactin subunit 2 OS=Homo sapiens GN=DCTN2 PE=1 SV=4	7	2	44.2
Q9NYP7	Elongation of very long chain fatty acids protein 5 OS=Homo sapiens GN=ELOVL5 PE=1 SV=1	7	2	35.3
A0A087WW40	Endophilin-B1 OS=Homo sapiens GN=SH3GLB1 PE=1 SV=1	7	2	44.2
Q9HBU6	Ethanolamine kinase 1 OS=Homo sapiens GN=ETNK1 PE=1 SV=1	7	2	50.9
Q9UKB1	F-box/WD repeat-containing protein 11 OS=Homo sapiens GN=FBXW11 PE=1 SV=1	7	2	62.1
Q9BZ67	FERM domain-containing protein 8 OS=Homo sapiens GN=FRMD8 PE=1 SV=1	7	2	51.2
P50440	Glycine amidinotransferase, mitochondrial OS=Homo sapiens GN=GATM PE=1 SV=1	7	2	48.4
Q9H8Y8	Golgi reassembly-stacking protein 2 OS=Homo sapiens GN=GORASP2 PE=1 SV=3	7	2	47.1
P52789	Hexokinase-2 OS=Homo sapiens GN=HK2 PE=1 SV=2	7	2	102.3
O95297	Myelin protein zero-like protein 1 OS=Homo sapiens GN=MPZL1 PE=1 SV=1	7	2	29.1
Q68D85	Natural cytotoxicity triggering receptor 3 ligand 1 OS=Homo sapiens GN=NCR3LG1 PE=1 SV=1	7	2	50.8
Q9Y6K9	NF-kappa-B essential modulator OS=Homo sapiens GN=IKBKG PE=1 SV=2	7	2	48.2
Q15233	Non-POU domain-containing octamer-binding protein OS=Homo sapiens GN=NONO PE=1 SV=4	7	2	54.2
O75570	Peptide chain release factor 1, mitochondrial OS=Homo sapiens GN=MTRF1 PE=1 SV=2	7	2	52.3
Q13093	Platelet-activating factor acetylhydrolase OS=Homo sapiens GN=PLA2G7 PE=1 SV=1	7	2	50
P25105	Platelet-activating factor receptor OS=Homo sapiens GN=PTAFR PE=1 SV=1	7	2	39.2
Q9NVV4	Poly(A) RNA polymerase, mitochondrial OS=Homo sapiens GN=MTPAP PE=1 SV=1	7	2	66.1
Q7Z2H8	Proton-coupled amino acid transporter 1 OS=Homo sapiens GN=SLC36A1 PE=1 SV=1	7	2	53
Q96TC7	Regulator of microtubule dynamics protein 3 OS=Homo sapiens GN=RMDN3 PE=1 SV=2	7	2	52.1
Q9NRX5	Serine incorporator 1 OS=Homo sapiens GN=SERINC1 PE=1 SV=1	7	2	50.5
Q9P0V8	SLAM family member 8 OS=Homo sapiens GN=SLAMF8 PE=1 SV=1	7	2	31.6
Q96AG3	Solute carrier family 25 member 46 OS=Homo sapiens GN=SLC25A46 PE=1 SV=1	7	2	46.1
P23246	Splicing factor, proline- and glutamine-rich OS=Homo sapiens GN=SFPQ PE=1 SV=2	7	2	76.1
P51649	Succinate-semialdehyde dehydrogenase, mitochondrial OS=Homo sapiens GN=ALDH5A1 PE=1 SV=2	7	2	57.2
Q99816	Tumor susceptibility gene 101 protein OS=Homo sapiens GN=TSG101 PE=1 SV=2	7	2	43.9
P54727	UV excision repair protein RAD23 homolog B OS=Homo sapiens GN=RAD23B PE=1 SV=1	7	2	43.1
O95562	Vesicle transport protein SFT2B OS=Homo sapiens GN=SFT2D2 PE=1 SV=1	7	2	17.8

Q92616	eIF-2-alpha kinase activator GCN1 OS=Homo sapiens GN=GCN1 PE=1 SV=6	6	10	292.6
E7ER45	Maltase-glucoamylase, intestinal OS=Homo sapiens GN=MGAM PE=1 SV=2	6	8	311.8
Q9NZM3	Intersectin-2 OS=Homo sapiens GN=ITSN2 PE=1 SV=3	6	7	193.3
Q96P48	Arf-GAP with Rho-GAP domain, ANK repeat and PH domain-containing protein 1 OS=Homo sapiens GN=ARAP1 PE=1 SV=3	6	6	162.1
Q9UQE7	Structural maintenance of chromosomes protein 3 OS=Homo sapiens GN=SMC3 PE=1 SV=2	6	6	141.5
A0A087X256	WASH complex subunit 4 OS=Homo sapiens GN=WASHC4 PE=1 SV=1	6	6	136.4
Q01970	1-phosphatidylinositol 4,5-bisphosphate phosphodiesterase beta-3 OS=Homo sapiens GN=PLCB3 PE=1 SV=2	6	5	138.7
O00411	DNA-directed RNA polymerase, mitochondrial OS=Homo sapiens GN=POLRMT PE=1 SV=2	6	5	138.5
O75694	Nuclear pore complex protein Nup155 OS=Homo sapiens GN=NUP155 PE=1 SV=1	6	5	155.1
O14974	Protein phosphatase 1 regulatory subunit 12A OS=Homo sapiens GN=PPP1R12A PE=1 SV=1	6	5	115.2
Q15057	Arf-GAP with coiled-coil, ANK repeat and PH domain-containing protein 2 OS=Homo sapiens GN=ACAP2 PE=1 SV=3	6	4	88
Q9ULG6	Cell cycle progression protein 1 OS=Homo sapiens GN=CCPG1 PE=1 SV=3	6	4	87.3
B5ME19	Eukaryotic translation initiation factor 3 subunit C-like protein OS=Homo sapiens GN=EIF3CL PE=3 SV=1	6	4	105.4
Q9NV70	Exocyst complex component 1 OS=Homo sapiens GN=EXOC1 PE=1 SV=4	6	4	101.9
O14980	Exportin-1 OS=Homo sapiens GN=XPO1 PE=1 SV=1	6	4	123.3
Q96F46	Interleukin-17 receptor A OS=Homo sapiens GN=IL17RA PE=1 SV=2	6	4	96.1
Q9HBR0	Putative sodium-coupled neutral amino acid transporter 10 OS=Homo sapiens GN=SLC38A10 PE=1 SV=2	6	4	119.7
Q8N8A2	Serine/threonine-protein phosphatase 6 regulatory ankyrin repeat subunit B OS=Homo sapiens GN=ANKRD44 PE=1 SV=3	6	4	107.5
A0A0G2JNW7	Solute carrier family 12 member 7 OS=Homo sapiens GN=SLC12A7 PE=1 SV=1	6	4	119.1
P22732	Solute carrier family 2, facilitated glucose transporter member 5 OS=Homo sapiens GN=SLC2A5 PE=1 SV=1	6	4	54.9
A0A087WY96	Transporter OS=Homo sapiens GN=SLC6A6 PE=1 SV=1	6	4	81.3
Q15029	116 kDa U5 small nuclear ribonucleoprotein component OS=Homo sapiens GN=EFTUD2 PE=1 SV=1	6	3	109.4
Q99460	26S proteasome non-ATPase regulatory subunit 1 OS=Homo sapiens GN=PSMD1 PE=1 SV=2	6	3	105.8
Q5T8D3	Acyl-CoA-binding domain-containing protein 5 OS=Homo sapiens GN=ACBD5 PE=1 SV=1	6	3	60.1
Q86X52	Chondroitin sulfate synthase 1 OS=Homo sapiens GN=CHSY1 PE=1 SV=3	6	3	91.7
P33993	DNA replication licensing factor MCM7 OS=Homo sapiens GN=MCM7 PE=1 SV=4	6	3	81.3
P41440	Folate transporter 1 OS=Homo sapiens GN=SLC19A1 PE=1 SV=3	6	3	64.8

Q6ZVF9	G protein-regulated inducer of neurite outgrowth 3 OS=Homo sapiens GN=GPRIN3 PE=2 SV=2	6	3	82.4
P06756	Integrin alpha-V OS=Homo sapiens GN=ITGAV PE=1 SV=2	6	3	116
O43278	Kunitz-type protease inhibitor 1 OS=Homo sapiens GN=SPINT1 PE=1 SV=2	6	3	58.4
B4E0T2	Peroxisomal targeting signal 1 receptor OS=Homo sapiens GN=PEX5 PE=1 SV=1	6	3	73.1
P00747	Plasminogen OS=Homo sapiens GN=PLG PE=1 SV=2	6	3	90.5
Q9P258	Protein RCC2 OS=Homo sapiens GN=RCC2 PE=1 SV=2	6	3	56
Q7L7X3	Serine/threonine-protein kinase TAO1 OS=Homo sapiens GN=TAOK1 PE=1 SV=1	6	3	116
Q9UPN7	Serine/threonine-protein phosphatase 6 regulatory subunit 1 OS=Homo sapiens GN=PPP6R1 PE=1 SV=5	6	3	96.7
C9JBG5	SID1 transmembrane family member 2 OS=Homo sapiens GN=SIDT2 PE=1 SV=2	6	3	96.7
Q9Y5Y6	Suppressor of tumorigenicity 14 protein OS=Homo sapiens GN=ST14 PE=1 SV=2	6	3	94.7
P86790	Vacuolar fusion protein CCZ1 homolog B OS=Homo sapiens GN=CCZ1B PE=1 SV=1	6	3	55.8
P04004	Vitronectin OS=Homo sapiens GN=VTN PE=1 SV=1	6	3	54.3
A4D1P6	WD repeat-containing protein 91 OS=Homo sapiens GN=WDR91 PE=1 SV=2	6	3	83.3
A0A087X188	Bridging integrator 2 OS=Homo sapiens GN=BIN2 PE=1 SV=1	6	2	65
P17655	Calpain-2 catalytic subunit OS=Homo sapiens GN=CAPN2 PE=1 SV=6	6	2	79.9
A0A024R6X1	Carboxylic ester hydrolase OS=Homo sapiens GN=CES2 PE=1 SV=1	6	2	68.9
Q8N7R7	Cyclin-Y-like protein 1 OS=Homo sapiens GN=CCNYL1 PE=1 SV=2	6	2	40.7
Q9H5Q4	Dimethyladenosine transferase 2, mitochondrial OS=Homo sapiens GN=TFB2M PE=1 SV=1	6	2	45.3
O75071	EF-hand calcium-binding domain-containing protein 14 OS=Homo sapiens GN=EFCAB14 PE=1 SV=1	6	2	55
Q8NFF5	FAD synthase OS=Homo sapiens GN=FLAD1 PE=1 SV=1	6	2	65.2
Q9Y5Y0	Feline leukemia virus subgroup C receptor-related protein 1 OS=Homo sapiens GN=FLVCR1 PE=1 SV=1	6	2	59.8
P16930	Fumarylacetoacetase OS=Homo sapiens GN=FAH PE=1 SV=2	6	2	46.3
P13807	Glycogen [starch] synthase, muscle OS=Homo sapiens GN=GYS1 PE=1 SV=2	6	2	83.7
P46976	Glycogenin-1 OS=Homo sapiens GN=GYG1 PE=1 SV=4	6	2	39.4
Q68CQ7	Glycosyltransferase 8 domain-containing protein 1 OS=Homo sapiens GN=GLT8D1 PE=1 SV=2	6	2	41.9
P51991	Heterogeneous nuclear ribonucleoprotein A3 OS=Homo sapiens GN=HNRNPA3 PE=1 SV=2	6	2	39.6
Q92769	Histone deacetylase 2 OS=Homo sapiens GN=HDAC2 PE=1 SV=2	6	2	55.3
Q9BYI3	Hyccin OS=Homo sapiens GN=FAM126A PE=1 SV=2	6	2	57.6

Q12905	Interleukin enhancer-binding factor 2 OS=Homo sapiens GN=ILF2 PE=1 SV=2	6	2	43
A0A0D9SGG1	Lymphocyte cytosolic protein 2 OS=Homo sapiens GN=LCP2 PE=1 SV=1	6	2	60.1
Q9H3U5	Major facilitator superfamily domain-containing protein 1 OS=Homo sapiens GN=MFSD1 PE=2 SV=2	6	2	51.2
Q8N8R3	Mitochondrial basic amino acids transporter OS=Homo sapiens GN=SLC25A29 PE=1 SV=2	6	2	32
Q9NTG7	NAD-dependent protein deacetylase sirtuin-3, mitochondrial OS=Homo sapiens GN=SIRT3 PE=1 SV=2	6	2	43.5
Q7Z3B4	Nucleoporin p54 OS=Homo sapiens GN=NUP54 PE=1 SV=2	6	2	55.4
J3KQ32	Obg-like ATPase 1 OS=Homo sapiens GN=OLA1 PE=1 SV=1	6	2	46.9
Q9BTY2	Plasma alpha-L-fucosidase OS=Homo sapiens GN=FUCA2 PE=1 SV=2	6	2	54
P16671	Platelet glycoprotein 4 OS=Homo sapiens GN=CD36 PE=1 SV=2	6	2	53
Q96EC8	Protein YIPF6 OS=Homo sapiens GN=YIPF6 PE=1 SV=2	6	2	26.2
Q14242	P-selectin glycoprotein ligand 1 OS=Homo sapiens GN=SELPLG PE=1 SV=1	6	2	43.2
Q13283	Ras GTPase-activating protein-binding protein 1 OS=Homo sapiens GN=G3BP1 PE=1 SV=1	6	2	52.1
Q14534	Squalene monooxygenase OS=Homo sapiens GN=SQLE PE=1 SV=3	6	2	63.9
P19971	Thymidine phosphorylase OS=Homo sapiens GN=TYMP PE=1 SV=2	6	2	49.9
Q6ZT21	Transmembrane protein with metallophosphoesterase domain OS=Homo sapiens GN=TMPPE PE=2 SV=2	6	2	49.4
Q06124	Tyrosine-protein phosphatase non-receptor type 11 OS=Homo sapiens GN=PTPN11 PE=1 SV=2	6	2	68.4
O94915	Protein furry homolog-like OS=Homo sapiens GN=FRYL PE=1 SV=2	5	11	339.4
A2RRP1	Neuroblastoma-amplified sequence OS=Homo sapiens GN=NBAS PE=1 SV=2	5	7	268.4
O95477	ATP-binding cassette sub-family A member 1 OS=Homo sapiens GN=ABCA1 PE=1 SV=3	5	6	254.1
Q9UIW2	Plexin-A1 OS=Homo sapiens GN=PLXNA1 PE=1 SV=3	5	6	210.9
A0A087WVT3	Acyloxyacyl hydrolase OS=Homo sapiens GN=AOAH PE=1 SV=1	5	5	77.9
O43861	Probable phospholipid-transporting ATPase IIB OS=Homo sapiens GN=ATP9B PE=2 SV=4	5	5	129.2
O94886	CSC1-like protein 1 OS=Homo sapiens GN=TMEM63A PE=1 SV=3	5	4	92.1
O95373	Importin-7 OS=Homo sapiens GN=IPO7 PE=1 SV=1	5	4	119.4
P19838	Nuclear factor NF-kappa-B p105 subunit OS=Homo sapiens GN=NFKB1 PE=1 SV=2	5	4	105.3
A0A0A0MT60	Peptidylprolyl isomerase (Fragment) OS=Homo sapiens GN=FKBP15 PE=1 SV=1	5	4	136.2
P53992	Protein transport protein Sec24C OS=Homo sapiens GN=SEC24C PE=1 SV=3	5	4	118.2
Q92900	Regulator of nonsense transcripts 1 OS=Homo sapiens GN=UPF1 PE=1 SV=2	5	4	124.3

Q5T5C0	Syntaxin-binding protein 5 OS=Homo sapiens GN=STXBP5 PE=1 SV=1	5	4	127.5
Q96AE7	Tetratricopeptide repeat protein 17 OS=Homo sapiens GN=TTC17 PE=1 SV=1	5	4	129.5
P48553	Trafficking protein particle complex subunit 10 OS=Homo sapiens GN=TRAPPC10 PE=1 SV=2	5	4	142.1
Q12768	WASH complex subunit 5 OS=Homo sapiens GN=WASHC5 PE=1 SV=1	5	4	134.2
A0A1C7CYZ0	Active breakpoint cluster region-related protein OS=Homo sapiens GN=ABR PE=1 SV=1	5	3	98.9
Q01432	AMP deaminase 3 OS=Homo sapiens GN=AMPD3 PE=1 SV=1	5	3	88.8
Q9NW15	Anoctamin-10 OS=Homo sapiens GN=ANO10 PE=1 SV=2	5	3	76.3
Q9UKQ2	Disintegrin and metalloproteinase domain-containing protein 28 OS=Homo sapiens GN=ADAM28 PE=2 SV=3	5	3	87.1
Q32MZ4	Leucine-rich repeat flightless-interacting protein 1 OS=Homo sapiens GN=LRRFIP1 PE=1 SV=2	5	3	89.2
O00308	NEDD4-like E3 ubiquitin-protein ligase WWP2 OS=Homo sapiens GN=WWP2 PE=1 SV=2	5	3	98.9
Q6ZNB6	NF-X1-type zinc finger protein NFXL1 OS=Homo sapiens GN=NFXL1 PE=1 SV=2	5	3	101.3
H3BVG0	Nuclear pore complex protein Nup93 OS=Homo sapiens GN=NUP93 PE=1 SV=1	5	3	99.5
P49757	Protein numb homolog OS=Homo sapiens GN=NUMB PE=1 SV=2	5	3	70.8
J3KPQ4	Rho GTPase activating protein 9, isoform CRA_a OS=Homo sapiens GN=ARHGAP9 PE=1 SV=1	5	3	90.5
O95352	Ubiquitin-like modifier-activating enzyme ATG7 OS=Homo sapiens GN=ATG7 PE=1 SV=1	5	3	77.9
Q96H55	Unconventional myosin-XIX OS=Homo sapiens GN=MYO19 PE=1 SV=2	5	3	109.1
P30520	Adenylosuccinate synthetase isozyme 2 OS=Homo sapiens GN=ADSS PE=1 SV=3	5	2	50.1
P22083	Alpha-(1,3)-fucosyltransferase 4 OS=Homo sapiens GN=FUT4 PE=2 SV=3	5	2	59
P02765	Alpha-2-HS-glycoprotein OS=Homo sapiens GN=AHSG PE=1 SV=1	5	2	39.3
Q92499	ATP-dependent RNA helicase DDX1 OS=Homo sapiens GN=DDX1 PE=1 SV=2	5	2	82.4
Q92685	Dol-P-Man:Man(5)GlcNAc(2)-PP-Dol alpha-1,3-mannosyltransferase OS=Homo sapiens GN=ALG3 PE=1 SV=1	5	2	50.1
O43854	EGF-like repeat and discoidin I-like domain-containing protein 3 OS=Homo sapiens GN=EDIL3 PE=1 SV=1	5	2	53.7
Q9BZD2	Equilibrative nucleoside transporter 3 OS=Homo sapiens GN=SLC29A3 PE=1 SV=3	5	2	51.8
Q96BI3	Gamma-secretase subunit APH-1A OS=Homo sapiens GN=APH1A PE=1 SV=1	5	2	29
G3V1A6	Gasdermin domain containing 1, isoform CRA_d OS=Homo sapiens GN=GSDMD PE=1 SV=1	5	2	57.7
P16260	Graves disease carrier protein OS=Homo sapiens GN=SLC25A16 PE=1 SV=3	5	2	36.2
I3L0A0	HCG2044781 OS=Homo sapiens GN=TMEM189-UBE2V1 PE=3 SV=1	5	2	42.2
O00425	Insulin-like growth factor 2 mRNA-binding protein 3 OS=Homo sapiens GN=IGF2BP3 PE=1 SV=2	5	2	63.7

Q86YT9	Junctional adhesion molecule-like OS=Homo sapiens GN=JAML PE=1 SV=1	5	2	44.3
Q9UNW1	Multiple inositol polyphosphate phosphatase 1 OS=Homo sapiens GN=MINPP1 PE=1 SV=1	5	2	55
Q8IUH4	Palmitoyltransferase ZDHHC13 OS=Homo sapiens GN=ZDHHC13 PE=1 SV=3	5	2	70.8
Q8TCG2	Phosphatidylinositol 4-kinase type 2-beta OS=Homo sapiens GN=PI4K2B PE=1 SV=1	5	2	54.7
Q9Y617	Phosphoserine aminotransferase OS=Homo sapiens GN=PSAT1 PE=1 SV=2	5	2	40.4
Q9H3G5	Probable serine carboxypeptidase CPVL OS=Homo sapiens GN=CPVL PE=1 SV=2	5	2	54.1
P35659	Protein DEK OS=Homo sapiens GN=DEK PE=1 SV=1	5	2	42.6
Q969X1	Protein lifeguard 3 OS=Homo sapiens GN=TMBIM1 PE=1 SV=2	5	2	34.6
Q15435	Protein phosphatase 1 regulatory subunit 7 OS=Homo sapiens GN=PPP1R7 PE=1 SV=1	5	2	41.5
Q7Z7A4	PX domain-containing protein kinase-like protein OS=Homo sapiens GN=PXK PE=1 SV=1	5	2	64.9
Q5VZM2	Ras-related GTP-binding protein B OS=Homo sapiens GN=RRAGB PE=1 SV=1	5	2	43.2
P42331	Rho GTPase-activating protein 25 OS=Homo sapiens GN=ARHGAP25 PE=1 SV=2	5	2	73.4
Q96DX4	RING finger and SPRY domain-containing protein 1 OS=Homo sapiens GN=RSPRY1 PE=1 SV=1	5	2	64.1
Q9H3T3	Semaphorin-6B OS=Homo sapiens GN=SEMA6B PE=1 SV=4	5	2	95.2
O75886	Signal transducing adapter molecule 2 OS=Homo sapiens GN=STAM2 PE=1 SV=1	5	2	58.1
Q96K37	Solute carrier family 35 member E1 OS=Homo sapiens GN=SLC35E1 PE=1 SV=2	5	2	44.7
P35610	Sterol O-acyltransferase 1 OS=Homo sapiens GN=SOAT1 PE=1 SV=3	5	2	64.7
Q96AN5	Transmembrane protein 143 OS=Homo sapiens GN=TMEM143 PE=2 SV=1	5	2	51.7
Q08J23	tRNA (cytosine(34)-C(5))-methyltransferase OS=Homo sapiens GN=NSUN2 PE=1 SV=2	5	2	86.4
P21580	Tumor necrosis factor alpha-induced protein 3 OS=Homo sapiens GN=TNFAIP3 PE=1 SV=1	5	2	89.6
Q9H3H5	UDP-N-acetylglucosamine--dolichyl-phosphate N-acetylglucosaminephosphotransferase OS=Homo sapiens GN=DPAGT1 PE=1 SV=2	5	2	46.1
Q8TAF3	WD repeat-containing protein 48 OS=Homo sapiens GN=WDR48 PE=1 SV=1	5	2	76.2
Q04637	Eukaryotic translation initiation factor 4 gamma 1 OS=Homo sapiens GN=EIF4G1 PE=1 SV=4	4	5	175.4
M0R0P8	Unconventional myosin-IXb OS=Homo sapiens GN=MYO9B PE=1 SV=1	4	5	243.2
O14727	Apoptotic protease-activating factor 1 OS=Homo sapiens GN=APAF1 PE=1 SV=2	4	4	141.7
Q04656	Copper-transporting ATPase 1 OS=Homo sapiens GN=ATP7A PE=1 SV=3	4	4	163.3
Q14005	Pro-interleukin-16 OS=Homo sapiens GN=IL16 PE=1 SV=4	4	4	141.7
F8W6H6	Unconventional myosin-Va OS=Homo sapiens GN=MYO5A PE=1 SV=2	4	4	215.2

Q13444	Disintegrin and metalloproteinase domain-containing protein 15 OS=Homo sapiens GN=ADAM15 PE=1 SV=4	4	3	92.9
Q8N3D4	EH domain-binding protein 1-like protein 1 OS=Homo sapiens GN=EHBP1L1 PE=1 SV=2	4	3	161.8
O60763	General vesicular transport factor p115 OS=Homo sapiens GN=USO1 PE=1 SV=2	4	3	107.8
P14735	Insulin-degrading enzyme OS=Homo sapiens GN=IDE PE=1 SV=4	4	3	117.9
P22033	Methylmalonyl-CoA mutase, mitochondrial OS=Homo sapiens GN=MUT PE=1 SV=4	4	3	83.1
A0A0D9SEY1	Mitogen-activated protein kinase kinase kinase kinase 4 OS=Homo sapiens GN=MAP4K4 PE=1 SV=1	4	3	151
Q6ZMZ3	Nesprin-3 OS=Homo sapiens GN=SYNE3 PE=1 SV=2	4	3	112.1
Q6WKZ4	Rab11 family-interacting protein 1 OS=Homo sapiens GN=RAB11FIP1 PE=1 SV=3	4	3	137.1
Q16827	Receptor-type tyrosine-protein phosphatase O OS=Homo sapiens GN=PTPRO PE=1 SV=2	4	3	138.3
Q16512	Serine/threonine-protein kinase N1 OS=Homo sapiens GN=PKN1 PE=1 SV=2	4	3	103.9
Q8NBP0	Tetratricopeptide repeat protein 13 OS=Homo sapiens GN=TTC13 PE=2 SV=3	4	3	96.8
Q13263	Transcription intermediary factor 1-beta OS=Homo sapiens GN=TRIM28 PE=1 SV=5	4	3	88.5
A0AVT1	Ubiquitin-like modifier-activating enzyme 6 OS=Homo sapiens GN=UBA6 PE=1 SV=1	4	3	117.9
Q9P253	Vacuolar protein sorting-associated protein 18 homolog OS=Homo sapiens GN=VPS18 PE=1 SV=2	4	3	110.1
P54802	Alpha-N-acetylglucosaminidase OS=Homo sapiens GN=NAGLU PE=1 SV=2	4	2	82.2
Q8IZ07	Ankyrin repeat domain-containing protein 13A OS=Homo sapiens GN=ANKRD13A PE=1 SV=3	4	2	67.6
Q14161	ARF GTPase-activating protein GIT2 OS=Homo sapiens GN=GIT2 PE=1 SV=2	4	2	84.5
P08243	Asparagine synthetase [glutamine-hydrolyzing] OS=Homo sapiens GN=ASNS PE=1 SV=4	4	2	64.3
P55061	Bax inhibitor 1 OS=Homo sapiens GN=TMBIM6 PE=1 SV=2	4	2	26.5
Q9Y646	Carboxypeptidase Q OS=Homo sapiens GN=CPQ PE=1 SV=1	4	2	51.9
Q14677	Clathrin interactor 1 OS=Homo sapiens GN=CLINT1 PE=1 SV=1	4	2	68.2
O60826	Coiled-coil domain-containing protein 22 OS=Homo sapiens GN=CCDC22 PE=1 SV=1	4	2	70.7
P02748	Complement component C9 OS=Homo sapiens GN=C9 PE=1 SV=2	4	2	63.1
Q9H5V8	CUB domain-containing protein 1 OS=Homo sapiens GN=CDCP1 PE=1 SV=3	4	2	92.9
Q13619	Cullin-4A OS=Homo sapiens GN=CUL4A PE=1 SV=3	4	2	87.6
Q16678	Cytochrome P450 1B1 OS=Homo sapiens GN=CYP1B1 PE=1 SV=2	4	2	60.8
G3V180	Dipeptidyl peptidase 3 OS=Homo sapiens GN=DPP3 PE=1 SV=1	4	2	84.3
Q96KC8	DnaJ homolog subfamily C member 1 OS=Homo sapiens GN=DNAJC1 PE=1 SV=1	4	2	63.8

Q8TAG9	Exocyst complex component 6 OS=Homo sapiens GN=EXOC6 PE=1 SV=3	4	2	93.7
P51114	Fragile X mental retardation syndrome-related protein 1 OS=Homo sapiens GN=FXR1 PE=1 SV=3	4	2	69.7
E7ESP4	Integrin alpha-2 OS=Homo sapiens GN=ITGA2 PE=1 SV=1	4	2	102.8
P26010	Integrin beta-7 OS=Homo sapiens GN=ITGB7 PE=1 SV=1	4	2	86.8
F5H1S1	Mannosyltransferase OS=Homo sapiens GN=PIGB PE=1 SV=2	4	2	65.1
O60669	Monocarboxylate transporter 2 OS=Homo sapiens GN=SLC16A7 PE=1 SV=2	4	2	52.2
P17480	Nucleolar transcription factor 1 OS=Homo sapiens GN=UBTF PE=1 SV=1	4	2	89.4
Q8NDX1	PH and SEC7 domain-containing protein 4 OS=Homo sapiens GN=PSD4 PE=1 SV=2	4	2	116.2
O14523	Phospholipid transfer protein C2CD2L OS=Homo sapiens GN=C2CD2L PE=1 SV=3	4	2	76.1
P09874	Poly [ADP-ribose] polymerase 1 OS=Homo sapiens GN=PARP1 PE=1 SV=4	4	2	113
A0A1B0GW05	Probable C-mannosyltransferase DPY19L1 OS=Homo sapiens GN=DPY19L1 PE=1 SV=1	4	2	84.5
Q5VW38	Protein GPR107 OS=Homo sapiens GN=GPR107 PE=1 SV=1	4	2	66.9
Q96QE2	Proton myo-inositol cotransporter OS=Homo sapiens GN=SLC2A13 PE=1 SV=3	4	2	70.3
A0A0A6YYJ8	Putative RNA-binding protein Luc7-like 2 OS=Homo sapiens GN=LUC7L2 PE=4 SV=1	4	2	54.2
Q9P2R3	Rabankyrin-5 OS=Homo sapiens GN=ANKFY1 PE=1 SV=2	4	2	128.3
Q8WTV0	Scavenger receptor class B member 1 OS=Homo sapiens GN=SCARB1 PE=1 SV=1	4	2	60.8
P02768	Serum albumin OS=Homo sapiens GN=ALB PE=1 SV=2	4	2	69.3
Q8TCT8	Signal peptide peptidase-like 2A OS=Homo sapiens GN=SPPL2A PE=1 SV=2	4	2	58.1
Q8TCT7	Signal peptide peptidase-like 2B OS=Homo sapiens GN=SPPL2B PE=1 SV=2	4	2	64.6
F8VX04	Sodium-coupled neutral amino acid transporter 1 OS=Homo sapiens GN=SLC38A1 PE=1 SV=1	4	2	56.2
Q6ZQN7	Solute carrier organic anion transporter family member 4C1 OS=Homo sapiens GN=SLCO4C1 PE=1 SV=1	4	2	78.9
Q5VV42	Threonylcarbamoyladenosine tRNA methylthiotransferase OS=Homo sapiens GN=CDKAL1 PE=1 SV=1	4	2	65.1
Q05209	Tyrosine-protein phosphatase non-receptor type 12 OS=Homo sapiens GN=PTPN12 PE=1 SV=3	4	2	88.1
H3BRJ5	Uncharacterized protein OS=Homo sapiens PE=4 SV=1	4	2	108.6
Q5T4S7	E3 ubiquitin-protein ligase UBR4 OS=Homo sapiens GN=UBR4 PE=1 SV=1	3	9	573.5
A0A0A0MRJ7	Coagulation factor V OS=Homo sapiens GN=F5 PE=1 SV=1	3	7	252.1
Q7Z6Z7	E3 ubiquitin-protein ligase HUWE1 OS=Homo sapiens GN=HUWE1 PE=1 SV=3	3	7	481.6
Q709C8	Vacuolar protein sorting-associated protein 13C OS=Homo sapiens GN=VPS13C PE=1 SV=1	3	7	422.1

P16885	1-phosphatidylinositol 4,5-bisphosphate phosphodiesterase gamma-2 OS=Homo sapiens GN=PLCG2 PE=1 SV=4	3	3	147.8
O00203	AP-3 complex subunit beta-1 OS=Homo sapiens GN=AP3B1 PE=1 SV=3	3	3	121.2
Q6YHK3	CD109 antigen OS=Homo sapiens GN=CD109 PE=1 SV=2	3	3	161.6
O60271	C-Jun-amino-terminal kinase-interacting protein 4 OS=Homo sapiens GN=SPAG9 PE=1 SV=4	3	3	146.1
Q9H7D0	Dedicator of cytokinesis protein 5 OS=Homo sapiens GN=DOCK5 PE=1 SV=3	3	3	215.2
E9PF32	DENN domain-containing protein 3 OS=Homo sapiens GN=DENND3 PE=1 SV=1	3	3	144.2
Q02880	DNA topoisomerase 2-beta OS=Homo sapiens GN=TOP2B PE=1 SV=3	3	3	183.2
P08069	Insulin-like growth factor 1 receptor OS=Homo sapiens GN=IGF1R PE=1 SV=1	3	3	154.7
O95197	Reticulon-3 OS=Homo sapiens GN=RTN3 PE=1 SV=2	3	3	112.5
Q9ULF5	Zinc transporter ZIP10 OS=Homo sapiens GN=SLC39A10 PE=1 SV=2	3	3	94.1
Q9Y2D5	A-kinase anchor protein 2 OS=Homo sapiens GN=AKAP2 PE=1 SV=3	3	2	94.6
Q09328	Alpha-1,6-mannosylglycoprotein 6-beta-N-acetylglucosaminyltransferase A OS=Homo sapiens GN=MGAT5 PE=2 SV=1	3	2	84.5
P49641	Alpha-mannosidase 2x OS=Homo sapiens GN=MAN2A2 PE=2 SV=3	3	2	130.5
Q7Z3C6	Autophagy-related protein 9A OS=Homo sapiens GN=ATG9A PE=1 SV=3	3	2	94.4
Q14444	Caprin-1 OS=Homo sapiens GN=CAPRIN1 PE=1 SV=2	3	2	78.3
O60716	Catenin delta-1 OS=Homo sapiens GN=CTNND1 PE=1 SV=1	3	2	108.1
Q96NY7	Chloride intracellular channel protein 6 OS=Homo sapiens GN=CLIC6 PE=2 SV=3	3	2	73
P11388	DNA topoisomerase 2-alpha OS=Homo sapiens GN=TOP2A PE=1 SV=3	3	2	174.3
Q9Y2E5	Epididymis-specific alpha-mannosidase OS=Homo sapiens GN=MAN2B2 PE=1 SV=4	3	2	113.9
M0R2A0	ER membrane protein complex subunit 10 OS=Homo sapiens GN=EMC10 PE=1 SV=1	3	2	39
Q8IYD1	Eukaryotic peptide chain release factor GTP-binding subunit ERF3B OS=Homo sapiens GN=GSPT2 PE=1 SV=2	3	2	68.8
P55060	Exportin-2 OS=Homo sapiens GN=CSE1L PE=1 SV=3	3	2	110.3
O75369	Filamin-B OS=Homo sapiens GN=FLNB PE=1 SV=2	3	2	278
F8VWL3	FYVE, RhoGEF and PH domain-containing protein 4 OS=Homo sapiens GN=FGD4 PE=1 SV=1	3	2	100.9
Q96CW5	Gamma-tubulin complex component 3 OS=Homo sapiens GN=TUBGCP3 PE=1 SV=2	3	2	103.5
A0A0G2JK23	Large proline-rich protein BAG6 OS=Homo sapiens GN=BAG6 PE=1 SV=1	3	2	119.3
Q9Y2H9	Microtubule-associated serine/threonine-protein kinase 1 OS=Homo sapiens GN=MAST1 PE=1 SV=2	3	2	170.6
Q96T76	MMS19 nucleotide excision repair protein homolog OS=Homo sapiens GN=MMS19 PE=1 SV=2	3	2	113.2

Q8TF71	Monocarboxylate transporter 10 OS=Homo sapiens GN=SLC16A10 PE=1 SV=1	3	2	55.5
O15357	Phosphatidylinositol 3,4,5-trisphosphate 5-phosphatase 2 OS=Homo sapiens GN=INPPL1 PE=1 SV=2	3	2	138.5
Q13563	Polycystin-2 OS=Homo sapiens GN=PKD2 PE=1 SV=3	3	2	109.6
Q9H2G2	STE20-like serine/threonine-protein kinase OS=Homo sapiens GN=SLK PE=1 SV=1	3	2	142.6
Q6PKC3	Thioredoxin domain-containing protein 11 OS=Homo sapiens GN=TXNDC11 PE=1 SV=2	3	2	110.5
Q6ZXV5	Transmembrane and TPR repeat-containing protein 3 OS=Homo sapiens GN=TMTC3 PE=1 SV=2	3	2	103.9
Q86UB9	Transmembrane protein 135 OS=Homo sapiens GN=TMEM135 PE=2 SV=2	3	2	52.3
Q92973	Transportin-1 OS=Homo sapiens GN=TNPO1 PE=1 SV=2	3	2	102.3
Q96JC1	Vam6/Vps39-like protein OS=Homo sapiens GN=VPS39 PE=1 SV=2	3	2	101.7
Q92504	Zinc transporter SLC39A7 OS=Homo sapiens GN=SLC39A7 PE=1 SV=2	3	2	50.1
E9PAV3	Nascent polypeptide-associated complex subunit alpha, muscle-specific form OS=Homo sapiens GN=NACA PE=1 SV=1	2	3	205.3
P46531	Neurogenic locus notch homolog protein 1 OS=Homo sapiens GN=NOTCH1 PE=1 SV=4	2	3	272.3
Q14667	Protein KIAA0100 OS=Homo sapiens GN=KIAA0100 PE=1 SV=3	2	3	253.5
Q2PPJ7	Ral GTPase-activating protein subunit alpha-2 OS=Homo sapiens GN=RALGAPA2 PE=1 SV=2	2	3	210.6
Q8TDZ2	[F-actin]-methionine sulfoxide oxidase MICAL1 OS=Homo sapiens GN=MICAL1 PE=1 SV=2	2	2	117.8
Q14246	Adhesion G protein-coupled receptor E1 OS=Homo sapiens GN=ADGRE1 PE=2 SV=3	2	2	97.6
Q08211	ATP-dependent RNA helicase A OS=Homo sapiens GN=DHX9 PE=1 SV=4	2	2	140.9
A0A0C4DGG9	Chromodomain-helicase-DNA-binding protein 4 OS=Homo sapiens GN=CHD4 PE=1 SV=1	2	2	220.3
Q15021	Condensin complex subunit 1 OS=Homo sapiens GN=NCAPD2 PE=1 SV=3	2	2	157.1
Q8IZA0	Dyslexia-associated protein KIAA0319-like protein OS=Homo sapiens GN=KIAA0319L PE=1 SV=2	2	2	115.6
Q8TEX9	Importin-4 OS=Homo sapiens GN=IPO4 PE=1 SV=2	2	2	118.6
A0A1B0GU45	Lymphoid-restricted membrane protein OS=Homo sapiens GN=LRMP PE=1 SV=1	2	2	164.9
Q3SY69	Mitochondrial 10-formyltetrahydrofolate dehydrogenase OS=Homo sapiens GN=ALDH1L2 PE=1 SV=2	2	2	101.7
Q9NP73	Putative bifunctional UDP-N-acetylglucosamine transferase and deubiquitinase ALG13 OS=Homo sapiens GN=ALG13 PE=1 SV=2	2	2	126
Q8N122	Regulatory-associated protein of mTOR OS=Homo sapiens GN=RPTOR PE=1 SV=1	2	2	148.9
Q7Z6I6	Rho GTPase-activating protein 30 OS=Homo sapiens GN=ARHGAP30 PE=1 SV=3	2	2	118.5
E7EV90	Solute carrier family 45 member 4 OS=Homo sapiens GN=SLC45A4 PE=1 SV=1	2	2	88.6

Q14683	Structural maintenance of chromosomes protein 1A OS=Homo sapiens GN=SMC1A PE=1 SV=2	2	2	143.1
P50443	Sulfate transporter OS=Homo sapiens GN=SLC26A2 PE=1 SV=2	2	2	81.6
Q92545	Transmembrane protein 131 OS=Homo sapiens GN=TMEM131 PE=1 SV=3	2	2	205
O60674	Tyrosine-protein kinase JAK2 OS=Homo sapiens GN=JAK2 PE=1 SV=2	2	2	130.6
Q9Y4E8	Ubiquitin carboxyl-terminal hydrolase 15 OS=Homo sapiens GN=USP15 PE=1 SV=3	2	2	112.3
A0A0G2JL52	Valine--tRNA ligase, mitochondrial OS=Homo sapiens GN=VARS2 PE=1 SV=1	2	2	121.5
O75592	E3 ubiquitin-protein ligase MYCBP2 OS=Homo sapiens GN=MYCBP2 PE=1 SV=3	1	5	509.8
A5YKK6	CCR4-NOT transcription complex subunit 1 OS=Homo sapiens GN=CNOT1 PE=1 SV=2	1	2	266.8
A6NC98	Coiled-coil domain-containing protein 88B OS=Homo sapiens GN=CCDC88B PE=1 SV=1	1	2	164.7
A0A0G2JPR0	Complement C4-A OS=Homo sapiens GN=C4A PE=1 SV=1	1	2	192.8
Q8TDJ6	DmX-like protein 2 OS=Homo sapiens GN=DMXL2 PE=1 SV=2	1	2	339.4
P49792	E3 SUMO-protein ligase RanBP2 OS=Homo sapiens GN=RANBP2 PE=1 SV=2	1	2	358
Q38SD2	Leucine-rich repeat serine/threonine-protein kinase 1 OS=Homo sapiens GN=LRRK1 PE=1 SV=3	1	2	225.2
P21359	Neurofibromin OS=Homo sapiens GN=NF1 PE=1 SV=2	1	2	319.2
Q93008	Probable ubiquitin carboxyl-terminal hydrolase FAF-X OS=Homo sapiens GN=USP9X PE=1 SV=3	1	2	292.1
J3KNL6	Protein transport protein Sec16A OS=Homo sapiens GN=SEC16A PE=1 SV=1	1	2	251.7
A6NHR9	Structural maintenance of chromosomes flexible hinge domain-containing protein 1 OS=Homo sapiens GN=SMCHD1 PE=1 SV=2	1	2	226.2
H3BQK9	Microtubule-actin cross-linking factor 1, isoforms 1/2/3/5 OS=Homo sapiens GN=MACF1 PE=1 SV=1	0	2	860.5

Table S3: Representative host macrophage *Ehrlichia* containing phagosomal proteins detected by proteomics methods by Cheng et al. 2014, PLoS ONE 9(2): e88461. https://doi.org/10.1371/journal.pone.0088461) which were also identified in our study

**Membrane**		
P26038	Moesin	MSN
P11234	Ras-related protein Ral-B	RALB
P49795	Regulator of G-protein signaling 19	RGS19
Q8N697	Solute carrier family 15 member 4	SLC15A4
P16284	Platelet endothelial cell adhesion molecule	PECAM1
P51828	Adenylate cyclase type 7	ADCY7
A0A024R571	EH domain-containing protein 1	EHD1
P61009	Signal peptidase complex subunit 3	SPCS3
D6RE79	Major facilitator superfamily domain-containing protein 10	MFSD10
Q9UJZ1	Stomatin-like protein 2, mitochondrial	STOML2
Q96KA5	Cleft lip and palate transmembrane protein 1-like protein	CLPTM1L
**Endoplasmic reticulum (ER)**		
O94905	Erlin-2	ERLIN2
Q86UP2	Kinectin	KTN1
Q96HE7	ERO1-like protein alpha	ERO1L; ERO1A
P04844	Dolichyl-diphosphooligosaccharide--protein glycosyltransferase subunit 2	RPN2
P04843	Dolichyl-diphosphooligosaccharide--protein glycosyltransferase subunit 1	RPN1
P46977	Dolichyl-diphosphooligosaccharide--protein glycosyltransferase subunit STT3A	STT3A
Q9UBS4	DnaJ homolog subfamily B member 11	DNAJB11
P16615	Sarcoplasmic/endoplasmic reticulum calcium ATPase 2	ATP2A2
P08240	Signal recognition particle receptor subunit alpha	SRPR; SRPRA
**Cytoplasm**		
Q9BUF5	Tubulin beta-6	TUBB6
P46781	40S ribosomal protein S9	RPS9
G8JLA2	Myosin light polypeptide 6	
Q13409	Cytoplasmic dynein 1 intermediate chain 2	DYNC1I2
Q9Y376	Calcium-binding protein 39	CAB39
P55072	Transitional endoplasmic reticulum ATPase	VCP
Q15019	Septin-2	SEPT2
Q9Y3I0	tRNA-splicing ligase RtcB homolog	C22orf28; RTCB
Q96C36	Pyrroline-5-carboxylate reductase 2	PYCR2

**Mitochondrion**		
P49748	Very long-chain specific acyl-CoA dehydrogenase, mitochondrial	ACADVL
P48735	Isocitrate dehydrogenase [NADP], mitochondrial	IDH2
P28331	NADH-ubiquinone oxidoreductase 75 kDa subunit, mitochondrial	NDUFS1
Q04837	Single-stranded DNA-binding protein, mitochondrial	SSBP1
P30048	Thioredoxin-dependent peroxide reductase, mitochondrial	PRDX3
P11177	Pyruvate dehydrogenase E1 component subunit beta, mitochondrial	PDHB
Q9Y4W6	AFG3-like protein 2	AFG3L2
P83111	Serine beta-lactamase-like protein LACTB, mitochondrial	LACTB
Q99798	Aconitate hydratase, mitochondrial	ACO2
P30837	Aldehyde dehydrogenase X, mitochondrial 3	ALDH1B1
E7EPT4	NADH dehydrogenase [ubiquinone] flavoprotein 2, mitochondrial	
Q9Y305	Acyl-coenzyme A thioesterase 9, mitochondrial	ACOT9
P00403	Cytochrome c oxidase subunit 2	COX2
P53396	ATP-citrate synthase	ACLY
P07954	Fumarate hydratase, mitochondrial	FH
Q02978	Mitochondrial 2-oxoglutarate/malate carrier protein	SLC25A11
P61604	10 kDa heat shock protein, mitochondrial	HSPE1
P10809	60 kDa heat shock protein, mitochondrial	HSPD1
P82650	28S ribosomal protein S22, mitochondrial 1	MRPS22
P48047	ATP synthase subunit O, mitochondrial	ATP5O
Q9Y6C9	Mitochondrial carrier homolog 2	MTCH2
P19367	Hexokinase-1	HK1
P00367	Glutamate dehydrogenase 1, mitochondrial	GLUD1
P31040	Succinate dehydrogenase [ubiquinone] flavoprotein subunit, mitochondrial	SDHA
P34897	Serine hydroxymethyltransferase, mitochondrial	SHMT2
P24752	Acetyl-CoA acetyltransferase, mitochondrial	ACAT1
P09622	Dihydrolipoyl dehydrogenase, mitochondrial	DLD
Q8IXI2	Mitochondrial Rho GTPase 1	RHOT1
P53007	Tricarboxylate transport protein, mitochondrial	SLC25A1
O95202	LETM1 and EF-hand domain-containing protein 1, mitochondrial	LETM1
P08574	Cytochrome c1, heme protein, mitochondrial	CYC1
O00330	Pyruvate dehydrogenase protein X component, mitochondrial	PDHX

Q02252	Methylmalonate-semialdehyde dehydrogenase [acylating], mitochondrial	ALDH6A1
P13804	Electron transfer flavoprotein subunit alpha, mitochondrial	ETFA
Q9GZT3	SRA stem-loop-interacting RNA-binding protein, mitochondrial	SLIRP
J3KQ48	Peptidyl-tRNA hydrolase 2, mitochondrial	PTRH2
Q9H9B4	Sideroflexin-1	SFXN1
Q96EY7	Pentatricopeptide repeat domain-containing protein 3, mitochondrial	PTCD3
P13073	Cytochrome c oxidase subunit 4 isoform 1, mitochondrial	COX4I1
P16615	Sarcoplasmic/endoplasmic reticulum calcium ATPase 2	ATP2A2
P36776	Lon protease homolog, mitochondrial	LONP1
P11182	Lipoamide acyltransferase component of branched-chain alpha-keto acid dehydrogenase complex, mitochondrial	DBT
Q02218	2-oxoglutarate dehydrogenase, mitochondrial	OGDH
Q9Y512	Sorting and assembly machinery component 50 homolog	SAMM50
O75947	ATP synthase subunit d, mitochondrial	ATP5H
P56385	ATP synthase subunit e, mitochondrial	ATP5I
Q9Y3D9	28S ribosomal protein S23, mitochondrial	MRPS23
P21912	Succinate dehydrogenase [ubiquinone] iron-sulfur subunit, mitochondrial	SDHB
Q8N983	39S ribosomal protein L43, mitochondrial	MRPL43
Q9Y2Q9	28S ribosomal protein S28, mitochondrial	MRPS28
Q96IX5	Up-regulated during skeletal muscle growth protein 5	USMG5
O95168	NADH dehydrogenase [ubiquinone] 1 beta subcomplex subunit 4	NDUFB4
Q99714	3-hydroxyacyl-CoA dehydrogenase type-2	HSD17B10

Table S4. Representative host phagosomal proteins detected in this study that were also identified in previous studies for other obligate phagosome-tropic bacteria containing vacuoles.

Accession	Protein	Reference
E5RJR5	S-phase kinase-associated protein 1 (SKP1)	1
P55072	Transitional endoplasmic reticulum ATPase (VCP)	1
P27824	Calnexin	1
P61204	ADP-ribosylation factor 3(AFR)	1
P62820	Ras-related protein Rab-1A (Rab1)	2
Q9NX57	Ras-related protein Rab-20 (Rab2)	1 and 2
P20339	Ras-related protein Rab-5A (Rab5)	1 and 3
Q96AH8	Ras-related protein Rab-7b (Rab7)	1 and 3
P61026	Ras-related protein Rab-10 (Rab10)	1 and 2
Q15907	Ras-related protein Rab-11B (Rab11)	1 and 2
Q12846	Syntaxin-4 (STX4)	1 and 4
O00161	Synaptosomal-associated protein 23(SNAP23)	1 and 4
O60493	Sorting nexin-3(SNX5)	1 and 4
O43707	Alpha-actinin-4 (ACTN4)	1
O75396	Vesicle-trafficking protein (SEC22b)	1
P42356	Phosphatidylinositol 4-kinase alpha(PI4KA)	1

1. Bruckert WM, Kwaik YA. 2015. Complete and ubiquitinated proteome of the *Legionella*-Containing Vacuole within human macrophages. J Proteome Res 14(1): 236–248. https://doi.org/10.1021/pr500765x

2. Cheng Y, Liu Y, Wu B, Zhang J-z, Gu J, Liao Y-l, et al. 2014 Proteomic analysis of the *Ehrlichia chaffeensis* phagosome in cultured DH82 Cells. PLoS ONE 9(2): e88461. https://doi.org/10.1371/journal.pone.0088461

3. Zerial M, McBride H. Rab proteins as membrane organizers. 2001. Nat Rev Mol Cell Biol. 2:107–117. **https:/**/doi: 10.1038/35052055.

4. Herweg J, Hansmeier N, Otto A, Geffken AC, Subbarayal et al. 2015. Purification and proteomics of pathogen-modified vacuoles and membranes. Front Cell Infect Microbiol 5: 48. https://doi: 10.3389/fcimb.2015.00048.

Table S5. Representative host phagosomal proteins detected by non-proteomics methods that were also identified in this study.

**Proteins**	**Detection method**	**Reference as**
Cathepsin D, Rab7, NSF, SNAP, SNAP23, P38 MapK, Rab11, TACO, Annexin THP-1, PI3K THP-1.	Western blotting	1
Rab10, Calnexin	Microscopy	1
Rab5, Transferrin, Rab14, Rab21, Rab22, CD1	Fluorescent microscopy	1
SYNGR2, Rab8A, Rab3, STX, STIM1L, SEC22b.	immunofluorescence microscopy	2

1. Li Q, Jagannath C, Rao PK, Singh CR, Lostumbo G. 2010. Analysis of phagosomal proteomes: from latex-bead to bacterial phagosomes. Proteomics 10(22):4098-116. https://doi: 10.1002/pmic.201000210.

2. Hartlova A, Krocova Z., Cerveny L, Stulik,J. 2011. A proteomic view of the host-pathogen interaction:the host perspective. Proteomics 11, 3212–3220. https://doi: 10.1002/pmic.201000767.
